# Prioritization of anti-malarial hits from nature: chemo-informatic profiling of natural products with in vitro antiplasmodial activities and currently registered anti-malarial drugs

**DOI:** 10.1186/s12936-016-1087-y

**Published:** 2016-01-29

**Authors:** Samuel Ayodele Egieyeh, James Syce, Sarel F. Malan, Alan Christoffels

**Affiliations:** South African Medial Research Council Bioinformatics Unit, South African National Bioinformatics Institute, University of the Western Cape, Bellville, Cape Town, South Africa; School of Pharmacy, University of the Western Cape, Bellville, Cape Town, South Africa

**Keywords:** Natural products, Antiplasmodial, Hit, Chemo-informatics, Profiling, Prioritization, Anti-malarial drugs

## Abstract

**Background:**

A large number of natural products have shown in vitro antiplasmodial activities. Early identification and prioritization of these natural products with potential for novel mechanism of action, desirable pharmacokinetics and likelihood for development into drugs is advantageous. Chemo-informatic profiling of these natural products were conducted and compared to currently registered anti-malarial drugs (CRAD).

**Methods:**

Natural products with in vitro antiplasmodial activities (NAA) were compiled from various sources. These natural products were sub-divided into four groups based on inhibitory concentration (IC_50_). Key molecular descriptors and physicochemical properties were computed for these compounds and analysis of variance used to assess statistical significance amongst the sets of compounds. Molecular similarity analysis, estimation of drug-likeness, in silico pharmacokinetic profiling, and exploration of structure–activity landscape were also carried out on these sets of compounds.

**Results:**

A total of 1040 natural products were selected and a total of 13 molecular descriptors were analysed. Significant differences were observed among the sub-groups of NAA and CRAD for at least 11 of the molecular descriptors, including number of hydrogen bond donors and acceptors, molecular weight, polar and hydrophobic surface areas, chiral centres, oxygen and nitrogen atoms, and shape index. The remaining molecular descriptors, including clogP, number of rotatable bonds and number of aromatic rings, did not show any significant difference when comparing the two compound sets. Molecular similarity and chemical space analysis identified natural products that were structurally diverse from CRAD. Prediction of the pharmacokinetic properties and drug-likeness of these natural products identified over 50 % with desirable drug-like properties. Nearly 70 % of all natural products were identified as potentially promiscuous compounds. Structure–activity landscape analysis highlighted compound pairs that form ‘activity cliffs’. In all, prioritization strategies for the NAA were proposed.

**Conclusions:**

Chemo-informatic profiling of NAA and CRAD have produced a wealth of information that may guide decisions and facilitate anti-malarial drug development from natural products. Articulation of the information provided within an interactive data-mining environment led to a prioritized list of NAA.

**Electronic supplementary material:**

The online version of this article (doi:10.1186/s12936-016-1087-y) contains supplementary material, which is available to authorized users.

## Background

Malaria is a major health burden in several developing countries and imposes a huge strain on health systems, particularly in Africa where over 50 % of the malaria deaths occur in children under the age of five and pregnant women [1, 2]. The emergence of drug-resistant *Plasmodium falciparum* strains, a major causative organism of malaria, has led to increasing numbers of fatal cases [1, 2]. Consequently there is an urgent need to discover or design new anti-malarial drugs with mechanism of actions that will circumvent the current resistance profile of *P. falciparum.* Arguably, natural products from plants (phytochemicals) have been the most consistent and successful source and template of anti-malarial drugs [3]. Starting with quinine (from the bark of *Cinchona*) [4] to artemisinin (from *Artemisia annua*) [5], natural plant products have provided an invaluable armament against malaria infection. More promising is the fact that the literature revealed an increasing number of natural products, from ethnomedicine in malaria-endemic regions, with good in vitro and/or in vivo antiplasmodial activities [6–9]. Yet, many of these natural products have not made it into or made much progress down the anti-malarial drug development pipeline [10, 11]. Therefore there is a dire need to begin the process of identifying natural products with potential for anti-malarial drug discovery.

Perhaps a pertinent question to ask in light of the high cost, long duration and high failure rate of drug discovery [12–15] is: Should natural products with in vitro antiplasmodial activities (NAA) that are most likely to be successfully developed into anti-malarial drug candidates be prioritized? One approach that may help prioritize such natural products is chemo-informatics profiling. Chemo-informatics integrates chemical information with biological information [16] and translate such information into knowledge that could be used to assist decision making in the area of compound prioritization, selection, optimization, and ultimately clinical development [17]. Chemo-informatics profiling of natural products with antiplasmodial activities, hereafter referred to as NAA, may allow researchers to prioritize and select NAA for the next stage of anti-malarial drug development. Conceivably, what may be more informative is to carry out such profiling with reference to currently registered anti-malarial drugs (CRAD). Currently registered anti-malarial drugs, which have successfully passed through all the drug development hurdles, have molecular descriptors and physicochemical properties to which NAA need to conform or, more interestingly, deviate.

Chemo-informatic profiling of NAA and CRAD was conducted. Specific approaches used included comparison of key molecular descriptors and physicochemical properties of NAA and CRAD, molecular similarity/diversity analysis, exploration of structure–activity landscape, estimation of drug-likeness, bioavailability, and toxicity profile. Literature search showed no report of chemo-informatic profiling of NAA in comparison with CRAD. The results from this analysis may provide insight into the important molecular features that define the reported in vitro antiplasmodial activities, potential for good bioavailability (which is essential for in vivo assay), toxicity liabilities and structural-activity relationships that may prioritize promising NAA. Such knowledge may expedite the progress of NAA for anti-malarial drug development.

## Methods

### Data collection and preparation of dataset

The trivial name, source, chemical class and antiplasmodial activity (IC_50_) of NAA were retrieved from published articles [6–9], MSc and PhD theses, textbook chapters, collaborative drug discovery (CDD) database [18], ChEMBL and PubChem (see Additional file 1). A total of 1040 NAA were selected based on availability of chemical structure and bioactivity data (C_50_) (see Additional file 2). The selected NAA were sub-divided into four categories based on a normalized IC_50_ (pIC_50_): highly active (HA) with IC_50_ less than 1 µM (pIC_50_ >0), active (A) with IC_50_ equal or greater than 1 µM but less than 5 µM (pIC_50_ ≤ 0 > −0.7), moderately active (MA) with IC_50_ equal or greater than 5 µM but less than 10 µM (pIC_50_ ≤ −0.7 but > −1) and low active(N) with IC_50_ equal or greater than 10 µM (pIC_50_ ≤ −1). The chemical structures of these NAA were downloaded from PubChem and ChEMBL databases in two dimensional (2D) SDF format. The NAA that were not found in public chemical databases were drawn with GchemPaint chemical structure editor for Linux and exported in molfile format. All chemical structures were combined and duplicates removed according to InChIKey generated by Open Babel [19]. CRAD were retrieved from ChEMBL (name and smiles format). Three dimensional (3D) structures were generated for all the compounds from either 2D SDF, smiles or molfile formats (using builder module), corrected and minimized (using MMFF94 force field) with Molecular Operating Environment (MOE) 2013 software [20].

### Calculation of molecular descriptors and physicochemical properties

The QuSAR module of the MOE package [20] was employed to calculate structure-related 2D molecular descriptors. Other physicochemical properties (e.g., ligand efficiency (LE), number chemical functional groups) were computed with ICM Chemist Pro (v3.7) from Molsoft Inc. and DataWarrior [21] running on a Linux platform on a Dell Vostro 2520 computer. Boxplots of the molecular descriptors and physicochemical properties were plotted for NAA (HA, A, MA, N) and CRAD using DataWarrior. The mean of the molecular descriptors and physicochemical properties for NAA (HA, A, MA, N) and CRAD were compared and statistical differences assessed with analysis of variance (ANOVA) with significance set at p < 0.05. Furthermore, the association between the in vitro antiplasmodial activities (pIC_50_) of NAA and the molecular descriptors and physicochemical properties were assessed using Spearman correlation coefficient (r).

## Molecular similarity/diversity analysis

### MoSS most common substructure (MoSSMCSS)

**Fig. 1:**
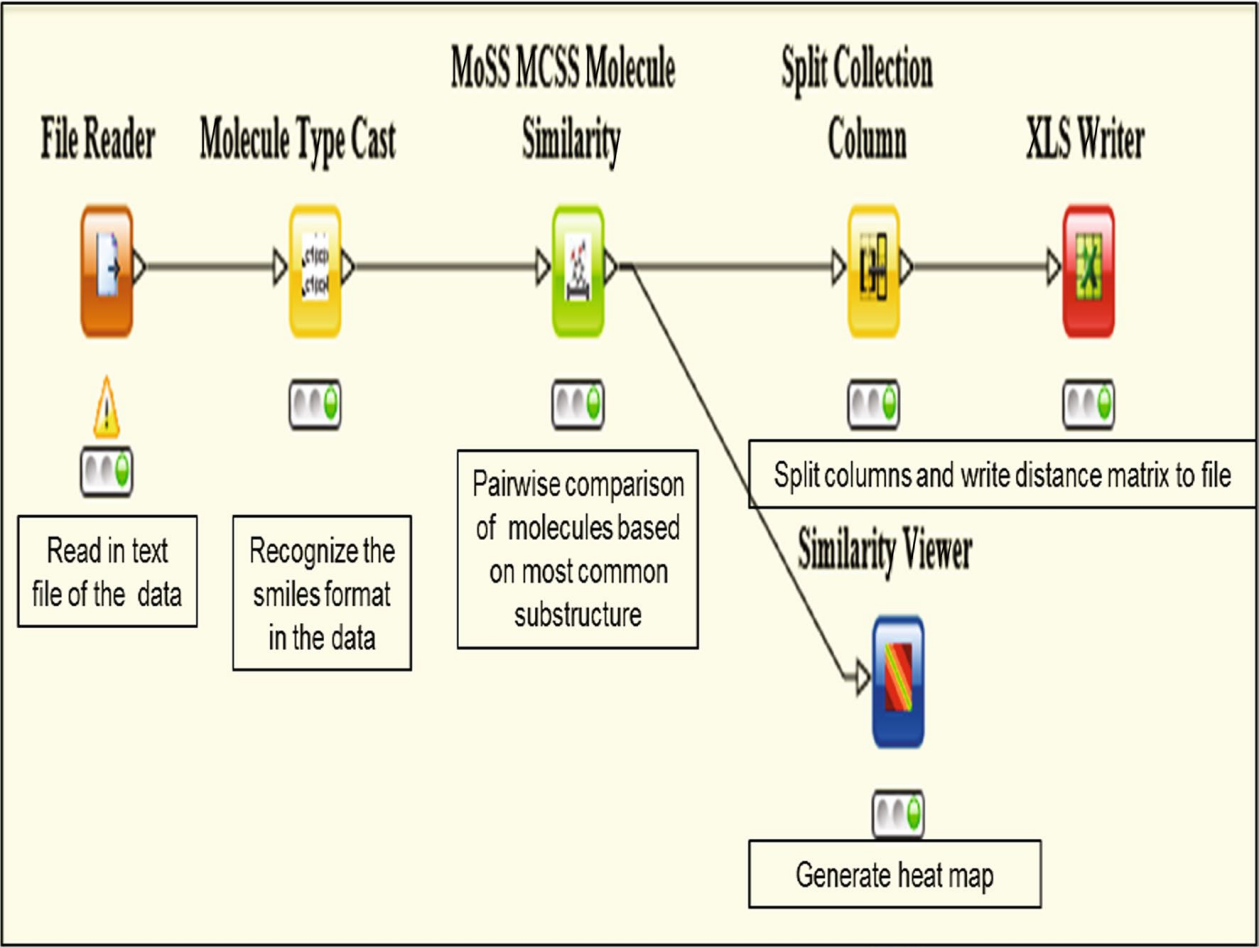
KNIME workflow to assess molecular similarity amongst the compound sets. A file containing smiles of the molecules was parsed into the File Reader. The Molecule Type Cast node was used to convert smiles in the file into molecule. The MoSSMCSS node was used for pair-wise comparison of all molecules. The similarity viewer generates heat maps. The molecules comparison is converted to a distance matrix and output as an Excel file with the XLS Writer

The KNIME (Konstanz Information Miner) workflow [22], shown in Fig. 1, was used to compute and visualize the molecular similarity, based on most common substructure (MoSSMCSS) [23], amongst the NAA and between NAA and CRAD. Molecular similarity within the dataset was visualized with a heat map.

### Chemical space analysis (ChemGPS-NP)

The coordinates of chemical space occupied by NAA relative to those occupied by CRAD were explored with ChemGPS-NP [24]. A text file containing the smiles and identifier for the compounds in the dataset was submitted to the ChemGPS-NP web service. The result, with four principal components added for each compound, was downloaded as a text file. The text file was then opened in DataWarrior and the four principal components plotted on a 3D scatter plot (markers were sized relative to one of the principal components).

### Analysis of structure–activity landscape (identifying activity cliffs)

In recent time, structure–activity landscape analysis was used to visualize the relationship between molecular structure and bio-activities, especially in large activity screening datasets [25]. The structure–activity landscape analysis was conducted on NAA using DataWarrior [21]. Similarity/activity cliff analysis was chosen from the Chemistry menu. SkelSpheres descriptors were used as similarity criteria for arranging molecules on a 2D self-organizing maps (SOM). The activity column containing the numeric value of IC_50_ of the NAA was selected to calculate the structure–activity landscape index (SALI). For any couple of molecules, the SALI value reflects how much activity is gained with a small modification of the chemical structure. The hit status, i.e., NAA sub-group (A, HA, MA, N) was chosen as the Identifier column. Similarity limit was set at 0.8 (Tanimoto coefficient). Visualization based on similarity relationships were created (markers were coloured by activity).

### Prediction of pharmacokinetic properties and drug-likeness of NAA and CRAD

Four models were used to characterize the bio-availability profile of NAA and CRAD namely: Lipinski’s rule of five [26], Egan Egg [27], golden triangle [28] and a model proposed by Veber et al. [29]. For the Lipinski’s rule of five, a 3D plot was constructed in DataWarrior for NAA and CRAD using the number of hydrogen bond donors (HBD) and acceptors (HBA), calculated logarithm of partition coefficient (clogP) and molecular weight (MW). Markers on the plot were sized by molecular weight. The filters for the descriptors (in Lipinski’s rule of five) were then adjusted, in the DataWarrrior software [21], to the cut-off proposed by the Lipinski’s model (HBD <5, HBA <10, clogP <5, MW <500). The proportion of NAA and CRAD that fell within these limits were retained.

ICM Chemist Pro (v3.7) from Molsoft Inc. was used to spot compounds with potential for good, borderline and poor absorption based on the Egan Egg model [27]. A plot of the clogP *versus* polar surface area (PSA) was also generated, using DataWarrior, to interactively visualize the proportion of the compounds in each sub-group of NAA and CRAD that fall within the Egan Egg (PSA <131.6 Å^2^, clogP < 5.88).

Johnson et al. [28] proposed a golden triangle, with a base between −2 and 5 for logD (pH 7.4) and peak at 500 Daltons, which enclose compounds with potential for good absorption and low clearance. This golden triangle was superimposed on a plot of logD (pH 7.4) *versus* MW for the sub-groups of NAA and CRAD. Compounds enclosed within the golden triangles were enumerated and recorded for each sub-group of NAA and CRAD.

For the model proposed by Veber et al. [29], a plot of PSA against number of rotatable bonds (NRB) for the sub-groups of NAA and CRAD was plotted in DataWarrior. The DataWarrior filters were then set to the cut-off proposed by the model (PSA [140 Å^2^] and NRB (10)). The proportion of compounds, in sub-groups of NAA and CRAD, within the area bounded by this cut-off were then tallied and noted.

The toxicity risk of NAA and CRAD was assessed with DataWarrior (from open molecules). The toxicity assessment was based on a search for reported toxic substructures or ‘toxicophores’ in the NAA. The collection of toxicophores in the software used for the prediction of toxicity (DataWarrior) were obtained by shredding compounds in the Registry of Toxic Effects of Chemical Substances (RTECS^®^). The toxicity information appearing in the Registry is derived from reports of the toxic effects of chemical substances. Registry of Toxic Effects of Chemical Substances (RTECS^®^) consists of tabulations of the lowest dose reported to have caused the listed toxic effect in the designated species (including mammalian cells) by the designated route of administration. The result was presented as a Table with each compound tag with none, low and high risk for mutagenic, tumorigenic and irritant toxicity class. Nasty or reactive chemical functions identified by the DataWarrior from each compound were also inserted into the table of results.

Fragment-based drug-likeness and quantitative estimate of drug-likeness(QED) were calculated with DataWarrior and ICM Chemist Pro (v3.7), respectively.

### Frequent hitters (promiscuous compounds)

MedChem rules [30], a set of 275 rules, was also used to identify compounds that may interfere with bioassays to produce ostensible activity (false positives). ICM Chemist Pro (v3.7) software from Molsoft Inc. was used to flag such compounds.

## Results

In this study, the approach to prioritizing the selected 1040 published NAA included calculation of molecular descriptors, calculation of LE metrics, assessment of structural similarity, overview of structural-activity landscape, pharmacokinetic profiling, toxicity profiling, and identification of frequent hitters. All these were done in relation to CRAD.

### Description of dataset

A description of the dataset used in this study is presented here. A total of 1040 NAA and 27 CRAD were included in the dataset. With regards to in vitro antiplasmodial bioactivity (IC_50_) described earlier in Methods, NAA consist of 21 % HA compounds, 28 % A compounds, 20 % MA, and 31 % N compounds. Concerning chemical class, a trend was observed between the prevalence of chemical class and bioactivity of NAA. The three most prevalent chemical class of compounds for each bioactivity category of NAA were: alkaloids (40 %), diterpenes (9 %) and flavonoids (9 %) for HA; alkaloids (34 %), terpenes (14 %) and flavonoids (13.5 %) for A; flavonoids (27 %), triterpenes (19 %) and alkaloids (15 %) for MA; triterpenes (30 %), alkaloids (25 %) and flavonoids (19 %) for N. The proportion of alkaloid compounds in NAA seems to decrease as the antiplasmodial potency decreases (i.e., most prevalent in HA bioactivity category). In contrast, percentage of flavonoids and the terpenoids (terpenes, diterpenes and triterpenes) appear to increase as the antiplasmodial activity decreases. This suggests that alkaloids may be an important chemical class for antiplasmodial activity.

### Molecular descriptors and physicochemical properties of NAA and CRAD

In this section, the distribution and summary statistics of selected molecular descriptors and physicochemical properties of NAA and CRAD were determined and assessed as a contribution towards the prioritization of NAA.

### Number of hydrogen bond acceptors and donors

**Fig. 2:**
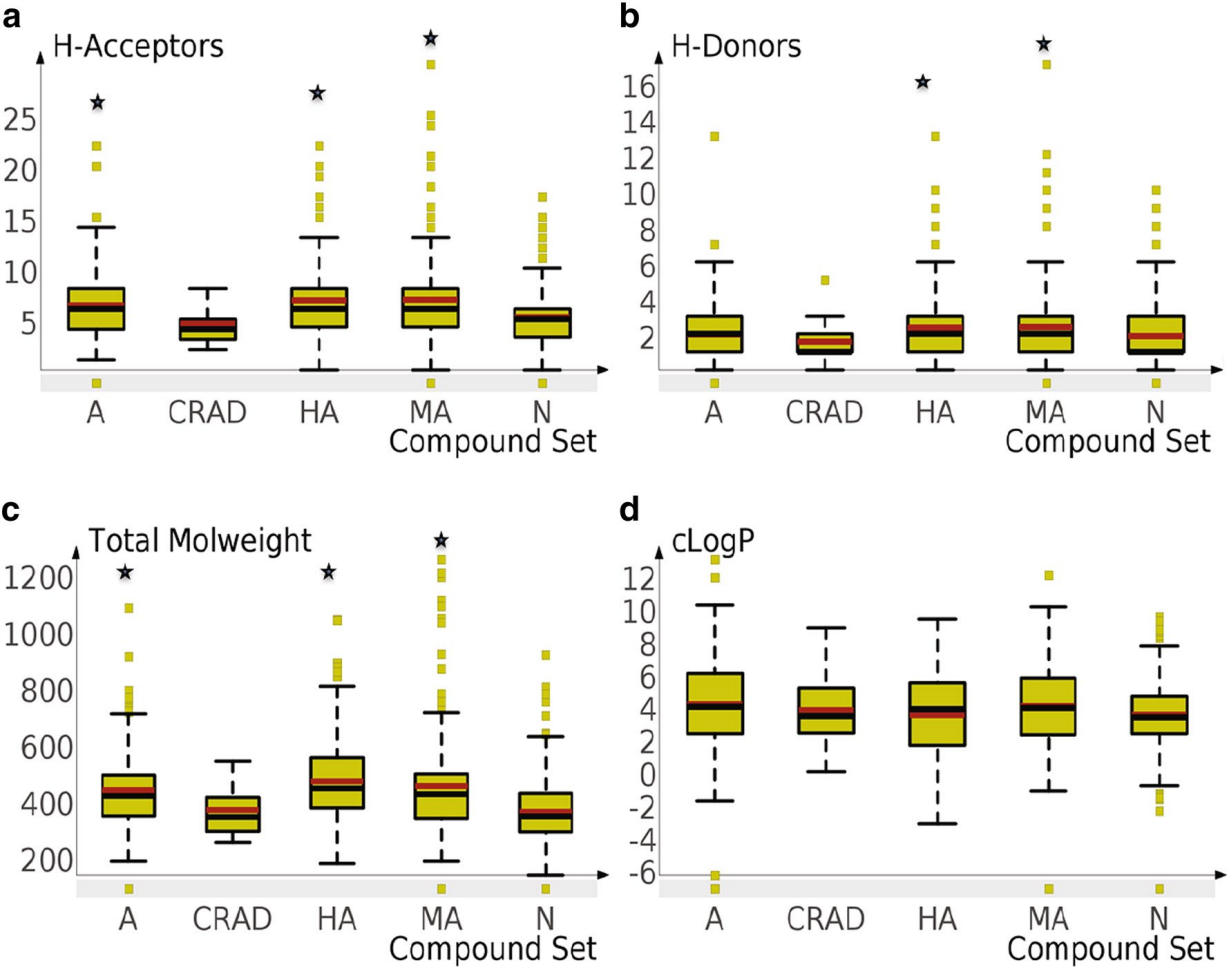
Boxplots showing distribution and summary statistics of key molecular descriptors for compound sets. Panel** a**,** b**,** c** and** d** represents the number of HBA, number of HBD, total MW and cLogP, respectively, for CRAD and sub-groups of NAA (HA, A, MA and N). The *red* and *black lines* represent the mean and median, respectively, for each distribution. Significant difference (p < 0.05) between CRAD and sub-groups of NAA are highlighted by *black asterisk* (*)

HBA and HBD are essential to mediate interactions between compounds and biochemical macromolecules. They are also determinants of oral absorption of compounds [26]. The results (Fig. 2, panel a) showed that the average number of HBA was similar for the A, HA and MA sub-groups of NAAs (median = 6). However, HBA was significantly lower in CRAD (median, 4) and low active sub-group of NAA (N) (median = 5 p < 0.05) in comparison to other sub-groups of NAA. In all cases the numbers of HBD (Fig. 2, panel b) were lower than the HBA. The HBD was significantly higher in the HA and MA than CRAD (p < 0.05). This observation aligned with other studies that report predominance of HBA and HBD in natural products over synthetic compounds [31–33]. The preponderance of strongly electronegative atoms (especially oxygen) in NAA may be responsible for the higher numbers of HBD and HBA [31–34]. In addition, there was very small negative correlation between the number of HBA (r = −0.20) and HBD (r = −0.10) and antiplasmodial activities (IC_50_) of NAA. This suggests the need for HBD and HBA for bioactivities. In conclusion, the results showed that the number of HBA and HBD was higher in the NAA compared to CRAD and showed slight association with in vitro antiplasmodial activities.

### Total molecular weight (TMW)

A low MW (less than 500 Daltons) is usually favoured because of its impact on oral absorption of compounds [26]. However, some natural products with MW >500 Daltons have been absorbed via the biological membrane [35]. From the results, the average computed total molecular weight (TMW) was similar for HA (462.47), A (430.93) and MA (447.26) but significantly different (p value < 0.05) from N and CRAD (Fig. 2, panel c). This result attests to the greater structural complexity of NAA over CRAD. A slight negative correlation (r = −0.303) was observed between TMW and antiplasmodial activities of the NAA i.e., most active (HA) showed the highest TMW. Although it has been demonstrated that the median MW of oral drugs has increased substantially over the past years with about 2 % having MW >500 Daltons [36], a TMW below 500 may be desirable for hit compounds that are yet to be optimized into lead compounds.

### Calculated logarithm of partition coefficient (clogP)

The clogP of a compound is a vital consideration for oral absorption [26] ] and it also influences binding to biological targets [37]. From the results (Fig. 2, panel d), there was no significant difference in the clogP of CRAD and NAA (HA, A, MA, N). Given that the average clogP value observed for all the CRAD and up to 70 % of NAA were lower than five, as prescribed by Lipinski’s rule of five [26], it is expected that these NAA may show good oral absorption profile comparable to the CRAD. However Lipinski’s rule of five has been reported to have exceptions, predominantly in natural products [26, 38]. It is therefore expected that NAA with clogP > 5 may also have suitable oral absorption.

In addition, clogP have been reported to be predictive of bioactivity [37]. Yet, correlation between clogP and the activity profiles of the NAA was negligible (r = −0.05). The inability of clogP to discriminate among the activity sub-groups of NAA highlights the contributions of other molecular properties to the observed antiplasmodial activity among the NAA. In view of the prospect for development into drug candidates, studies have shown that the clogP of molecules that make it all the way to commercialization have remained in the same range (2.6) for a number of years and that optimal range for clogP between one and three is vital for desirable physicochemical properties [41]. From the results, though the average clogP of the NAA was similar to CRAD, it was however higher than the 2.6 reported for commercialized drugs [39, 40]. Given that the logP of hits and leads increase as they move down the drug development pipeline [39] and that high logP [>5] comes with some liabilities, e.g., receptor promiscuity [42, 43], poor metabolic clearance [41] and toxicity [44, 45], it is desirable to select NAA with minimal clogP values to ensure that eventual anti-malarial leads and drug candidates have clogP less than four [46] or less than five [26].

### Number of rotatable bonds

A minimal NRB is desired for a NAA that may be prioritized and selected for development. The results (see Additional file 3) showed no significant difference (p > 0.05) in the average NRB between CRAD and the NAA. Although the NRB of HA (median, 3) was lower than that detected for A (median, 4) and MA (median, 4), no significant correlation was observed between NRB and bioactivity of the NAA (r = 0.03). NRB and PSA have been reported as determinants of oral absorption in a rat model [29] and specifically, compounds which meet the criterion of ten or fewer NRB are predicted to have good oral bioavailability [29]. Glaxo SmithKline (GSK) has also devised the rule of seven rotatable bonds: “Less than seven rotatable bonds are essential for good bioavailablity” [47]. Based on Veber et al. criteria [29], the results revealed that 92 % of HA had less than ten NRB, which is similar to 96.2 % observed in CRAD. Using the GSK criterion, a higher percentage of HA (81.27 %) had less than seven NRB in comparison to CRAD (70.3 %). This result attests to the potential of NAA to have good oral absorption.

### Total polar surface area

Total polar surface area (TPSA) has also been implicated as a predictor for gastro-intestinal tract (GIT) penetration by many investigators [47, 48], and may be a key factor to consider during prioritization of NAA for lead development. The TPSA of CRAD was significantly lower (p < 0.05) than the TPSA of sub-groups of NAA (see Additional file 3). Among the latter, the highest TPSA was observed in MA followed by the HA, A and N groups. There was no significant correlation between TPSA and the activity of the NAA (r = 0.13). According to Veber et al. [29], the upper limit for TPSA for a molecule to penetrate the GIT is around 140 Å^2^.

All of the CRAD, up to 75 % of the HA and over 75 % of A compounds fell below the upper limit for the TPSA. This implies that most of the active NAA are expected to have good oral absorption, which is particularly relevant during the next stage of in vivo anti-malarial assessment. The high number of polar groups (hydroxyl and carbonyl groups) may be responsible for the high TPSA observed in some of the NAA. In addition, TPSA may also determine the extent of plasma protein binding [49]. Albumin is involved in binding of mainly polar compounds [50], and high TPSA resulting from ionization of polar groups (e.g., acidic groups) may increase plasma protein binding. The higher a compound is bound to plasma protein the lower the proportion of the compound free for therapeutic effect. It is likely that some NAA that have good activity in in vitro assays may show poor activity in in vivo assays due to high plasma protein binding resulting from high TPSA. Optimization strategies that are geared towards reduction of TPSA (e.g., methylation of hydroxyl groups)may improve the oral absorption, reduce plasma protein binding and consequently improve bioactivity in vivo.

### Van der Waals hydrophobic surface areas of hydrophobic atoms (vsa_hyd)

The Van der Waals hydrophobic surface area of hydrophobic atoms (vsa_hyd) measures the level of hydrophobicity of compounds. The vsa_hyd of HA and MA of NAA were significantly higher (p < 0.05) than that of CRAD (see Additional file 3). The vsa_hyd of N was lower than the vsa_hyd of CRAD. The vsa_hyd of the active NAA (A, HA, MA) were significantly (p < 0.05) higher than the low active NAA (N) suggesting that hydrophobicity may be vital for bioactivity. This observation was corroborated by the slight negative correlation (r = −0.231) seen between vsa_hyd and IC_50_ of NAA.

Hydrophobicity determines many biological processes, such as transport, distribution, metabolism, and molecular interactions of biological molecules. It is reported that the binding affinity and drug efficacy can be optimized and increased by incorporating hydrophobic groups [51, 52]. It is estimated that addition of a methyl group will lead to a 3.5-fold increase in binding constant [53]. Therefore, moderately active NAA (MA) may be optimized as described above to improve bioactivity. Notably, improved hydrophobic interactions may also increase incidence of side effects and toxicity [54]. With regard to absorption of bioactive compounds via biological membrane, hydrophobicity is also a key factor in various absorption models, e.g., Lipinski’s rule of five [26]. Poor absorption or permeation is more likely for compounds with low hydrophobicity and the NAA that possess a higher vsa_hyd (hydrophobicity) than CRAD may be expected to show good absorption and permeation.

Overall, this brings to fore the pivotal role of hydrophobicity in achieving delicate balance of desirable activity, low toxicity and good absorption. Optimization of hydrophobicity of NAA towards a reference point, as observed for CRAD and as reported for marketed drugs, may be desirable to ensure successful development of these compounds.

### Number of chiral centres

Chiral centres (asymmetric) are tetrahedral atoms (usually carbons) that have four different substituents [55]. Compounds that have chiral centres are optically active and rotate the plane of polarized light to the left (levorotatory) or to the right (dextrorotatory) [55]. Such optically active pairs are referred to as enantiomers [55].

The average number of chiral centres for HA and MA were significantly higher (p < 0.05) than CRAD (see Additional file 3). Among the NAA, the average number of chiral centres showed slight negative correlation (r = −0.2) with antiplasmodial activity (IC_50_); HA had the highest average number of chiral centres and N had the least. This result suggests that a high number of chiral centres may be essential for antiplasmodial activity. This may be because a high number of chiral centres increase flexibility of compounds and their tendency for more interaction with binding site of macromolecules. Moreover, the higher number of chiral centres observed in the NAA compared to CRAD, which have been previously observed between natural products and synthetic compounds [11], suggests that more compounds in HA, A and MA may have enantiomers. Given that enantiomeric molecules may interact in a different mode with biological receptors, binding affinities can differ between enantiomers [56]. In clinical settings, enantiomers of chiral drugs can have decreased, had no, or even adverse effects [57–59]. Therefore, it is imperative to elucidate which of the enantiomers may be responsible for the observed antiplasmodial activities of the NAA and separate such enantiomers. Although the technology for the separation and analysis of chiral compounds has greatly advanced [59, 60], this may not yet be available in many laboratories in least developed countries where malaria is endemic and where these NAA may be sourced. In light of this limitation it may be necessary to prioritize NAAs without chiral centres (15 % of HA have no chiral centre) for preclinical development. Another option is to create racemic mixture (containing two enantiomers) provided the safety and efficacy can be justified as required by the Food Drug Administration of the United States of America [61]. In conclusion, most of the compounds in NAA had higher number of chiral centres than those in CRAD and the number of chiral centres seems to correlate with antiplasmodial activities of NAA.

### Number of oxygen atoms

The NAA had significantly (p value < 0.05) greater number of oxygen atoms than the CRAD (see Additional file 3). This may be because the NAA (consisting of alkaloids, terpenoids and flavonoids) are rich in oxygen atom as earlier reported for natural products [31–33, 62]. There was no significant difference (p > 0.05) in the number of oxygen (nO) among the sub-groups of NAA and little correlation of nO with bioactivity (r = −0.09). Although oxygen atoms, particularly sp^2^ -oxygen atom is important for ligands to form hydrogen bonds with receptors/enzymes [63], the low correlation with bioactivity [IC_50_] observed in the results suggests that the number of oxygen atoms may not be the sole contributing factor to the reported antiplasmodial activities of the NAA.

### Number of nitrogen atoms and amine functional groups

The average number of nitrogen atoms was significantly higher (p value < 0.05) in CRAD than NAA (see Additional file 3), in accord with previous observation for natural products [31–33], although the most prevalent chemical class in the NAA was alkaloids (which usually contain nitrogen atoms). However, the other prevalent chemical classes, terpenoids and flavonoids, have no or low number of nitrogen atoms, which may explain the lower average number of nitrogen atoms observed in NAA compared to CRAD. Among the NAA, a slight negative correlation (r = −0.17) was observed between the number of nitrogen atoms and bioactivity. This suggests that the nitrogen atom may be relevant for anti-malarial activities [64].

Most of the nitrogen atoms were present as amine functional groups. The amine groups were predominant in CRAD and some of the compounds in HA sub-group of NAA. The presence of amine groups (or basic nitrogen) in nearly all CRAD highlights its importance for anti-malarial activity [64, 65]. However, the absence of the amine group in some of the HA and A compounds suggest that other functional groups, present within NAA compounds, are also vital for good antiplasmodial activities. These non-amine functional groups may have the potential to be the basis of a new series of anti-malarial compounds. A typical example is artemisinin-based drugs, which are endoperoxides.

### Number of aromatic rings

Limited numbers of aromatic rings have been shown to improve developability of hits to drug candidates [66]. The median number of aromatic rings for CRAD and all the sub-groups of NAA were between one and two. The average number of aromatic rings for CRAD was similar to the majority of the NAA (see Additional file 3). There was no correlation between the number of aromatic rings and bioactivity of NAA (r = −0.03).

Increase in aromatic ring count correlates with decreased aqueous solubility (with attendant poor absorption) [41, 62, 66, 67], increased plasma protein binding (leading to low clearance and therapeutic efficacy) [66], increased potential for inhibition of enzyme [with attendant toxicity and drug interactions] [68], increased mean observed hERG activity [hERG toxicity] [66] and decreased chance of developability of compounds into marketed drugs [66, 69, 70]. On these premise, NAA with minimal aromatic rings are expected to possess good aqueous solubility that is essential for oral absorption and exhibit reduced plasma protein binding that may lead to a greater free fraction of such compounds in systemic circulation, particularly during in vivo assay. In addition, low potential for enzyme inhibition and minimal toxicity [including hERG toxicity] as well as potential to be developed into successful anti-malarial drug candidates may also be expected from these NAA. It is however worth noting that a high number of aromatic rings may be desirable. This is because increasing number of aromatic rings decreases entropy [random movement] of molecules and favours binding of compounds to biological targets [66, 71, 72]. Increasing the number of aromatic rings during lead optimization may be favoured as a means to increase potency. Another reason why aromatic rings are favoured in drug design programmes is because of the well-established synthetic methods to make aryl–aryl links [66], which has made it attractive to design and synthesize compounds with increased number of aromatic rings in most combinatorial libraries [73].

Overall, limiting the number of aromatic rings during optimization of these NAA will make them broadly more developable and more ‘drug-like’ despite the likelihood of aromatic rings to increase potency and be readily amenable to synthesis and transformation [66].

### Shape index

Shape index, computed with Datawarrior, is a parameter that estimates the 3D shapes of compounds. Shape index less than 0.5 suggests presence of 3D (non-flat or spherical) scaffolds while shape index greater than 0.5 is for flat scaffolds. The average shape index of CRAD (0.53) was significantly (p < 0.05) higher than that of HA (0.43), A (0.45) and MA (0.46) but not significantly (p = 0.34) different from N (0.52) (see Additional file 3). This result suggests that CRAD and N contain compounds with flat scaffolds while HA, A and MA contain compounds with spherical or non-flat scaffolds. Other authors [74, 75] have reported the predominance of non-flat compounds in natural products as observed for NAA. Among the sub-groups of NAA, only the low active (N) NAA showed mean shape index >0.5, indicating the prevalence of compounds with flat scaffolds. There was some positive correlation between the shape index and in vitro antiplasmodial activity (r = 0.319). This suggests that non-flat scaffolds may be essential for antiplasmodial activity.

Generally, the results showed significant difference (p < 0.05) between the shape indices of NAA and CRAD. The presence of non-flat scaffolds seems to be essential for antiplasmodial activity amongst the NAA.

### Synthetic feasibility [rsynth]

This parameter estimates how feasible it is to synthesize the compounds, with 1 being the most synthetically feasible and 0 the least synthetically feasible. The results (see Additional file 3) showed that all the sub-groups of NAA showed mean synthetic feasibility (rsynth) values that were significantly lower (p < 0.05) than that of CRAD (0.659). Among the NAA, there was very little positive correlation (r = 0.12) between rsynth and bioactivity (IC_50_). These results suggest that the CRAD and NAA N may be relatively easier to synthesize. The prevalence of flat and low MW (low complexity) compounds in N may be responsible for this observation. Synthetic feasibility and cost of synthesis may have significant impact on the development and eventual cost of drugs. This is particular relevant for neglected diseases in low-to-middle income countries where low-cost drugs are desired. Natural products have a high number of chiral centres that require advanced chemical synthetic techniques and chiral separation technology. This is evident in artemisinin, a recent drug of choice for malaria, which is still being sourced from the plant, *A. annua*, because its chemical synthesis, although achieved in 1983, is too expensive for commercialization [76].

### Ligand efficiency metrics

The calculation of the binding efficiency metrics for HA, A, MA, and N sub-groups of NAA are described as well as the contribution to, and impact of these metrics, on prioritization and selection of NAA to take forward into anti-malarial drug development.

### Ligand efficiency

Ligand efficiency assesses the contribution of heavy atoms in or MW of a compound to potency or binding affinity of such a compound i.e., potency or binding affinity per heavy atom/molecular weight, given by Eq. (1) [77].

1 $$LE = \varDelta G/HA$$

where *ΔG* = −*RT* ln (IC_50_/2), R = gas constant and T = absolute temperature. The unit of LE is kcal/mol/non hydrogen atom or heavy atom.

**Fig. 3:**
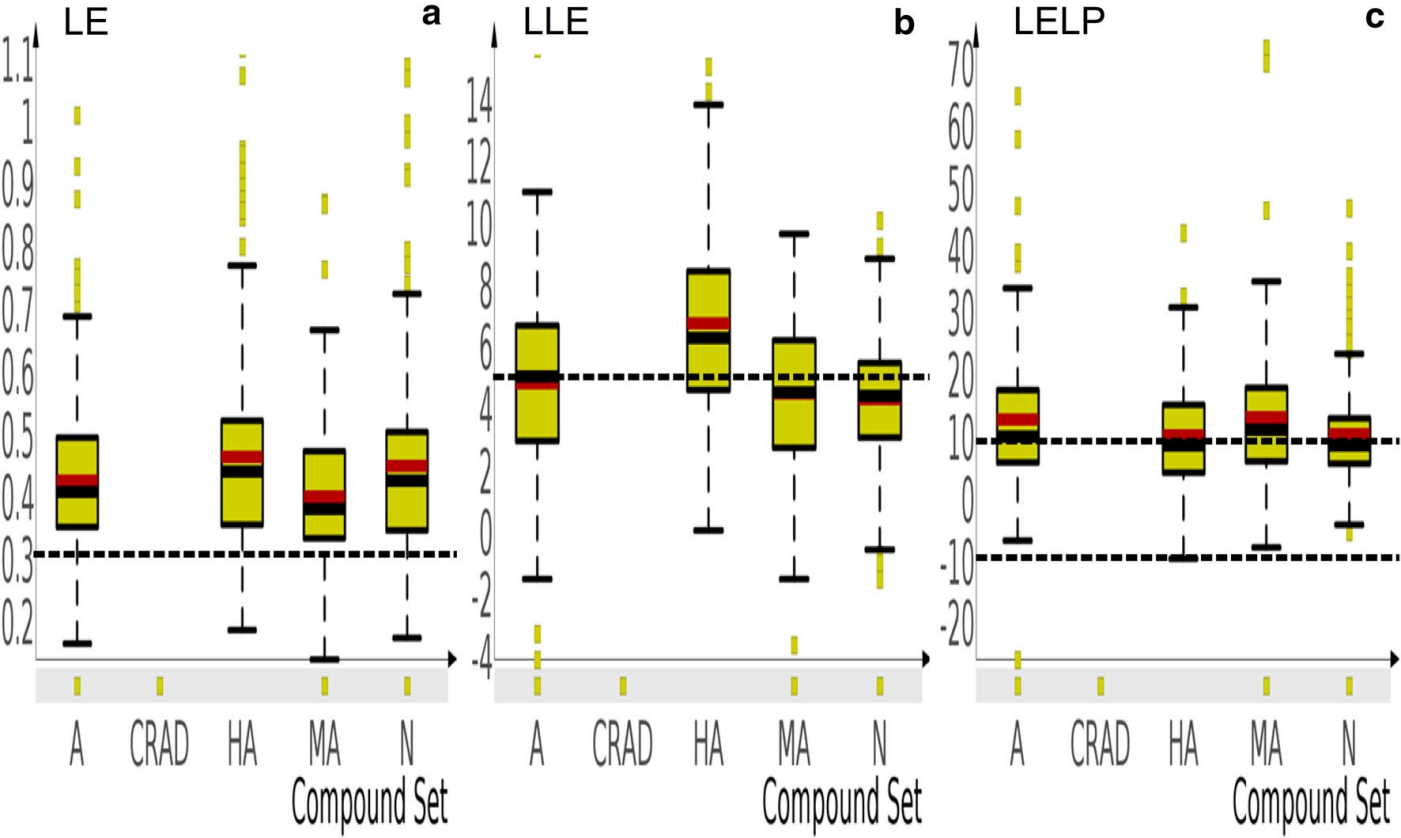
Boxplots of ligand efficiency metrics for sub-groups of natural products with in vitro antiplasmodial activity. Panel** a**,** b** and** c** represents the LE, LLE and LELP, respectively. The mean (*red line*) and median (*black line*) are indicated. CRAD were excluded from this analysis. The *horizontal black line* indicates the exemplar value cut off for each metric (LE, >0.3; LLE, >5; LELP, between 10 and 10)

Figure 3, panel a showed that the mean LE for HA (0.48) was significantly higher than that of A (0.44) and MA (0.42) but not N (0.45). The exemplar values for LE should be greater than 0.3 kcal per mole per heavy atom [78] and the results (Fig. 3, panel a) showed that the mean LE values for most of the NAA (A, HA, MA, N) were within these exemplar values. This indicates that a good proportion of NAA (up to 80 %) have desired LE (potency at the right weight). It is particularly important to identify compounds with low weight and low potency (may be present within the MA sub-group of NAA) because it has been reported that such compounds have ‘room’ for optimization to increase potency and pharmacokinetic properties without the risk of losing LE [77, 79, 80]. A downside of LE is that it does not take lipophilicity, which is an important determinant of binding and/or potency, into account in its estimation of efficiency of binding or potency [77]. Ligand lipophilicity efficiency (LLE) however provides a link between potency/binding affinity and lipophilicity.

### Ligand lipophilicity efficiency

LLE measures how efficiently a ligand/compound exploits its lipophilicity to bind to target protein or create its potency [Eq. (2)]. In other words, it evaluates how well compounds improve potency while maintaining low lipophilicity [81].

2 $$LLE = pIC_{50} {-\!\!-}clogP \, [or \, logD \, if \, the \, compound \, is \, ionizable]$$

The results (Fig. 3, panel b) showed that HA displayed a significantly higher average LLE value (HA, 6.05) than A (4.63), MA (4.14), and N (4.12). The ideal value for LLE has been reported to be greater than five [81] and only HA had a mean LLE value above five in spite of the similar clogP value with A and MA sub-groups of NAA. This suggests that the hydrophobic region of compounds in HA may be in such orientation that ensures optimal interaction with biological targets that brought about the observed bioactivity. Since the ultimate goal is to have compounds with good potency at the minimal lipophilicity, the results therefore suggests that HA consists of compounds that may be good starting points for anti-malarial drug development. Other studies have used LLE as criteria to find compounds suitable as starting points for optimization and drug development [82]. On the other hand, compounds with low potency and low lipophilicity (that may be found in the MA sub-group of NAA) have also been reported as good starting points for drug development [83, 84]. This is because such compounds have big ‘lipophilicity room’ that are generally ‘filled’ during optimization towards improved potency. Monitoring the LLE of a compound collection during optimization will also allow medicinal chemists to track the efficiency of each lipophilic addition made towards improved potency.

One limitation of LLE is that it does not account for molecular size [heavy atom or MW]. A binding efficiency metric that combine lipophilicity, molecular size and potency is the ligand efficiency dependent lipophilicity index [LELP] [85].

### Ligand efficiency dependent lipophilicity index

LELP is calculated using Eq. (3). LELP has been shown to reliably identify fragments, lead-like and drug-like compounds [77, 85]. Moreover, LELP was a better predictor of pharmacokinetic liabilities than LLE [77]. The ideal LELP values have been stated to be between -10 and 10 for acceptable leads [77].

3 $$LELP = logP/LE$$

Looking at the results (Fig. 3 panel c), average LELP obtained for HA (8.93) was significantly lower than that of A (11.46) and MA (11.81) but somewhat similar to that of N (9.09). In addition, only HA and N showed mean LELP value within the ideal range. In so much as it has been reported that compounds with LELP values outside the exemplar range may not proceed far in the drug development pipeline [86], it is anticipated that NAA that fall outside the ideal LELP range may have lower chance of success in the anti-malarial drug development process. Moreover, lead optimization strategies should aim to increase LE or reduce logP in order to bring elevated LELP values within the desired range. In addition, monitoring LELP will help to control essential physicochemical properties that will maintain desirable potency and pharmacokinetic profile during optimization [80, 84].

**Fig. 4:**
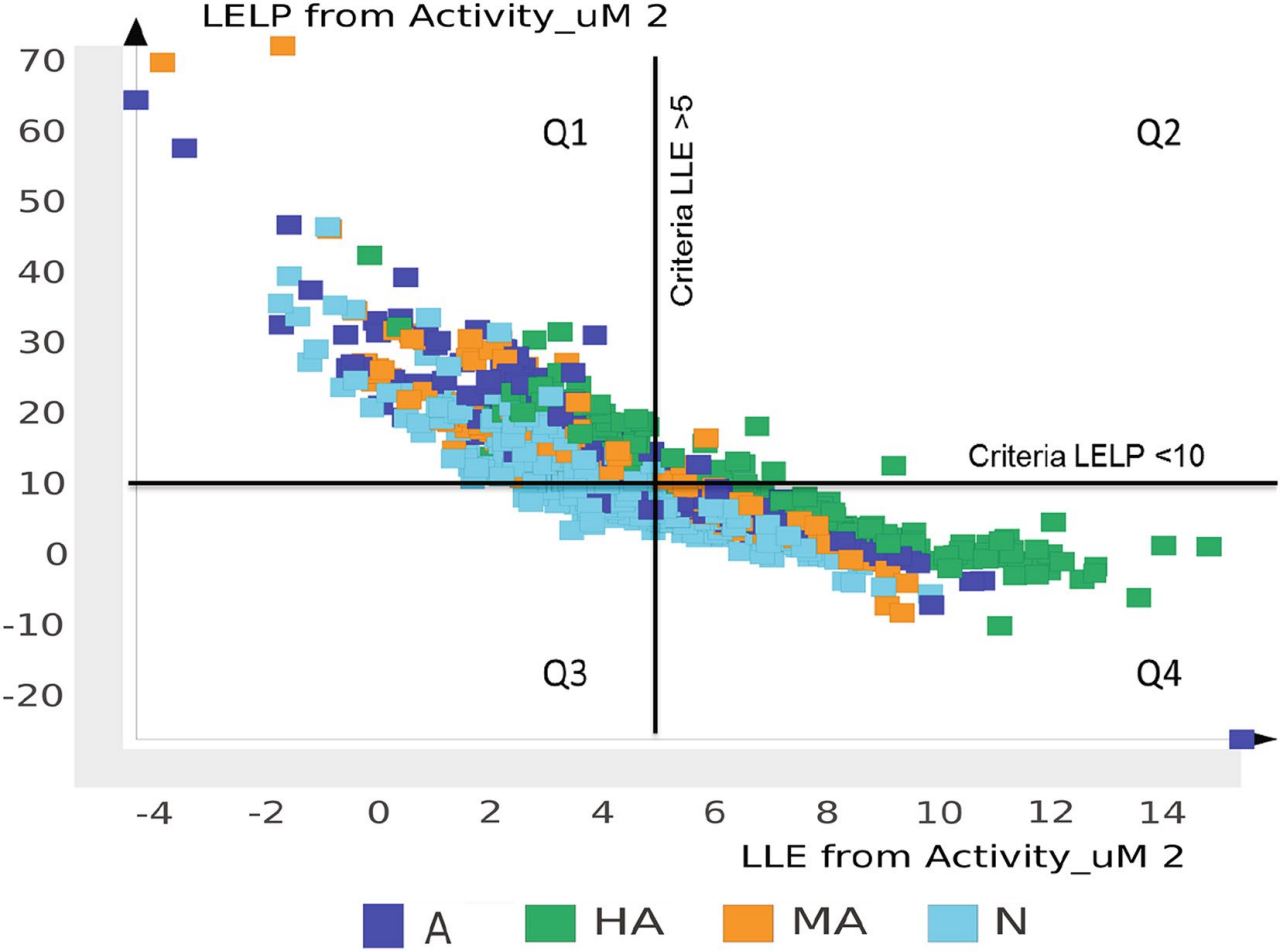
A scatter plot of LLE *versus* LELP index. The markers represent sub-groups of NAA (A, HA, MA, N). The exemplar values for LELP and LLE was used to divided the plot area into four quadrants:* Q1* (likely position for hits from bioassays and leads compounds),* Q2* (no description),* Q3* (likely position for successful leads) and* Q4* (likely position for compounds in phase 2 clinical trials and approved drugs)

In conclusion, ranking and selection of NAA from the initial list of potential antiplasmodial hits is a critical step in successful anti-malarial drug discovery [87, 88]. Given the influence of logP and molecular size [heavy atoms or MW] on potency and pharmacokinetic properties, the use of binding efficiency indices [LE, LLE, LELP] as a guiding criteria is important not only for hit selection, but also for lead generation and optimization [80, 84]. A plot of LELP against LLE (Fig. 4), previously described by Tarcsay et al. [85], may give medicinal chemists an idea of where the NAA compounds are in terms of these parameters and guide the optimization process to get the compounds to the desired region (as shown in Fig. 4). A key consideration is to be aware of the optimizing strategies that can increase potency and keep LE more or less constant or within exemplar limits.

### Molecular similarity between CRAD and NAA

Molecular similarity analysis was conducted on NAA and CRAD. The objectives of this analysis were: firstly, to examine the extent of molecular diversity within the NAA and secondly to identify NAA that are structurally similar to and diverse from CRAD. Highly active NAA that are structurally diverse from CRAD may be potentially new anti-malarial agents with novel mechanism of actions.

**Fig. 5:**
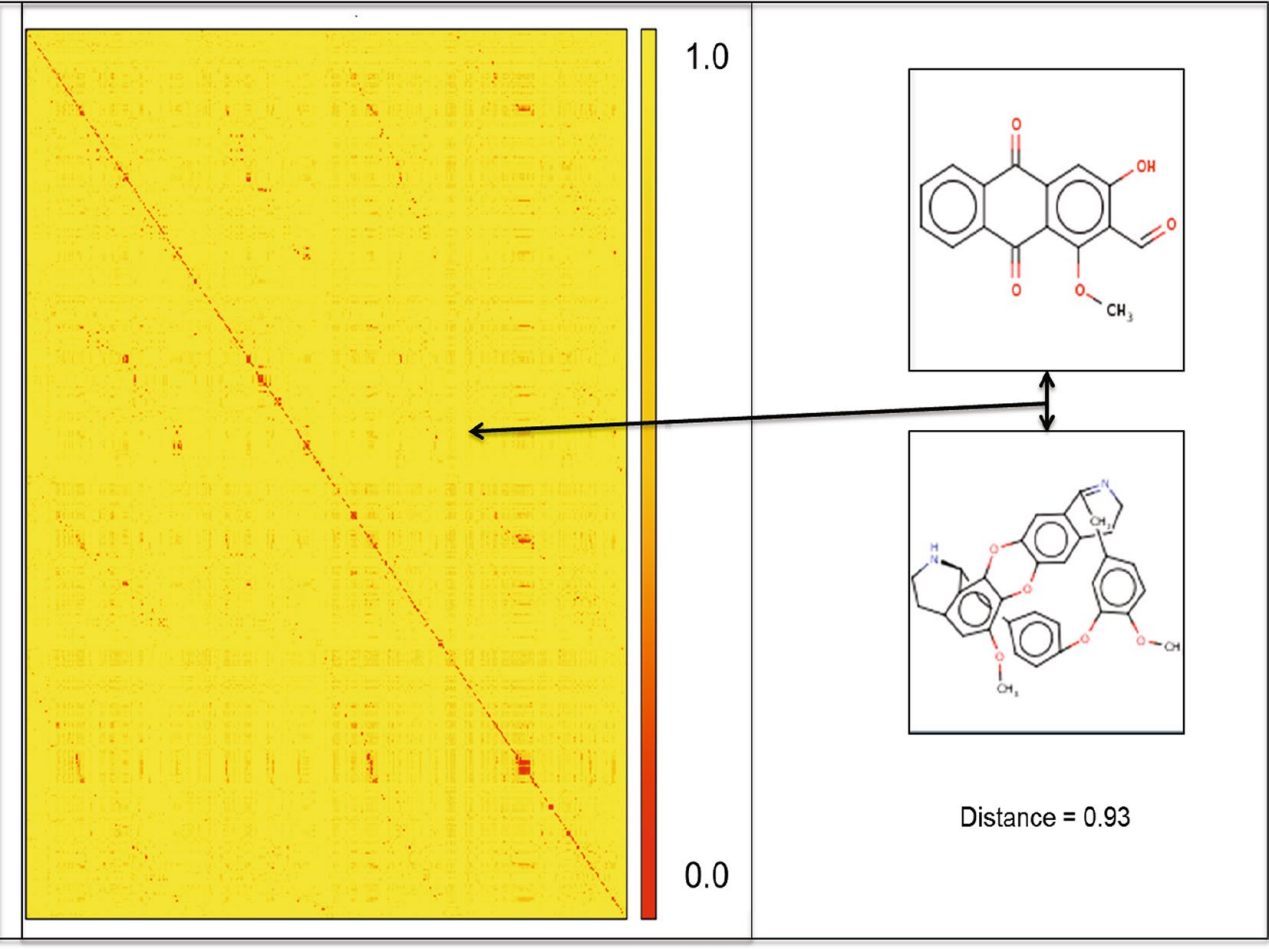
Molecular similarity among the compound sets. The heat map was generated based on most common substructure (MoSSMCS) in KNIME. It provided a visualization of the molecular similarity amongst the NAA and CRAD. Predominate *yellow colour* (high distance) on the heat map signifies that most of the compounds are structurally far apart or highly diverse. The distance between two of the compounds at a *data point* on the heat map is highlighted

The result of the molecular similarity assessment based on most common substructure, using the MoSSMCSS algorithm [23] is presented as a heat map (Fig. 5). The heat map showed a larger area of low similarity (lower values of Tanimoto coefficient) amongst the compounds. This is indicative of the substructural diversity amongst the NAA. A closer look at the heat map, using similarity viewer in KNIME, revealed Tanimoto coefficient in the range 0.1–0.7 between NAA and CRAD. This suggests that most of the NAA are structurally diverse from CRAD.

**Fig. 6:**
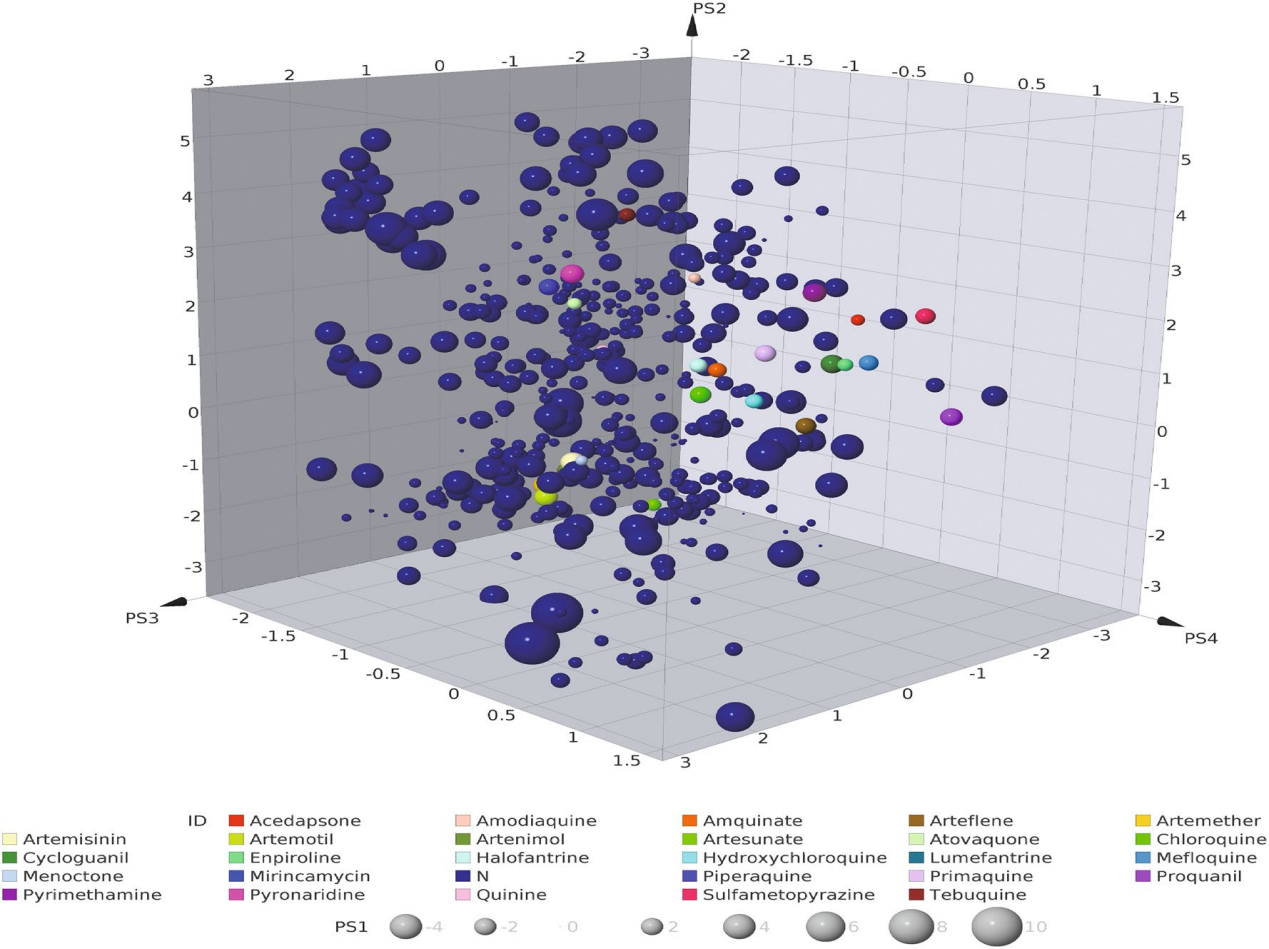
Chemical space coordinates of compound sets. Coordinates were generated for NAA and CRAD from ChemGPS-NP. Dimensions: PS1-size (represented by size of the markers), PS2-aromaticity, PS3-lipophilicity and PS4-flexibility. Most NAA (*dark blue markers*) occupy distinct positions in the chemical space in comparison to CRAD

A similar observation was made when ChemGPS-NP [24] was used to compare the spatial coordinates and volume of chemical space occupied by NAA relative to CRAD. The result (Fig. 6) shows a plot of the first four dimensions: aromaticity, lipophilicity and flexibility, representing PC2, PC3 and PC4, respectively, plotted on the x, y and z axes. PC1, which represented size, was indicated by the size of the markers on the plot. The compounds in CRAD were identified by coloured markers. A cursory look at Fig. 6 shows that the CRAD did not form a tight cluster but were dispersed within the chemical space. A wider dispersion was however observed for NAA particularly in the positive direction of PC1 (size) A closer look revealed that NAA were bigger in size (PC1) and more aromatic (PC2) than the CRAD. Additionally, the dispersal of NAA and CRAD appears to be similar along the PC4 (flexibility) but slightly different along the PC3 (lipophilicity) with the NAA tending towards less lipophilicity. The proximity of some of the NAA to some of the CRAD may be a sign of their desirable drug-like properties and their amenability as a starting point in anti-malarial drug development. Although other studies have revealed that natural products occupy, in comparison to drug-like compounds, unique regions of property space [32, 89, 90], this result showed that some of the NAA occupy similar chemical spaces as CRAD, which also consist of natural product based anti-malarial drugs.

Based on the ‘similarity property principle’ [91], NAA that are diverse from CRAD may have different bioactive profiles or mechanism of action. Structurally diverse NAA may have different spatial orientation within biological targets in *Plasmodium* with resultant unique molecular interactions [92]. This is particularly relevant in light of recent resistance of *Plasmodium* to CRAD. Compounds that may have different interactions pattern with targets implicated in *Plasmodium* resistance to CRAD or that interact with new targets are highly desirable for anti-malarial drug development. Moreover, such structurally diverse active antiplasmodial compounds provide insight into new chemical groups required for anti-malarial activities.

Conversely, NAA that are structurally similar to CRAD may have similar pharmacokinetic properties and may be drug-like. Since pharmacokinetic properties have been implicated as a major determinant of compound success or attrition during drug development [93, 94], NAA that are structurally similar to CRAD may suffer less attrition going through anti-malarial drug development. In addition, the chemical space analysis (Fig. 6), using the CRAD as reference or signpost, allows the visualization of relative position of NAA in chemical space compared to CRAD. Such view may enable the medicinal chemist to identify NAA that are within or outside the desired region (i.e., space occupied by CRAD). It may also assist the medicinal chemist to recognize the necessary properties to optimize and the extent of optimization required to move the NAA towards the desirable drug-like region or ‘sweet spot’ [83].

Overall, molecular diversity from CRAD may be indicative of new mechanism of action and potential for circumvention of current drug resistance while molecular similarity to CRAD may be indicative of favourable drug-like profile.

### Structure–activity landscape: identifying activity cliffs

Exploration of structure–activity landscape represents a core aspect of medicinal chemistry [95]. Activity cliff has been defined as pair of structurally similar compounds with large difference in bioactivity/potency [96] and has been of interest to the medicinal and computational chemist for a long time [97, 98]. To identify a pair of NAA that display activity cliffs, structure–activity similarity analysis was conducted with DataWarrior [21].

**Fig. 7:**
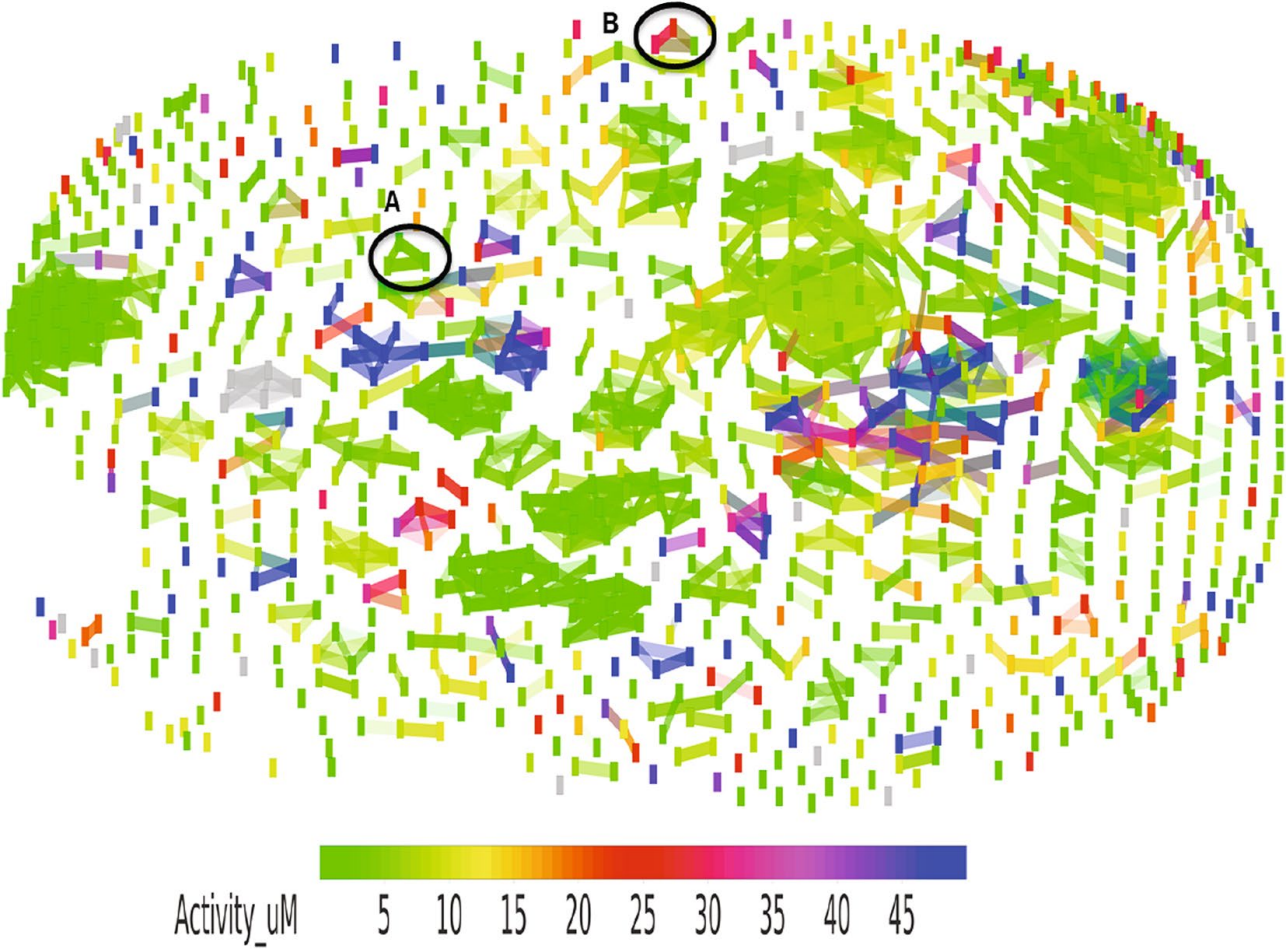
Self-organizing map depicting structural-activity landscape of the natural products with in vitro antiplasmodial activities. The SOM was generated based on a measure of structural and activity similarities among the NAA. Markers connected by *lines* represent similar compounds. *Markers* are coloured by activities (IC50). *Connected markers* that have *different colours* (e.g., cluster marked* B*) represent structurally similar compounds with different in vitro antiplasmodial activities (activity cliff). The cluster marked* A*, which consists of structurally similar compounds with similar in vitro antiplasmodial activities represent smooth region

The result is presented as a SOM (Fig. 7) that display the relative position, in a 2D space, of all the NAA. Similar compounds are connected with a line and the markers are coloured by antiplasmodial activity (IC_50_) of the compounds from green (active NAA (≤5 uM)) to dark blue (inactive NAA (≥45 uM)). Clusters of green markers connected with lines (one of the cluster is marked ‘A’) representing similar NAA with similar antiplasmodial activity were observed within the landscape. This group of NAA form the ‘smooth region’ of the structure–activity landscape where minor changes in molecular structure usually lead to small change in bioactivity. This collection of NAA may be particularly appealing because it will allow the medicinal chemist to rationalize chemical substitutions that will improve pharmacokinetic parameters without sacrificing potency or bioactivity. This group of compounds are also amenable to quantitative structural activity relationship (QSAR) modelling because their structure–activity property aligns with the assumption of statistical modelling [95].

**Fig. 8:**
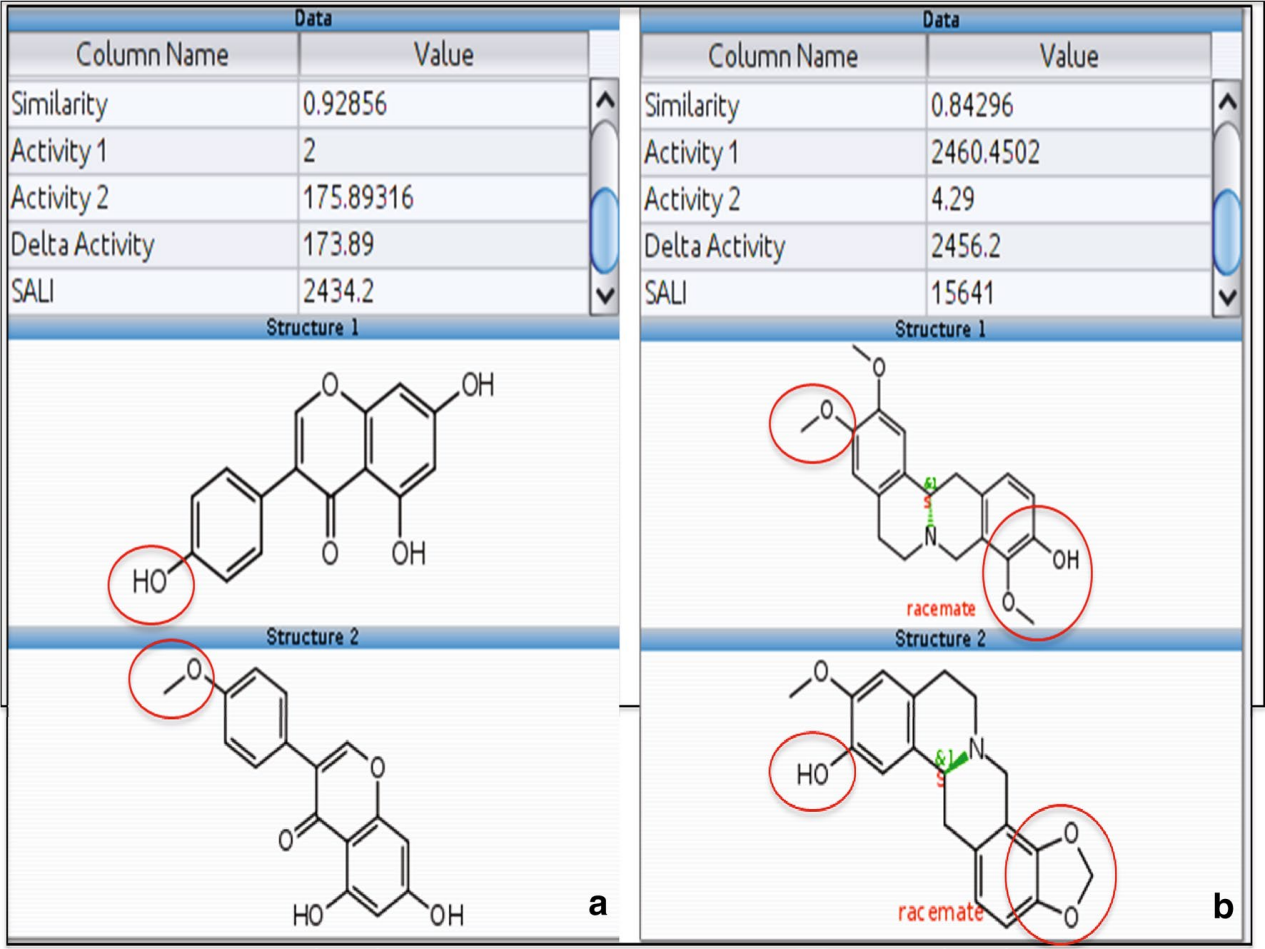
Pairs of natural products with in vitro antiplasmodial activities that represent activity cliffs. Panels **a** and **b** show the structures, the value of structural similarity (similarity), activity (IC_50_) of each compound (Activity 1 and Activity 2), difference in activity (Delta activity) and structure–activity landscape index (SALI). The major structural differences between the compounds are highlighted with red circles. On Panel **a**, the hydroxyl group and methoxy group highlighted on Structure 1 and Structure 2 seem to make the difference between the antiplasmodial activities. This structural-activity landscape analysis provides valuable information about structural features in NAA required for antiplasmodial activity

A closer look at Fig. 7 also reveals few clusters, one is marked ‘B’, which contains green markers (active NAA) connected to red and blue markers (inactive NAA). These clusters of NAA are structurally similar but have diverse bioactivity representing typical activity cliffs (see Additional file 4). Two examples of pairs of NAA that displayed activity cliff are shown in Fig. 8.

A plot of molecular similarity against activity similarity between pairs of compounds with markers coloured by fold change in normalized activity value [Δp(IC_50_)], provides another visualization for identification of the pairs of NAA that exhibit activity cliff (see Additional file 5). Markers that fall within activity similarity <0.8 (low activity similarity) and molecular similarity >0.8 (high molecular similarity) as well as high Δp(IC_50_) value signify pairs of NAA that show an activity cliff. Conversely, markers with activity similarity >0.8 (high activity similarity) and molecular similarity >0.8 (high molecular similarity) as well as low Δp(IC_50_) value signify pairs of NAA that are in the smooth region of the structure–activity landscape. The smooth region consists of compounds that adhere to the similarity principle and are pliable and desirable for QSAR models, as mentioned earlier.

Although Maggiora [98] proposed that activity cliffs may be responsible for the inefficient performance of many quantitative QSAR models [95], activity cliffs help pinpoint regions of the activity landscape that contain maximum information for structure activity relationship [SAR] studies. This is because this allows the medicinal chemist to identify the subtle molecular difference between a pair of compounds responsible for a dramatic shift in bioactivity. The rich SAR information from activity cliffs have been used in many drug discovery studies [99–101].

### Pharmacokinetic profiling and drug-likeness of NAA

The pharmacokinetic profile and drug-likeness of NAA were assessed in silico using the models discussed below:

### Absorption models

#### Lipinski’s rule of five

Lipinski’s rule of five got its name from the cut-off values for each of the four parameters that define the potential of a drug candidate for good absorption: the molecule has less than five HBD and less than ten HBA, its MW is below 500, and its LogP is less than five [26].

These four parameters were calculated for NAA as well as CRAD and plotted on a 3D graph with markers sized by MW (see Additional file 6). It was observed that most of the CRAD occupied space that was within the Lipinski’s rule of five, while most of the NAA (HA, A, MA) were dispersed away from Lipinski’s rule of five space. This suggests that the NAA (HA (47 %), A (52 %), MA (48 %), and N (71 %)) within Lipinski’s rule of five space may likely have good passive absorption. It was noted that a greater proportion of the N NAA showed propensity for good absorption. This suggests that though violation of Lipinski’s rule of five confers absorption liabilities it may be associated with good bioactivity among the NAA. Although limitations of Lipinski’s rule of five to predict the absorption of natural products and molecules that are actively transported have been reported [102–105], it is desirable that NAA that fall within Lipinski’s rule of five space be prioritized to lower attrition rates during anti-malarial drug development and increase the chance of new anti-malarial drugs reaching the market [26, 81].

Although formulation and drug delivery strategies have been developed to improve the absorption of compounds that violate Lipinski’s rule of five (i.e., poorly absorbed drugs) [106–110], it is important to be aware of the cost of such technology and its impact on the eventual market price of an anti-malarial drug. This is in view of the economically disadvantaged population in malaria-endemic regions of the world that require these anti-malarial drugs.

#### Veber et al. model

Veber et al. [29] suggested NRB and PSA of a compound as determinants of oral absorption. A plot of NRB and PSA was generated for NAA and CRAD (see Additional file 7, panel A). Veber et al. found that the majority of compounds with good oral bioavailability in rats had fewer than 10 rotatable bonds and PSA less than 140 Å^2^.

All the CRAD, except one with 11 NRB (halofantrine), were within the desirable area (red rectangle). A large number of the NAA [A (81 %), HA (76 %)and MA (80 %)] were also present within this region suggesting that these compounds may be well absorbed orally. The NAA that were dispersed outside the desired area and along the NRB axis have long aliphatic chains in their structure, while those dispersed along the PSA axis contain high number of hydroxyl and carbonyl groups. Lead optimization strategies may change single aliphatic bonds to double bonds to reduce rotation and polar groups may be methylated to reduce PSA to improve oral absorption of such compounds. However, the effect of such modification on the bioactivity of the compounds needs to be monitored.

#### Egan Egg plot [passive gut absorption]

Similar to Lipinski’s rule of five, Egan et al. [27] used statistical analysis to correlate passive intestinal absorption with PSA and clogP. A plot of PSA against clogP for NAA and CRAD is shown in Additional file 7, panel B. The ellipsoidal area of the plot (aka Egan Egg) enclose compounds that are expected to have good passive gut absorption. Compounds that fall outside the outer Egan Egg are predicted to have poor passive gut absorption, but may be absorbed by active transport processes. The results showed that most of the NAA, like the CRAD, fall within and at the border of the Egan Egg, suggesting that they may be well passively absorbed. A closer look revealed that 50 % of compounds in HA may have good oral absorption, 15 % may have borderline oral absorption while 45 % may show poor oral absorption. Slightly similar distribution in proportion was observed for compounds in A and MA. This model not only identifies NAA, especially HA and A, that may have poor absorption but the implicated physicochemical properties (PSA or clogP) may be identified and noted as one of the parameters to be addressed during lead optimization.

#### Golden triangle model

An analysis of Caco-2 permeability data for more than 16,000 compounds and human liver microsome clearance data for about 47,000 on a plot of distribution constant (logD) *versus* MW showed that compounds with good permeability and low clearance are concentrated within a triangular-shaped area (golden triangle) [28]. A similar plot, logD (at pH 7.4) *versus* MW, was generated for the compounds in the dataset (see Additional file 7, panel C). The results showed a small proportion of the NAA (as well as CRAD) within the golden triangle (defined by: base of triangle is logD between −2 and 5 and apex is at MW 500). Using DataWarrior to select compounds within the triangle, it was observed that the following proportions of NAA were predicted to have tendency for good permeability and low clearance: A (35 %), HA (25 %) and MA (33 %). Approximately 33 % of the compounds in CRAD were predicted by this model to possess propensity for good permeability and low clearance. The bioavailability and clearance [half-life] data provided in the drug bank [111] for the CRAD predicted to possess good permeability and low clearance were explored to ascertain the consistency of the prediction. The data provided by drug bank for the identified compounds in CRAD align with some of the predictions by this model.

#### Fragment-based drug-likeness

**Fig. 9:**
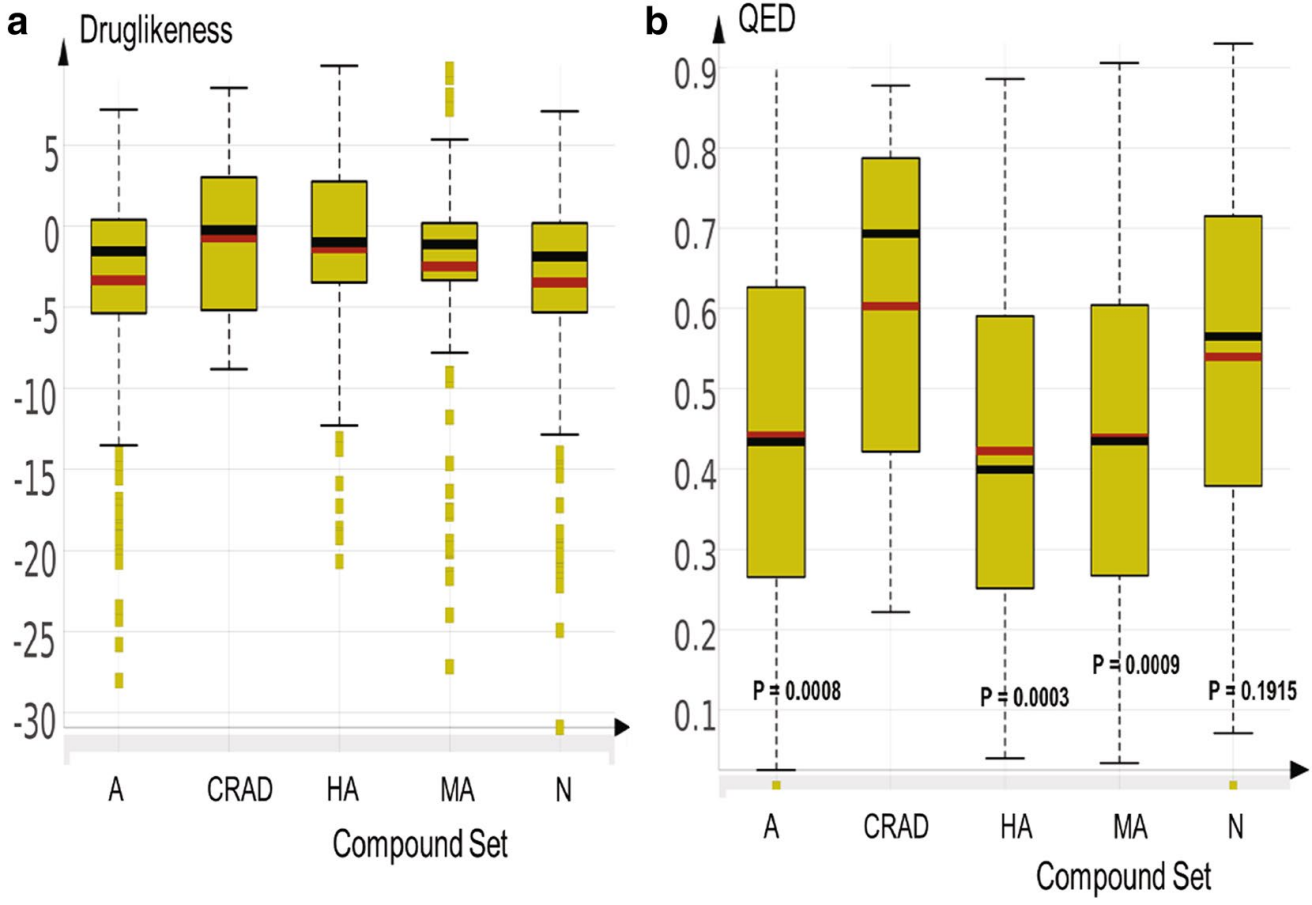
Boxplots showing the distribution and summary statistics of drug-likeness. Panel** a** is the fragment-based drug-likeness index from Datawarrior and panel** b** is the QED for sub-groups of NAA (A, HA, MA, N) and CRAD. The *red* and *black lines* represent the mean and median, respectively, for each distribution. No significant difference in fragment-based drug-likeness index was observed between CRAD and sub-groups of NAA in panel** a**. The sub-groups of NAA: HA, A and MA showed mean QED values that were significantly lower than that of CRAD (see p values inserted in graph)

Fragment-based drug-likeness of NAA and CRAD, calculated with DataWarrior [21], is presented as boxplots (Fig. 9, panel a). A higher proportion of the compounds in CRAD and HA, and a lower proportion of A, MA and N were in the positive region of the drug-likeness score (drug-likeness score >0). Comparison of the mean drug-likeness score of NAA to CRAD showed that the HA (mean = 1.389) was slightly lower while A, MA and N were significantly lower than CRAD.

This approach to assessment of drug-likeness is based on presence of substructure fragments which are frequently present in commercial drugs. A positive value shows that the query molecule contains predominantly fragments that are frequently present in commercial drugs. HA, among the NAA, contains a higher proportion of compounds that may be drug-like and may have greater chance of success during anti-malarial drug development.

#### Quantitative estimate of drug-likeness

Bickerton et al. [112] recommended a new metric, the QED, to estimate drug-likeness of hits, leads or drug candidates. The QED value (‘desirability functions’) is mapped onto a scale between 0 and 1, where a desirability of 1 signifies an ideal value of the drug-like property and a desirability of 0 relates to a completely intolerable outcome.

The QED calculated for CRAD and NAA (sub-groups), using ICM Chemist Pro (Molsoft), is summarized as a boxplot (Fig. 9, panel b). As expected, CRAD showed a distribution that tends towards 1 (ideal value of the drug-like property) with an average QED value of 0.602. The average QED value for HA, A and MA were significantly lower than that of CRAD. On the other hand, N showed a similar drug-like score to CRAD. This is a contrast to what was observed using the fragment-based drug-likeness score which shows that HA had similar drug-likeness score to CRAD.

This observation may be due to the different approach used to estimate the QED value. While the fragment-based drug-likeness is based on presence of substructure fragments in commercial drugs, QED is generated from eight properties commonly used to define drug-likeness: MW, logP, HBA, HBD, PSA, number of aromatic groups [AROM], NRB and ALERTS [the number of matches to undesirable functionalities] [112]. The distributions of these eight properties for a set of oral drugs were conducted and a desirability of 1 was assigned to the property values of oral drugs that occur most commonly, and 0 to property values that are not observed.

The low QED value estimated for HA and A thus signify that these compounds have low similarity to bulk of oral drugs and may have reduced chance of success during drug development. On the other hand, the low similarity of HA and A to CRAD and bulk of oral drugs may also be an indication of their structural peculiarity or novelty (as observed during molecular similarity analysis). These compounds may be the starting point of new anti-malarial drugs with unique mechanism of action.

#### Toxicity potential assessment

**Fig. 10:**
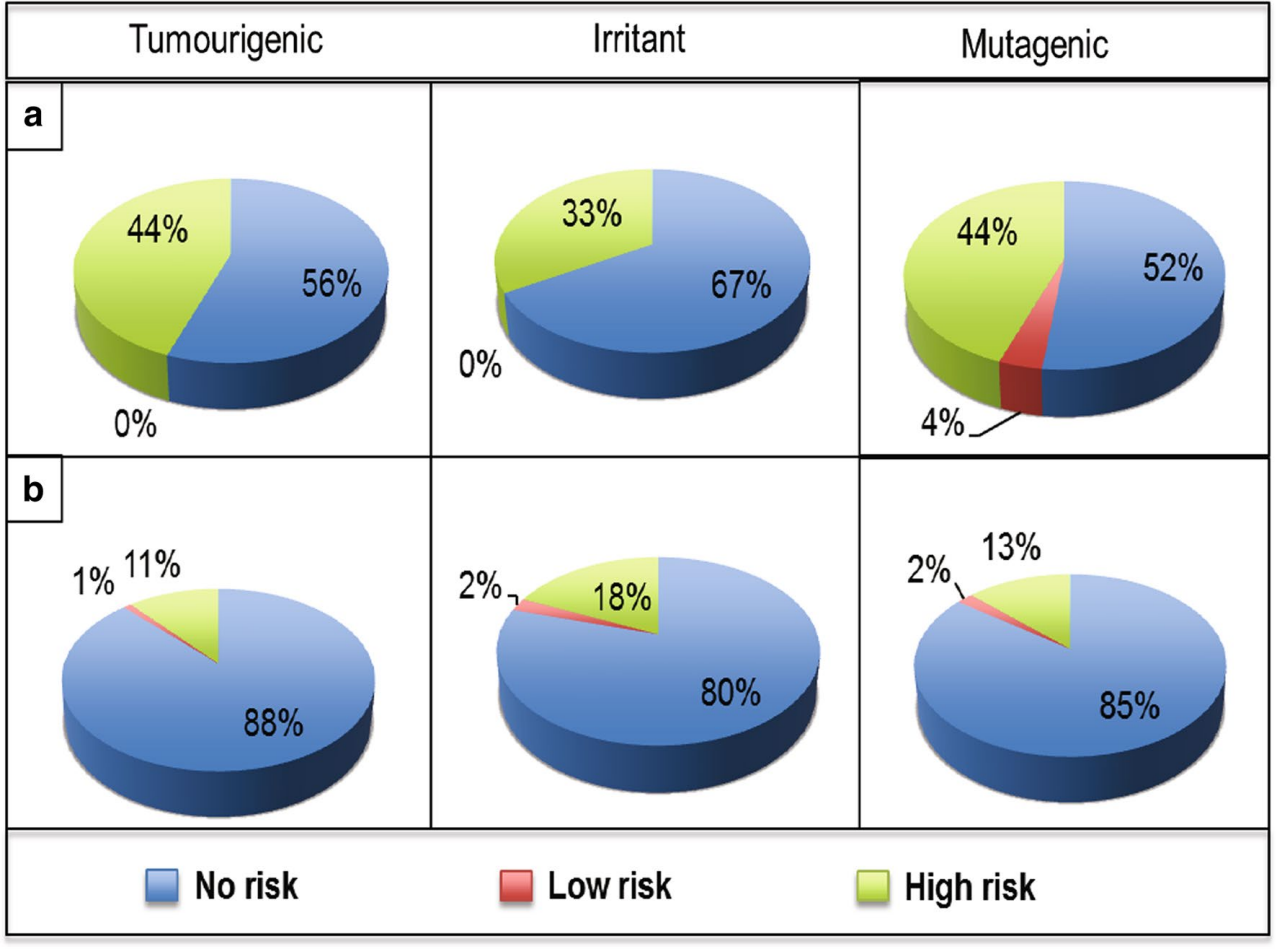
Toxicity profile of currently registered anti-malarial drugs and natural products with in vitro antiplasmodial activities. The upper panel** a** is for the CRAD while the lower panel** b** is for NAA. Three indicators were used to assess the toxicity: tumourigenic, irritant and mutagenic. A greater proportion of NAA were predicted to have low risk of the toxicity indicators assessed

The potential for toxicity from the NAA was assessed by checking for the presence of reactive chemical groups and potential to cause tumour, irritation and mutagenesis. Assessment of tumourigenic, irritant and mutagenic risk seeks to identify compounds with possibility to cause tumour, irritation and mutation in vivo. Figure 10, a visualization of the results of the toxicity assessment, showed that a greater proportion of NAA (>80 %) showed no risk for tumourigenic, irritant and mutagenic potential in comparison to CRAD (<60 %). This may attest to the low toxicity of these NAA, as previously observed for other natural products [113], and their potential as a source of new and safe anti-malarial drug candidates. However, it is worth noting that drug approvals are based on rigorous benefit-risk assessment [114, 115] and NAA, with high risk of the assessed toxicity parameters, may be considered for anti-malarial drug development provided the benefits from such compounds outweigh the potential risk.

In the context of drug development, reactive groups are usually electrophiles or free radicals that may react readily [via covalent binding] with nucleophilic components, such as DNA and proteins within the biological system [116, 117]. Reactive groups were observed in 33.3 % of CRAD and that was significantly lower than observed for the NAA (47.49 % in HA, 47.37 % in A, 55.4 % in MA, and 44.58 % in N). Reactive groups identified include peroxo, oxiran/aziridine, allyl/benzyl chloride, 2-halo-enone, 3-halo-enone, and quaternary ammonium. Reactive groups present within some drugs or formed after metabolism of such drugs within the biological system, have been implicated for unexpected toxicities of drugs that become apparent only after the launch of such drug entities [117–119].

Overall, these results showed that NAA have lower potential for toxicity in comparison to CRAD. Although the presence of reactive groups, tumourigenic, irritant and mutagenic risk is indicative of toxicity risk, these toxicity risk alerts are by no means to be taken as fully reliable predictors of toxicity. Nor should the absence of these toxicity risk alerts be a confirmatory indication that a compound will be completely free of toxicity. Nonetheless, in silico toxicity assessment of NAA early during hit profiling allows the de-prioritization of compounds that may have unexpected toxicity issues. In addition, implicated chemical groups may be replaced with other groups while retaining the biological activity of the compound through scaffold hopping. This may help to design and bring safer anti-malarial drugs to the market.

#### Assessing promiscuity of NAA: Eli Lilly MedChem rules

Promiscuous compounds or frequent hitters in NAA may be false positives from antiplasmodial assays [120]. Identifying and flagging such compounds will guide selection of NAA hits, preferably excluding such frequent hitters, for the next stage of anti-malarial drug development.

Applying the Eli Lilly MedChem rules on the NAA and CRAD identified promiscuous or reactive compounds in both datasets. Approximately 63 % of the compounds in CRAD failed the rules with the predominate reasons for failure being the presence of ‘peroxide’ and ‘para quinone’ groups [30]. The former captures direct oxidants, which may be the artemisinin derivatives present in the CRAD, while the latter identify para-positioned quinones that have high redox potential. Although these compounds in CRAD are registered drugs, they represent chemotypes that would have been triaged out of screening sets due to their high reactivity.

Promiscuity indicates a compound with non-specific mode of action and may lead to increased incidence of toxicity [121]. In the case of NAA, 82 % of the compounds in HA failed the rules, 79 % compounds failed in A, 78 % in MA, while 64 % failed in N. The predominant reason for failure in all the sub-groups of the NAA was ‘presence of catechol’ group which implies the presence of dihydroxybenzene groups [30]. Such groups, which have high redox capacity and are highly promiscuous, are predominant in natural products from plants, e.g., flavonoids, anthraquinones, terpenoids compounds, etc. However, there was no significant (p > 0.05) difference in the proportion of compounds with reactive groups between CRAD and sub-groups of NAA (except MA) (see Additional file 3). No correlation (r = −0.031) was observed between the number of reactive compounds and the bioactivity (IC_50_) of the compounds (see Additional file 3). These observations either underscore the aggressiveness of the Eli Lilly MedChem rules or attest to the presence of natural products and natural product-derived compounds in CRAD. One may argue that the rule should be used with caution but it is worthwhile as it will flag compounds with bioactivity to be looked at more closely. This will guard against the use of false positives or non-specific NAA as potential starting points for anti-malarial drug development.

#### Prioritization of NAA: integration of chemo-informatic profiling data

Using the information provided in this study, especially within an interactive data-mining environment such as DataWarrior software [21], it is possible to provide an answer to the key question posed: Should NAA that are most likely to be successfully developed into anti-malarial drug candidates be prioritized? One way to provide an affirmative response to this question was to adjust the filters of the molecular descriptors or/and physicochemical properties within DataWarrior to the reported exemplar values for all the calculated molecular descriptors and physicochemical properties. The result of this operation will be a prioritized list of NAA that possess the desired molecular descriptors or/and physicochemical properties. About 28 % of the NAA fell within these exemplar values or limits. However, the proportion of NAA prioritized may vary based on the selected molecular descriptors and physicochemical properties and the choice of exemplar values. In addition, the use of filters will permanently remove seemingly interesting compounds that do not fall within the range set on the filters.

Another approach used to get a prioritized list of NAA was to generate a consensus scoring function for each compound in NAA. The cut-off or exemplar values for each calculated molecular descriptor and physicochemical property were used as the benchmark to score each compound (molecular descriptor and physicochemical property that were not significantly different between the two datasets, i.e., CRAD and NAA, were excluded). Compounds with the values of molecular descriptors and physicochemical properties outside the desirable range were penalized with a score of −1 while compounds within the desirable range were rewarded with a score of 1. The average of these scores for all the molecular descriptors and physicochemical properties discussed in this study were taken as the consensus score. A consensus score of 1 suggests that such compounds have all molecular descriptors and physicochemical properties within the acceptable range and may be prioritized for the next stage of pre-clinical anti-malarial drug development. The prioritized list of the NAA with their consensus score [list was sorted by the consensus score, highest to lowest] is shown in Additional file 8. As expected, the consensus scoring showed high values (0.5–1.0) for over 90 % of the CRAD, which have successfully passed through- anti-malarial drug development. Prospective NAA that showed high score (e.g., above 0.5) share similar drug-like properties with CRAD and may stand a greater chance of successfully passing to development to become anti-malarial drugs.

**Fig. 11:**
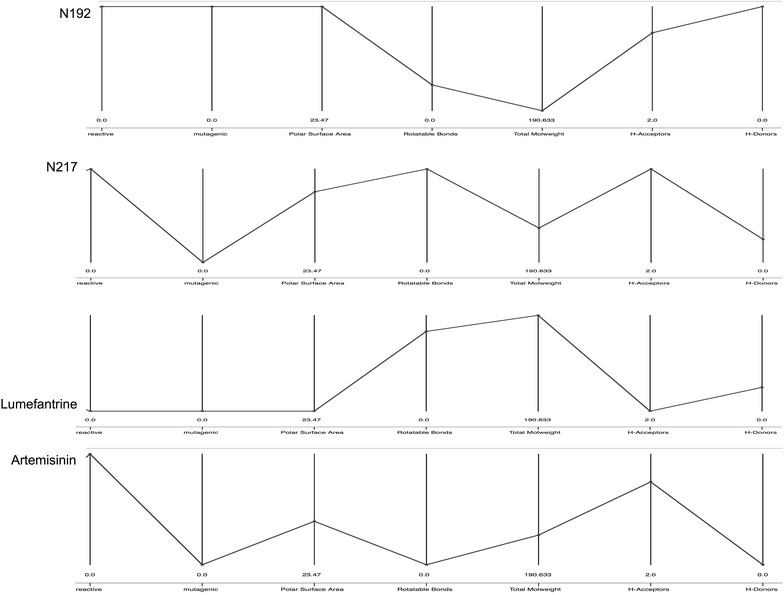
Parallel coordinate graph showing the chemo-informatic profiles of selected compounds. The graph was created with KNIME. On the y-axis are the compounds and on the x-axis selected molecular descriptors. N197 and N217 are representatives of NAA. Lumefantrine and artemisinin represent CRAD. The graph allows visualization of the profile of NAA in comparison to CRAD based on selected molecular descriptors

Overall, compounds within NAA with consensus score close to 1 (i.e., those that fall on the positive side of the cut-off values of the various chemo-informatic properties assessed) may have greater chance of success during anti-malarial pre-clinical drug development. Alternatively, visualization of all metrics in the context of CRAD can aid prioritization and selection of NAA for downstream anti-malarial drug discovery (Fig. 11). The graph allows visualization of the profile of NAA in comparison to CRAD based on selected molecular descriptors. It is expected that NAA that have similar profile to CRAD (i.e., have similar values of selected molecular descriptors) should be more drug-like and therefore be prioritized for the next stage of anti-malarial drug development.

## Conclusions

Chemo-informatic profiling of NAA and CRAD has led to development of prioritization strategies and prioritized lists of at least 1000 compounds that may guide decisions and facilitate anti-malarial drug development from natural products with antiplasmodial activities. This prioritized list includes structurally diverse NAA that may encompass new biologically relevant chemical space and could be developed into anti-malarial drug candidates with possible different mechanism of action. Structure–activity landscape analysis revealed NAA pairs that form activity cliffs, which are particular relevant for SAR studies. Finally, this study was able to identify NAA with desired drug-like properties and toxicity liabilities as well as promiscuous compounds or ‘frequent hitters’ among the NAA.

## Additional files

10.1186/s12936-016-1087-yNatural products with in vitro antiplasmodial activities.
10.1186/s12936-016-1087-y Selected natural products with in vitro antiplasmodial activities and currently registered anti-malarial drugs.
10.1186/s12936-016-1087-y Summary statistics of selected molecular descriptors and physicochemical properties.
10.1186/s12936-016-1087-y Activity cliffs within the natural products with in vitro antiplasmodial activities. Columns show the structures of pairs of compounds that form activity cliffs, identities of compounds (ID1 and ID2), structural similarity between the compounds (Tanimoto coefficient), bioactivity (IC_50_) of each compound (Activity 1 and Activity 2), difference in bioactivities (Delta activity) and structural-activity landscape index (SALI). The SALI value reflects how much activity is gained with a small modification of the chemical structure.
10.1186/s12936-016-1087-y Scatter plot of activity similarity and molecular similarity of natural products with in vitro antiplasmodial activities. Markers are coloured by change in activity (Delta pActivity). Activity cliff region is bounded by activity similarity below 0.8 and molecular similarity above 0.8.
10.1186/s12936-016-1087-y Four-dimensional plot of Lipinski’s rule of five for compound sets. NAA relative to CRAD.
10.1186/s12936-016-1087-y Pharmacokinetic models depicting the proportion of compounds that fall within desired regions of good bioavailability. The models shown include: model by Veber et al. (X), Egan Egg model (Y) and golden triangle model (Z). The models were applied on the sub-groups of NAA (A, HA and MA) and CRAD. Marked regions encompass compounds that fall within desired regions and that may possess good bioavailability.
10.1186/s12936-016-1087-y Prioritized list of natural products with in vitro antiplasmodial activities. Columns include the structure of the compounds, the identity of the compounds (ID), activity_status (A, HA,MA or CRAD) and the consensus score. List was sorted by the activity status.

## Abbreviations

NAA: natural products with in vitro antiplasmodial activities
HA: highly active natural products with in vitro antiplasmodial activities
A: active natural products with in vitro antiplasmodial activities
MA: moderately active natural products with in vitro antiplasmodial activities
N: low active natural products with in vitro antiplasmodial activities
CRAD: currently registered anti-malarial drugs
HBD: hydrogen bond donor
HBA: hydrogen bond acceptor
TMW: total molecular weight
NRB: number of rotatable bonds
TPSA: total polar surface area
vsa_hyd: Van der Waals hydrophobic surface area of hydrophobic atoms
rsynth: synthetic feasibility
LE: ligand efficiency
LLE: ligand lipophilicity efficiency
LELP: ligand efficiency dependent lipophilicity
SOM: self-organizing map
KNIME: Konstanz information miner
QSAR: quantitative structural activity relationship
SAR: structural activity relationship
QED: quantitative estimate of drug-likeness
GIT: gastro instestinal tract
MoSSMCSS: MoSS most common substructure
SALI: structure activity landscape index
