# Supplementary material for: Prioritization of anti-malarial hits from nature: chemo-informatic profiling of natural products with in vitro antiplasmodial activities and currently registered anti-malarial drugs
Source: Malar J. 2016 Jan 29;15:50. doi: 10.1186/s12936-016-1087-y (PMC4731946; doi:10.1186/s12936-016-1087-y)
Supplement: Supplementary file 4 — 10.1186/s12936-016-1087-y Activity cliffs within the natural products with in vitro antiplasmodial activities. Columns show the structures of pairs of compounds that form activity cliffs, identities of compounds (ID1 and ID2), structural similarity between the compounds (Tanimoto coefficient), bioactivity (IC50) of each compound (Activity 1 and Activity 2), difference in bioactivities (Delta activity) and structural-activity landscape index (SALI). The SALI value reflects how much activity is gained with a small modification of the chemical structure. [file 12936_2016_1087_MOESM4_ESM.pdf]

Structure 1	Structure 2	ID 1	ID 2	Similarity	Activity 1	Activity 2	Delta Activity	SALI
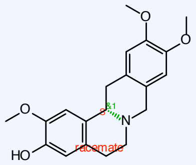	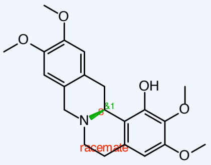	N408	N1228	0.87452	1.6	5223.1006	5221.5	41613
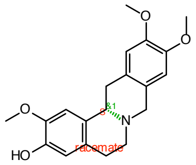	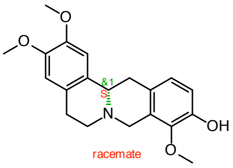	N408	N1225	0.88696	1.6	2460.4502	2458.9	21751
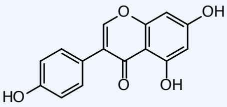	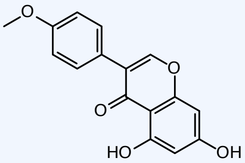	N436	N1210	0.92856	2	175.89316	173.89	2434.2
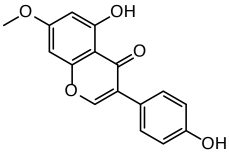	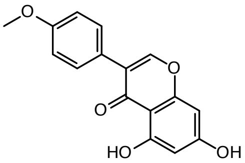	N848	N1210	0.92464	8.5500002	175.89316	167.34	2220.6
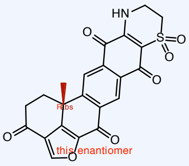	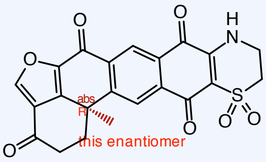	N343	N572	0.99861	1.1	3.9000001	2.8	2019.2
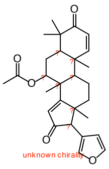	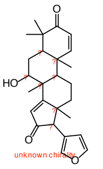	N750	N1202	0.93418	6.4000001	122.39007	115.99	1762.3
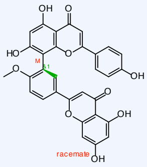	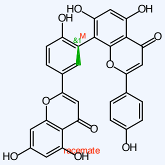	N219	N1148	0.97061	0.30000001	50.700001	50.4	1714.8
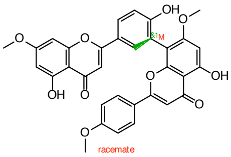	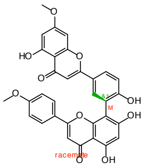	N203	N1138	0.97212	0.25999999	47.130001	46.87	1681.1
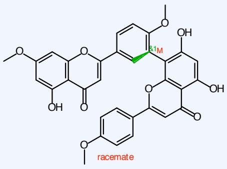	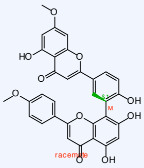	N378	N1138	0.97212	1.4	47.130001	45.73	1640.3
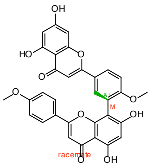	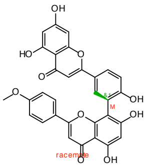	N545	N1142	0.97138	3.5	49.599998	46.1	1610.8
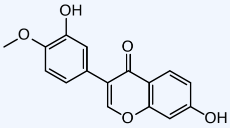	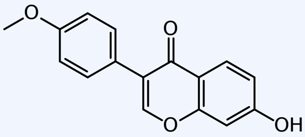	N355	N1212	0.88459	1.2	186.38351	185.18	1604.5

Structure 1	Structure 2	ID 1	ID 2	Similarity	Activity 1	Activity 2	Delta Activity	SALI
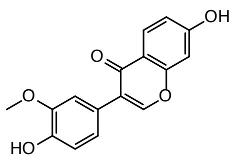	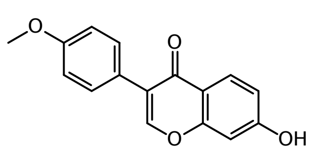	N771	N1212	0.88001	6.8000002	186.38351	179.58	1496.7
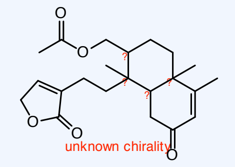	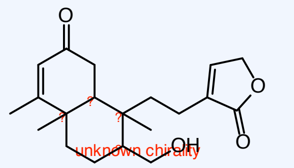	N887	N1198	0.92683	9.8000002	114.9	105.1	1436.3
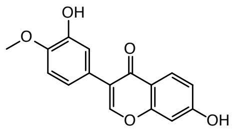	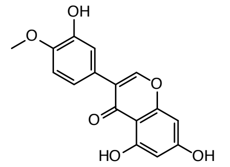	N355	N1208	0.88278	1.2	166.52075	165.32	1410.4
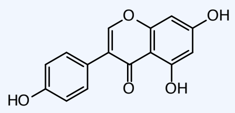	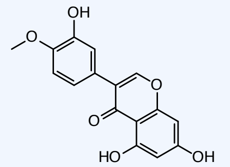	N436	N1208	0.87695	2	166.52075	164.52	1337
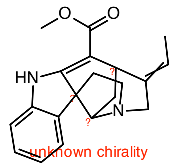	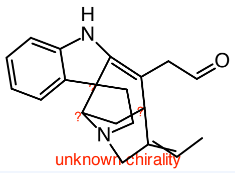	N490	N1203	0.88759	2.7915556	129	126.21	1122.8
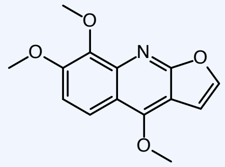	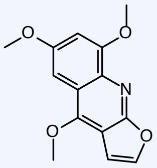	N874	N1207	0.86023	9.3000002	155.82974	146.53	1048.4
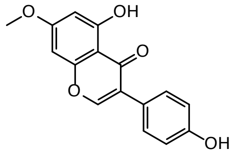	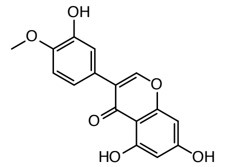	N848	N1208	0.84721	8.5500002	166.52075	157.97	1033.9
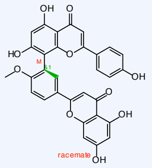	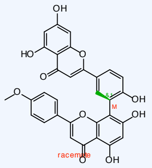	N219	N1142	0.9512	0.30000001	49.599998	49.3	1010.2
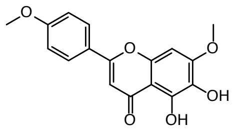	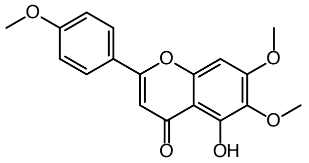	N867	N1184	0.93327	9	74.900002	65.9	987.5
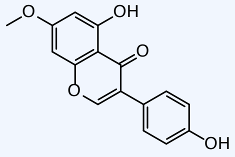	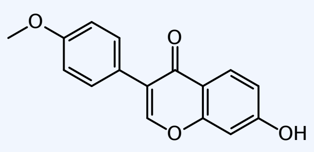	N848	N1212	0.81858	8.5500002	186.38351	177.83	980.2
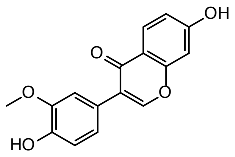	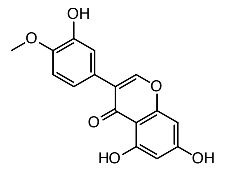	N771	N1208	0.83358	6.8000002	166.52075	159.72	959.73

Structure 1	Structure 2	ID 1	ID 2	Similarity	Activity 1	Activity 2	Delta Activity	SALI
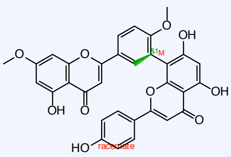	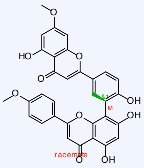	N441	N1138	0.95245	2	47.130001	45.13	949.03
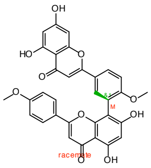	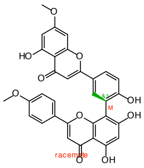	N545	N1138	0.95245	3.5	47.130001	43.63	917.49
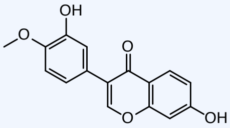	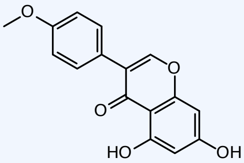	N355	N1210	0.80737	1.2	175.89316	174.69	906.89
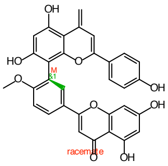	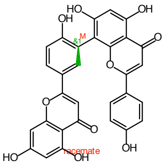	N220	N1148	0.94432	0.30000001	50.700001	50.4	905.19
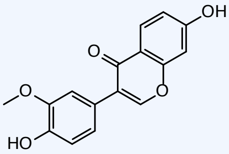	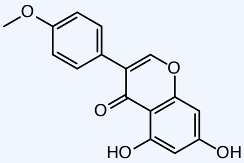	N771	N1210	0.81211	6.8000002	175.89316	169.09	899.98
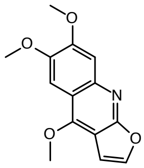	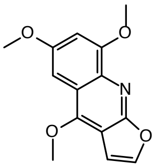	N511	N1207	0.82948	3	155.82974	152.83	896.28
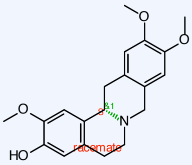	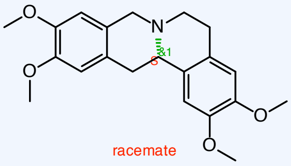	N408	N1151	0.94338	1.6	52.299999	50.7	895.46
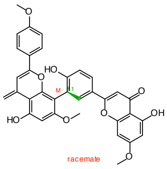	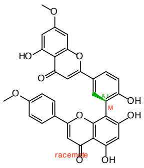	N206	N1138	0.94713	0.25999999	47.130001	46.87	886.45
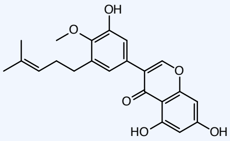	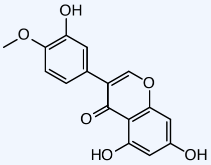	N858	N1208	0.82138	8.6999998	166.52075	157.82	883.55
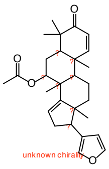	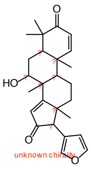	N19	N1202	0.85122	1.21	122.39007	121.18	814.48
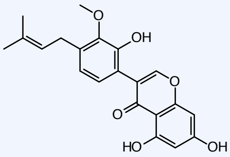	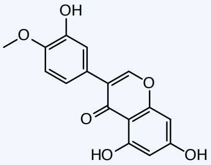	N748	N1208	0.80261	6.3000002	166.52075	160.22	811.71

Structure 1	Structure 2	ID 1	ID 2	Similarity	Activity 1	Activity 2	Delta Activity	SALI
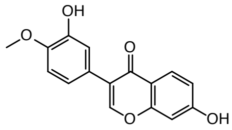	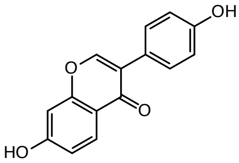	N355	N1195	0.86904	1.2	103.7	102.5	782.7
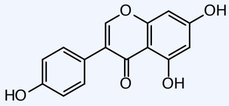	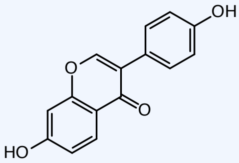	N436	N1195	0.86721	2	103.7	101.7	765.87
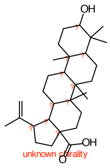	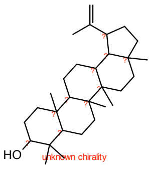	N889	N1161	0.93422	9.8999996	58.586784	48.687	740.2
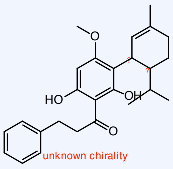	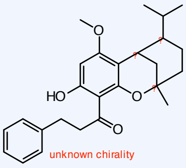	N693	N1193	0.87063	5.5999999	101.27	95.67	739.51
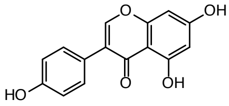	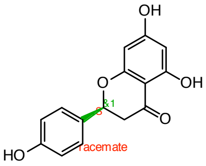	N436	N1206	0.80547	2	141	139	714.54
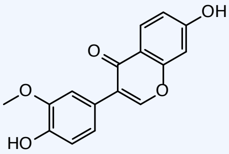	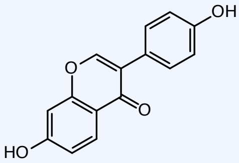	N771	N1195	0.86427	6.8000002	103.7	96.9	713.91
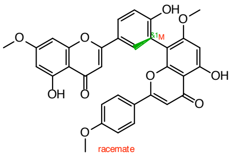	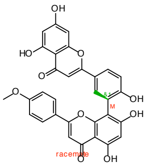	N203	N1142	0.93015	0.2599999	49.599998	49.34	706.41
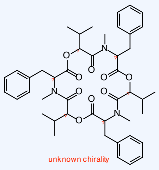	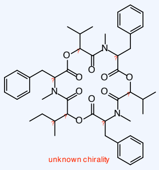	N447	N988	0.98155	2.0409496	15.038058	12.997	704.3
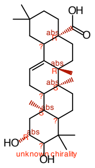	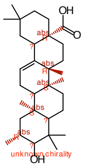	N851	N1045	0.98194	8.6000004	21.1	12.5	692.05
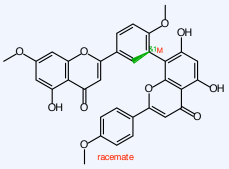	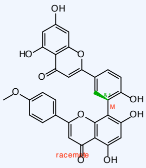	N378	N1142	0.93015	1.4	49.599998	48.2	690.09
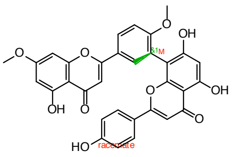	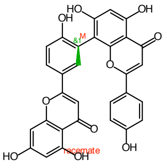	N441	N1148	0.92835	2	50.700001	48.7	679.7

Structure 1	Structure 2	ID 1	ID 2	Similarity	Activity 1	Activity 2	Delta Activity	SALI
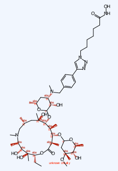	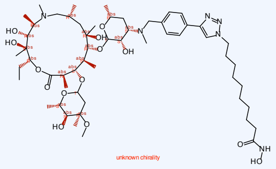	N149	N530	0.99514	0.09499999	3.3	3.205	659.36
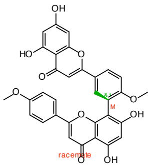	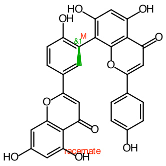	N545	N1148	0.92835	3.5	50.700001	47.2	658.77
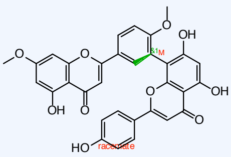	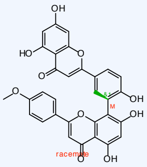	N441	N1142	0.92606	2	49.599998	47.6	643.74
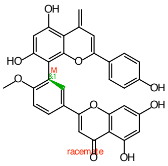	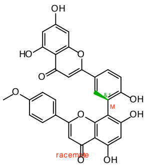	N220	N1142	0.92279	0.30000001	49.599998	49.3	638.5
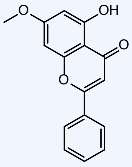	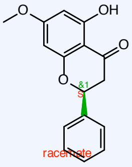	N577	N1201	0.80547	3.98	120	116.02	596.41
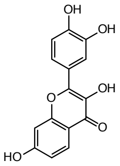	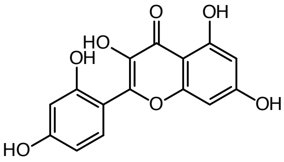	N837	N1197	0.81057	8.1999998	110.4	102.2	539.52
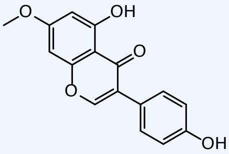	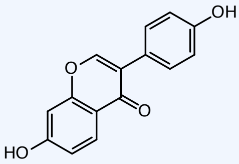	N848	N1195	0.82039	8.5500002	103.7	95.15	529.75
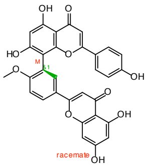	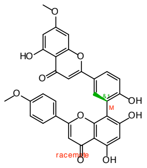	N219	N1138	0.90854	0.30000001	47.130001	46.83	512.04
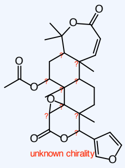	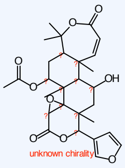	N581	N1118	0.93298	4	37.896145	33.896	505.79
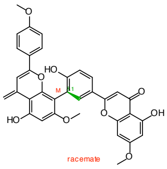	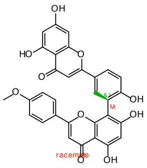	N206	N1142	0.90062	0.25999999	49.599998	49.34	496.46
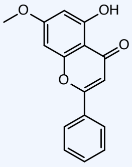	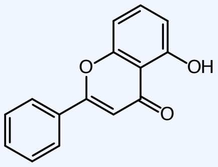	N577	N1171	0.8703	3.98	65.900002	61.92	477.39

Structure 1	Structure 2	ID 1	ID 2	Similarity	Activity 1	Activity 2	Delta Activity	SALI
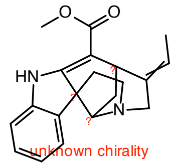	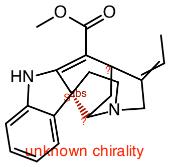	N490	N1126	0.91891	2.7915556	41.299999	38.508	474.87
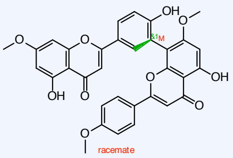	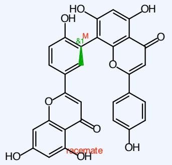	N203	N1148	0.88118	0.2599999	50.700001	50.44	424.5
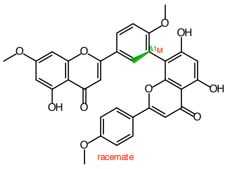	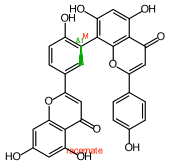	N378	N1148	0.88118	1.4	50.700001	49.3	414.91
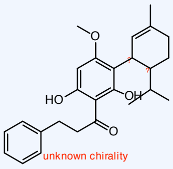	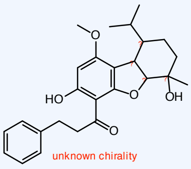	N693	N1177	0.84015	5.5999999	68.400002	62.8	392.87
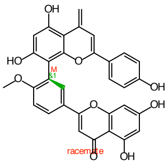	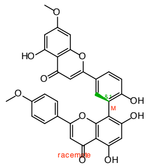	N220	N1138	0.87747	0.3000000	47.130001	46.83	382.18
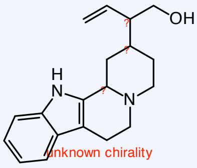	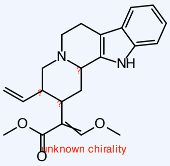	N804	N1186	0.80669	7.5100002	81.099998	73.59	380.69
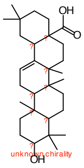	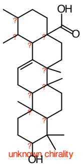	N123	N1115	0.90226	0.0390000	36	35.961	367.94
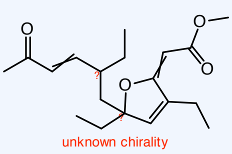	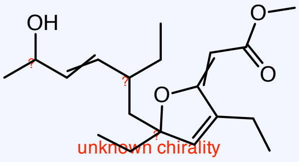	N401	N1104	0.91811	1.5604362	31.013609	29.453	359.66
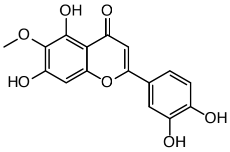	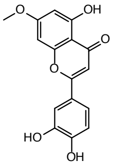	N763	N1137	0.88944	6.5999999	46	39.4	356.36
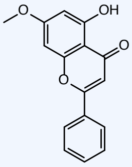	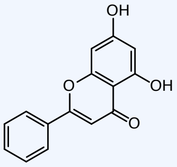	N577	N1094	0.92368	3.98	29.799999	25.82	338.33
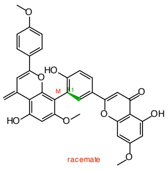	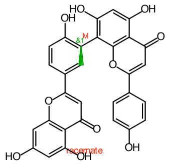	N206	N1148	0.84897	0.2599999	50.700001	50.44	333.97

Structure 1	Structure 2	ID 1	ID 2	Similarity	Activity 1	Activity 2	Delta Activity	SALI
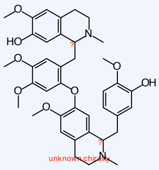	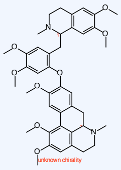	N388	N1122	0.88362	1.5	40.299999	38.8	333.38
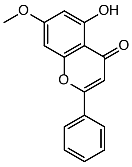	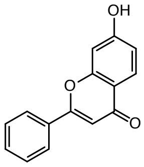	N577	N1170	0.80337	3.98	65.5	61.52	312.87
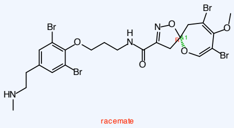	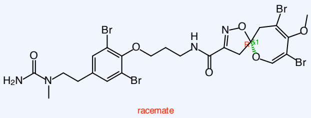	N377	N1041	0.93922	1.4	20	18.6	306.02
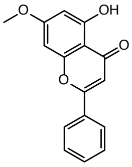	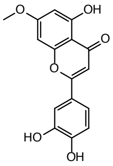	N577	N1137	0.85878	3.98	46	42.02	297.56
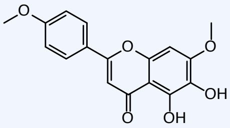	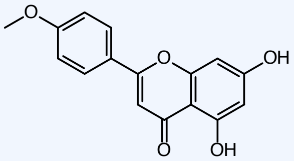	N867	N1134	0.87552	9	44.325073	35.325	283.77
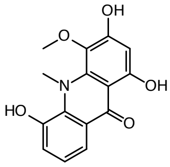	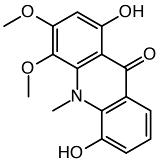	N861	N1095	0.92493	8.8999996	29.9	21	279.74
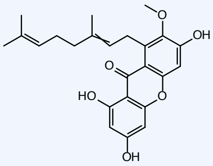	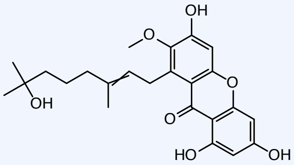	N729	N1091	0.91993	6	28	22	274.76
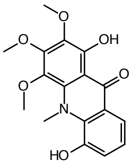	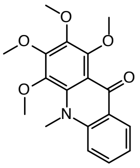	N738	N1152	0.82451	6.0999999	53	46.9	267.25
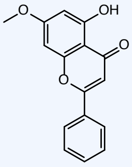	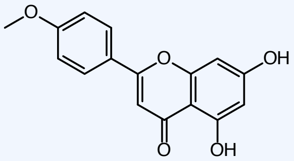	N577	N1134	0.84732	3.98	44.325073	40.345	264.25
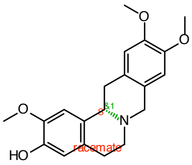	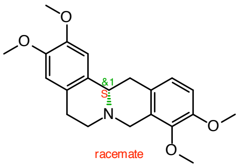	N408	N1108	0.88064	1.6	32.599998	31	259.72
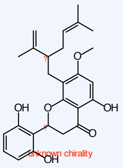	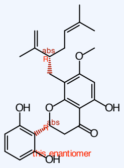	N96	N997	0.93686	0.0106	15.963046	15.952	252.66

Structure 1	Structure 2	ID 1	ID 2	Similarity	Activity 1	Activity 2	Delta Activity	SALI
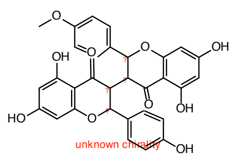	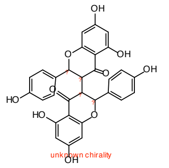	N322	N795	0.97238	0.97032207	7.3000002	6.3297	229.21
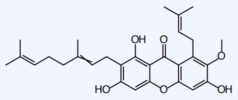	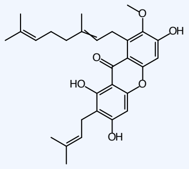	N720	N743	0.99883	6	6.2685881	0.26859	229.13
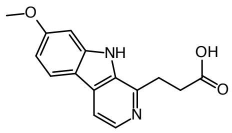	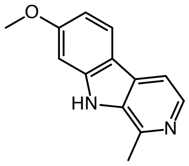	N351	N1117	0.83953	1.1617441	37.691902	36.53	227.64
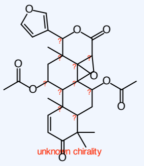	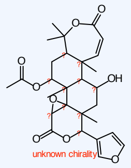	N701	N1118	0.85813	5.752852	37.896145	32.143	226.57
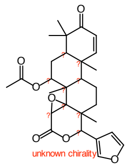	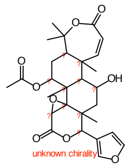	N382	N1118	0.83618	1.4920259	37.896145	36.404	222.22
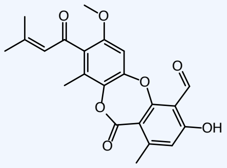	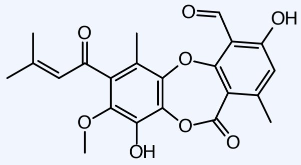	N520	N1052	0.9103	3.0999999	22	18.9	210.7
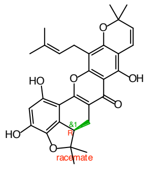	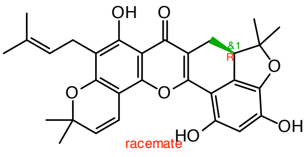	N267	N639	0.97947	0.55000001	4.8000002	4.25	207.01
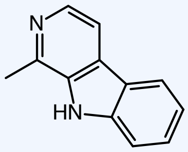	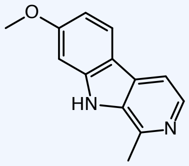	N387	N1117	0.81874	1.5	37.691902	36.192	199.66
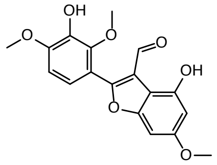	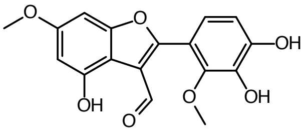	N740	N1022	0.94027	6.0999999	17.9	11.8	197.56
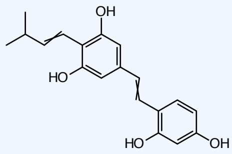	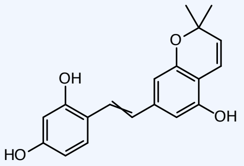	N286	N1099	0.84495	0.64028746	30.28899	29.649	191.22
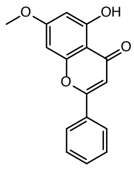	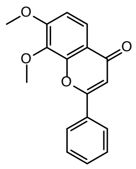	N577	N1123	0.80401	3.98	40.900002	36.92	188.37

Structure 1	Structure 2	ID 1	ID 2	Similarity	Activity 1	Activity 2	Delta Activity	SALI
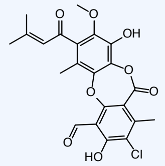	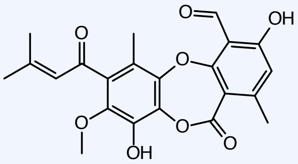	N792	N1052	0.91993	7.1999998	22	14.8	184.84
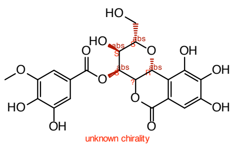	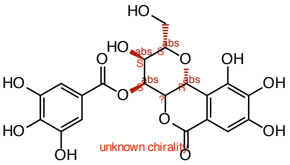	N358	N844	0.9608	1.3	8.3999996	7.1	181.14
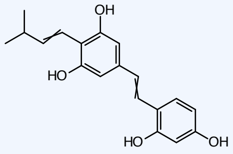	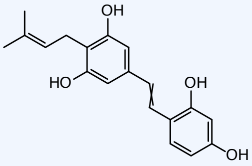	N286	N1084	0.85759	0.6402874(	26.251785	25.611	179.84
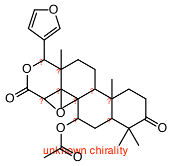	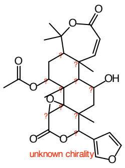	N682	N1118	0.81693	5.4273667	37.896145	32.469	177.36
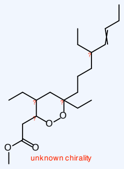	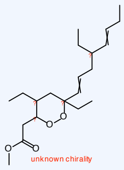	N370	N1054	0.87946	1.353928	22.694511	21.341	177.04
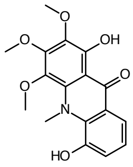	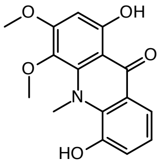	N738	N1095	0.86177	6.0999999	29.9	23.8	172.17
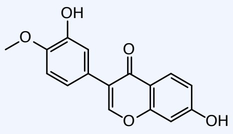	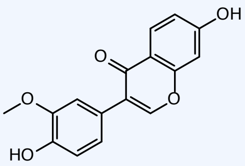	N355	N771	0.96694	1.2	6.8000002	5.6	169.38
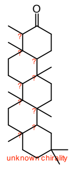	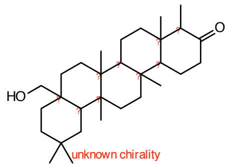	N812	N1013	0.94476	7.6999998	17	9.3	168.35
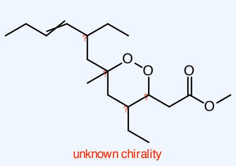	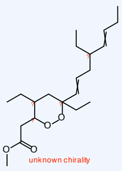	N245	N1054	0.86752	0.40000001	22.694511	22.295	168.29
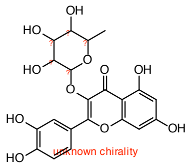	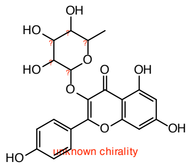	N86	N927	0.93418	0.0020000(	10.625	10.623	161.4
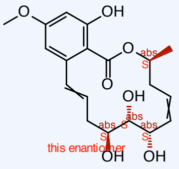	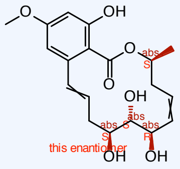	N114	N782	0.95559	0.02	7	6.98	157.18

Structure 1	Structure 2	ID 1	ID 2	Similarity	Activity 1	Activity 2	Delta Activity	SALI
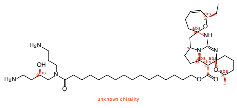	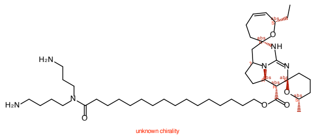	N198	N621	0.97269	0.2399999	4.5	4.26	155.96
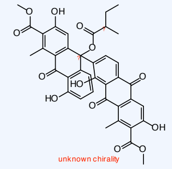	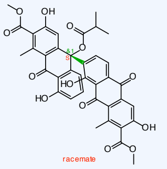	N418	N820	0.96006	1.7	7.9000001	6.2	155.22
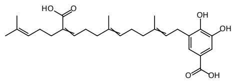	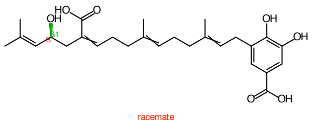	N775	N983	0.94673	7	15	8	150.17
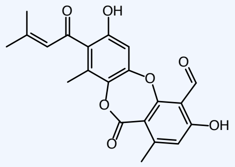	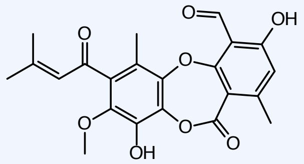	N779	N1052	0.8973	7	22	15	146.05
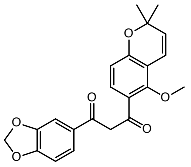	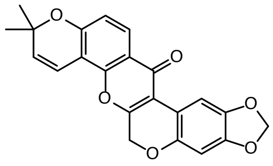	N878	N1112	0.82392	9.5	33.299999	23.8	135.17
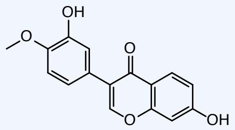	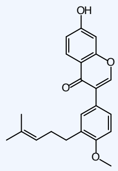	N355	N1053	0.83872	1.2	22.5	21.3	132.07
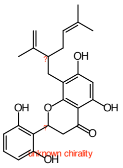	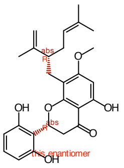	N98	N997	0.87695	0.0108	15.963046	15.952	129.64
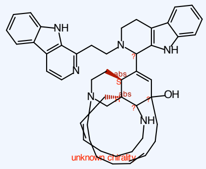	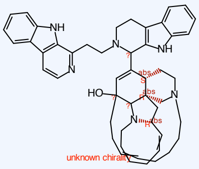	N306	N880	0.9308	0.8000000	9.6000004	8.8	127.17
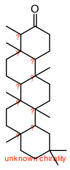	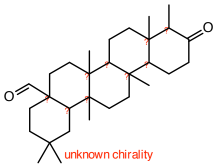	N812	N986	0.94215	7.6999998	15	7.3	126.19
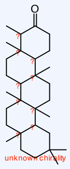	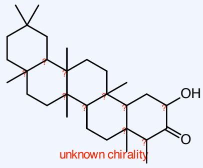	N812	N999	0.93418	7.6999998	16	8.3	126.11
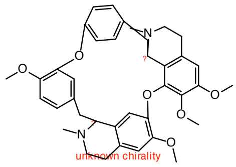	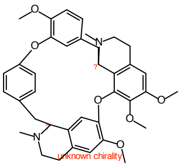	N241	N207	0.99915	0.3709354	0.2649538	0.10598	124.43

Structure 1	Structure 2	ID 1	ID 2	Similarity	Activity 1	Activity 2	Delta Activity	SALI
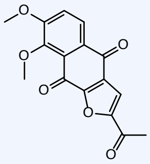	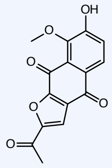	N496	N946	0.92895	2.96	11.8	8.84	124.42
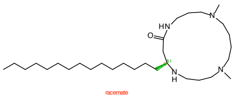	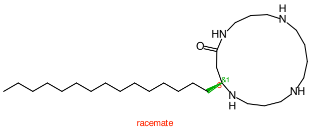	N186	N987	0.87829	0.2	15	14.8	121.6
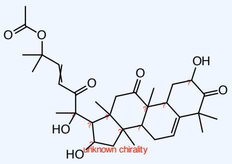	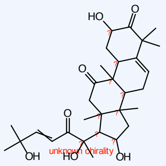	N406	N811	0.94943	1.6	7.6999998	6.1	120.63
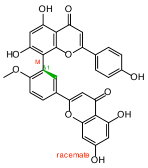	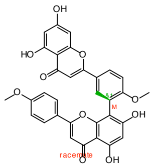	N219	N545	0.97311	0.30000001	3.5	3.2	119.01
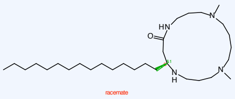	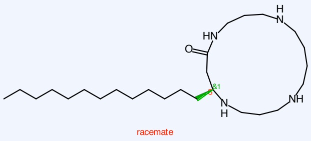	N186	N974	0.88214	0.2	14	13.8	117.09
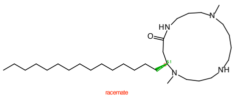	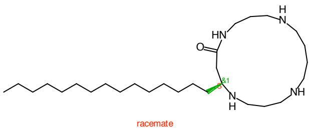	N199	N987	0.87271	0.23999999	15	14.76	115.95
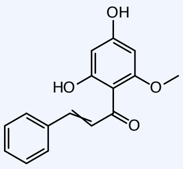	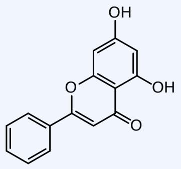	N876	N1094	0.82229	9.5	29.799999	20.3	114.23
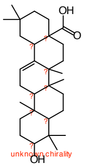	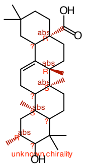	N123	N1045	0.81333	0.0390000	21.1	21.061	112.82
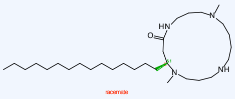	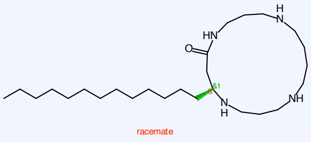	N199	N974	0.87642	0.23999999	14	13.76	111.34
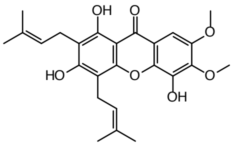	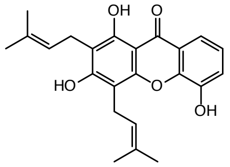	N362	N998	0.86745	1.3	16	14.7	110.91
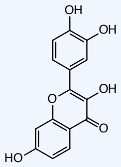	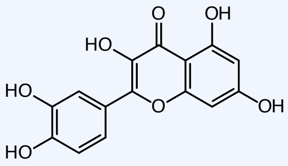	N837	N1042	0.88713	8.1999998	20.099339	11.899	105.42

Structure 1	Structure 2	ID 1	ID 2	Similarity	Activity 1	Activity 2	Delta Activity	SALI
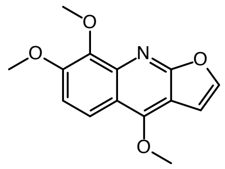	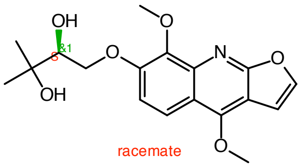	N874	N1066	0.85377	9.3000002	24.5	15.2	103.95
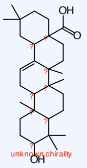	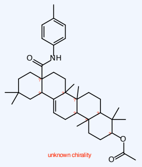	N123	N1012	0.83274	0.0390000(	17	16.961	101.4
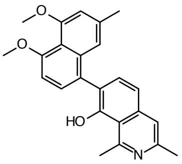	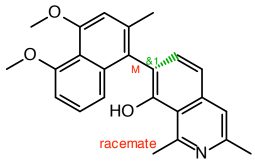	N361	N921	0.90883	1.3	10.5	9.2	100.91
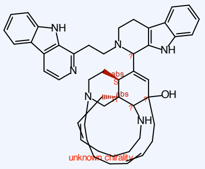	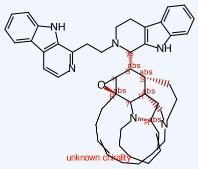	N306	N1003	0.84621	0.80000001	16.299999	15.5	100.79
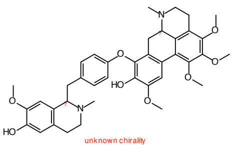	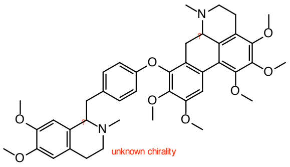	N366	N798	0.94042	1.3158381	7.3045239	5.9887	100.51
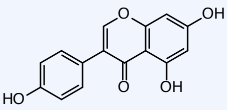	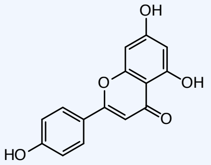	N436	N1018	0.84495	2	17.4	15.4	99.323
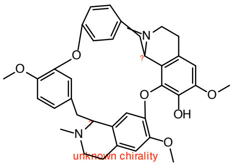	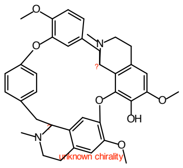	N176	N142	0.99913	0.17183505	0.08542469	0.08641	98.883
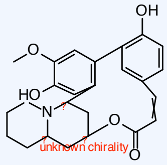	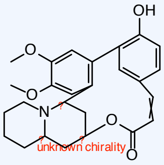	N768	N934	0.95739	6.6999998	10.9	4.2	98.578
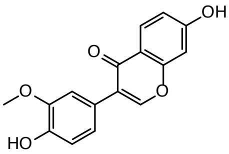	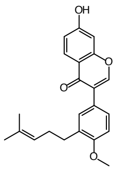	N771	N1053	0.83872	6.8000002	22.5	15.7	97.346
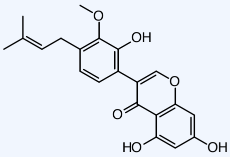	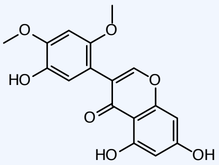	N748	N1073	0.80821	6.3000002	24.799999	18.5	96.459
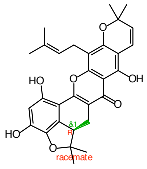	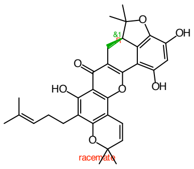	N267	N628	0.95752	0.55000001	4.645926	4.0959	96.428

Structure 1	Structure 2	ID 1	ID 2	Similarity	Activity 1	Activity 2	Delta Activity	SALI
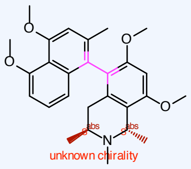	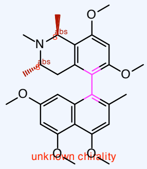	N864	N996	0.92652	9	15.9	6.9	93.902
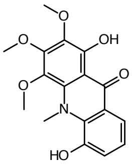	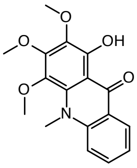	N738	N979	0.90712	6.0999999	14.7	8.6	92.591
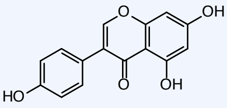	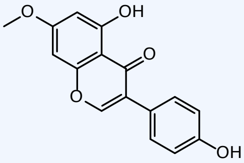	N436	N848	0.92856	2	8.5500002	6.55	91.687
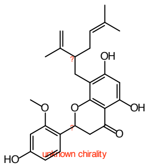	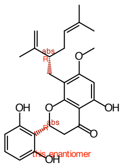	N451	N997	0.84799	2.0999999	15.963046	13.863	91.198
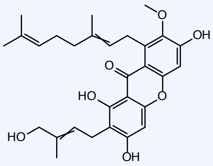	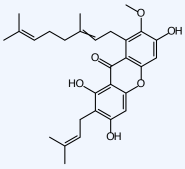	N524	N743	0.96668	3.2350938	6.2685881	3.0335	91.033
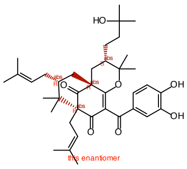	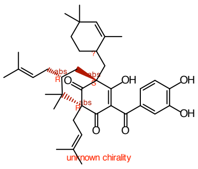	N655	N984	0.88924	5	15	10	90.288
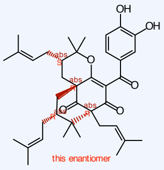	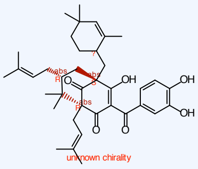	N651	N984	0.88773	5	15	10	89.069
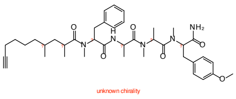	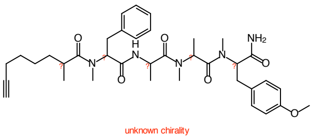	N600	N725	0.98078	4.3000002	6	1.7	88.435
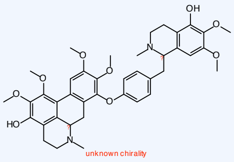	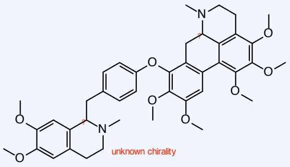	N229	N798	0.91946	0.3405832(	7.3045239	6.9639	86.463
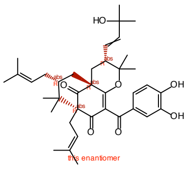	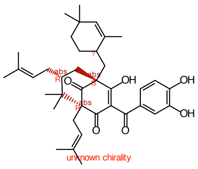	N654	N984	0.88041	5	15	10	83.619
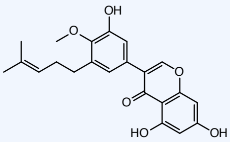	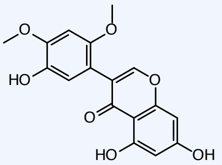	N858	N1073	0.807	8.6999998	24.799999	16.1	83.422

Structure 1	Structure 2	ID 1	ID 2	Similarity	Activity 1	Activity 2	Delta Activity	SALI
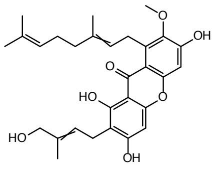	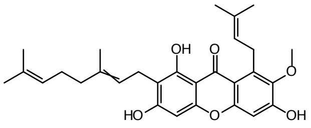	N524	N720	0.96668	3.2350938	6	2.7649	82.972
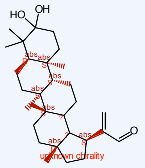	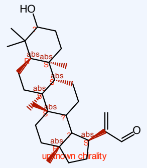	N550	N925	0.91556	3.5999999	10.596756	6.9968	82.862
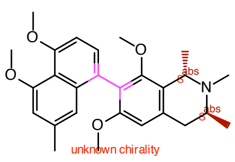	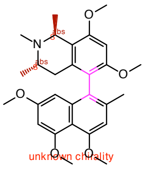	N442	N996	0.83011	2	15.9	13.9	81.819
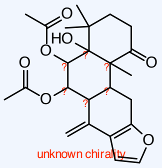	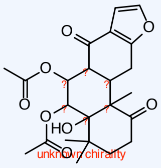	N492	N328	0.97642	2.9000001	1	1.9	80.578
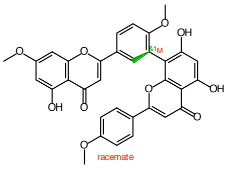	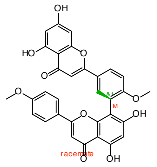	N378	N545	0.97381	1.4	3.5	2.1	80.175
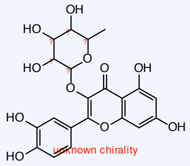	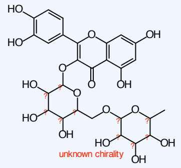	N86	N785	0.91135	0.0020000(	7.0999999	7.098	80.064
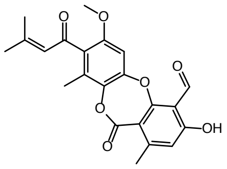	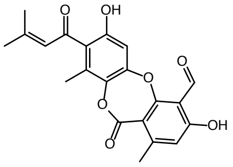	N520	N779	0.95123	3.0999999	7	3.9	79.97
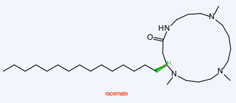	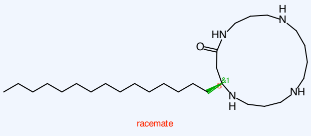	N178	N987	0.81061	0.1800000	15	14.82	78.251
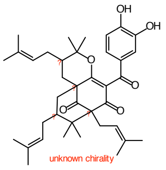	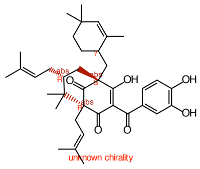	N457	N984	0.8351	2.2	15	12.8	77.622
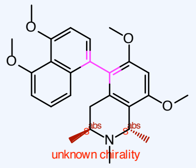	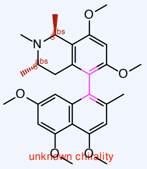	N616	N996	0.85242	4.5	15.9	11.4	77.246
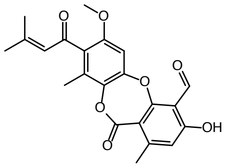	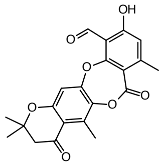	N520	N957	0.88025	3.0999999	12.3	9.2	76.829

Structure 1	Structure 2	ID 1	ID 2	Similarity	Activity 1	Activity 2	Delta Activity	SALI
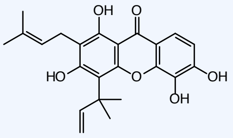	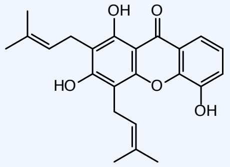	N344	N998	0.80512	1.1	16	14.9	76.457
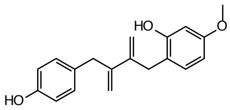	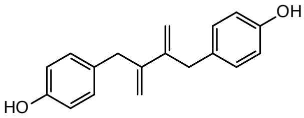	N866	N1043	0.84958	9	20.5	11.5	76.452
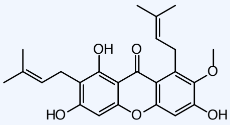	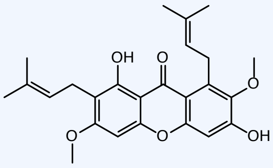	N589	N784	0.96054	4.0999999	7.0673671	2.9674	75.208
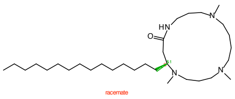	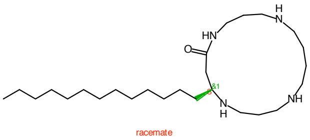	N178	N974	0.81554	0.1800000[illegible]	14	13.82	74.923
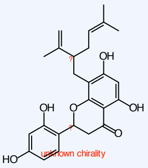	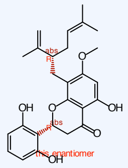	N482	N997	0.82157	2.5999999	15.963046	13.363	74.891
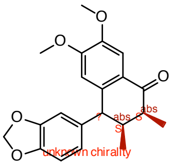	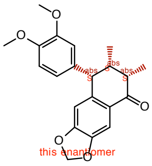	N205	N826	0.89608	0.2599999[illegible]	8	7.74	74.478
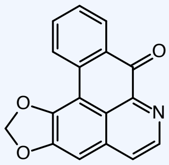	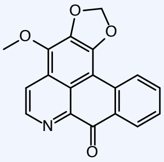	N468	N966	0.85816	2.3699999	12.8	10.43	73.533
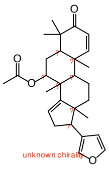	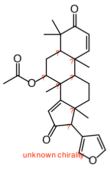	N19	N750	0.92901	1.21	6.4000001	5.19	73.112
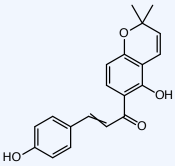	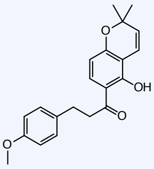	N539	N1009	0.81482	3.4000001	16.92	13.52	73.01
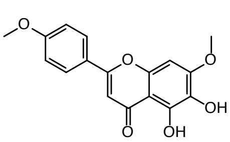	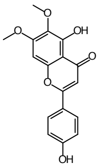	N867	N1008	0.89051	9	16.9	7.9	72.156
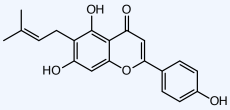	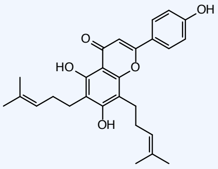	N766	N1046	0.7981	6.6999998	21.172588	14.473	71.681

Structure 1	Structure 2	ID 1	ID 2	Similarity	Activity 1	Activity 2	Delta Activity	SALI
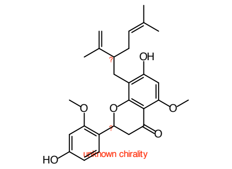	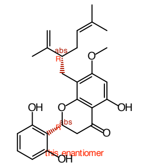	N469	N997	0.81007	2.4000001	15.963046	13.563	71.412
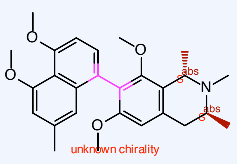	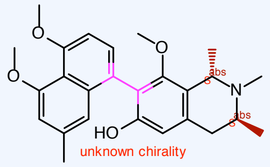	N442	N662	0.95739	2	5	3	70.413
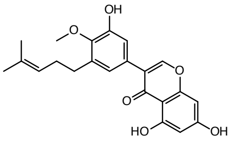	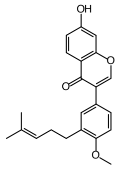	N858	N1053	0.80103	8.6999998	22.5	13.8	69.356
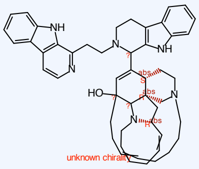	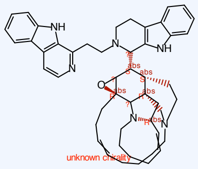	N880	N1003	0.90325	9.6000004	16.299999	6.7	69.25
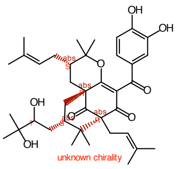	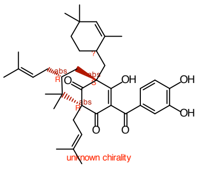	N656	N984	0.85528	5	15	10	69.098
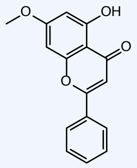	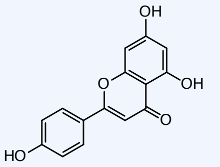	N577	N1018	0.80547	3.98	17.4	13.42	68.986
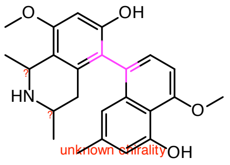	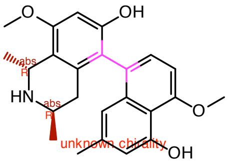	N266	N665	0.93395	0.5286225	5.0829086	4.5543	68.953
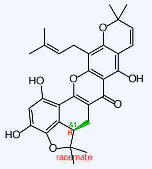	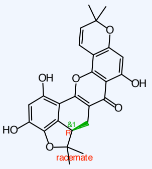	N267	N847	0.88416	0.5500000	8.5167503	7.9668	68.773
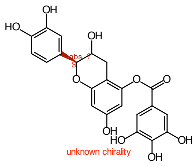	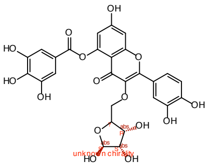	N352	N977	0.80547	1.2	14.5	13.3	68.37
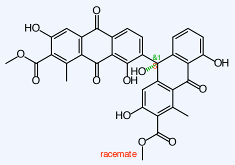	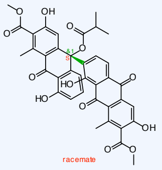	N582	N820	0.94068	4	7.9000001	3.9	65.75
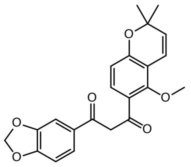	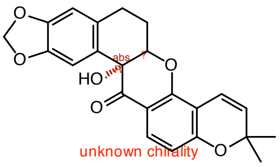	N878	N1051	0.80862	9.5	22	12.5	65.315

Structure 1	Structure 2	ID 1	ID 2	Similarity	Activity 1	Activity 2	Delta Activity	SALI
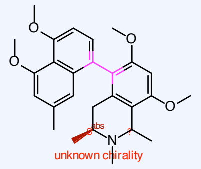	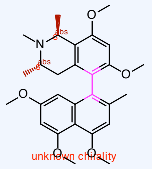	N605	N996	0.82114	4.4000001	15.9	11.5	64.295
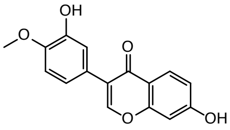	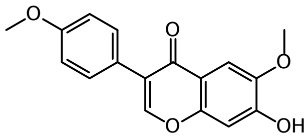	N355	N935	0.84721	1.2	10.91	9.71	63.553
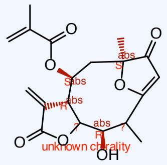	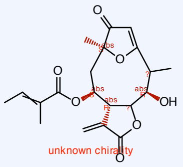	N603	N824	0.94314	4.4000001	8	3.6	63.311
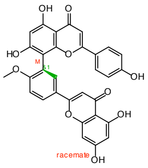	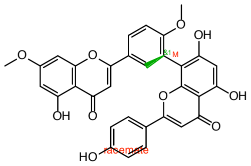	N219	N441	0.97311	0.30000001	2	1.7	63.225
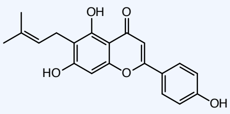	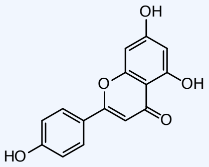	N766	N1018	0.82972	6.6999998	17.4	10.7	62.836
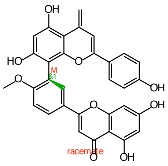	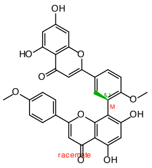	N220	N545	0.94777	0.30000001	3.5	3.2	61.262
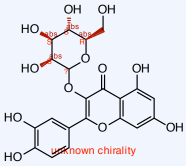	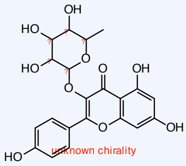	N88	N927	0.82657	0.0023000(	10.625	10.623	61.249
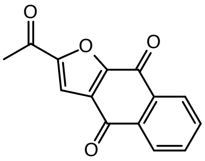	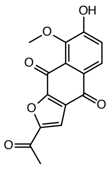	N85	N946	0.807	0.0020000(	11.8	11.798	61.131
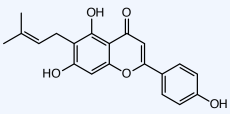	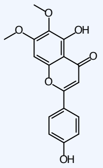	N766	N1008	0.83272	6.6999998	16.9	10.2	60.976
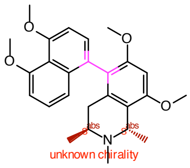	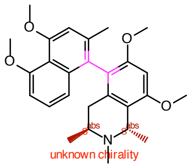	N616	N864	0.92602	4.5	9	4.5	60.827
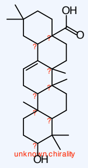	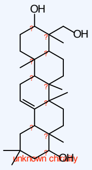	N123	N894	0.83607	0.0390000(	10	9.961	60.763

Structure 1	Structure 2	ID 1	ID 2	Similarity	Activity 1	Activity 2	Delta Activity	SALI
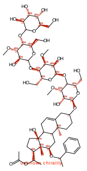	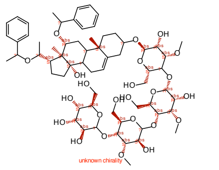	N802	N698	0.96995	7.5	5.6999998	1.8	59.904
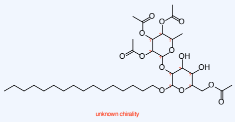	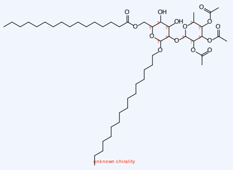	N544	N836	0.92215	3.5	8.1000004	4.6	59.087
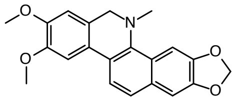	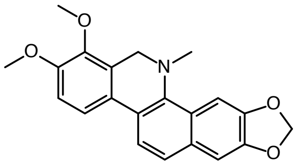	N645	N926	0.90311	4.9000001	10.6	5.7	58.832
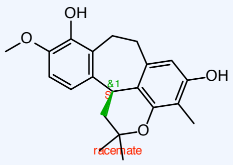	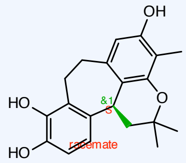	N485	N741	0.94027	2.6438484	6.1277084	3.4839	58.329
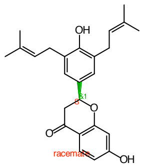	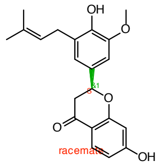	N808	N972	0.89839	7.6999998	13.6	5.9	58.064
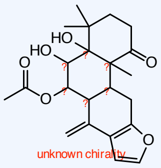	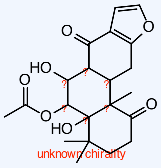	N705	N165	0.90236	5.8000002	0.14	5.66	57.97
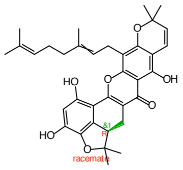	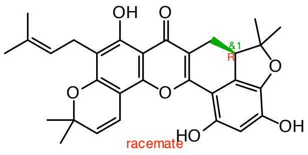	N364	N639	0.93904	1.3099999	4.8000002	3.49	57.247
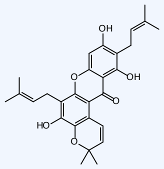	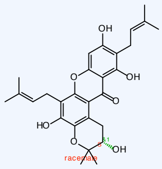	N295	N718	0.90645	0.69999999	6	5.3	56.654
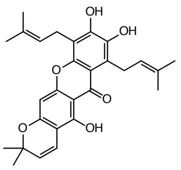	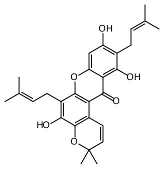	N719	N295	0.90332	6	0.69999999	5.3	54.819
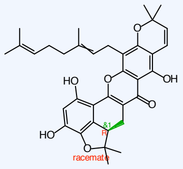	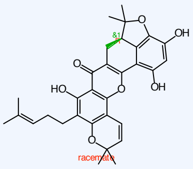	N364	N628	0.93878	1.3099999	4.645926	3.3359	54.488
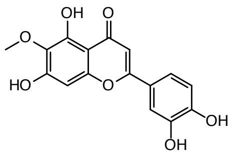	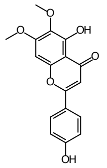	N763	N1008	0.81067	6.5999999	16.9	10.3	54.403

Structure 1	Structure 2	ID 1	ID 2	Similarity	Activity 1	Activity 2	Delta Activity	SALI
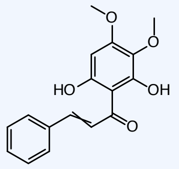	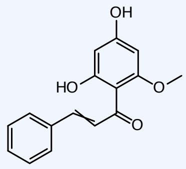	N471	N876	0.86791	2.4000001	9.5	7.1	53.751
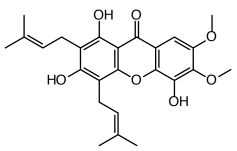	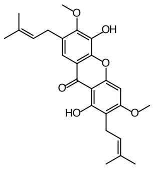	N362	N794	0.88885	1.3	7.25	5.95	53.53
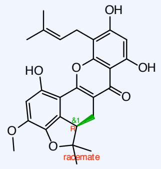	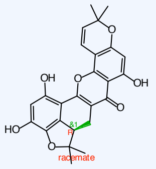	N179	N847	0.84045	0.1800000	8.5167503	8.3368	52.251
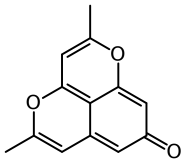	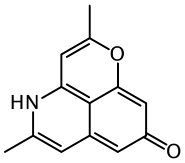	N633	N117	0.90963	4.6999998	0.0234999	4.6765	51.75
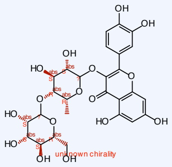	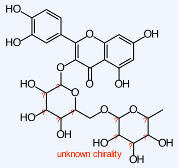	N89	N785	0.862	0.0029	7.0999999	7.0971	51.428
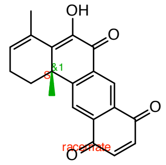	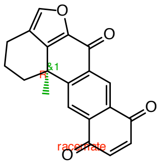	N872	N346	0.84089	9.1999998	1.1	8.1	50.907
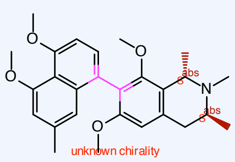	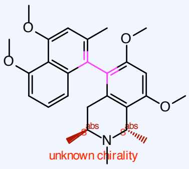	N442	N864	0.85706	2	9	7	48.973
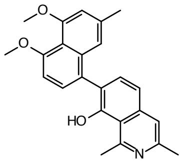	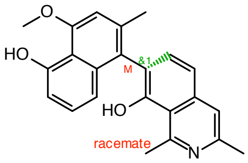	N361	N853	0.85025	1.3	8.6000004	7.3	48.748
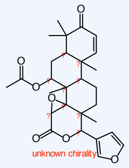	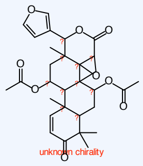	N382	N701	0.91238	1.4920259	5.752852	4.2608	48.631
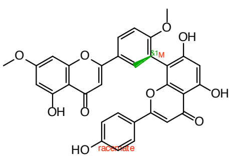	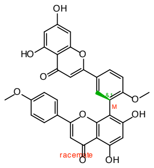	N441	N545	0.96914	2	3.5	1.5	48.613
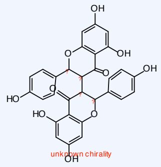	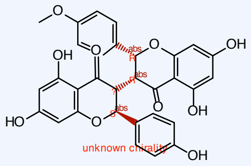	N795	N320	0.86861	7.3000002	0.9599999	6.34	48.253

Structure 1	Structure 2	ID 1	ID 2	Similarity	Activity 1	Activity 2	Delta Activity	SALI
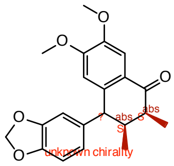	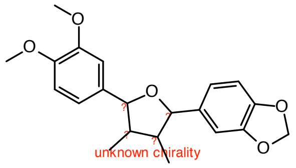	N205	N904	0.79546	0.2599999	10.100662	9.8407	48.111
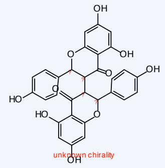	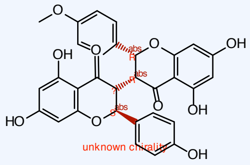	N795	N326	0.86861	7.3000002	1	6.3	47.949
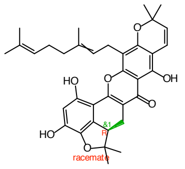	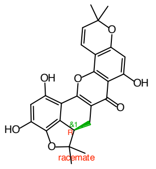	N364	N847	0.84883	1.3099999	8.5167503	7.2068	47.673
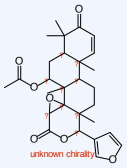	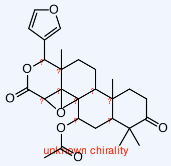	N382	N682	0.91712	1.4920259	5.4273667	3.9353	47.482
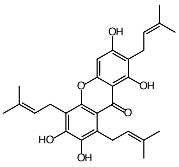	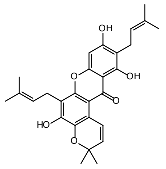	N722	N295	0.88707	6	0.6999999	5.3	46.93
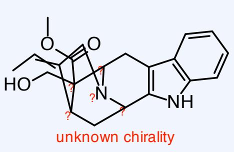	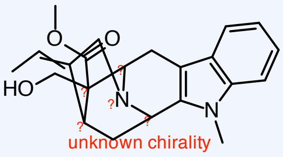	N478	N783	0.90377	2.5537214	7.0650177	4.5113	46.882
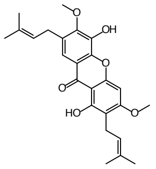	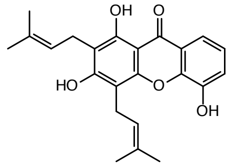	N794	N998	0.81324	7.25	16	8.75	46.851
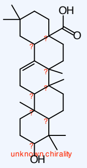	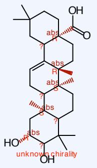	N123	N851	0.8152	0.0390000	8.6000004	8.561	46.325
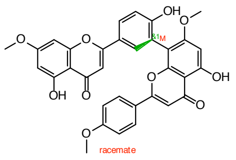	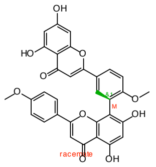	N203	N545	0.92999	0.2599999	3.5	3.24	46.277
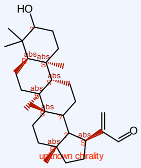	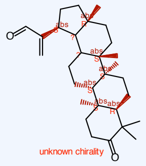	N789	N548	0.92074	7.1999998	3.533287	3.6667	46.262
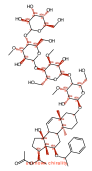	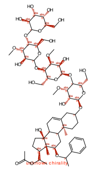	N883	N802	0.95452	9.6000004	7.5	2.1	46.178

Structure 1	Structure 2	ID 1	ID 2	Similarity	Activity 1	Activity 2	Delta Activity	SALI
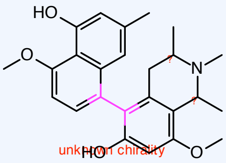	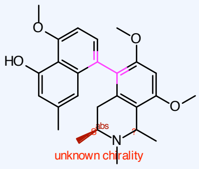	N368	N742	0.89329	1.3202387	6.1999998	4.8798	45.727
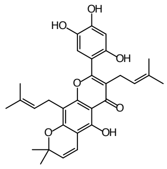	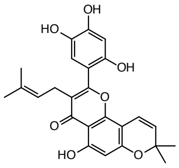	N340	N749	0.88255	1.04	6.4000001	5.36	45.636
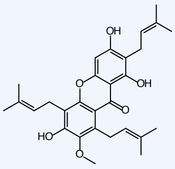	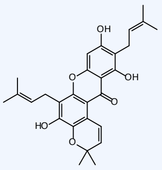	N675	N295	0.89965	5.2238231	0.69999999	4.5238	45.081
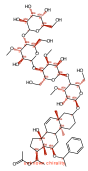	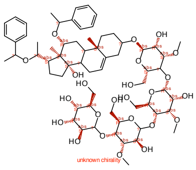	N883	N698	0.9132	9.6000004	5.6999998	3.9	44.932
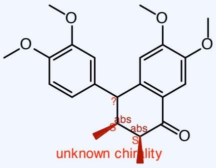	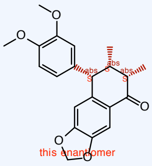	N223	N826	0.82831	0.31999999	8	7.68	44.732
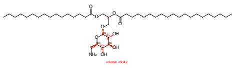	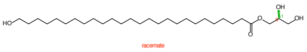	N622	N964	0.81653	4.5	12.7	8.2	44.693
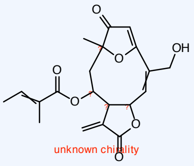	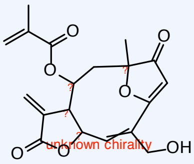	N310	N529	0.94314	0.80000001	3.3	2.5	43.966
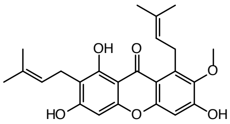	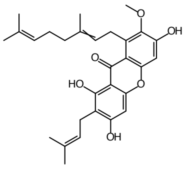	N589	N743	0.95062	4.0999999	6.2685881	2.1686	43.919
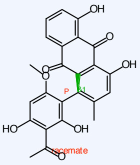	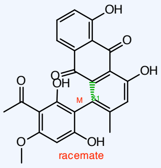	N213	N400	0.971	0.28	1.5423753	1.2624	43.529
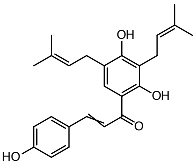	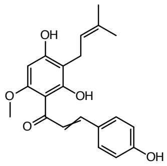	N307	N838	0.82892	0.80000001	8.1999998	7.4	43.254
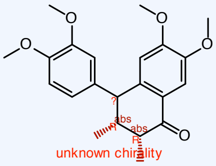	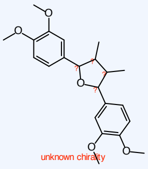	N187	N842	0.81211	0.2	8.3000002	8.1	43.111

Structure 1	Structure 2	ID 1	ID 2	Similarity	Activity 1	Activity 2	Delta Activity	SALI
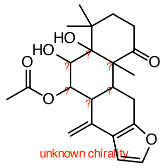	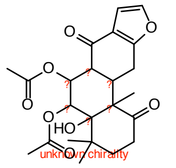	N705	N328	0.8886	5.8000002	1	4.8	43.087
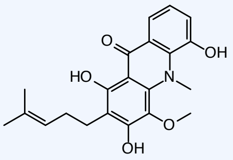	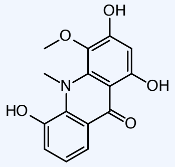	N218	N861	0.79964	0.3000000	8.8999996	8.6	42.922
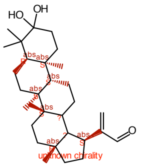	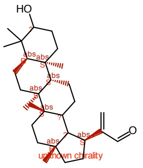	N550	N789	0.91556	3.5999999	7.1999998	3.6	42.634
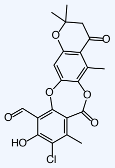	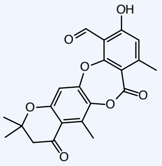	N852	N957	0.91248	8.6000004	12.3	3.7	42.278
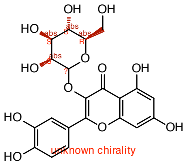	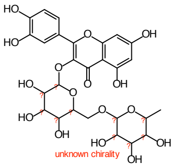	N88	N785	0.83121	0.0023000	7.0999999	7.0977	42.052
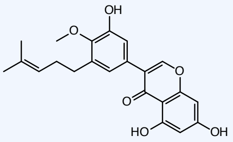	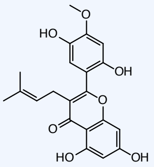	N858	N160	0.79546	8.6999998	0.12	8.58	41.948
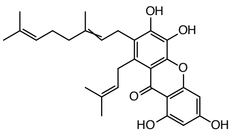	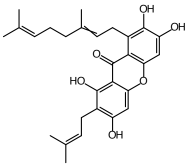	N448	N746	0.89795	2.0665154	6.3000002	4.2335	41.483
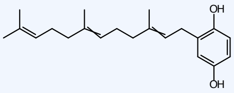	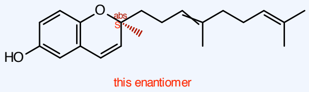	N371	N879	0.80199	1.37	9.5799999	8.21	41.463
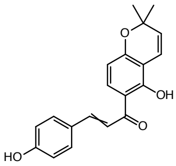	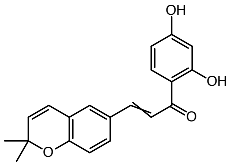	N539	N882	0.849	3.4000001	9.6000004	6.2	41.06
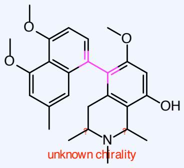	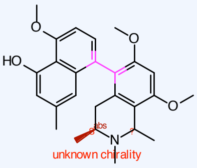	N235	N742	0.85745	0.3558477	6.1999998	5.8442	40.998
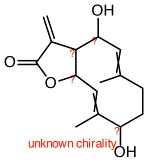	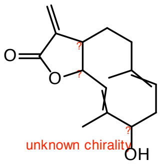	N385	N886	0.79952	1.5	9.6999998	8.2	40.902

Structure 1	Structure 2	ID 1	ID 2	Similarity	Activity 1	Activity 2	Delta Activity	SALI
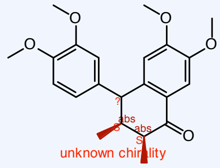	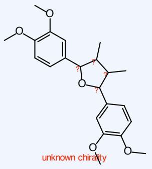	N223	N842	0.80473	0.3199999	8.3000002	7.98	40.866
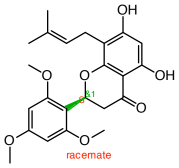	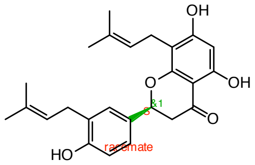	N363	N869	0.80879	1.3099999	9.1000004	7.79	40.742
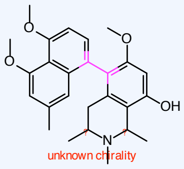	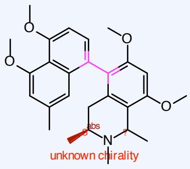	N235	N605	0.90055	0.3558477	4.4000001	4.0442	40.667
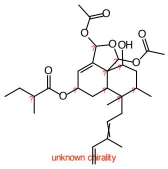	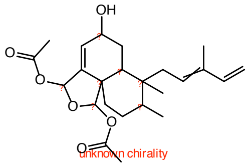	N281	N821	0.82039	0.62	7.9000001	7.28	40.532
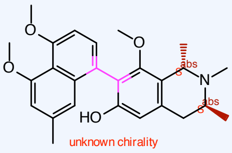	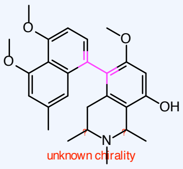	N662	N235	0.88503	5	0.3558477	4.6442	40.394
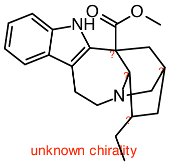	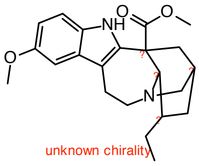	N381	N647	0.91351	1.4714427	4.9122138	3.4408	39.781
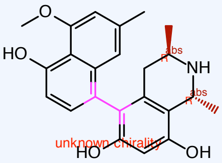	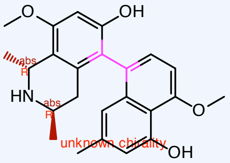	N254	N665	0.88355	0.4743721	5.0829086	4.6085	39.574
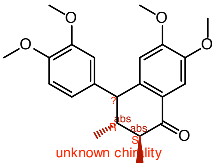	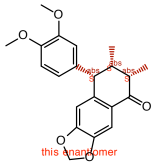	N282	N826	0.81337	0.63	8	7.37	39.489
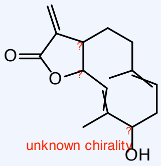	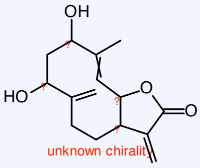	N886	N991	0.85804	9.6999998	15.3	5.6	39.448
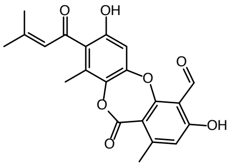	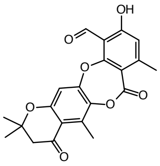	N779	N957	0.86564	7	12.3	5.3	39.445
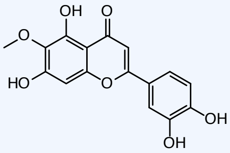	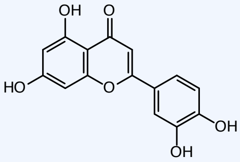	N763	N937	0.88831	6.5999999	11	4.4	39.395

Structure 1	Structure 2	ID 1	ID 2	Similarity	Activity 1	Activity 2	Delta Activity	SALI
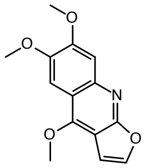	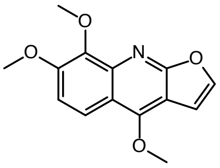	N511	N874	0.83981	3	9.3000002	6.3	39.329
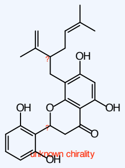	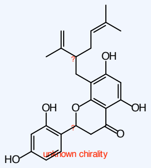	N98	N482	0.93414	0.0108	2.5999999	2.5892	39.313
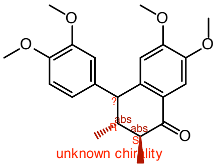	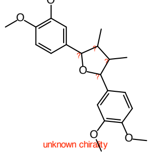	N282	N842	0.80473	0.63	8.3000002	7.67	39.279
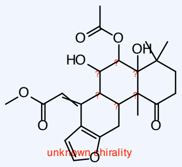	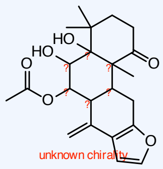	N197	N705	0.85787	0.2399999[illegible]	5.8000002	5.56	39.118
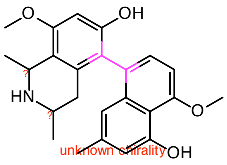	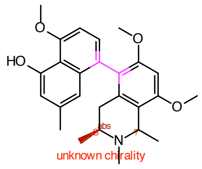	N266	N742	0.85465	0.5286225[illegible]	6.1999998	5.6714	39.019
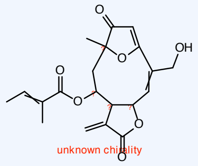	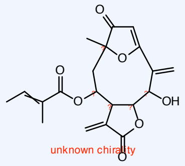	N310	N692	0.87695	0.8000000[illegible]	5.5999999	4.8	39.008
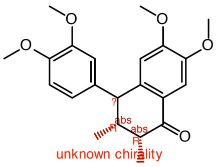	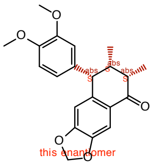	N187	N826	0.7983	0.2	8	7.8	38.671
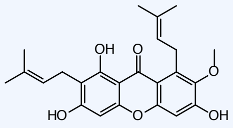	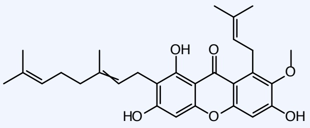	N589	N720	0.95062	4.0999999	6	1.9	38.48
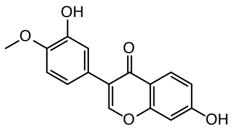	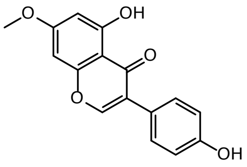	N355	N848	0.80737	1.2	8.5500002	7.35	38.156
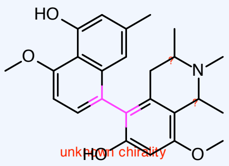	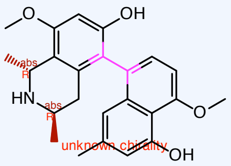	N368	N665	0.90109	1.3202387	5.0829086	3.7627	38.043
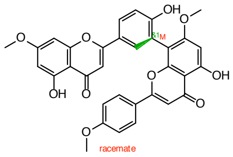	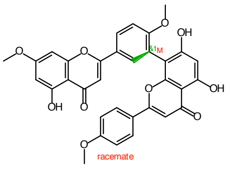	N203	N378	0.96993	0.2599999[illegible]	1.4	1.14	37.914

Structure 1	Structure 2	ID 1	ID 2	Similarity	Activity 1	Activity 2	Delta Activity	SALI
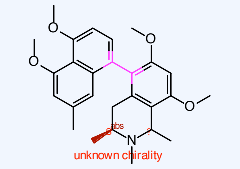	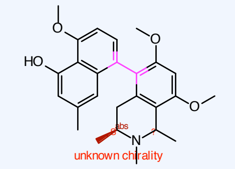	N605	N742	0.95243	4.4000001	6.1999998	1.8	37.836
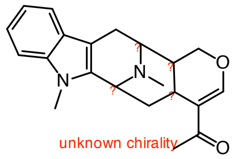	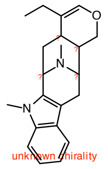	N884	N963	0.91928	9.6700001	12.7	3.03	37.538
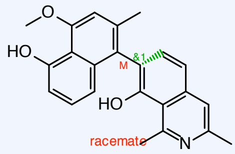	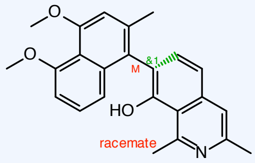	N853	N921	0.94883	8.6000004	10.5	1.9	37.132
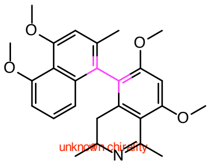	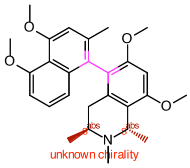	N638	N864	0.88589	4.7674356	9	4.2326	37.092
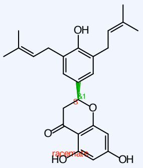	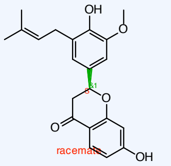	N737	N972	0.79546	6.0999999	13.6	7.5	36.668
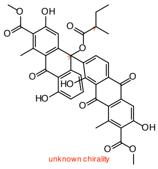	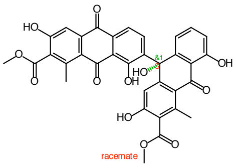	N418	N582	0.93686	1.7	4	2.3	36.428
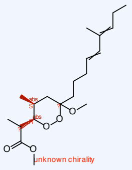	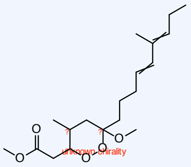	N345	N770	0.84296	1.1	6.8000002	5.7	36.297
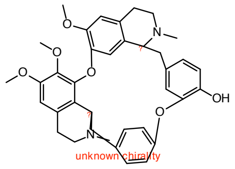	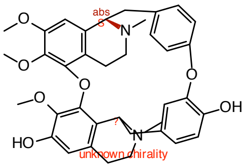	N264	N609	0.89039	0.5092625[illegible]	4.4819884	3.9727	36.244
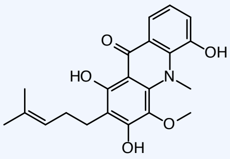	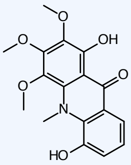	N218	N738	0.83921	0.3000000[illegible]	6.0999999	5.8	36.072
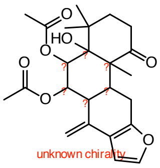	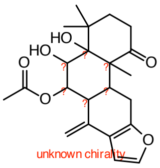	N492	N705	0.91934	2.9000001	5.8000002	2.9	35.955
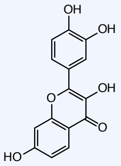	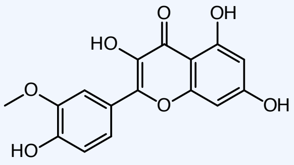	N837	N980	0.81814	8.1999998	14.7	6.5	35.741

Structure 1	Structure 2	ID 1	ID 2	Similarity	Activity 1	Activity 2	Delta Activity	SALI
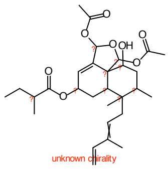	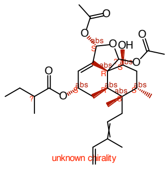	N281	N716	0.84849	0.62	6	5.38	35.509
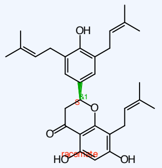	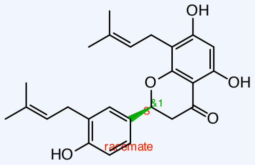	N676	N869	0.89277	5.3000002	9.1000004	3.8	35.437
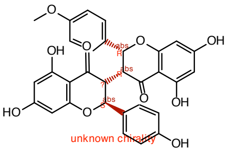	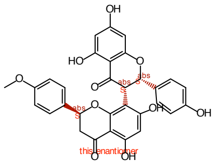	N320	N734	0.85706	0.9599999[illegible]	6	5.04	35.26
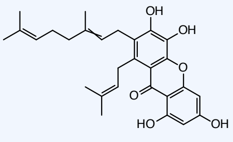	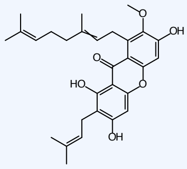	N448	N743	0.8808	2.0665154	6.2685881	4.2021	35.251
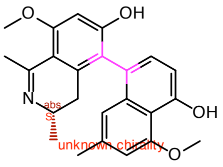	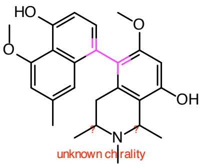	N681	N230	0.85645	5.4000001	0.3435565[illegible]	5.0564	35.224
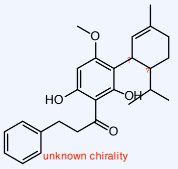	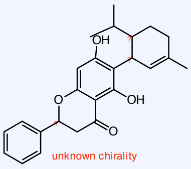	N693	N910	0.86621	5.5999999	10.3	4.7	35.131
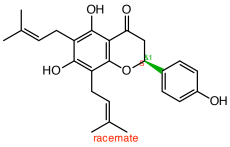	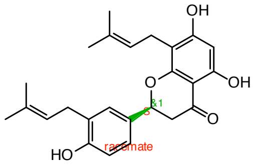	N573	N869	0.85143	3.9000001	9.1000004	5.2	34.999
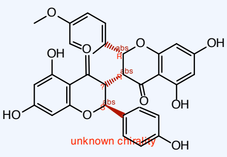	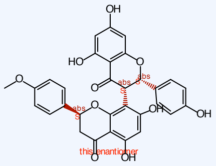	N326	N734	0.85706	1	6	5	34.981
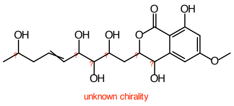	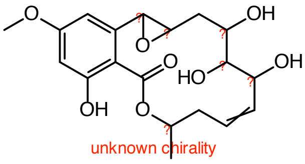	N99	N780	0.80002	0.0109	7	6.9891	34.948
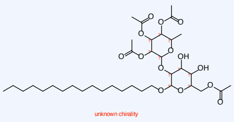	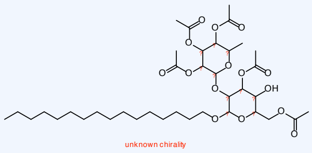	N544	N636	0.96563	3.5	4.6999998	1.2	34.909
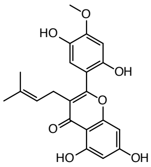	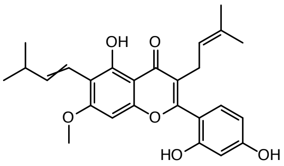	N160	N773	0.80547	0.12	6.9000001	6.78	34.853

Structure 1	Structure 2	ID 1	ID 2	Similarity	Activity 1	Activity 2	Delta Activity	SALI
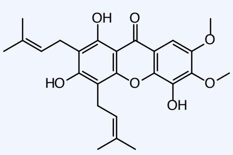	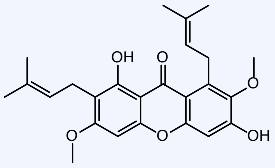	N362	N784	0.83409	1.3	7.0673671	5.7674	34.761
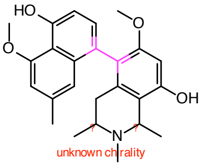	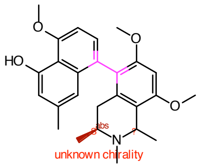	N230	N742	0.8305	0.3435565	6.1999998	5.8564	34.552
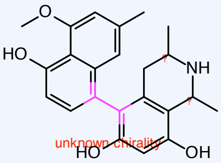	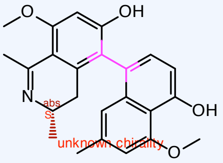	N136	N681	0.84324	0.0632496	5.4000001	5.3368	34.044
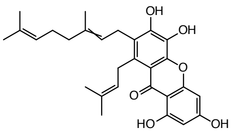	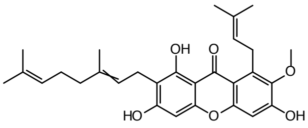	N448	N720	0.88352	2.0665154	6	3.9335	33.77
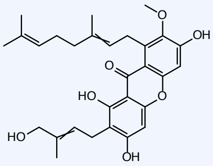	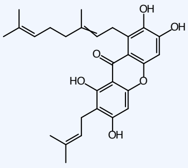	N524	N746	0.9091	3.2350938	6.3000002	3.0649	33.716
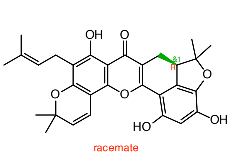	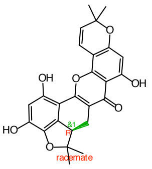	N639	N847	0.8896	4.8000002	8.5167503	3.7168	33.666
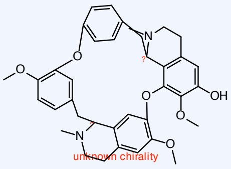	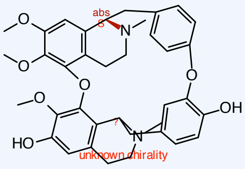	N191	N609	0.87306	0.2135617	4.4819884	4.2684	33.627
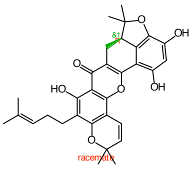	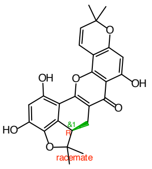	N628	N847	0.88448	4.645926	8.5167503	3.8708	33.507
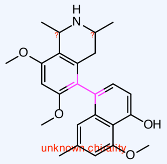	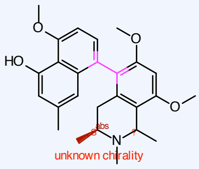	N356	N742	0.85291	1.2760671	6.1999998	4.9239	33.475
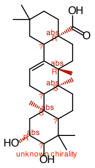	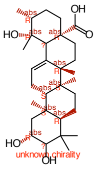	N851	N431	0.79789	8.6000004	1.841624	6.7584	33.44
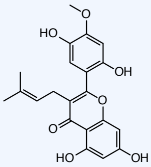	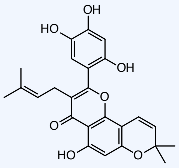	N160	N749	0.81211	0.12	6.4000001	6.28	33.425

Structure 1	Structure 2	ID 1	ID 2	Similarity	Activity 1	Activity 2	Delta Activity	SALI
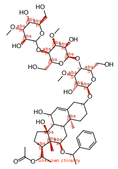	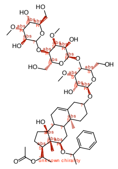	N757	N799	0.97307	6.5	7.4000001	0.9	33.414
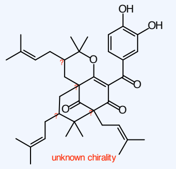	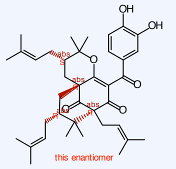	N457	N651	0.91599	2.2	5	2.8	33.327
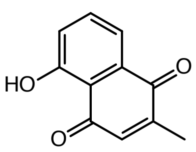	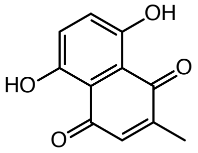	N210	N702	0.83324	0.27000001	5.79	5.52	33.102
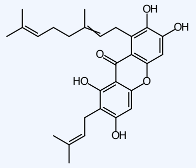	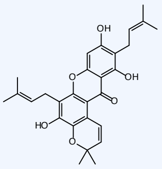	N746	N295	0.83055	6.3000002	0.69999999	5.6	33.048
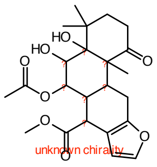	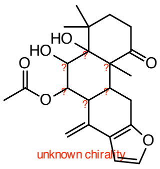	N407	N705	0.87267	1.6	5.8000002	4.2	32.985
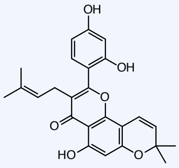	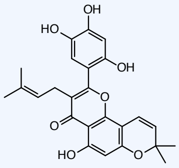	N617	N749	0.94215	4.5	6.4000001	1.9	32.844
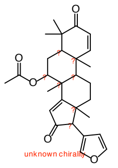	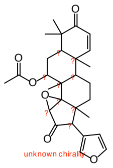	N750	N413	0.85508	6.4000001	1.6503562	4.7496	32.775
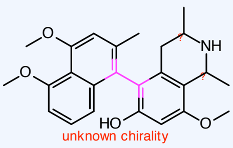	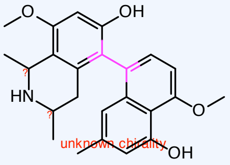	N646	N266	0.86621	4.9079509	0.52862251	4.3793	32.734
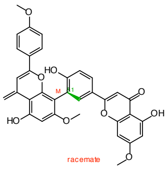	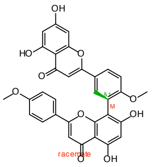	N206	N545	0.90073	0.25999999	3.5	3.24	32.637
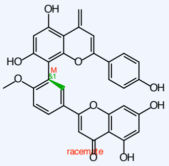	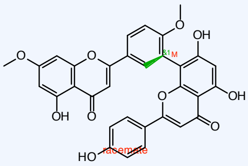	N220	N441	0.94777	0.30000001	2	1.7	32.546
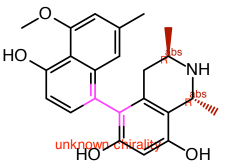	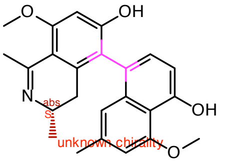	N254	N681	0.84666	0.47437215	5.4000001	4.9256	32.122

Structure 1	Structure 2	ID 1	ID 2	Similarity	Activity 1	Activity 2	Delta Activity	SALI
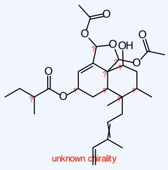	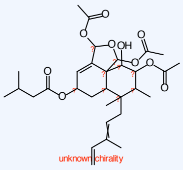	N281	N673	0.85675	0.62	5.1999998	4.58	31.972
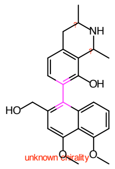	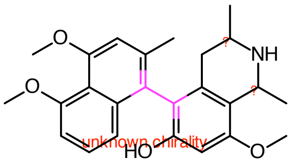	N103	N646	0.8466	0.01270727	4.9079509	4.8952	31.912
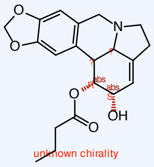	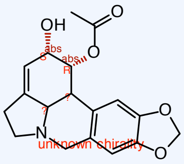	N339	N515	0.93686	1.0352538	3.0363095	2.0011	31.693
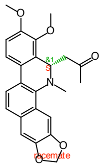	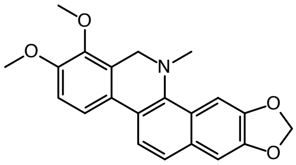	N699	N926	0.84495	5.6999998	10.6	4.9	31.603
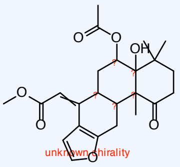	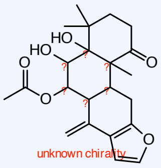	N289	N705	0.83667	0.64999998	5.8000002	5.15	31.531
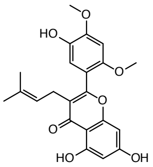	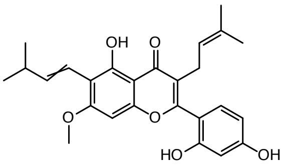	N291	N773	0.80121	0.66000003	6.9000001	6.24	31.39
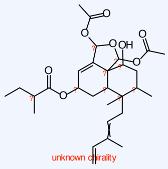	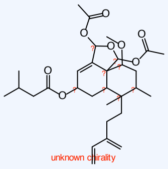	N281	N686	0.84393	0.62	5.5	4.88	31.268
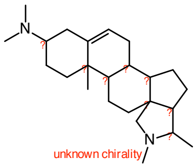	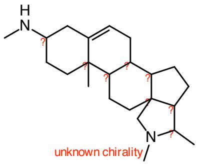	N341	N534	0.92342	1.04	3.3900001	2.35	30.687
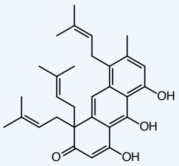	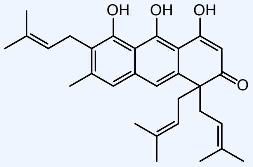	N488	N658	0.92395	2.7	5	2.3	30.243
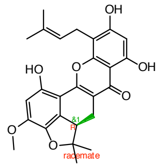	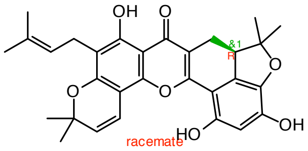	N179	N639	0.84651	0.18000001	4.8000002	4.62	30.099
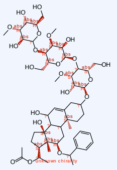	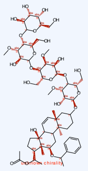	N757	N883	0.89699	6.5	9.6000004	3.1	30.094

Structure 1	Structure 2	ID 1	ID 2	Similarity	Activity 1	Activity 2	Delta Activity	SALI
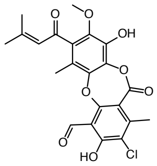	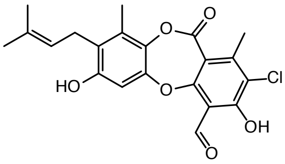	N792	N955	0.83335	7.1999998	12.2	5	30.002
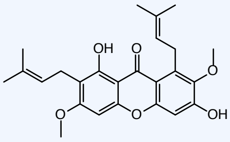	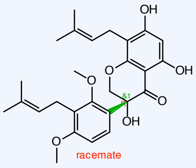	N784	N965	0.807	7.0673671	12.8	5.7326	29.703
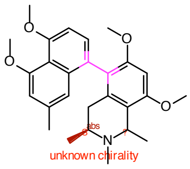	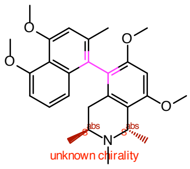	N605	N864	0.84495	4.4000001	9	4.6	29.668
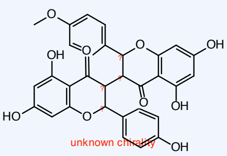	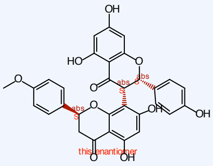	N322	N734	0.83026	0.97032207	6	5.0297	29.632
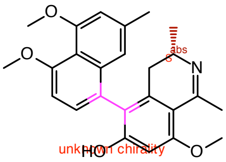	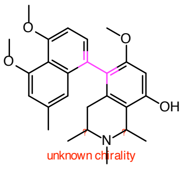	N604	N235	0.86238	4.4000001	0.35584772	4.0442	29.387
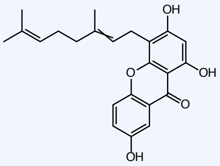	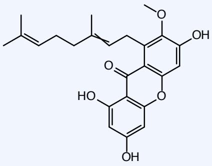	N360	N729	0.83822	1.3	6	4.7	29.051
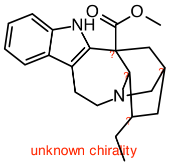	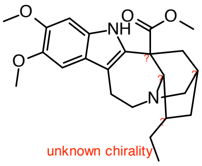	N381	N688	0.85929	1.4714427	5.5559001	4.0845	29.028
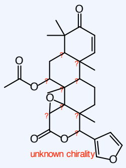	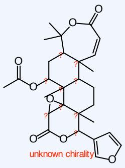	N382	N581	0.91348	1.4920259	4	2.508	28.986
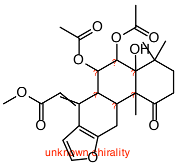	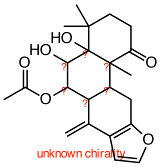	N327	N705	0.83409	1	5.8000002	4.8	28.93
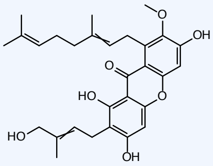	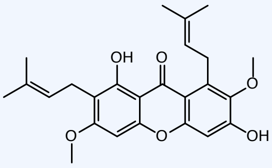	N524	N784	0.86599	3.2350938	7.0673671	3.8323	28.596
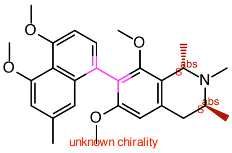	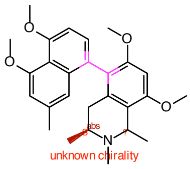	N442	N605	0.91522	2	4.4000001	2.4	28.308

Structure 1	Structure 2	ID 1	ID 2	Similarity	Activity 1	Activity 2	Delta Activity	SALI
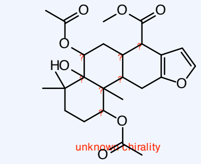	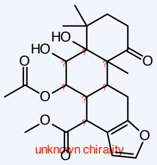	N754	N407	0.82657	6.5	1.6	4.9	28.253
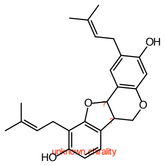	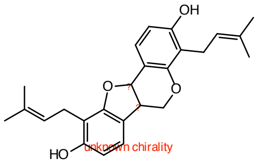	N756	N855	0.92206	6.5	8.6999998	2.2	28.228
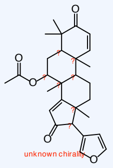	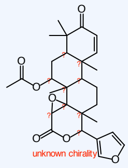	N750	N382	0.82475	6.4000001	1.4920259	4.908	28.006
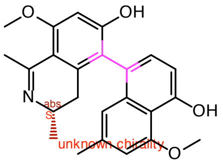	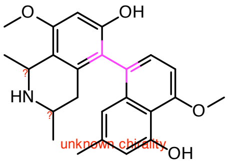	N681	N266	0.82465	5.4000001	0.52862251	4.8714	27.781
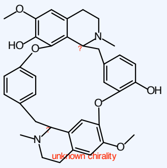	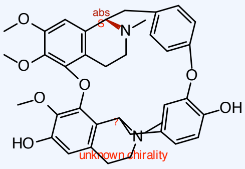	N234	N609	0.85036	0.35299999	4.4819884	4.129	27.592
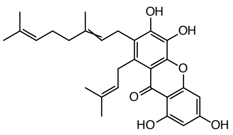	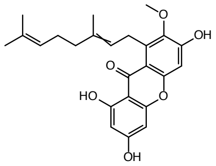	N448	N729	0.85706	2.0665154	6	3.9335	27.519
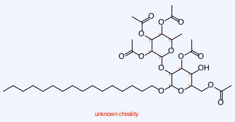	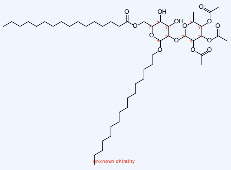	N636	N836	0.87525	4.6999998	8.1000004	3.4	27.255
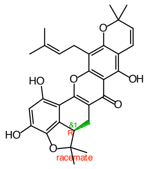	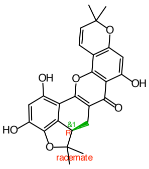	N267	N562	0.88416	0.55000001	3.7	3.15	27.192
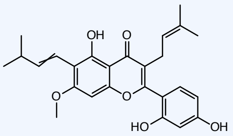	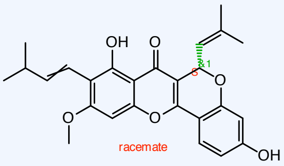	N773	N890	0.88963	6.9000001	9.8999996	3	27.182
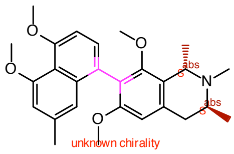	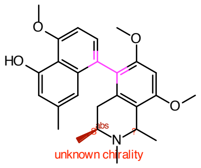	N442	N742	0.8434	2	6.1999998	4.2	26.821
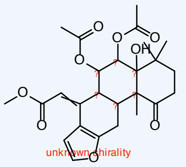	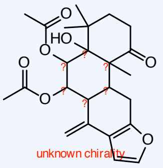	N327	N492	0.92913	1	2.9000001	1.9	26.811

Structure 1	Structure 2	ID 1	ID 2	Similarity	Activity 1	Activity 2	Delta Activity	SALI
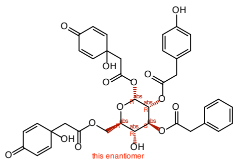	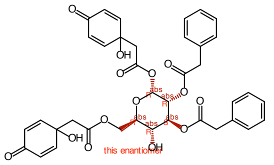	N305	N384	0.97356	0.8000000[illegible]	1.5	0.7	26.476
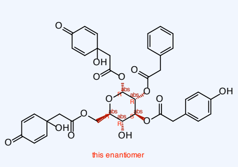	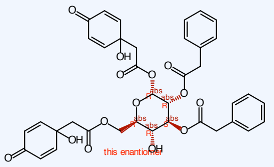	N304	N384	0.97356	0.8000000[illegible]	1.5	0.7	26.476
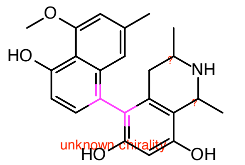	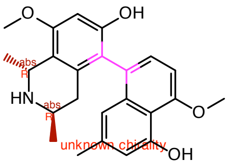	N136	N665	0.80862	0.0632496[illegible]	5.0829086	5.0197	26.229
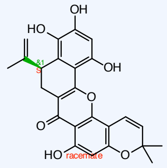	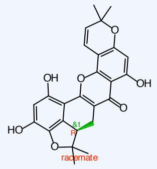	N772	N562	0.87793	6.9000001	3.7	3.2	26.215
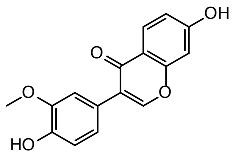	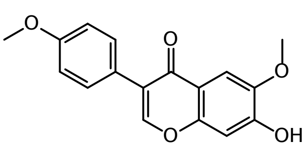	N771	N935	0.84268	6.8000002	10.91	4.11	26.126
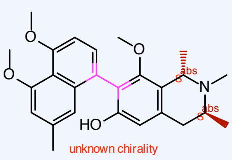	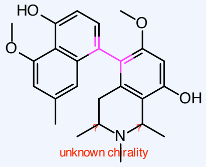	N662	N230	0.82079	5	0.3435565[illegible]	4.6564	25.984
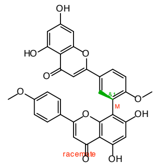	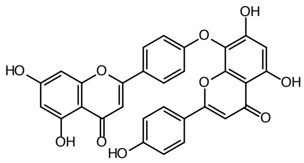	N545	N255	0.88322	3.5	0.4799999[illegible]	3.02	25.86
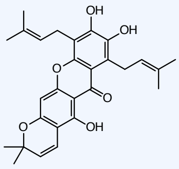	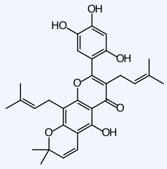	N719	N340	0.80698	6	1.04	4.96	25.697
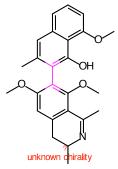	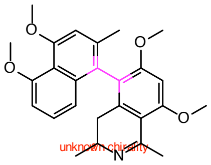	N201	N638	0.82404	0.2466175[illegible]	4.7674356	4.5208	25.692
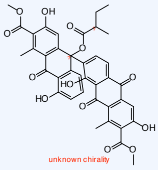	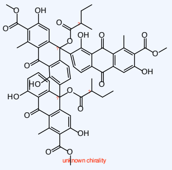	N418	N661	0.87057	1.7	5	3.3	25.497
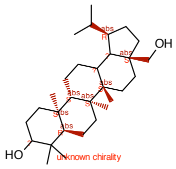	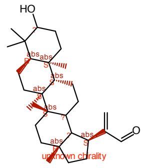	N787	N925	0.86606	7.1953335	10.596756	3.4014	25.395

Structure 1	Structure 2	ID 1	ID 2	Similarity	Activity 1	Activity 2	Delta Activity	SALI
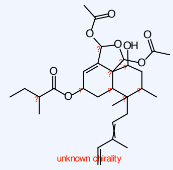	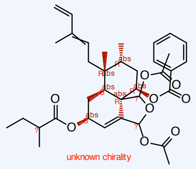	N281	N629	0.84046	0.62	4.6599998	4.04	25.323
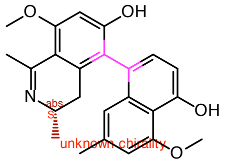	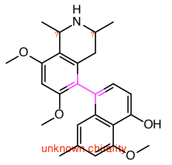	N681	N356	0.83667	5.4000001	1.2760671	4.1239	25.249
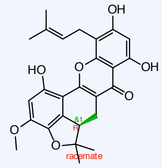	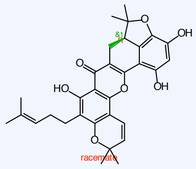	N179	N628	0.82215	0.18000001	4.645926	4.4659	25.11
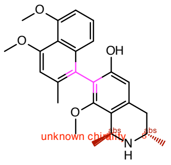	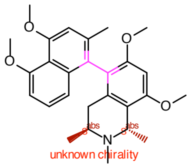	N640	N864	0.83232	4.8000002	9	4.2	25.048
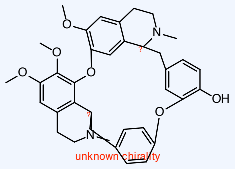	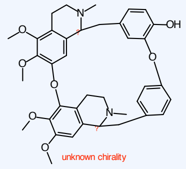	N264	N527	0.88846	0.50926256	3.2876763	2.7784	24.91
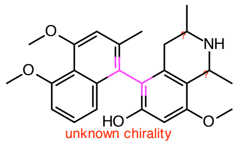	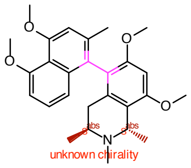	N646	N864	0.83549	4.9079509	9	4.092	24.874
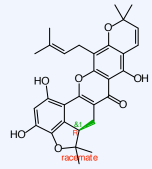	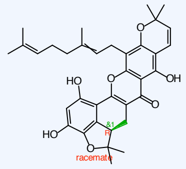	N267	N364	0.96944	0.55000001	1.3099999	0.76	24.87
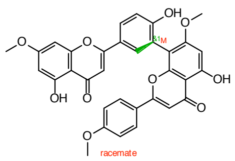	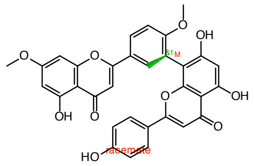	N203	N441	0.92999	0.25999999	2	1.74	24.852
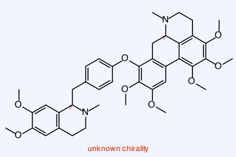	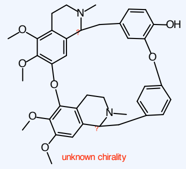	N798	N527	0.83825	7.3045239	3.2876763	4.0168	24.834
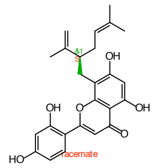	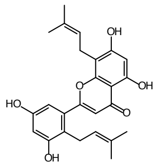	N483	N759	0.83865	2.5999999	6.5999999	4	24.791
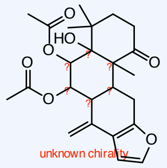	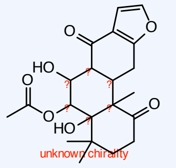	N492	N165	0.8886	2.9000001	0.14	2.76	24.775

Structure 1	Structure 2	ID 1	ID 2	Similarity	Activity 1	Activity 2	Delta Activity	SALI
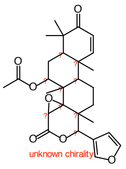	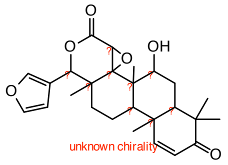	N382	N519	0.93434	1.4920259	3.0999999	1.608	24.491
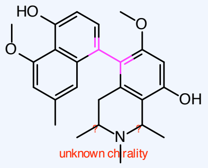	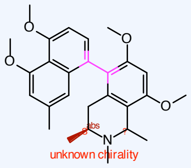	N230	N605	0.83232	0.3435565[illegible]	4.4000001	4.0564	24.192
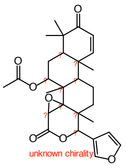	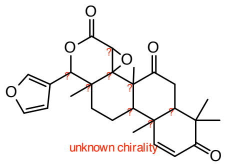	N382	N591	0.89184	1.4920259	4.0999999	2.608	24.112
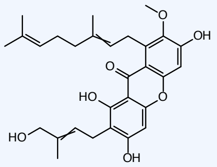	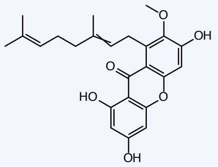	N524	N729	0.88499	3.2350938	6	2.7649	24.04
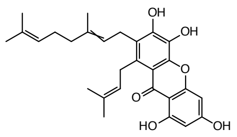	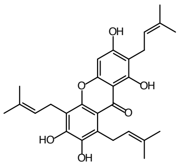	N448	N722	0.83633	2.0665154	6	3.9335	24.033
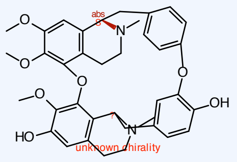	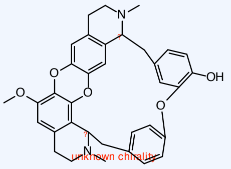	N609	N347	0.85925	4.4819884	1.1	3.382	24.029
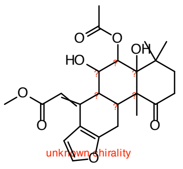	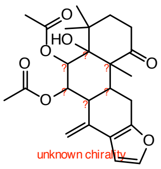	N197	N492	0.88885	0.2399999[illegible]	2.9000001	2.66	23.931
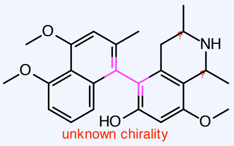	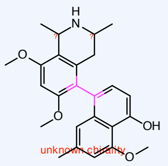	N646	N356	0.84819	4.9079509	1.2760671	3.6319	23.924
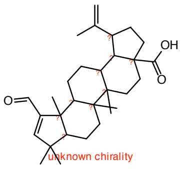	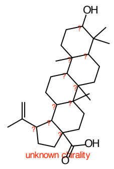	N758	N889	0.85962	6.5999999	9.8999996	3.3	23.507
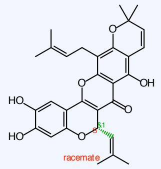	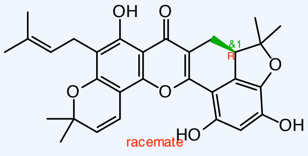	N113	N639	0.79653	0.02	4.8000002	4.78	23.492
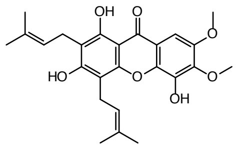	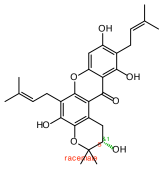	N362	N718	0.7992	1.3	6	4.7	23.406

Structure 1	Structure 2	ID 1	ID 2	Similarity	Activity 1	Activity 2	Delta Activity	SALI
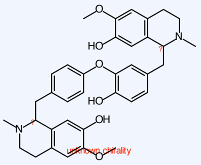	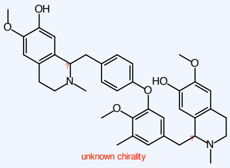	N171	N357	0.95191	0.16	1.28	1.12	23.288
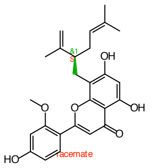	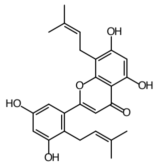	N452	N759	0.80579	2.0999999	6.5999999	4.5	23.17
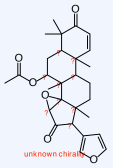	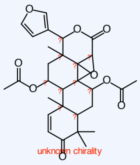	N413	N701	0.82278	1.6503562	5.752852	4.1025	23.149
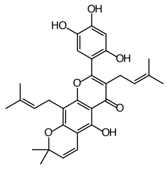	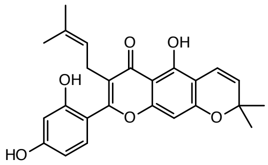	N340	N672	0.81855	1.04	5.1999998	4.16	22.927
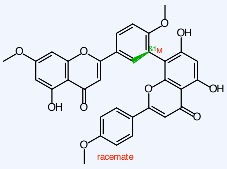	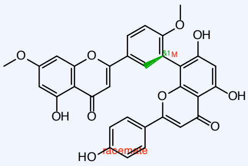	N378	N441	0.97381	1.4	2	0.6	22.907
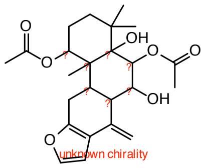	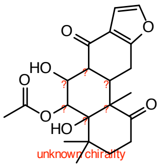	N568	N165	0.83999	3.8	0.14	3.66	22.873
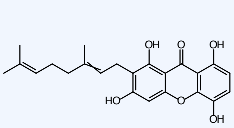	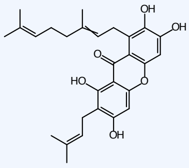	N504	N746	0.85572	3	6.3000002	3.3	22.873
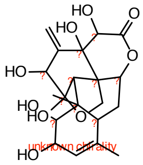	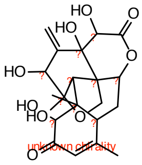	N439	N150	0.91599	2	0.0979433[illegible]	1.9021	22.64
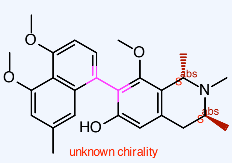	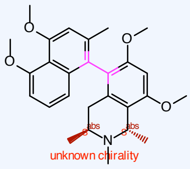	N662	N864	0.82157	5	9	4	22.417
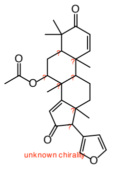	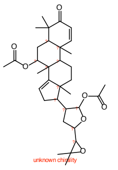	N750	N434	0.80052	6.4000001	1.9340975	4.4659	22.387
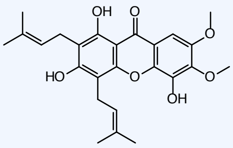	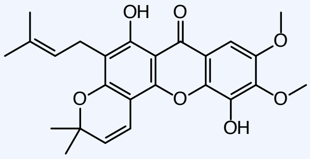	N362	N575	0.8838	1.3	3.9000001	2.6	22.376

Structure 1	Structure 2	ID 1	ID 2	Similarity	Activity 1	Activity 2	Delta Activity	SALI
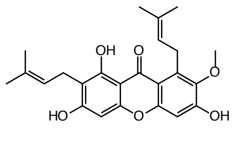	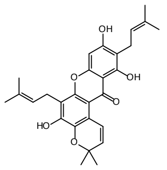	N589	N295	0.84799	4.0999999	0.6999999	3.4	22.367
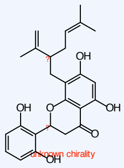	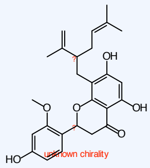	N98	N451	0.90632	0.0108	2.0999999	2.0892	22.303
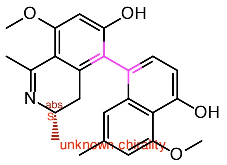	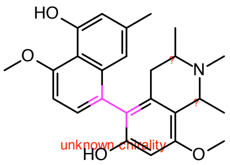	N681	N368	0.81661	5.4000001	1.3202387	4.0798	22.246
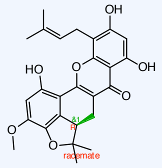	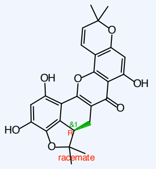	N179	N562	0.84045	0.1800000	3.7	3.52	22.062
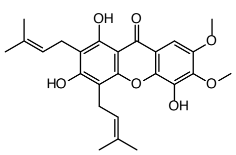	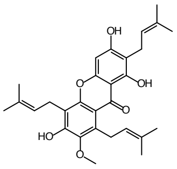	N362	N675	0.82113	1.3	5.2238231	3.9238	21.936
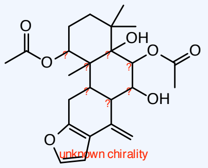	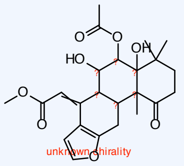	N568	N197	0.8372	3.8	0.2399999	3.56	21.867
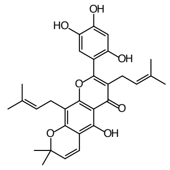	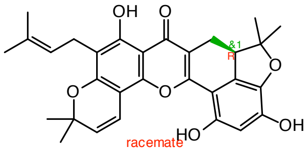	N340	N639	0.8275	1.04	4.8000002	3.76	21.797
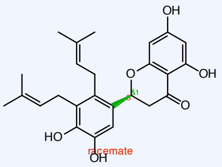	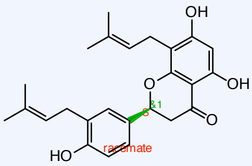	N671	N869	0.82079	5.1999998	9.1000004	3.9	21.763
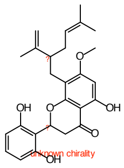	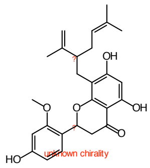	N96	N451	0.90399	0.0106	2.0999999	2.0894	21.762
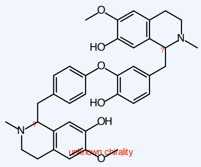	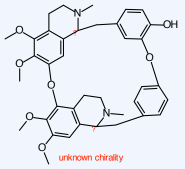	N171	N527	0.85539	0.16	3.2876763	3.1277	21.629
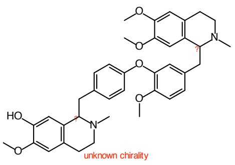	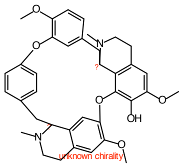	N389	N142	0.93448	1.5	0.0854246	1.4146	21.59

Structure 1	Structure 2	ID 1	ID 2	Similarity	Activity 1	Activity 2	Delta Activity	SALI
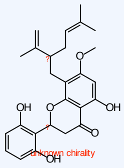	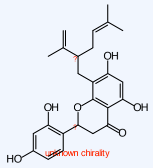	N96	N482	0.87994	0.0106	2.5999999	2.5894	21.567
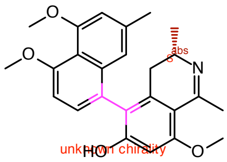	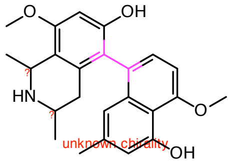	N604	N266	0.81997	4.4000001	0.5286225	3.8714	21.504
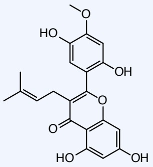	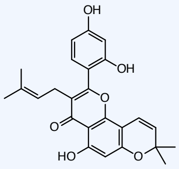	N160	N617	0.79631	0.12	4.5	4.38	21.503
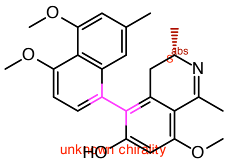	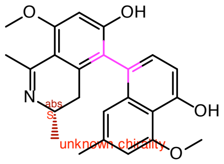	N604	N681	0.95345	4.4000001	5.4000001	1	21.48
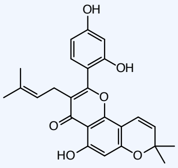	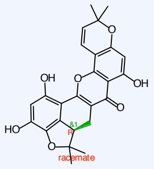	N617	N847	0.81211	4.5	8.5167503	4.0168	21.379
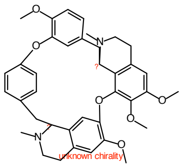	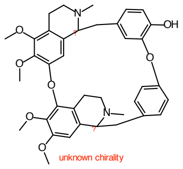	N207	N527	0.85861	0.2649538	3.2876763	3.0227	21.379
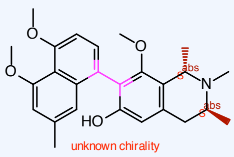	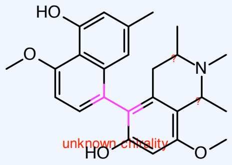	N662	N368	0.82727	5	1.3202387	3.6798	21.304
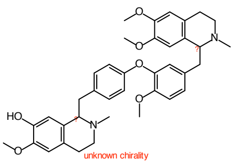	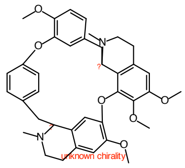	N389	N207	0.94195	1.5	0.2649538	1.235	21.276
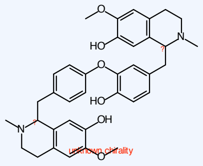	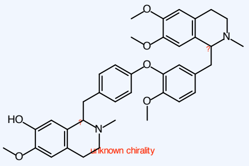	N171	N389	0.93664	0.16	1.5	1.34	21.15
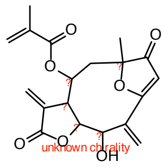	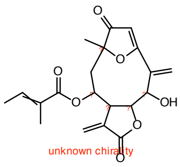	N602	N692	0.94314	4.4000001	5.5999999	1.2	21.104
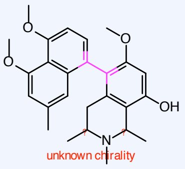	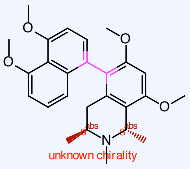	N235	N616	0.80354	0.3558477	4.5	4.1442	21.094

Structure 1	Structure 2	ID 1	ID 2	Similarity	Activity 1	Activity 2	Delta Activity	SALI
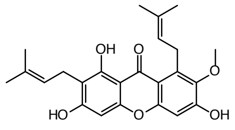	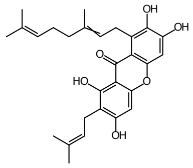	N589	N746	0.89541	4.0999999	6.3000002	2.2	21.035
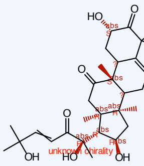	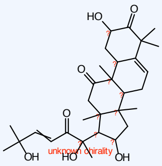	N580	N811	0.82377	4	7.6999998	3.7	20.995
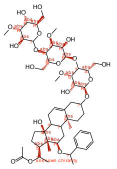	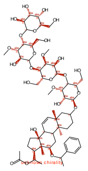	N799	N883	0.89483	7.4000001	9.6000004	2.2	20.919
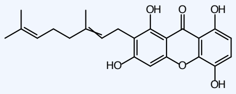	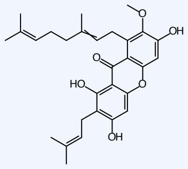	N504	N743	0.84309	3	6.2685881	3.2686	20.83
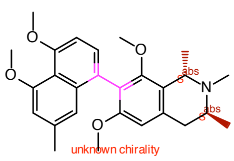	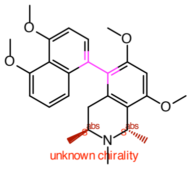	N442	N616	0.87994	2	4.5	2.5	20.823
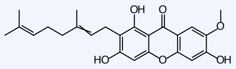	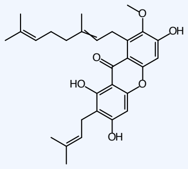	N555	N743	0.87425	3.6544397	6.2685881	2.6141	20.788
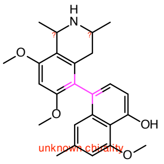	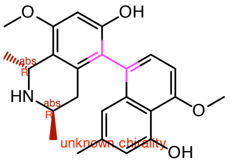	N356	N665	0.81661	1.2760671	5.0829086	3.8068	20.758
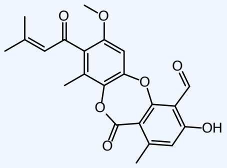	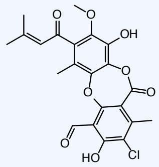	N520	N792	0.80214	3.0999999	7.1999998	4.1	20.721
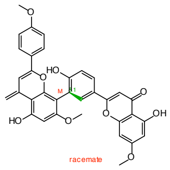	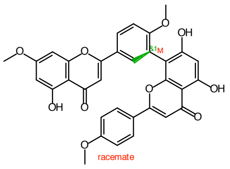	N206	N378	0.94485	0.2599999	1.4	1.14	20.672
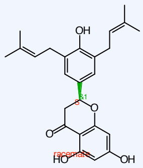	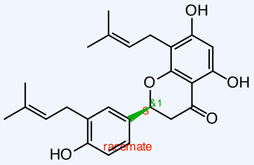	N737	N869	0.85465	6.0999999	9.1000004	3	20.64
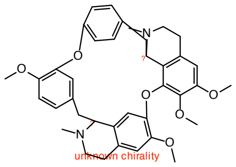	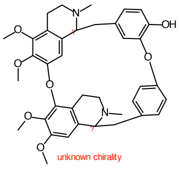	N241	N527	0.85861	0.3709354	3.2876763	2.9167	20.629

Structure 1	Structure 2	ID 1	ID 2	Similarity	Activity 1	Activity 2	Delta Activity	SALI
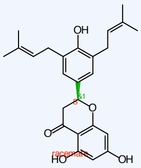	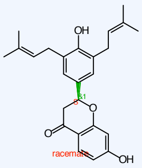	N737	N808	0.92232	6.0999999	7.6999998	1.6	20.597
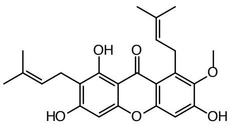	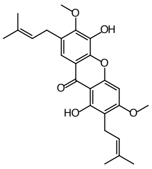	N589	N794	0.84655	4.0999999	7.25	3.15	20.527
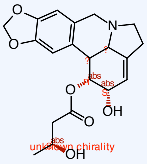	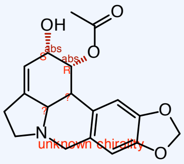	N410	N515	0.9302	1.6068573	3.0363095	1.4295	20.481
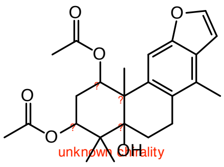	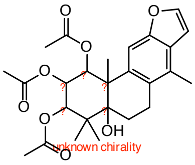	N584	N755	0.87793	4	6.5	2.5	20.48
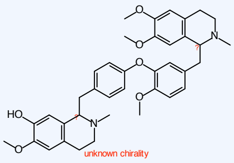	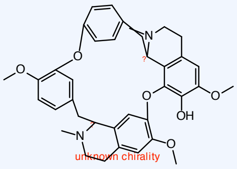	N389	N176	0.93448	1.5	0.1718350[illegible]	1.3282	20.271
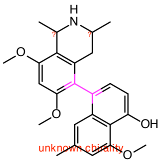	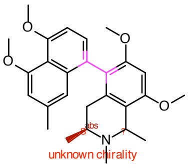	N356	N605	0.84495	1.2760671	4.4000001	3.1239	20.148
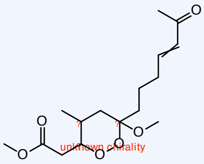	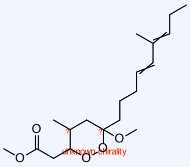	N660	N770	0.91037	5	6.8000002	1.8	20.082
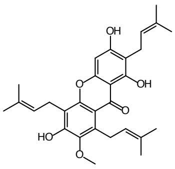	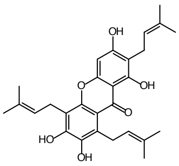	N675	N722	0.96116	5.2238231	6	0.77618	19.985
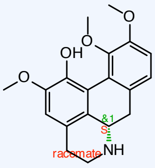	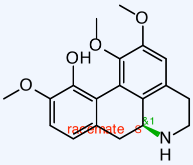	N516	N578	0.95384	3.0799999	4	0.92	19.929
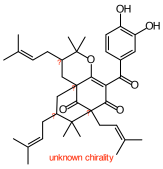	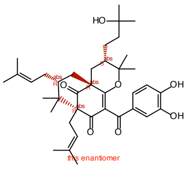	N457	N655	0.85792	2.2	5	2.8	19.707
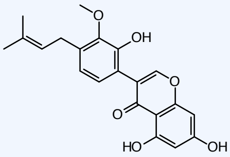	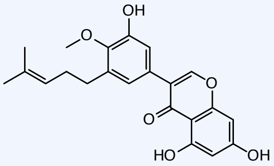	N748	N858	0.87809	6.3000002	8.6999998	2.4	19.687

Structure 1	Structure 2	ID 1	ID 2	Similarity	Activity 1	Activity 2	Delta Activity	SALI
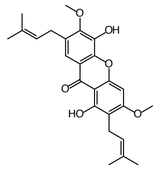	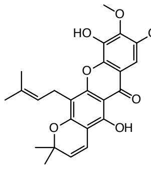	N794	N547	0.80895	7.25	3.5	3.75	19.629
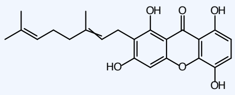	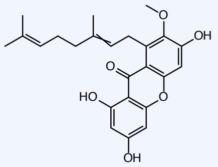	N504	N729	0.8466	3	6	3	19.557
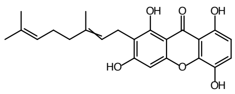	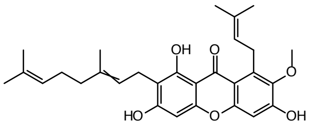	N504	N720	0.84614	3	6	3	19.498
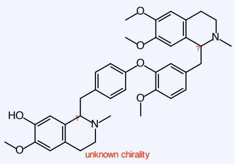	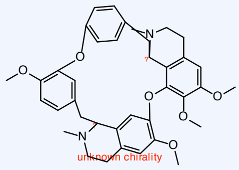	N389	N241	0.94195	1.5	0.3709354	1.1291	19.45
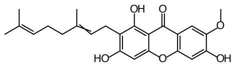	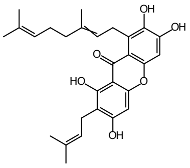	N555	N746	0.86307	3.6544397	6.3000002	2.6456	19.321
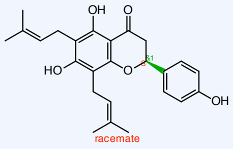	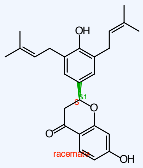	N573	N808	0.8031	3.9000001	7.6999998	3.8	19.299
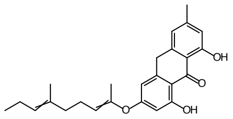	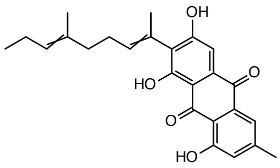	N416	N678	0.80986	1.6799999	5.3400002	3.66	19.249
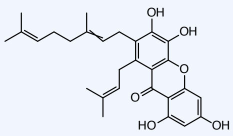	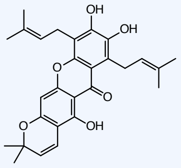	N448	N719	0.79546	2.0665154	6	3.9335	19.231
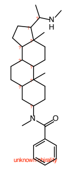	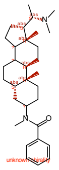	N760	N908	0.80736	6.5999999	10.3	3.7	19.207
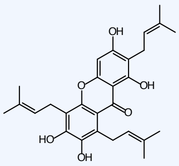	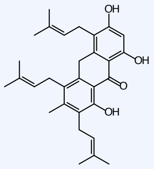	N722	N563	0.87993	6	3.7	2.3	19.155
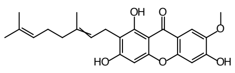	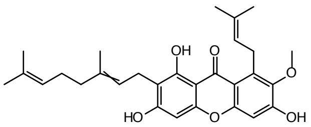	N555	N720	0.87716	3.6544397	6	2.3456	19.095

Structure 1	Structure 2	ID 1	ID 2	Similarity	Activity 1	Activity 2	Delta Activity	SALI
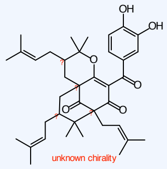	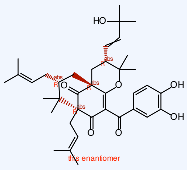	N457	N654	0.85336	2.2	5	2.8	19.094
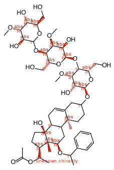	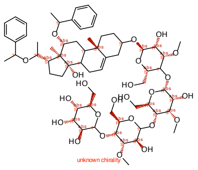	N799	N698	0.91096	7.4000001	5.6999998	1.7	19.093
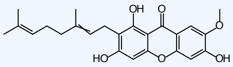	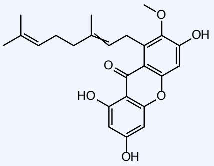	N555	N729	0.87695	3.6544397	6	2.3456	19.061
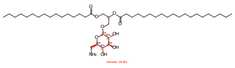	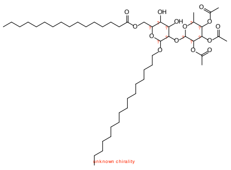	N622	N836	0.81087	4.5	8.1000004	3.6	19.035
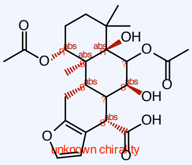	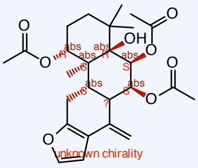	N776	N537	0.80815	7	3.4000001	3.6	18.765
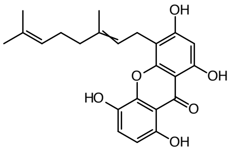	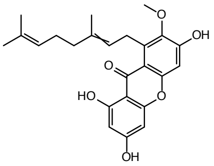	N508	N729	0.83999	3	6	3	18.749
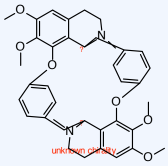	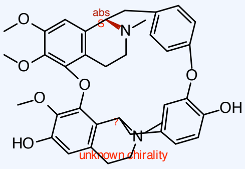	N391	N609	0.84036	1.5	4.4819884	2.982	18.68
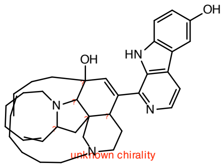	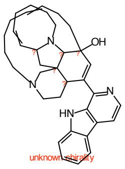	N300	N101	0.96077	0.7436785[illegible]	0.0125	0.73118	18.638
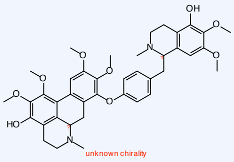	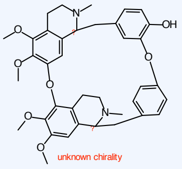	N229	N527	0.84167	0.3405832[illegible]	3.2876763	2.9471	18.613
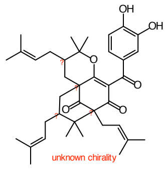	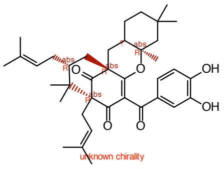	N457	N652	0.84908	2.2	5	2.8	18.553
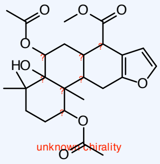	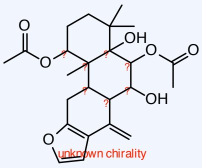	N754	N568	0.85405	6.5	3.8	2.7	18.499

Structure 1	Structure 2	ID 1	ID 2	Similarity	Activity 1	Activity 2	Delta Activity	SALI
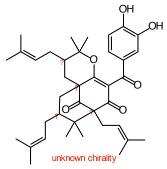	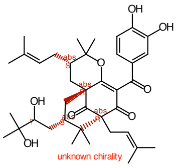	N457	N656	0.84849	2.2	5	2.8	18.48
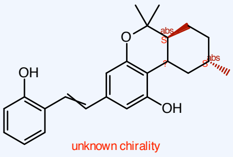	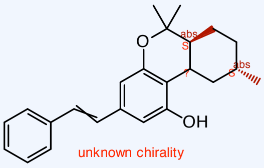	N225	N422	0.92394	0.32923862	1.7217735	1.3925	18.308
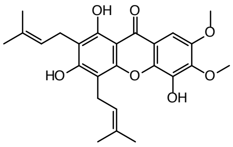	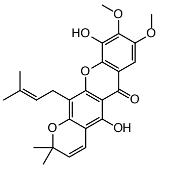	N362	N547	0.87793	1.3	3.5	2.2	18.022
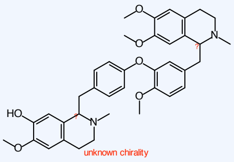	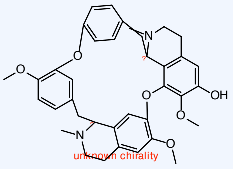	N389	N191	0.92856	1.5	0.2135617[illegible]	1.2864	18.008
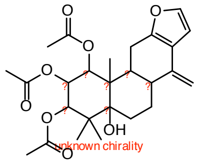	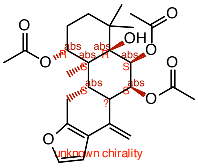	N184	N537	0.82184	0.2	3.4000001	3.2	17.962
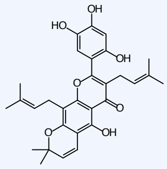	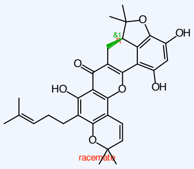	N340	N628	0.79878	1.04	4.645926	3.6059	17.92
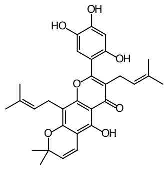	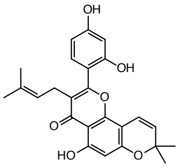	N340	N617	0.80675	1.04	4.5	3.46	17.904
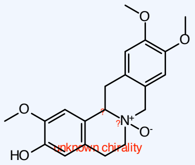	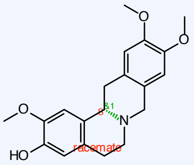	N595	N408	0.85446	4.1999998	1.6	2.6	17.865
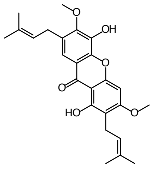	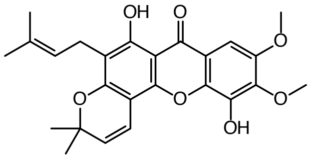	N794	N575	0.81211	7.25	3.9000001	3.35	17.83
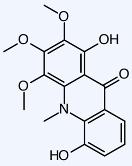	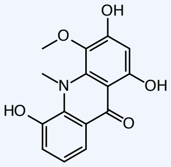	N738	N861	0.84278	6.0999999	8.8999996	2.8	17.81
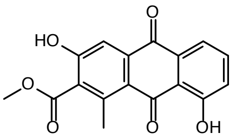	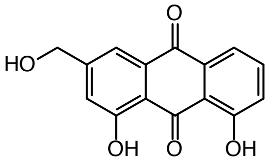	N583	N14	0.829	4	7.0308681	3.0309	17.725

Structure 1	Structure 2	ID 1	ID 2	Similarity	Activity 1	Activity 2	Delta Activity	SALI
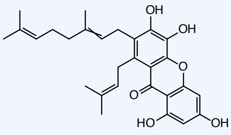	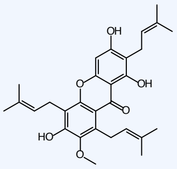	N448	N675	0.82182	2.0665154	5.2238231	3.1573	17.72
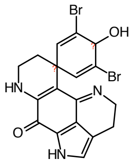	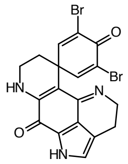	N163	N437	0.89438	0.13	2	1.87	17.704
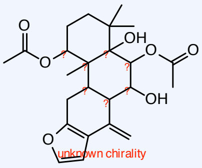	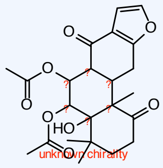	N568	N328	0.84181	3.8	1	2.8	17.7
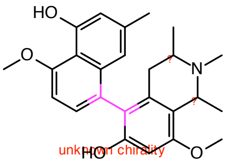	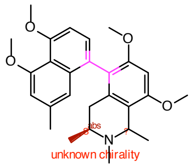	N368	N605	0.82597	1.3202387	4.4000001	3.0798	17.697
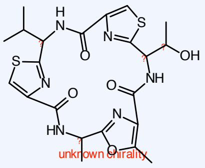	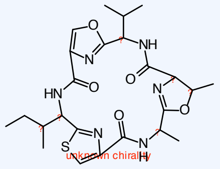	N694	N465	0.81305	5.5999999	2.3	3.3	17.652
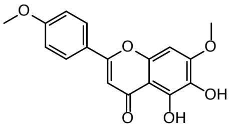	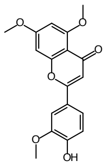	N867	N959	0.80929	9	12.366135	3.3661	17.651
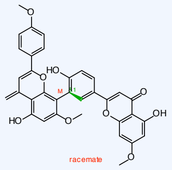	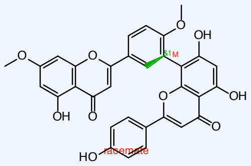	N206	N441	0.90073	0.25999999	2	1.74	17.527
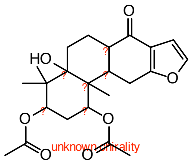	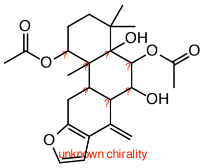	N204	N568	0.79793	0.25999999	3.8	3.54	17.518
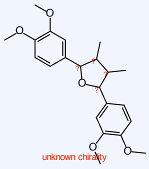	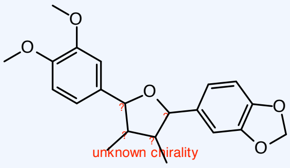	N842	N904	0.89688	8.3000002	10.100662	1.8007	17.461
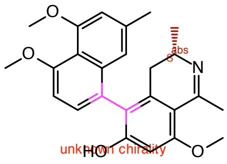	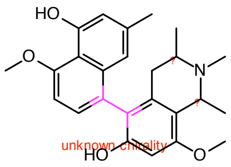	N604	N368	0.82204	4.4000001	1.3202387	3.0798	17.306
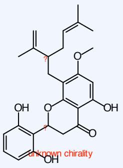	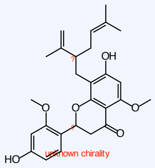	N96	N469	0.86132	0.0106	2.4000001	2.3894	17.229

Structure 1	Structure 2	ID 1	ID 2	Similarity	Activity 1	Activity 2	Delta Activity	SALI
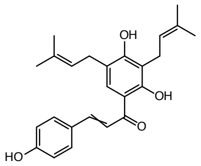	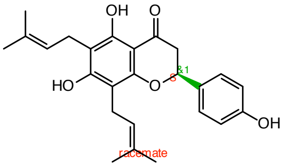	N307	N573	0.81997	0.80000001	3.9000001	3.1	17.219
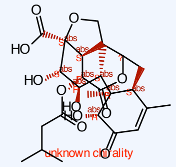	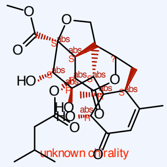	N277	N354	0.96512	0.60000002	1.2	0.6	17.202
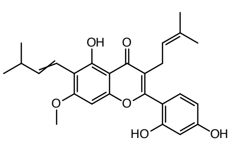	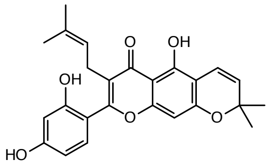	N773	N672	0.90055	6.9000001	5.1999998	1.7	17.095
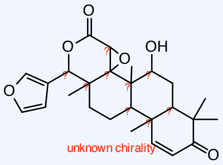	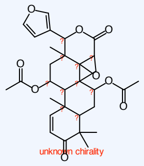	N519	N701	0.84449	3.0999999	5.752852	2.6529	17.059
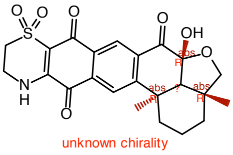	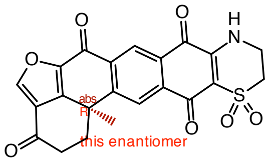	N7	N572	0.79944	0.5	3.9000001	3.4	16.952
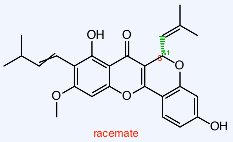	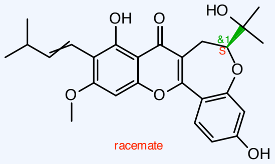	N890	N813	0.86721	9.8999996	7.6999998	2.2	16.567
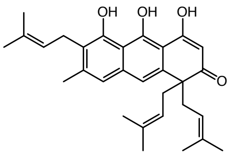	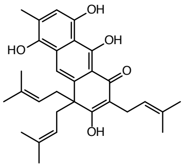	N658	N428	0.80663	5	1.8	3.2	16.549
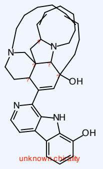	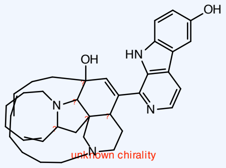	N97	N300	0.95564	0.01062397	0.74367851	0.73305	16.526
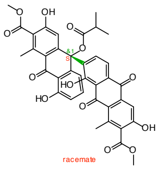	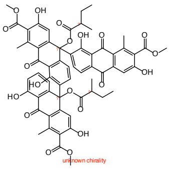	N820	N661	0.82347	7.9000001	5	2.9	16.428
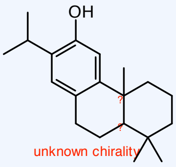	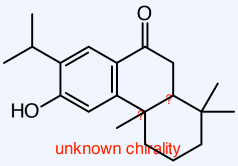	N533	N374	0.88278	3.3164415	1.4	1.9164	16.349
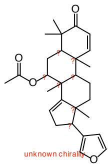	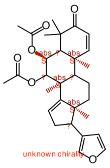	N19	N596	0.81636	1.21	4.1999998	2.99	16.282

Structure 1	Structure 2	ID 1	ID 2	Similarity	Activity 1	Activity 2	Delta Activity	SALI
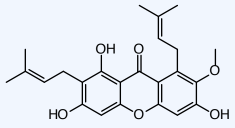	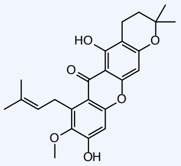	N589	N732	0.88322	4.0999999	6	1.9	16.27
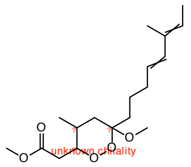	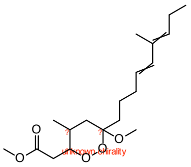	N727	N770	0.95082	6	6.8000002	0.8	16.265
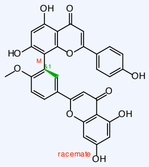	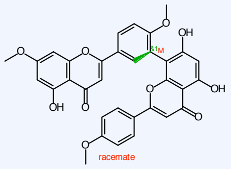	N219	N378	0.93225	0.3000000[illegible]	1.4	1.1	16.236
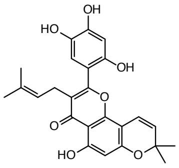	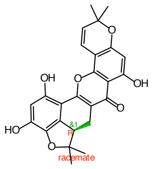	N749	N562	0.83272	6.4000001	3.7	2.7	16.141
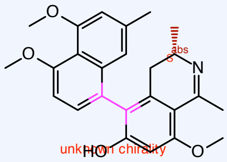	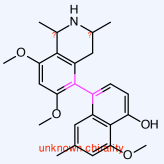	N604	N356	0.80547	4.4000001	1.2760671	3.1239	16.059
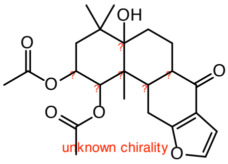	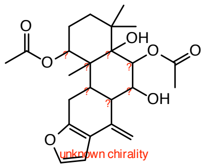	N308	N568	0.81103	0.8000000[illegible]	3.8	3	15.875
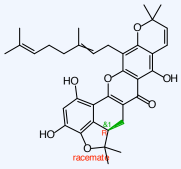	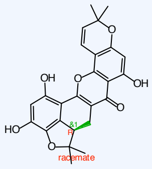	N364	N562	0.84883	1.3099999	3.7	2.39	15.81
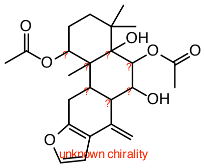	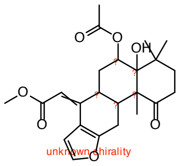	N568	N289	0.80031	3.8	0.6499999[illegible]	3.15	15.774
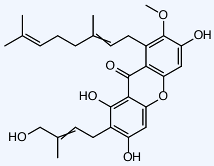	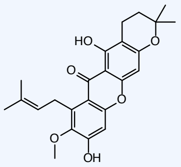	N524	N732	0.8234	3.2350938	6	2.7649	15.656
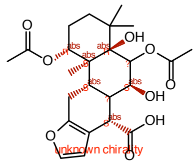	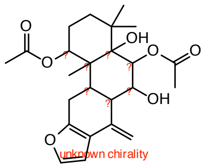	N776	N568	0.79546	7	3.8	3.2	15.645
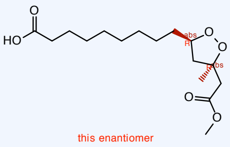	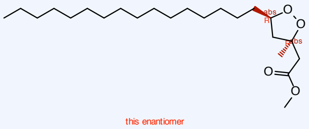	N217	N498	0.82694	0.3000000[illegible]	3	2.7	15.602

Structure 1	Structure 2	ID 1	ID 2	Similarity	Activity 1	Activity 2	Delta Activity	SALI
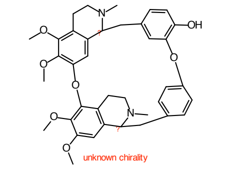	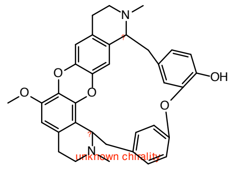	N527	N347	0.85975	3.2876763	1.1	2.1877	15.599
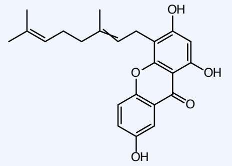	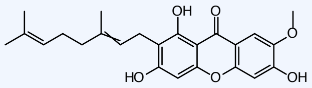	N360	N555	0.8483	1.3	3.6544397	2.3544	15.521
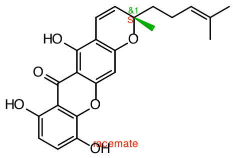	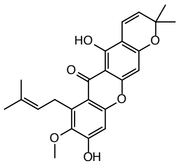	N509	N731	0.80648	3	6	3	15.503
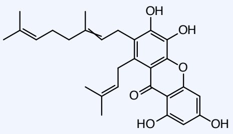	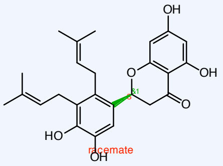	N448	N671	0.79777	2.0665154	5.1999998	3.1335	15.495
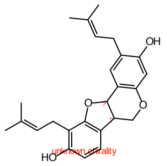	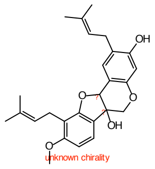	N756	N865	0.83844	6.5	9	2.5	15.474
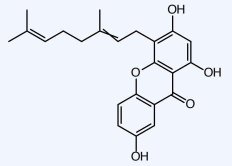	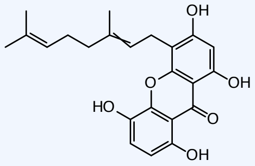	N360	N508	0.89009	1.3	3	1.7	15.467
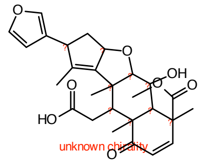	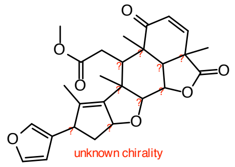	N404	N565	0.86038	1.5891421	3.7297204	2.1406	15.331
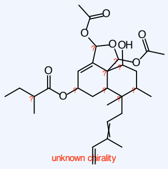	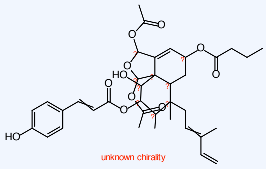	N281	N552	0.80223	0.62	3.5999999	2.98	15.068
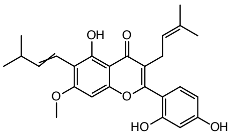	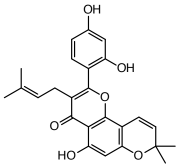	N773	N617	0.8403	6.9000001	4.5	2.4	15.029
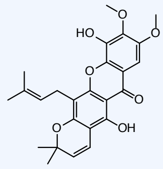	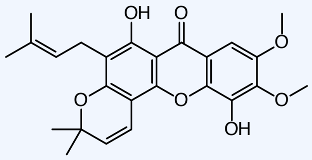	N547	N575	0.97319	3.5	3.9000001	0.4	14.918
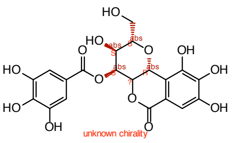	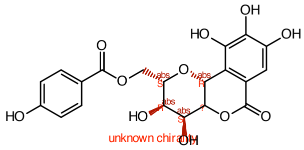	N844	N941	0.80484	8.3999996	11.3	2.9	14.86

Structure 1	Structure 2	ID 1	ID 2	Similarity	Activity 1	Activity 2	Delta Activity	SALI
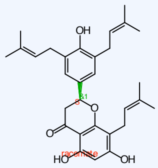	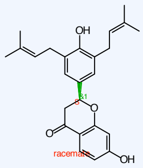	N676	N808	0.83815	5.3000002	7.6999998	2.4	14.828
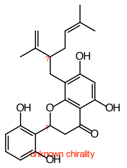	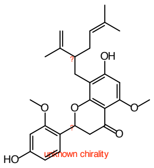	N98	N469	0.83885	0.0108	2.4000001	2.3892	14.826
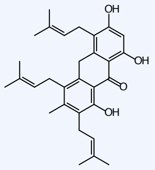	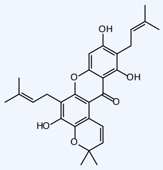	N563	N295	0.79653	3.7	0.6999999	3	14.744
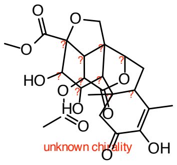	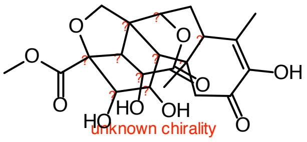	N116	N337	0.93165	0.0228946	1.0286819	1.0058	14.715
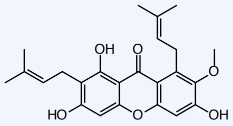	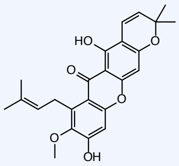	N589	N731	0.87063	4.0999999	6	1.9	14.687
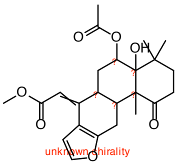	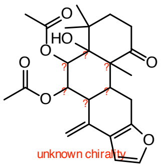	N289	N492	0.84495	0.6499999	2.9000001	2.25	14.512
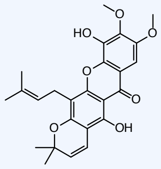	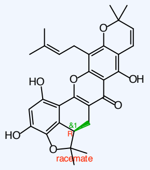	N547	N267	0.79603	3.5	0.5500000	2.95	14.463
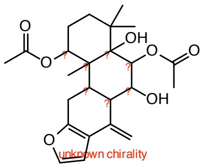	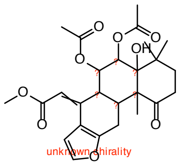	N568	N327	0.80607	3.8	1	2.8	14.439
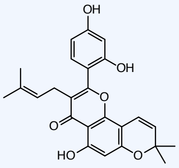	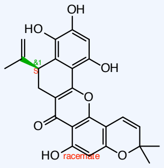	N617	N772	0.83096	4.5	6.9000001	2.4	14.198
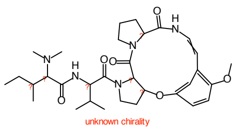	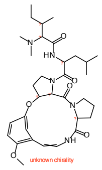	N712	N751	0.96438	5.9000001	6.4000001	0.5	14.035
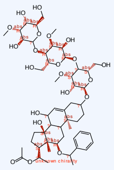	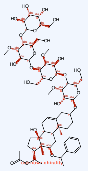	N757	N802	0.92865	6.5	7.5	1	14.016

Structure 1	Structure 2	ID 1	ID 2	Similarity	Activity 1	Activity 2	Delta Activity	SALI
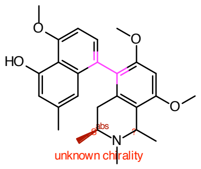	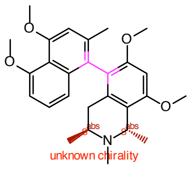	N742	N864	0.79944	6.1999998	9	2.8	13.961
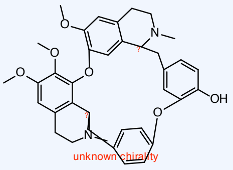	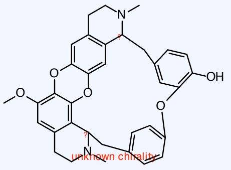	N264	N347	0.95755	0.5092625(	1.1	0.59074	13.916
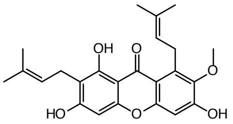	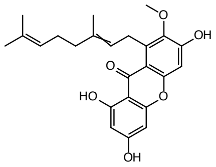	N589	N729	0.86107	4.0999999	6	1.9	13.676
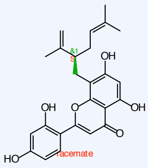	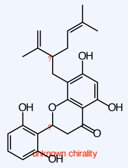	N483	N98	0.80997	2.5999999	0.0108	2.5892	13.625
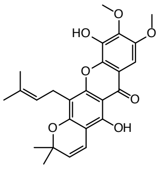	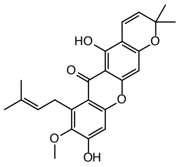	N547	N731	0.81639	3.5	6	2.5	13.616
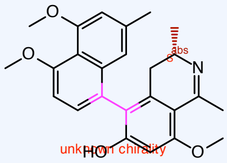	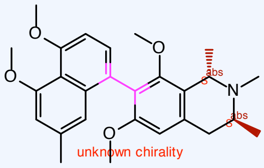	N604	N442	0.82278	4.4000001	2	2.4	13.543
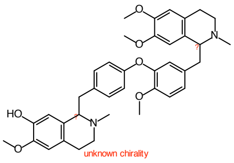	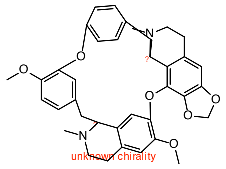	N389	N194	0.9061	1.5	0.2307537:	1.2692	13.516
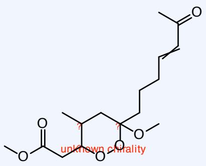	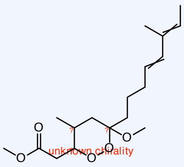	N660	N727	0.92547	5	6	1	13.417
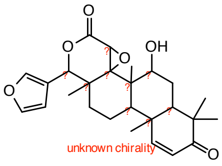	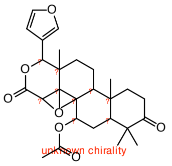	N519	N682	0.82592	3.0999999	5.4273667	2.3274	13.37
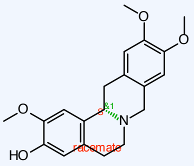	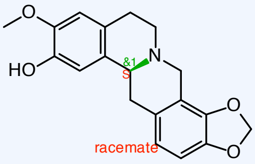	N408	N598	0.79853	1.6	4.29	2.69	13.352
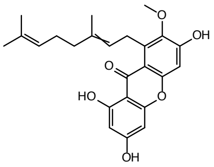	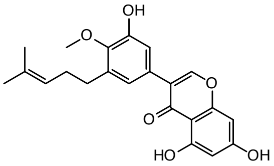	N729	N858	0.79719	6	8.6999998	2.7	13.313

Structure 1	Structure 2	ID 1	ID 2	Similarity	Activity 1	Activity 2	Delta Activity	SALI
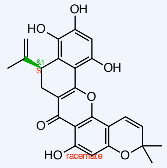	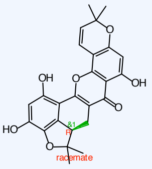	N772	N847	0.87793	6.9000001	8.5167503	1.6168	13.244
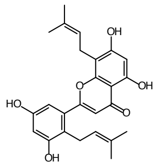	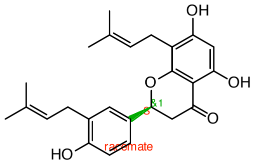	N759	N869	0.81103	6.5999999	9.1000004	2.5	13.229
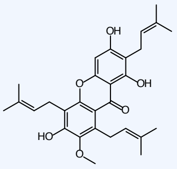	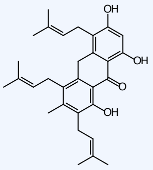	N675	N563	0.88464	5.2238231	3.7	1.5238	13.209
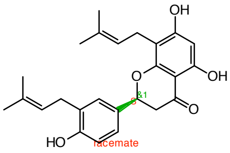	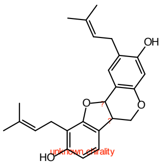	N869	N756	0.8031	9.1000004	6.5	2.6	13.205
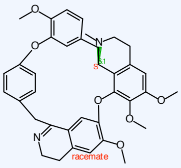	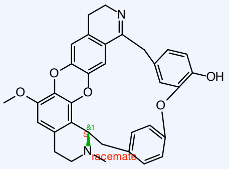	N175	N412	0.88897	0.17	1.63	1.46	13.15
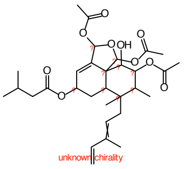	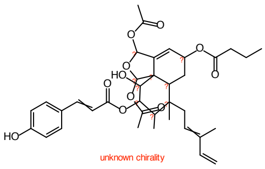	N673	N552	0.8776	5.1999998	3.5999999	1.6	13.072
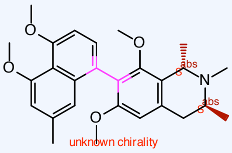	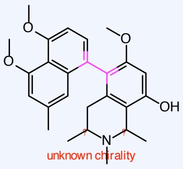	N442	N235	0.87394	2	0.3558477[illegible]	1.6442	13.043
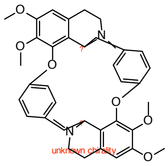	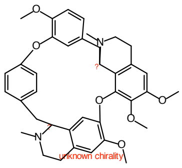	N391	N207	0.90407	1.5	0.2649538[illegible]	1.235	12.875
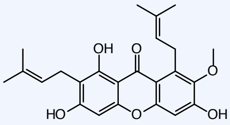	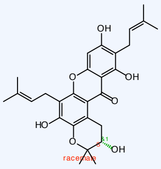	N589	N718	0.85242	4.0999999	6	1.9	12.874
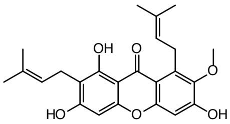	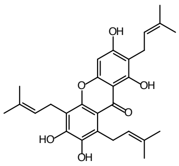	N589	N722	0.85102	4.0999999	6	1.9	12.754
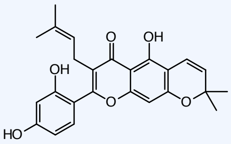	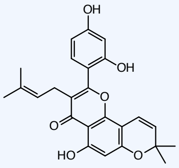	N672	N617	0.94488	5.1999998	4.5	0.7	12.701

Structure 1	Structure 2	ID 1	ID 2	Similarity	Activity 1	Activity 2	Delta Activity	SALI
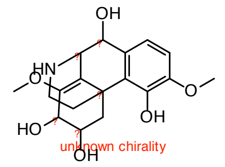	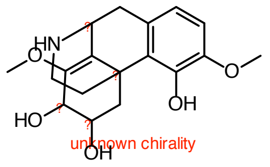	N521	N620	0.88969	3.0999999	4.5	1.4	12.692
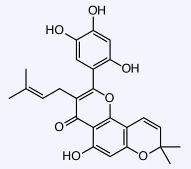	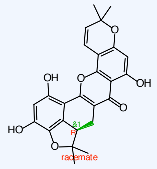	N749	N847	0.83272	6.4000001	8.5167503	2.1168	12.654
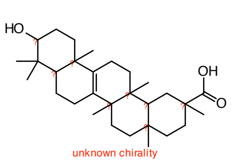	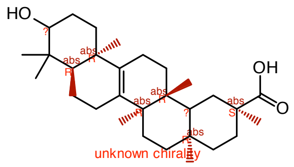	N443	N601	0.81737	2	4.3000002	2.3	12.594
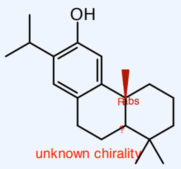	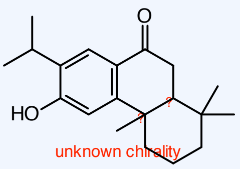	N541	N374	0.83358	3.4909911	1.4	2.091	12.564
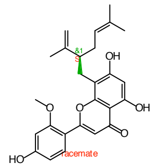	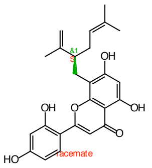	N452	N483	0.95983	2.0999999	2.5999999	0.5	12.448
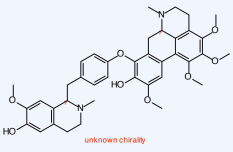	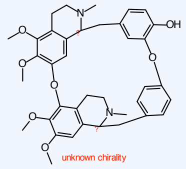	N366	N527	0.84125	1.3158381	3.2876763	1.9718	12.421
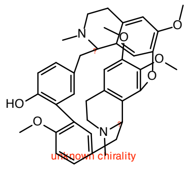	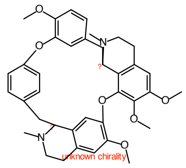	N350	N207	0.92842	1.14	0.2649538	0.87505	12.224
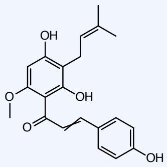	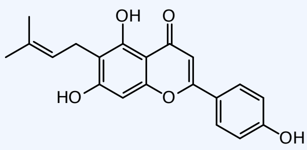	N838	N766	0.87695	8.1999998	6.6999998	1.5	12.19
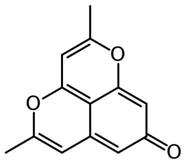	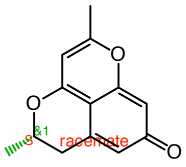	N633	N464	0.80172	4.6999998	2.3	2.4	12.104
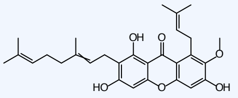	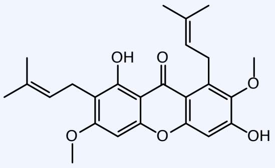	N720	N784	0.91165	6	7.0673671	1.0674	12.081
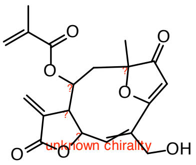	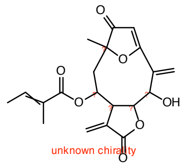	N529	N692	0.80961	3.3	5.5999999	2.3	12.08

Structure 1	Structure 2	ID 1	ID 2	Similarity	Activity 1	Activity 2	Delta Activity	SALI
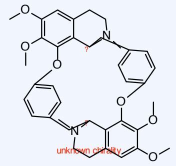	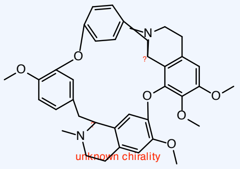	N391	N241	0.90615	1.5	0.3709354	1.1291	12.03
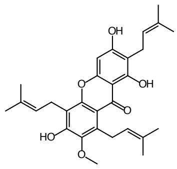	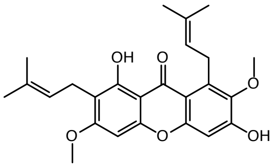	N675	N784	0.8461	5.2238231	7.0673671	1.8435	11.979
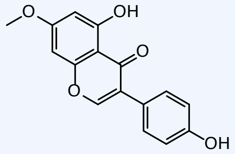	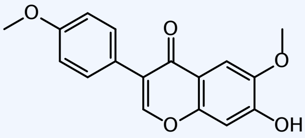	N848	N935	0.80128	8.5500002	10.91	2.36	11.876
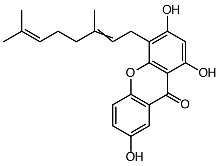	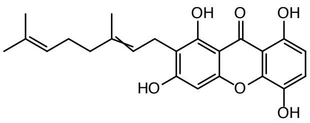	N360	N504	0.85685	1.3	3	1.7	11.876
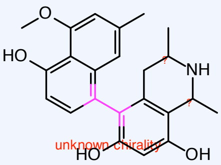	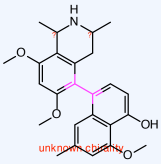	N136	N356	0.8973	0.0632496	1.2760671	1.2128	11.809
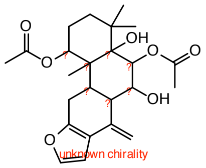	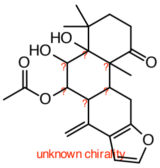	N568	N705	0.83001	3.8	5.8000002	2	11.766
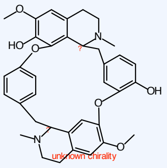	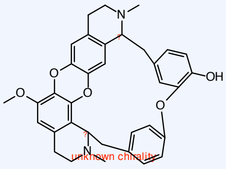	N234	N347	0.93641	0.3529999	1.1	0.747	11.747
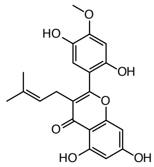	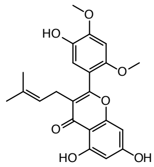	N160	N291	0.95401	0.12	0.6600000	0.54	11.741
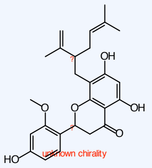	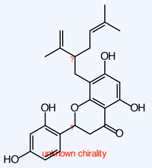	N451	N482	0.95739	2.0999999	2.5999999	0.5	11.736
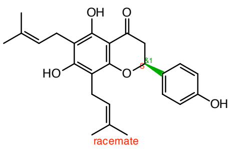	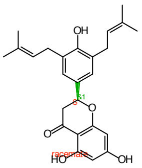	N573	N737	0.81211	3.9000001	6.0999999	2.2	11.709
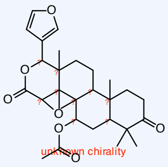	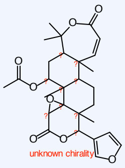	N682	N581	0.87714	5.4273667	4	1.4274	11.618

Structure 1	Structure 2	ID 1	ID 2	Similarity	Activity 1	Activity 2	Delta Activity	SALI
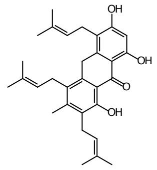	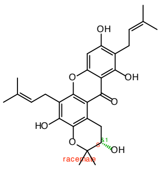	N563	N718	0.8016	3.7	6	2.3	11.593
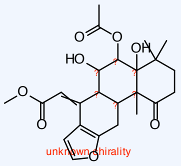	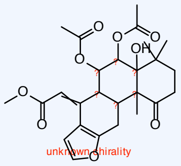	N197	N327	0.93434	0.2399999	1	0.76	11.575
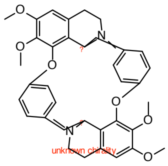	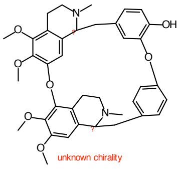	N391	N527	0.84544	1.5	3.2876763	1.7877	11.566
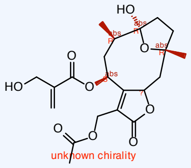	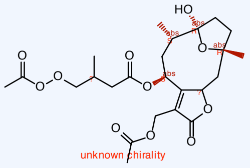	N557	N685	0.84345	3.7	5.5	1.8	11.498
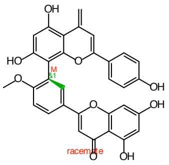	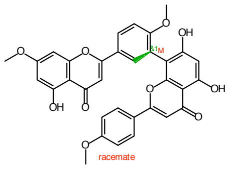	N220	N378	0.90286	0.3000000	1.4	1.1	11.323
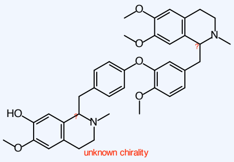	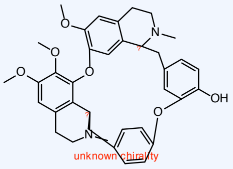	N389	N264	0.91233	1.5	0.5092625	0.99074	11.301
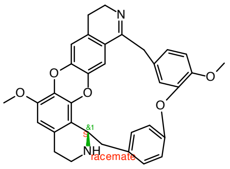	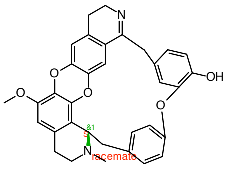	N269	N412	0.90523	0.5616411	1.63	1.0684	11.273
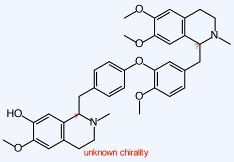	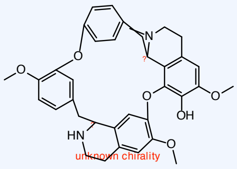	N389	N154	0.87581	1.5	0.1059363	1.3941	11.225
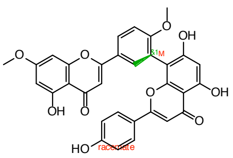	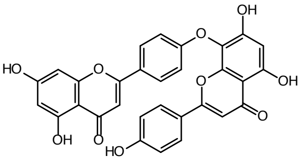	N441	N255	0.86427	2	0.4799999	1.52	11.199
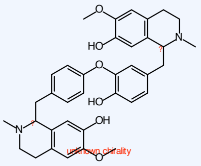	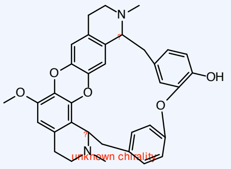	N171	N347	0.91543	0.16	1.1	0.94	11.115
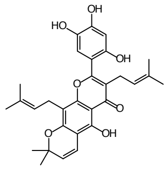	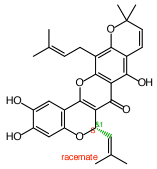	N340	N113	0.90817	1.04	0.02	1.02	11.107

Structure 1	Structure 2	ID 1	ID 2	Similarity	Activity 1	Activity 2	Delta Activity	SALI
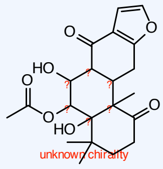	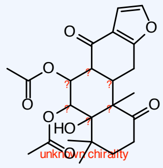	N165	N328	0.92232	0.14	1	0.86	11.071
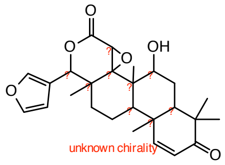	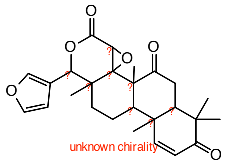	N519	N591	0.90963	3.0999999	4.0999999	1	11.066
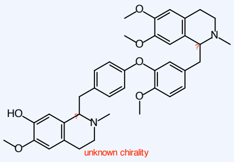	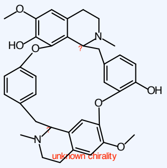	N389	N234	0.8954	1.5	0.3529999[illegible]	1.147	10.965
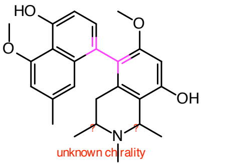	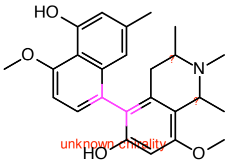	N230	N368	0.91076	0.3435565[illegible]	1.3202387	0.97668	10.944
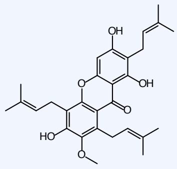	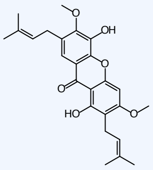	N675	N794	0.81323	5.2238231	7.25	2.0262	10.849
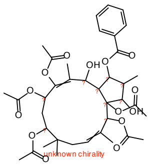	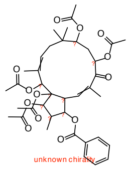	N717	N643	0.8979	6	4.9000001	1.1	10.774
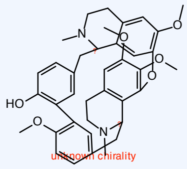	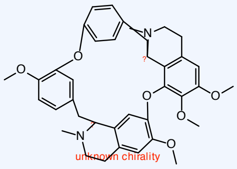	N350	N241	0.92842	1.14	0.3709354[illegible]	0.76906	10.744
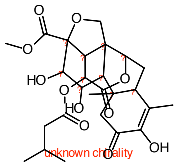	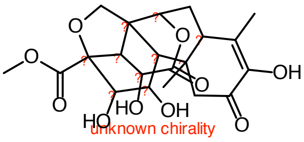	N115	N337	0.90599	0.0210509[illegible]	1.0286819	1.0076	10.718
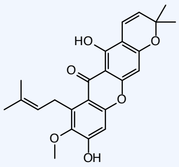	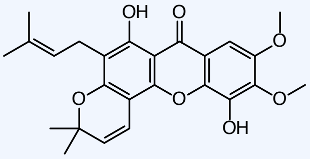	N731	N575	0.80354	6	3.9000001	2.1	10.689
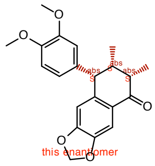	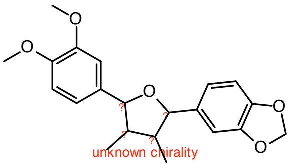	N826	N904	0.80316	8	10.100662	2.1007	10.672
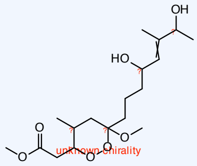	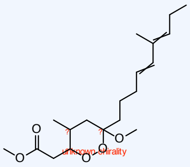	N659	N770	0.83096	5	6.8000002	1.8	10.649

Structure 1	Structure 2	ID 1	ID 2	Similarity	Activity 1	Activity 2	Delta Activity	SALI
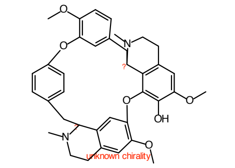	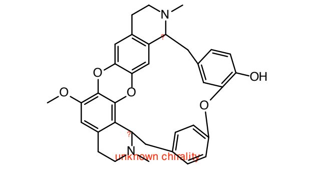	N142	N347	0.90462	0.08542469	1.1	1.0146	10.637
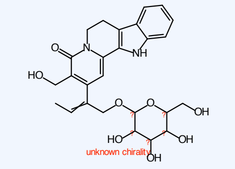	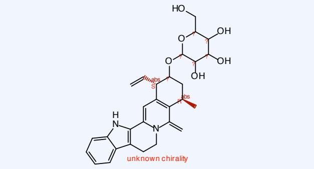	N885	N823	0.83868	9.6999998	8	1.7	10.538
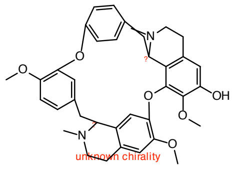	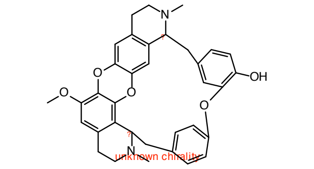	N191	N347	0.9154	0.21356173	1.1	0.88644	10.478
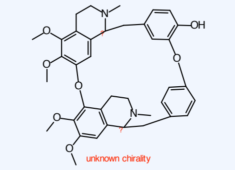	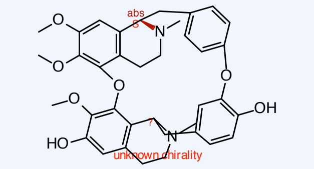	N527	N609	0.88569	3.2876763	4.4819884	1.1943	10.448
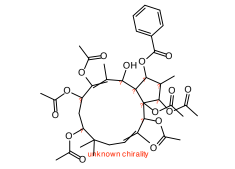	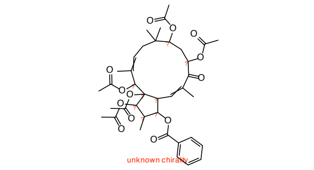	N703	N643	0.91383	5.8000002	4.9000001	0.9	10.444
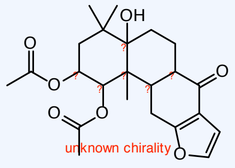	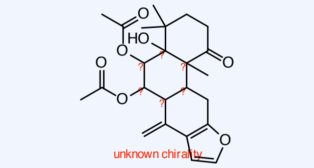	N308	N492	0.79793	0.80000001	2.9000001	2.1	10.392
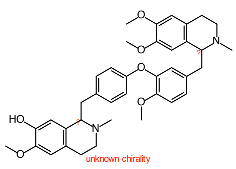	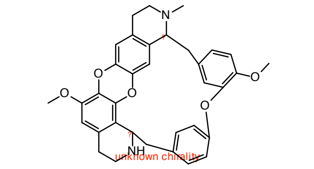	N389	N177	0.87153	1.5	0.17239699	1.3276	10.334
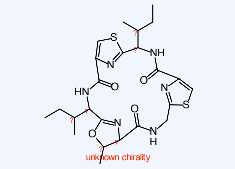	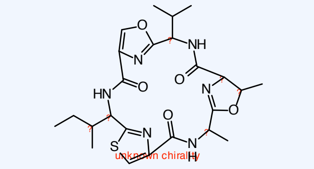	N296	N465	0.84495	0.69999999	2.3	1.6	10.319
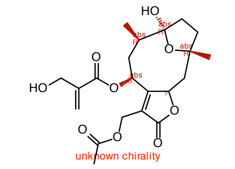	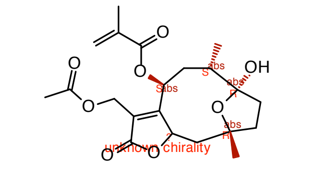	N557	N615	0.92232	3.7	4.5	0.8	10.298
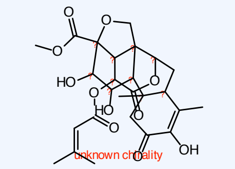	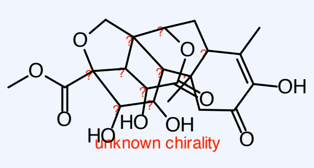	N90	N337	0.90064	0.0057634[illegible]	1.0286819	1.0229	10.295
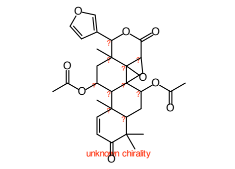	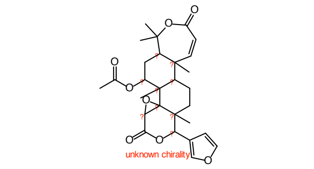	N701	N581	0.8297	5.752852	4	1.7529	10.293

Structure 1	Structure 2	ID 1	ID 2	Similarity	Activity 1	Activity 2	Delta Activity	SALI
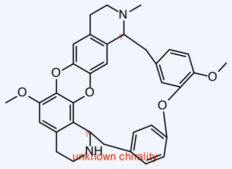	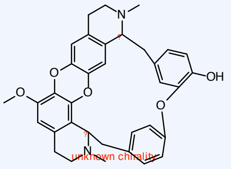	N177	N347	0.90982	0.1723969	1.1	0.9276	10.286
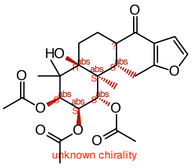	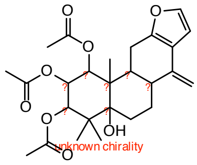	N438	N184	0.82475	2	0.2	1.8	10.271
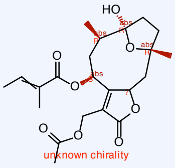	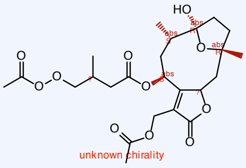	N571	N685	0.84345	3.9000001	5.5	1.6	10.22
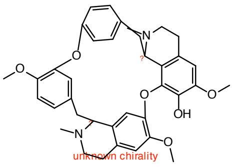	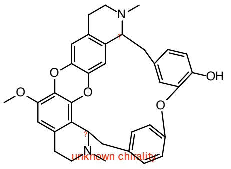	N176	N347	0.90896	0.1718350	1.1	0.92816	10.195
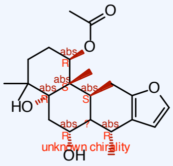	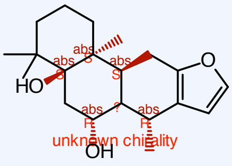	N244	N450	0.83321	0.4000000	2.0999999	1.7	10.193
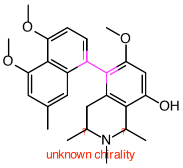	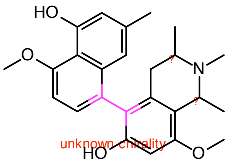	N235	N368	0.90529	0.3558477	1.3202387	0.96439	10.182
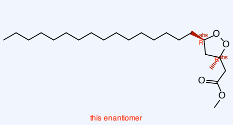	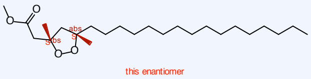	N498	N586	0.90171	3	4	1	10.174
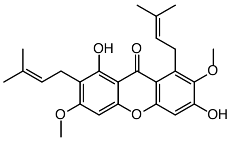	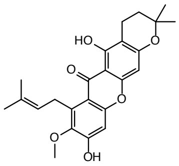	N784	N732	0.89329	7.0673671	6	1.0674	10.002
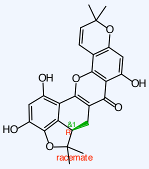	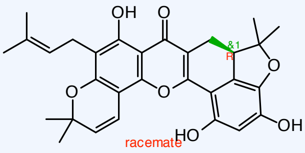	N562	N639	0.8896	3.7	4.8000002	1.1	9.9637
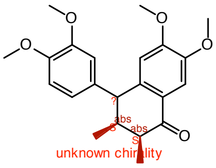	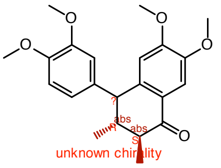	N223	N282	0.96885	0.3199999	0.63	0.31	9.9532
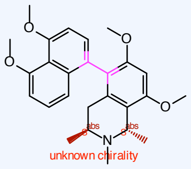	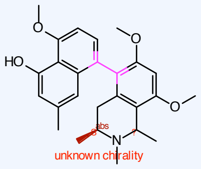	N616	N742	0.82915	4.5	6.1999998	1.7	9.9503

Structure 1	Structure 2	ID 1	ID 2	Similarity	Activity 1	Activity 2	Delta Activity	SALI
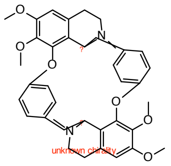	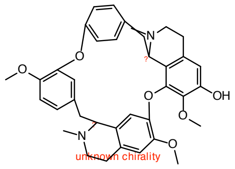	N391	N191	0.86956	1.5	0.2135617	1.2864	9.8625
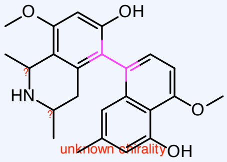	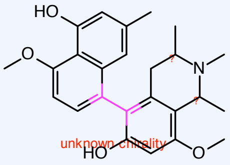	N266	N368	0.91934	0.5286225	1.3202387	0.79162	9.8146
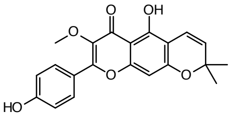	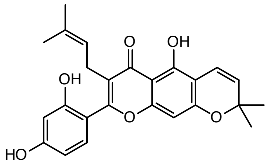	N540	N672	0.8144	3.4000001	5.1999998	1.8	9.6983
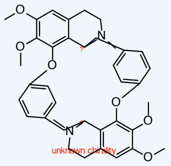	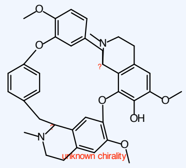	N391	N142	0.85404	1.5	0.0854246	1.4146	9.6917
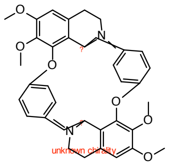	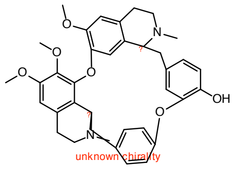	N391	N264	0.89767	1.5	0.5092625	0.99074	9.6821
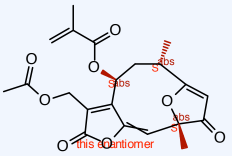	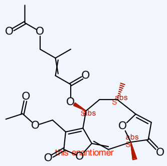	N607	N684	0.89473	4.4732213	5.4799461	1.0067	9.563
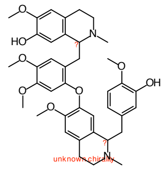	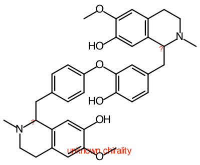	N388	N171	0.85775	1.5	0.16	1.34	9.4197
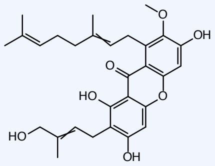	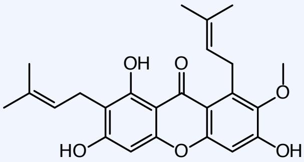	N524	N589	0.90735	3.2350938	4.0999999	0.86491	9.3354
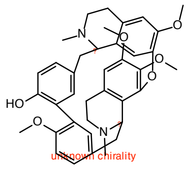	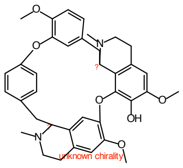	N350	N142	0.88699	1.14	0.0854246	1.0546	9.3313
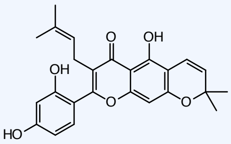	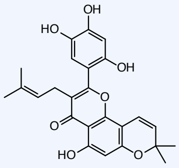	N672	N749	0.87093	5.1999998	6.4000001	1.2	9.2974
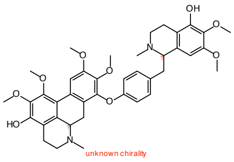	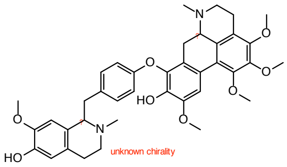	N229	N366	0.89465	0.3405832	1.3158381	0.97525	9.2577

Structure 1	Structure 2	ID 1	ID 2	Similarity	Activity 1	Activity 2	Delta Activity	SALI
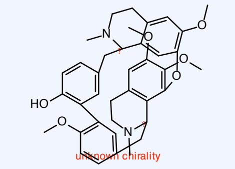	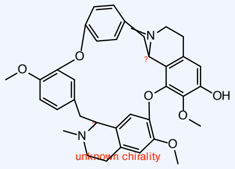	N350	N191	0.89979	1.14	0.2135617	0.92644	9.245
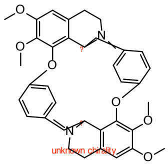	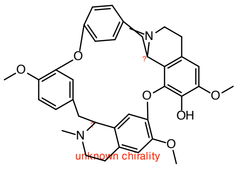	N391	N176	0.85627	1.5	0.1718350	1.3282	9.241
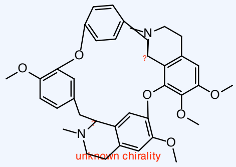	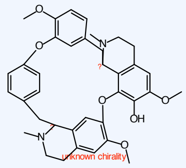	N241	N142	0.9691	0.3709354	0.0854246	0.28551	9.2388
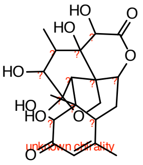	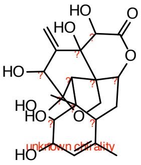	N193	N439	0.80753	0.2265998	2	1.7734	9.2141
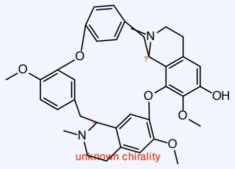	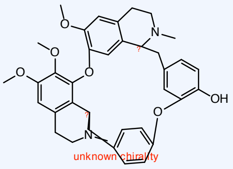	N191	N264	0.96787	0.2135617	0.5092625	0.2957	9.2036
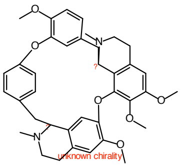	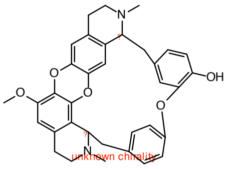	N207	N347	0.90899	0.2649538	1.1	0.83505	9.1751
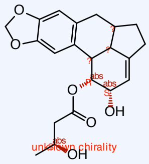	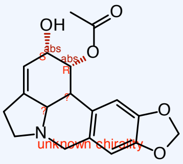	N405	N515	0.84304	1.6	3.0363095	1.4363	9.1508
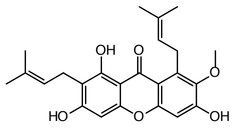	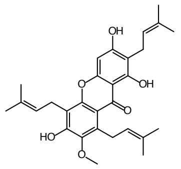	N589	N675	0.87716	4.0999999	5.2238231	1.1238	9.1489
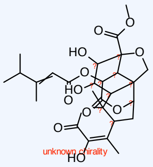	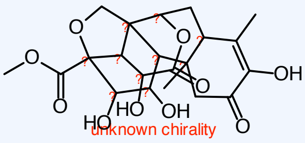	N82	N337	0.88764	0.0014583	1.0286819	1.0272	9.1419
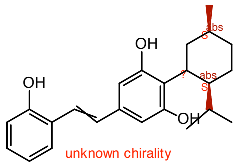	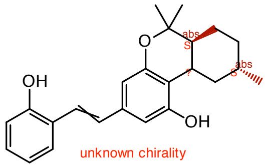	N424	N225	0.84495	1.7462807	0.3292386	1.417	9.1393
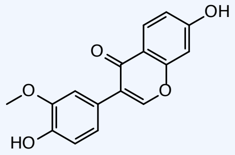	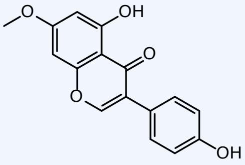	N771	N848	0.80737	6.8000002	8.5500002	1.75	9.0848

Structure 1	Structure 2	ID 1	ID 2	Similarity	Activity 1	Activity 2	Delta Activity	SALI
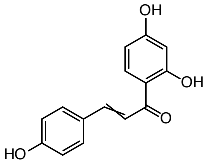	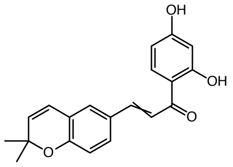	N815	N882	0.80128	7.8000002	9.6000004	1.8	9.0582
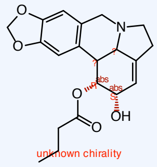	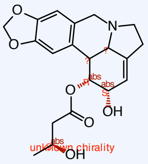	N339	N410	0.93686	1.0352538	1.6068573	0.5716	9.0532
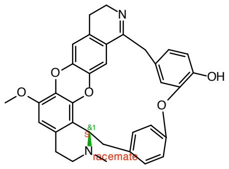	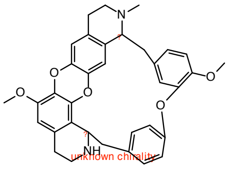	N412	N177	0.83668	1.63	0.1723969[illegible]	1.4576	8.9251
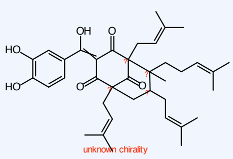	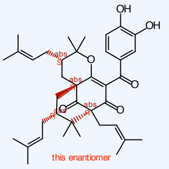	N522	N651	0.79719	3.2	5	1.8	8.8752
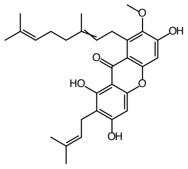	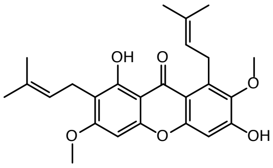	N743	N784	0.90892	6.2685881	7.0673671	0.79878	8.7704
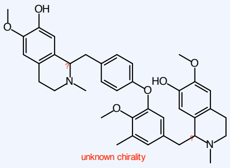	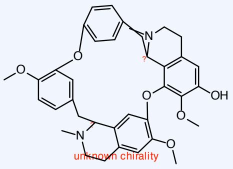	N357	N191	0.87832	1.28	0.2135617[illegible]	1.0664	8.7643
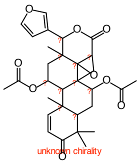	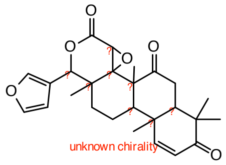	N701	N591	0.81086	5.752852	4.0999999	1.6529	8.7386
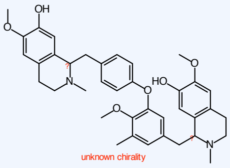	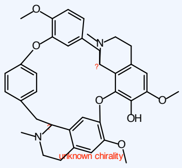	N357	N142	0.86294	1.28	0.0854246[illegible]	1.1946	8.7157
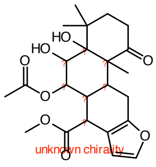	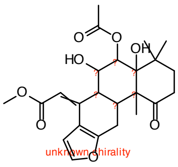	N407	N197	0.8434	1.6	0.2399999[illegible]	1.36	8.6848
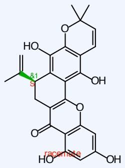	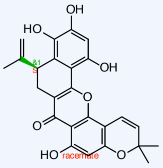	N819	N772	0.8838	7.9000001	6.9000001	1	8.6061
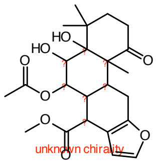	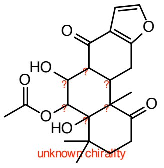	N407	N165	0.83001	1.6	0.14	1.46	8.5888

Structure 1	Structure 2	ID 1	ID 2	Similarity	Activity 1	Activity 2	Delta Activity	SALI
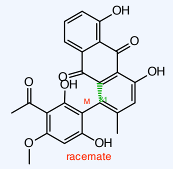	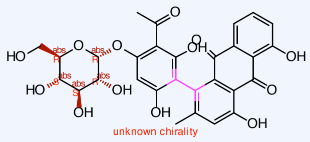	N400	N297	0.90216	1.5423753	0.7038518	0.83852	8.57
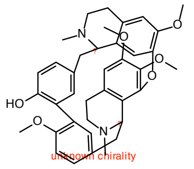	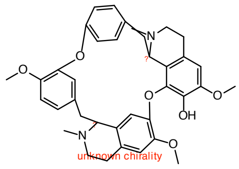	N350	N176	0.88699	1.14	0.1718350	0.96816	8.5667
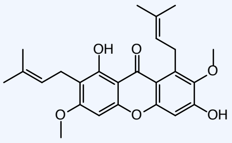	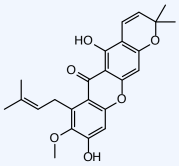	N784	N731	0.87488	7.0673671	6	1.0674	8.5308
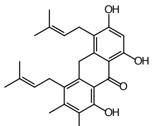	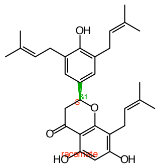	N563	N676	0.81244	3.7	5.3000002	1.6	8.5305
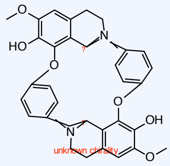	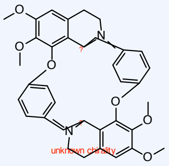	N314	N391	0.92871	0.8920000	1.5	0.608	8.5283
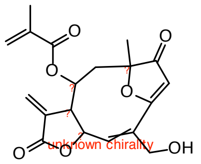	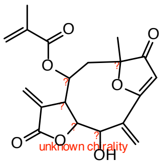	N529	N602	0.87087	3.3	4.4000001	1.1	8.5188
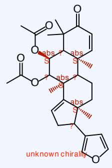	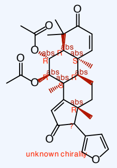	N596	N700	0.82381	4.1999998	5.6999998	1.5	8.5137
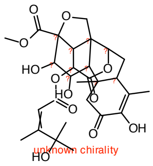	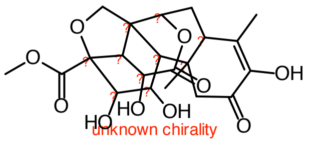	N95	N337	0.87883	0.0088561	1.0286819	1.0198	8.4166
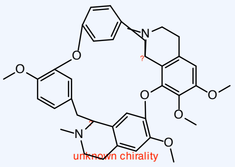	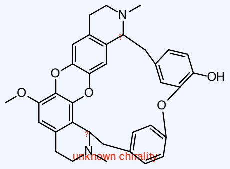	N241	N347	0.91325	0.3709354	1.1	0.72906	8.4041
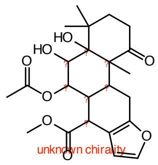	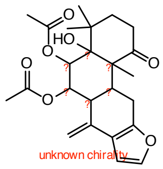	N407	N492	0.84495	1.6	2.9000001	1.3	8.3844
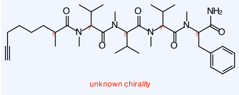	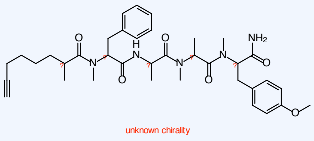	N809	N725	0.79719	7.6999998	6	1.7	8.3822

Structure 1	Structure 2	ID 1	ID 2	Similarity	Activity 1	Activity 2	Delta Activity	SALI
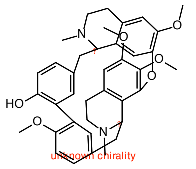	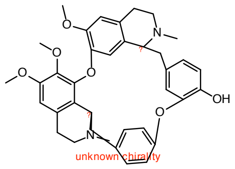	N350	N264	0.92456	1.14	0.5092625	0.63074	8.361
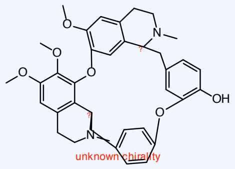	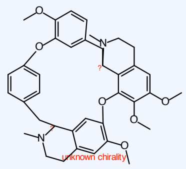	N264	N207	0.97074	0.5092625	0.2649538	0.24431	8.3506
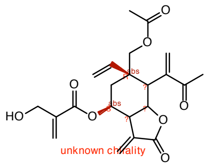	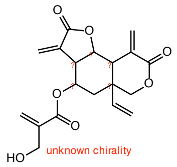	N506	N373	0.80618	3	1.4	1.6	8.2552
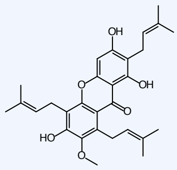	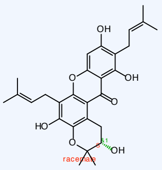	N675	N718	0.90541	5.2238231	6	0.77618	8.2058
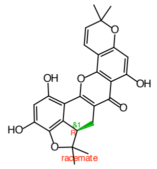	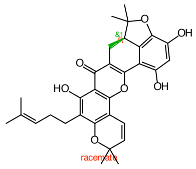	N562	N628	0.88448	3.7	4.645926	0.94593	8.1883
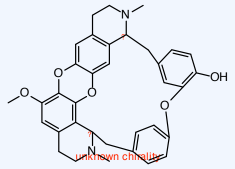	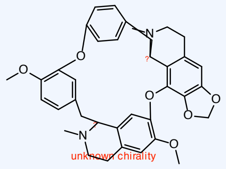	N347	N194	0.89364	1.1	0.2307537	0.86925	8.1725
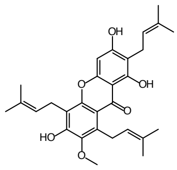	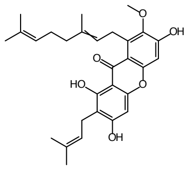	N675	N743	0.87192	5.2238231	6.2685881	1.0448	8.1572
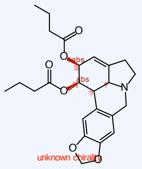	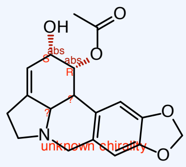	N402	N515	0.81814	1.5672873	3.0363095	1.469	8.0776
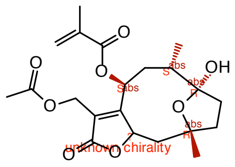	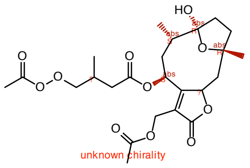	N615	N685	0.87498	4.5	5.5	1	7.9986
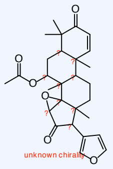	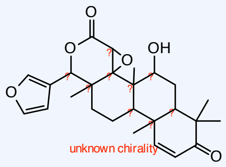	N413	N519	0.81814	1.6503562	3.0999999	1.4496	7.971
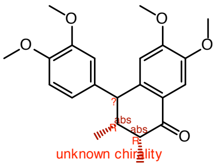	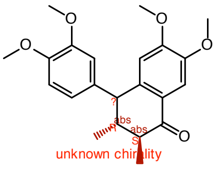	N187	N282	0.94605	0.2	0.63	0.43	7.9708

Structure 1	Structure 2	ID 1	ID 2	Similarity	Activity 1	Activity 2	Delta Activity	SALI
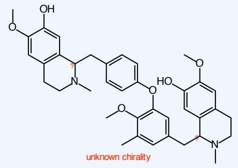	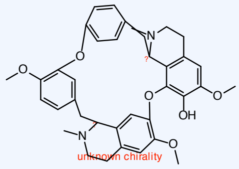	N357	N176	0.86072	1.28	0.17183505	1.1082	7.9566
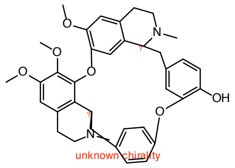	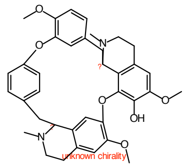	N264	N142	0.94629	0.50926256	0.08542469	0.42384	7.8908
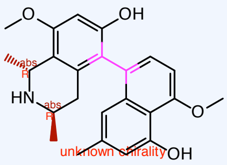	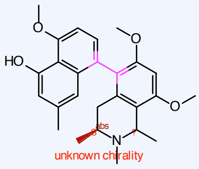	N665	N742	0.85787	5.0829086	6.1999998	1.1171	7.8595
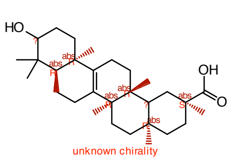	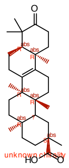	N601	N558	0.92321	4.3000002	3.7	0.6	7.8132
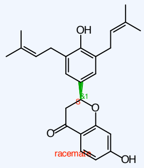	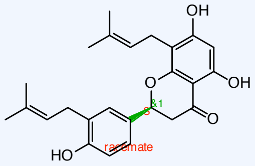	N808	N869	0.81997	7.6999998	9.1000004	1.4	7.7764
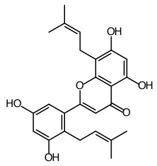	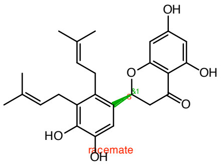	N759	N671	0.81639	6.5999999	5.1999998	1.4	7.6248
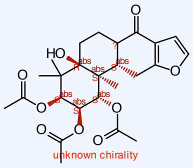	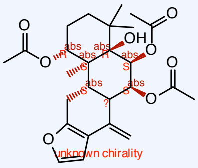	N438	N537	0.81601	2	3.4000001	1.4	7.6092
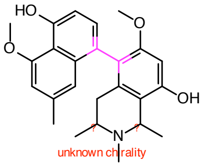	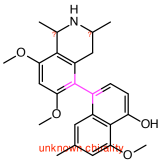	N230	N356	0.87695	0.34355655	1.2760671	0.93251	7.5781
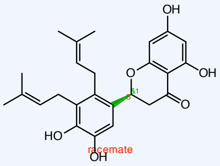	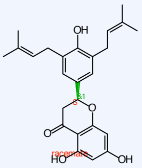	N671	N737	0.88106	5.1999998	6.0999999	0.9	7.5671
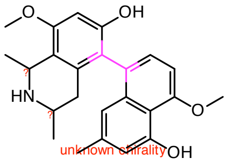	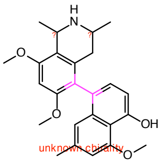	N266	N356	0.90109	0.52862251	1.2760671	0.74744	7.5571
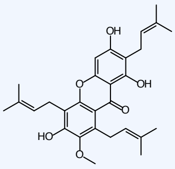	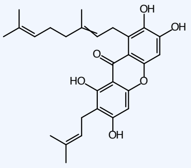	N675	N746	0.8559	5.2238231	6.3000002	1.0762	7.4681

Structure 1	Structure 2	ID 1	ID 2	Similarity	Activity 1	Activity 2	Delta Activity	SALI
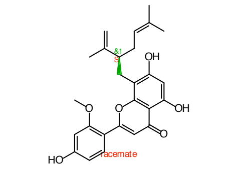	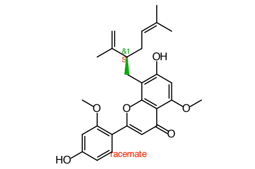	N452	N470	0.95916	2.0999999	2.4000001	0.3	7.3457
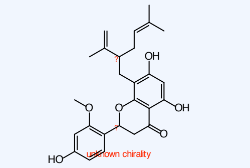	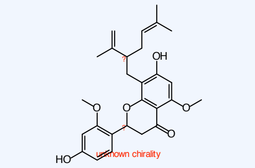	N451	N469	0.95916	2.0999999	2.4000001	0.3	7.3457
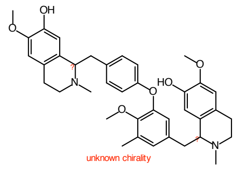	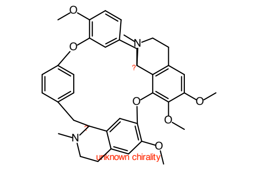	N357	N207	0.86119	1.28	0.26495388	1.015	7.3127
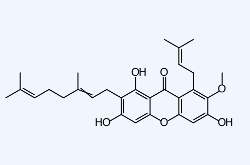	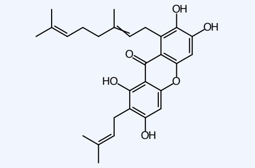	N720	N746	0.95895	6	6.3000002	0.3	7.3083
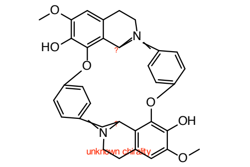	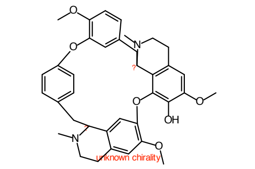	N314	N142	0.88871	0.89200002	0.08542469	0.80658	7.2475
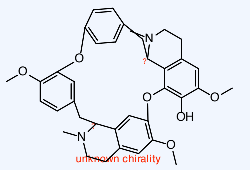	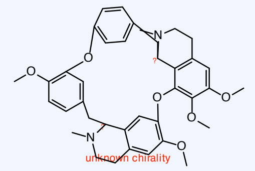	N176	N241	0.97237	0.17183505	0.37093544	0.1991	7.2056
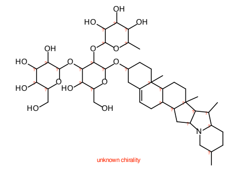	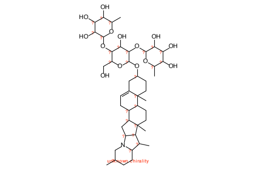	N249	N299	0.95824	0.41299999	0.71380001	0.3008	7.2031
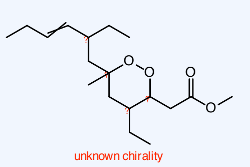	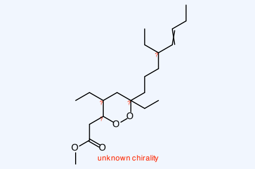	N245	N370	0.86752	0.40000001	1.353928	0.95393	7.2007
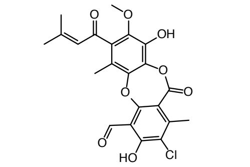	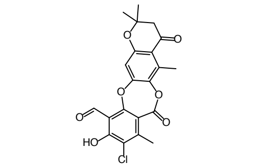	N792	N852	0.80547	7.1999998	8.6000004	1.4	7.1968
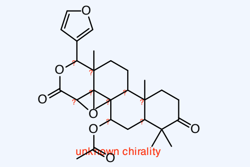	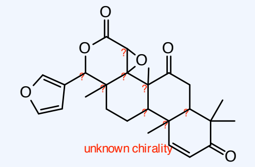	N682	N591	0.81112	5.4273667	4.0999999	1.3274	7.0277
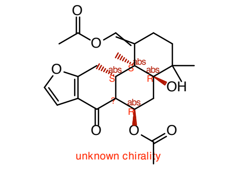	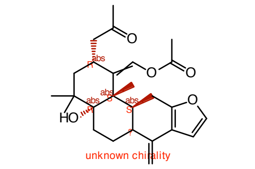	N518	N417	0.80031	3.0999999	1.7	1.4	7.0108

Structure 1	Structure 2	ID 1	ID 2	Similarity	Activity 1	Activity 2	Delta Activity	SALI
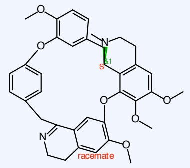	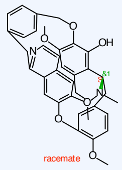	N175	N393	0.80936	0.17	1.5	1.33	6.9763
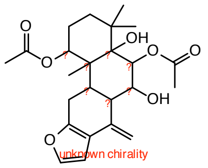	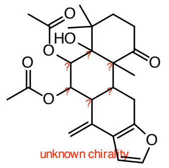	N568	N492	0.86981	3.8	2.9000001	0.9	6.913
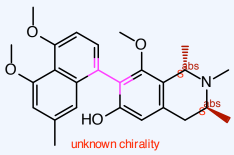	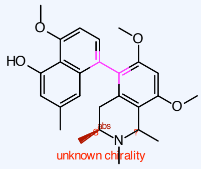	N662	N742	0.82597	5	6.1999998	1.2	6.8953
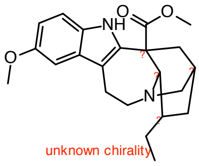	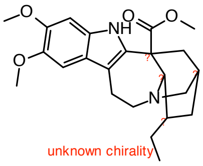	N647	N688	0.90561	4.9122138	5.5559001	0.64369	6.8192
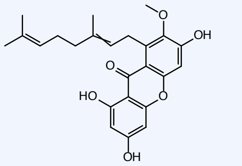	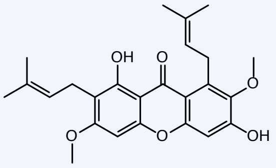	N729	N784	0.84335	6	7.0673671	1.0674	6.8138
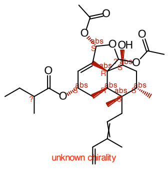	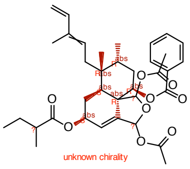	N716	N629	0.80321	6	4.6599998	1.34	6.8093
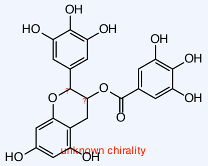	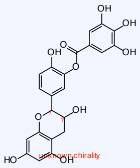	N139	N333	0.86427	0.0790000(	1	0.921	6.7855
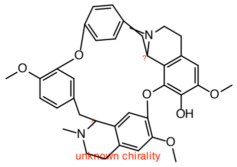	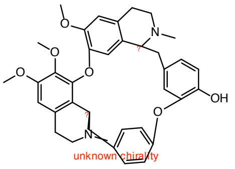	N176	N264	0.95005	0.1718350!	0.5092625(	0.33743	6.7552
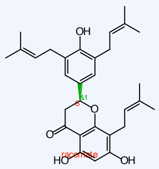	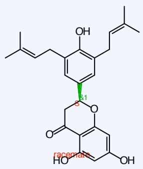	N676	N737	0.88125	5.3000002	6.0999999	0.8	6.7371
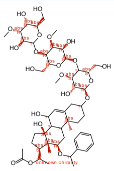	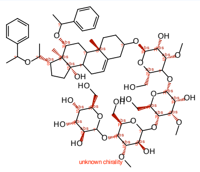	N757	N698	0.88111	6.5	5.6999998	0.8	6.7288
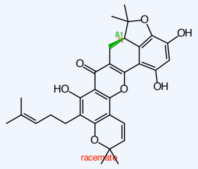	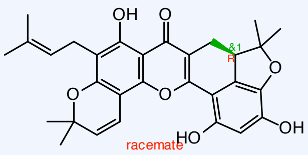	N628	N639	0.9771	4.645926	4.8000002	0.15407	6.7287

Structure 1	Structure 2	ID 1	ID 2	Similarity	Activity 1	Activity 2	Delta Activity	SALI
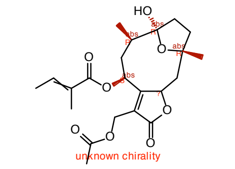	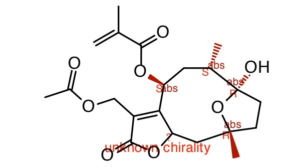	N571	N615	0.9103	3.9000001	4.5	0.6	6.6889
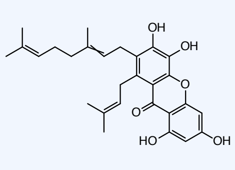	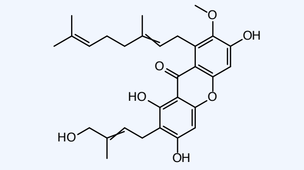	N448	N524	0.82464	2.0665154	3.2350938	1.1686	6.6639
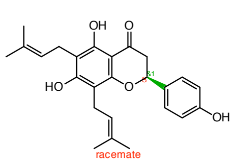	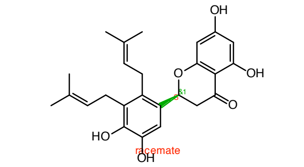	N573	N671	0.80449	3.9000001	5.1999998	1.3	6.6492
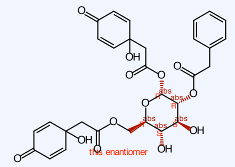	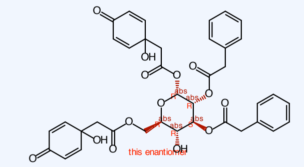	N331	N384	0.92452	1	1.5	0.5	6.6243
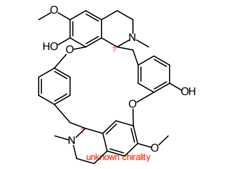	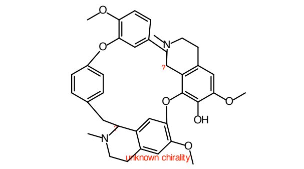	N234	N142	0.95958	0.35299999	0.08542469	0.26758	6.6191
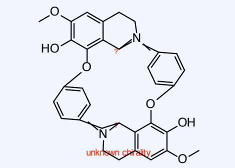	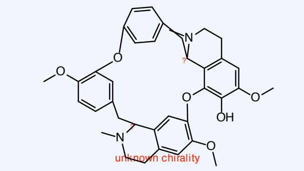	N314	N176	0.89089	0.89200002	0.17183505	0.72016	6.6006
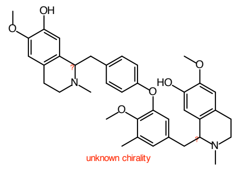	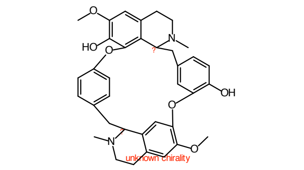	N357	N234	0.858	1.28	0.35299999	0.927	6.5281
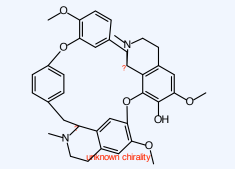	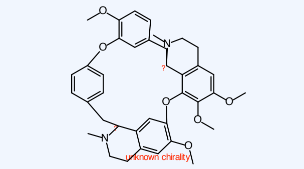	N142	N207	0.97237	0.08542469	0.26495388	0.17953	6.4973
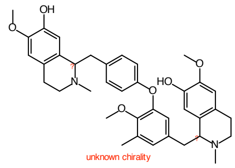	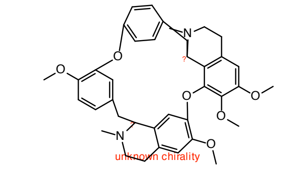	N357	N241	0.85899	1.28	0.37093544	0.90906	6.447
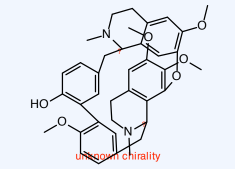	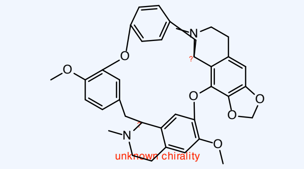	N350	N194	0.8585	1.14	0.23075373	0.90925	6.4258
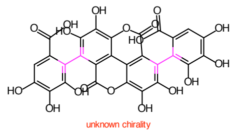	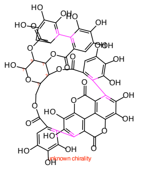	N803	N859	0.79671	7.5	8.8000002	1.3	6.3948

Structure 1	Structure 2	ID 1	ID 2	Similarity	Activity 1	Activity 2	Delta Activity	SALI
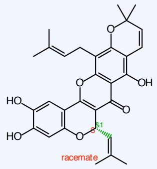	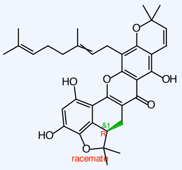	N113	N364	0.79741	0.02	1.3099999	1.29	6.3676
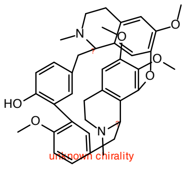	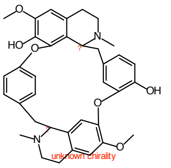	N350	N234	0.8736	1.14	0.35299999	0.787	6.2262
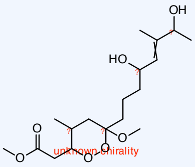	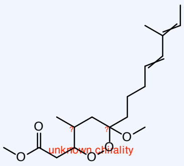	N659	N727	0.83681	5	6	1	6.1278
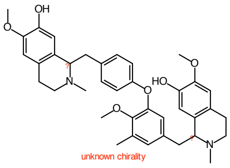	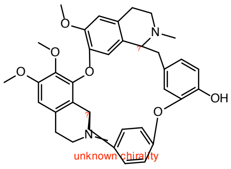	N357	N264	0.87395	1.28	0.50926256	0.77074	6.1147
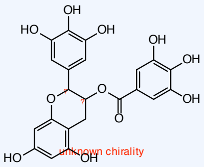	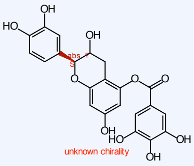	N139	N352	0.81619	0.0790000(	1.2	1.121	6.0988
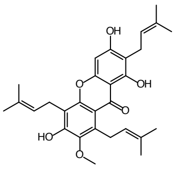	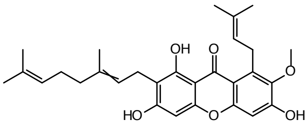	N675	N720	0.87192	5.2238231	6	0.77618	6.0601
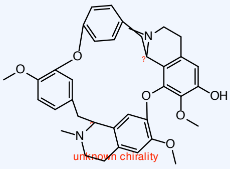	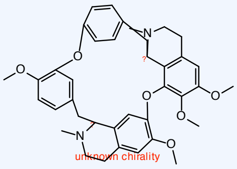	N191	N241	0.97397	0.2135617:	0.3709354	0.15737	6.046
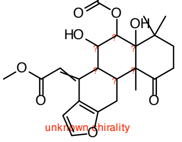	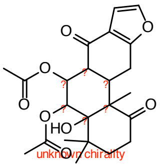	N197	N328	0.87394	0.23999999	1	0.76	6.0291
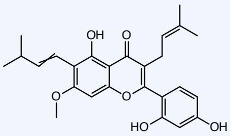	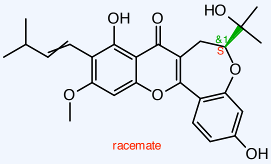	N773	N813	0.86721	6.9000001	7.6999998	0.8	6.0245
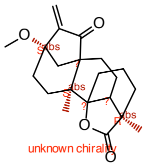	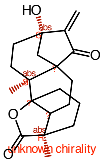	N403	N445	0.92286	1.5677412	2.0277345	0.45999	5.9633
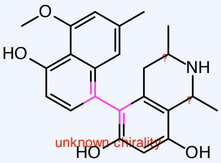	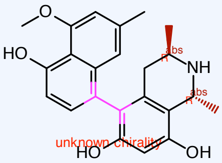	N136	N254	0.9308	0.06324962	0.4743721	0.41112	5.9414

Structure 1	Structure 2	ID 1	ID 2	Similarity	Activity 1	Activity 2	Delta Activity	SALI
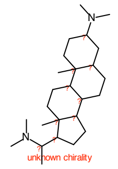	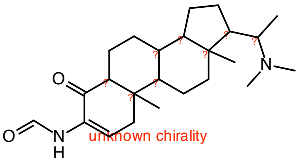	N472	N543	0.81453	2.4000001	3.5	1.1	5.9307
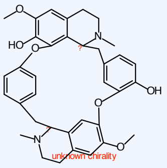	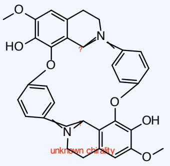	N234	N314	0.90808	0.3529999	0.8920000	0.539	5.8635
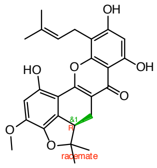	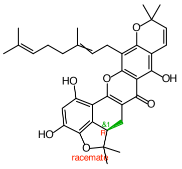	N179	N364	0.80719	0.1800000	1.3099999	1.13	5.8607
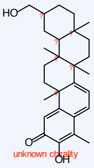	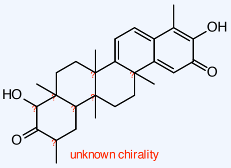	N133	N311	0.87093	0.0541884	0.8000000	0.74581	5.7784
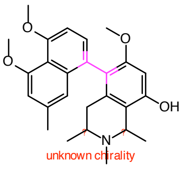	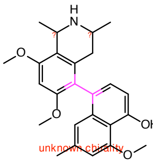	N235	N356	0.84015	0.3558477	1.2760671	0.92022	5.7568
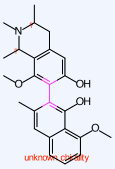	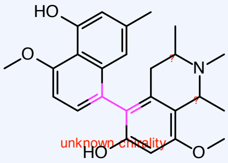	N200	N368	0.81211	0.2453975	1.3202387	1.0748	5.7207
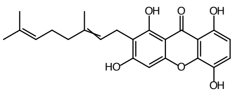	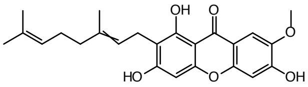	N504	N555	0.88544	3	3.6544397	0.65444	5.7125
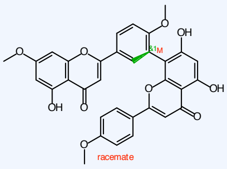	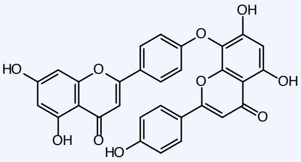	N378	N255	0.83594	1.4	0.4799999	0.92	5.6079
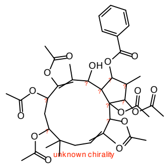	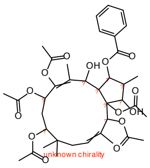	N703	N717	0.96414	5.8000002	6	0.2	5.5772
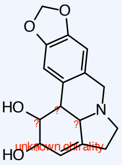	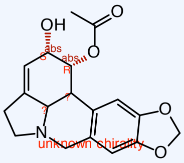	N449	N515	0.82972	2.0883331	3.0363095	0.94798	5.567
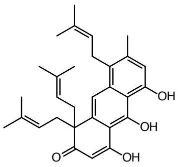	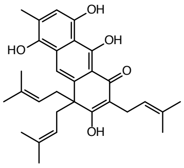	N488	N428	0.83832	2.7	1.8	0.9	5.5664

Structure 1	Structure 2	ID 1	ID 2	Similarity	Activity 1	Activity 2	Delta Activity	SALI
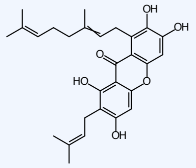	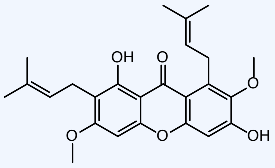	N746	N784	0.85836	6.3000002	7.0673671	0.76737	5.4177
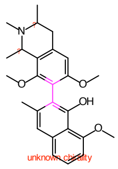	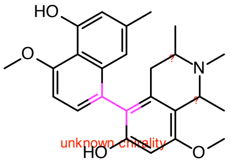	N196	N368	0.79793	0.23723181	1.3202387	1.083	5.3594
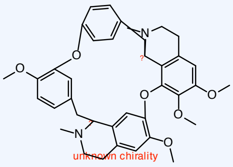	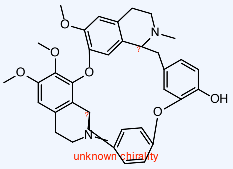	N241	N264	0.97397	0.37093544	0.50926256	0.13833	5.3143
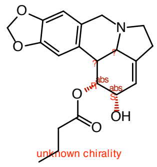	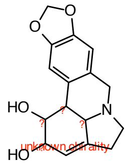	N339	N449	0.80079	1.0352538	2.0883331	1.0531	5.2863
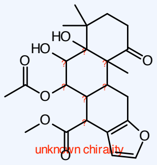	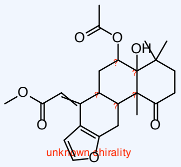	N407	N289	0.81959	1.6	0.64999998	0.95	5.2658
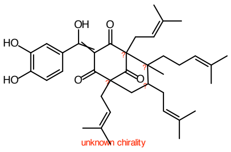	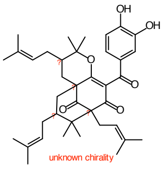	N522	N457	0.80912	3.2	2.2	1	5.2389
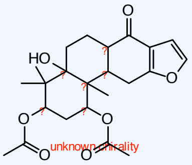	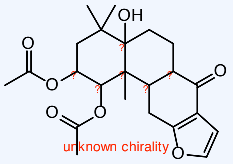	N204	N308	0.8956	0.25999999	0.80000001	0.54	5.1726
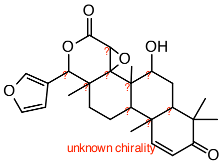	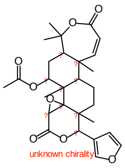	N519	N581	0.82585	3.0999999	4	0.9	5.168
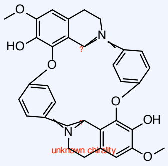	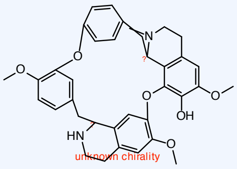	N314	N154	0.84777	0.89200002	0.10593635	0.78606	5.1638
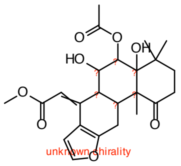	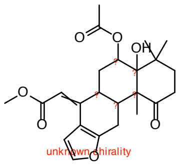	N197	N289	0.92048	0.23999999	0.64999998	0.41	5.1559
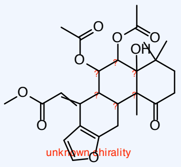	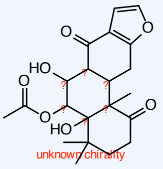	N327	N165	0.82784	1	0.14	0.86	4.9953

Structure 1	Structure 2	ID 1	ID 2	Similarity	Activity 1	Activity 2	Delta Activity	SALI
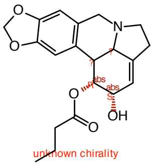	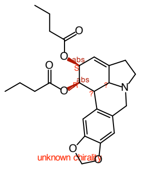	N339	N402	0.89334	1.0352538	1.5672873	0.53203	4.9881
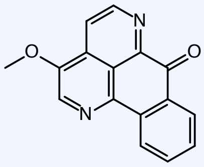	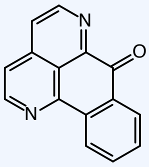	N342	N429	0.85007	1.0676314	1.8084987	0.74087	4.9413
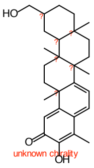	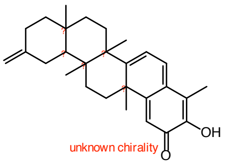	N133	N250	0.92566	0.0541884	0.4201844	0.366	4.9232
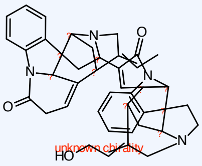	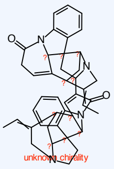	N152	N185	0.97931	0.1	0.2	0.1	4.8339
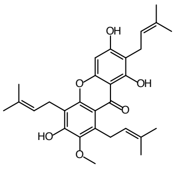	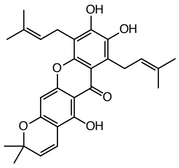	N675	N719	0.83612	5.2238231	6	0.77618	4.7364
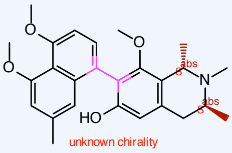	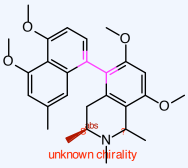	N662	N605	0.87093	5	4.4000001	0.6	4.6487
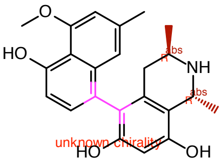	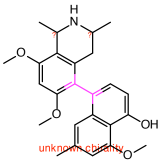	N254	N356	0.82465	0.4743721	1.2760671	0.80169	4.572
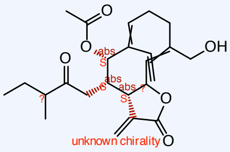	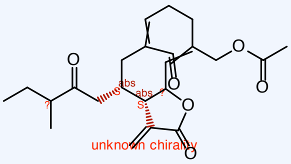	N461	N491	0.85318	2.23	2.9000001	0.67	4.5633
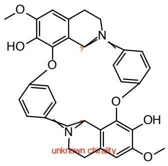	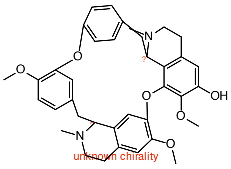	N314	N191	0.85067	0.8920000	0.2135617	0.67844	4.5431
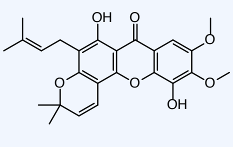	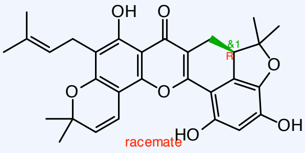	N575	N639	0.80187	3.9000001	4.8000002	0.9	4.5425
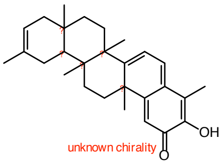	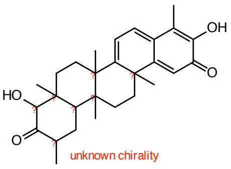	N195	N311	0.87288	0.2323372	0.8000000	0.56766	4.4654

Structure 1	Structure 2	ID 1	ID 2	Similarity	Activity 1	Activity 2	Delta Activity	SALI
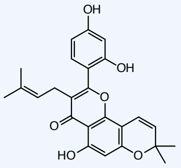	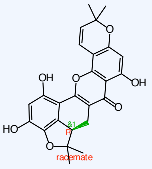	N617	N562	0.81211	4.5	3.7	0.8	4.2579
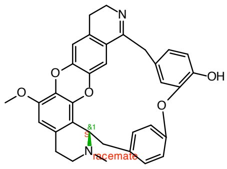	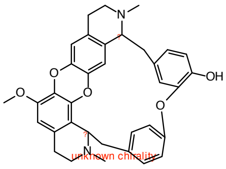	N412	N347	0.8752	1.63	1.1	0.53	4.2468
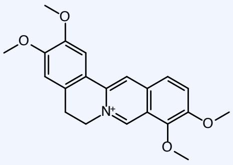	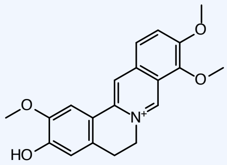	N302	N338	0.94338	0.79454333	1.034349	0.23981	4.2354
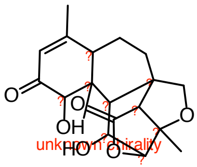	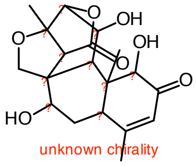	N189	N283	0.89885	0.20379445	0.63119245	0.4274	4.2252
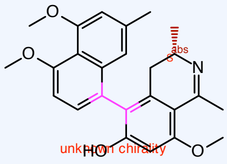	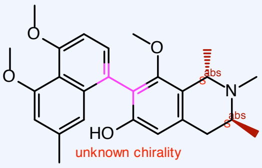	N604	N662	0.85608	4.4000001	5	0.6	4.1688
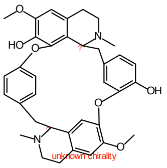	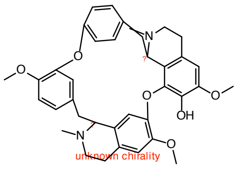	N234	N176	0.95595	0.35299999	0.17183505	0.18116	4.1123
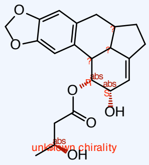	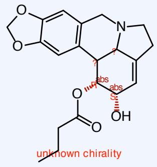	N405	N339	0.86137	1.6	1.0352538	0.56475	4.0739
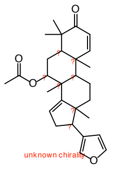	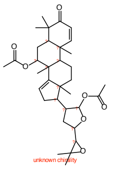	N19	N434	0.82073	1.21	1.9340975	0.7241	4.0392
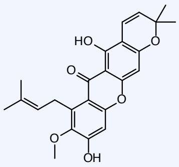	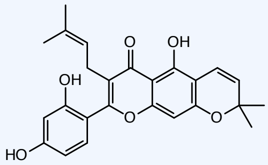	N731	N672	0.80121	6	5.1999998	0.8	4.0243
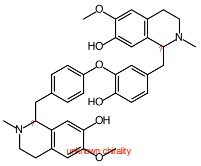	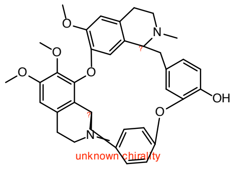	N171	N264	0.91234	0.16	0.50926256	0.34926	3.9841
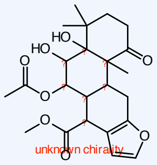	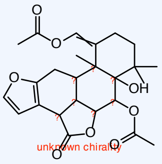	N407	N303	0.79859	1.6	0.80000001	0.8	3.972

Structure 1	Structure 2	ID 1	ID 2	Similarity	Activity 1	Activity 2	Delta Activity	SALI
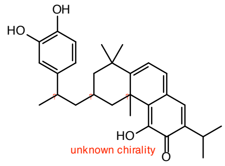	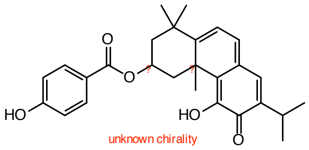	N630	N677	0.84645	4.6999998	5.3000002	0.6	3.9075
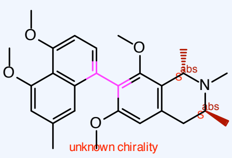	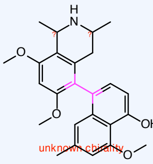	N442	N356	0.81318	2	1.2760671	0.72393	3.8751
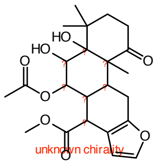	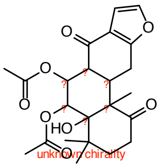	N407	N328	0.84495	1.6	1	0.6	3.8697
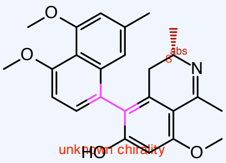	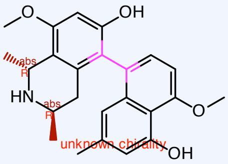	N604	N665	0.82332	4.4000001	5.0829086	0.68291	3.8653
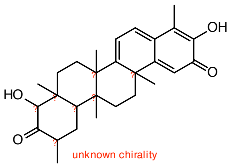	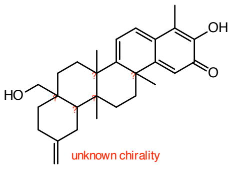	N311	N212	0.86183	0.80000001	0.2710519	0.52895	3.8284
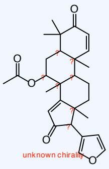	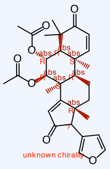	N750	N700	0.8154	6.4000001	5.6999998	0.7	3.7919
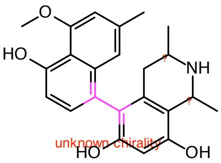	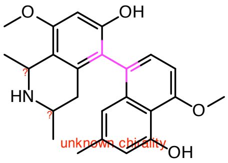	N136	N266	0.87695	0.0632496	0.5286225	0.46537	3.7819
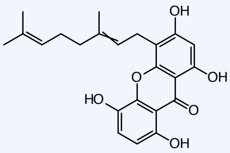	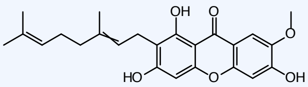	N508	N555	0.82667	3	3.6544397	0.65444	3.7757
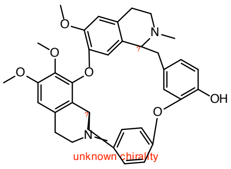	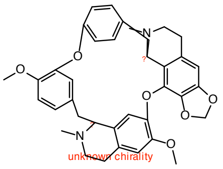	N264	N194	0.92467	0.5092625	0.2307537	0.27851	3.6974
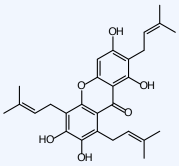	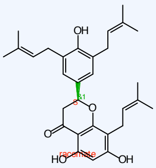	N722	N676	0.80847	6	5.3000002	0.7	3.6547
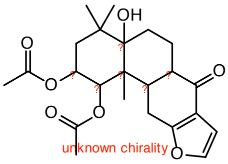	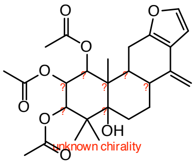	N308	N184	0.83579	0.8000000	0.2	0.6	3.6538

Structure 1	Structure 2	ID 1	ID 2	Similarity	Activity 1	Activity 2	Delta Activity	SALI
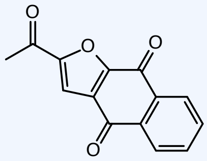	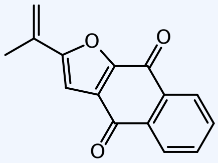	N85	N168	0.95923	0.0020000(	0.15000001	0.148	3.6297
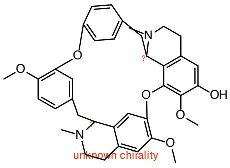	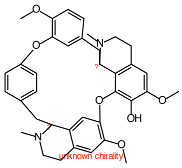	N191	N142	0.96445	0.21356173	0.08542469	0.12814	3.6048
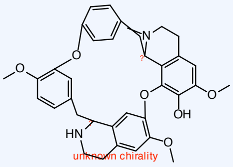	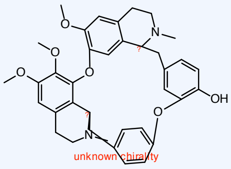	N154	N264	0.88652	0.10593635	0.5092625(	0.40333	3.5541
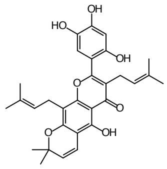	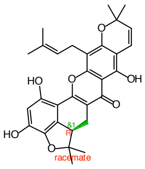	N340	N267	0.85954	1.04	0.5500000	0.49	3.4885
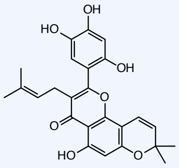	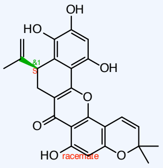	N749	N772	0.85405	6.4000001	6.9000001	0.5	3.4258
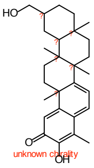	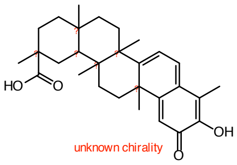	N133	N246	0.89828	0.0541884	0.4014561	0.34727	3.4141
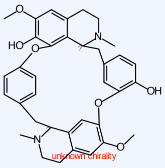	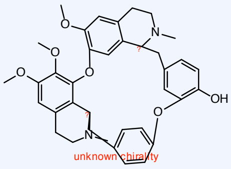	N234	N264	0.95411	0.3529999	0.5092625(	0.15626	3.4048
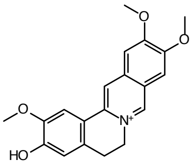	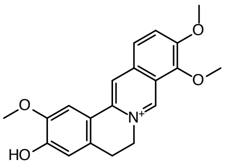	N285	N338	0.88322	0.6399999	1.034349	0.39435	3.3768
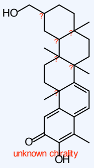	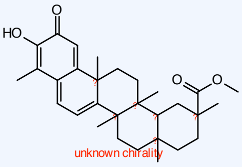	N133	N247	0.89443	0.0541884	0.4097829	0.35559	3.3682
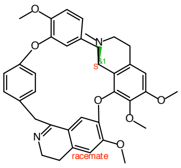	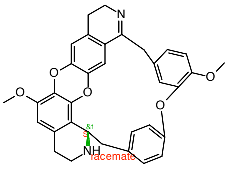	N175	N269	0.88218	0.17	0.5616411	0.39164	3.3242
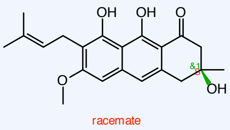	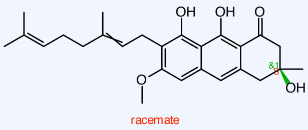	N394	N420	0.9398	1.5	1.7	0.2	3.322

Structure 1	Structure 2	ID 1	ID 2	Similarity	Activity 1	Activity 2	Delta Activity	SALI
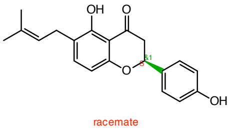	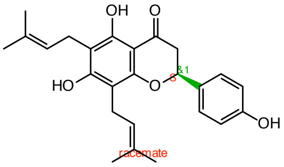	N525	N573	0.80103	3.24	3.9000001	0.66	3.317
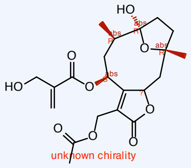	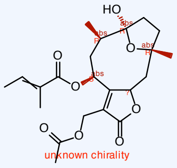	N557	N571	0.93965	3.7	3.9000001	0.2	3.314
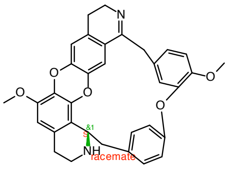	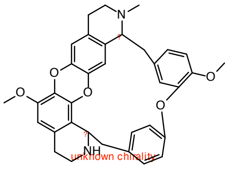	N269	N177	0.88226	0.5616411	0.17239699	0.38924	3.3058
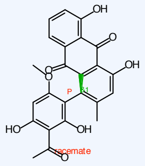	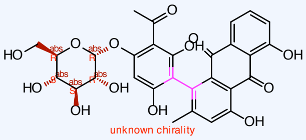	N213	N297	0.87157	0.28	0.70385188	0.42385	3.3003
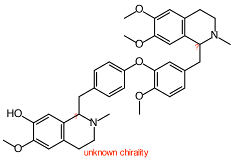	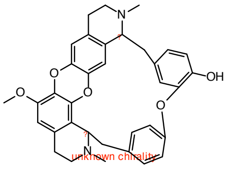	N389	N347	0.87828	1.5	1.1	0.4	3.2861
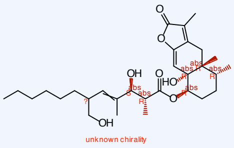	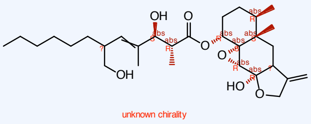	N680	N715	0.81564	5.4000001	6	0.6	3.2545
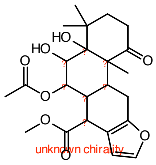	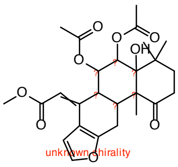	N407	N327	0.81211	1.6	1	0.6	3.1934
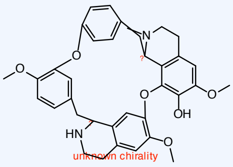	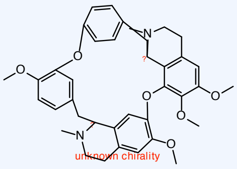	N154	N241	0.91648	0.10593635	0.37093544	0.265	3.173
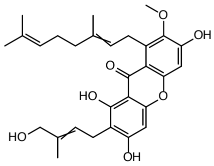	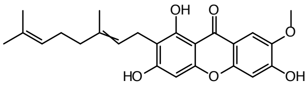	N524	N555	0.86776	3.2350938	3.6544397	0.41935	3.171
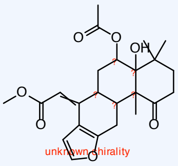	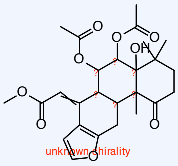	N289	N327	0.88831	0.64999998	1	0.35	3.1337
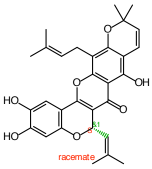	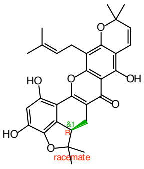	N113	N267	0.82917	0.02	0.55000001	0.53	3.1025

Structure 1	Structure 2	ID 1	ID 2	Similarity	Activity 1	Activity 2	Delta Activity	SALI
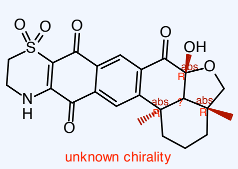	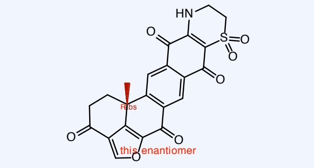	N7	N343	0.80579	0.5	1.1	0.6	3.0894
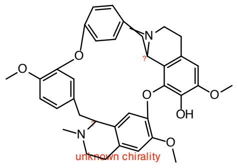	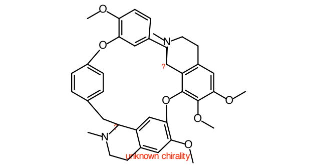	N176	N207	0.9691	0.1718350	0.2649538	9.3119e-2	3.0132
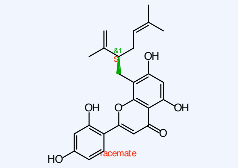	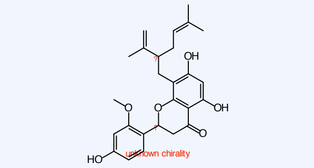	N483	N451	0.83096	2.5999999	2.0999999	0.5	2.958
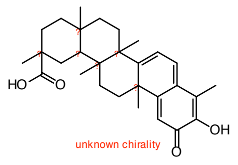	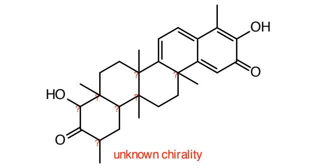	N246	N311	0.86427	0.4014561	0.8000000	0.39854	2.9363
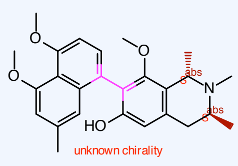	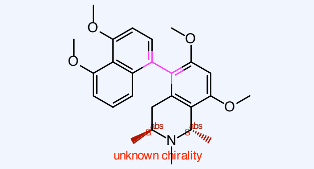	N662	N616	0.82915	5	4.5	0.5	2.9266
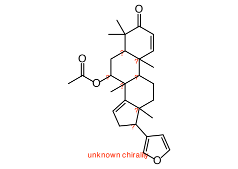	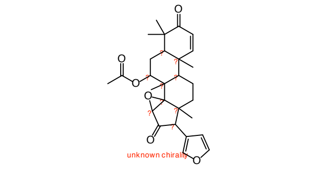	N19	N413	0.8479	1.21	1.6503562	0.44036	2.8951
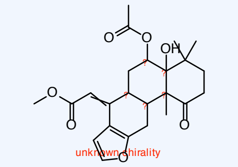	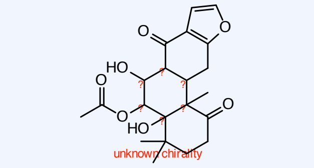	N289	N165	0.82332	0.6499999	0.14	0.51	2.8866
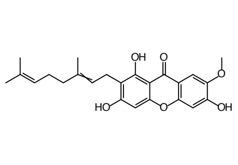	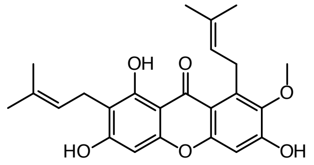	N555	N589	0.84495	3.6544397	4.0999999	0.44556	2.8737
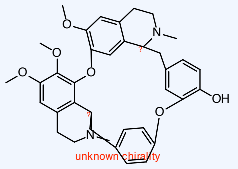	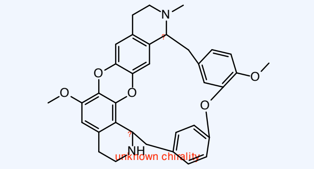	N264	N177	0.88247	0.5092625	0.1723969	0.33687	2.8662
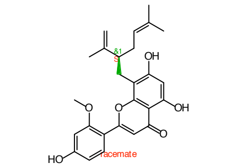	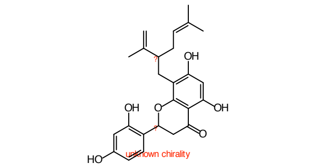	N452	N482	0.82471	2.0999999	2.5999999	0.5	2.8523
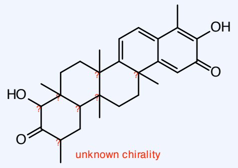	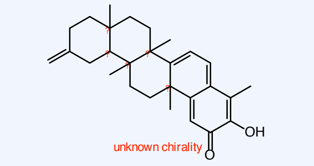	N311	N250	0.86674	0.8000000	0.4201844	0.37982	2.8501

Structure 1	Structure 2	ID 1	ID 2	Similarity	Activity 1	Activity 2	Delta Activity	SALI
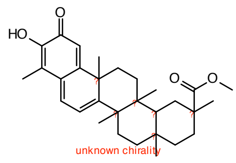	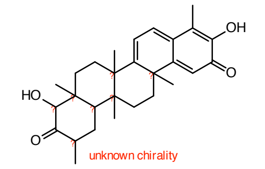	N247	N311	0.86078	0.4097829	0.8000000	0.39022	2.8028
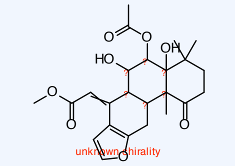	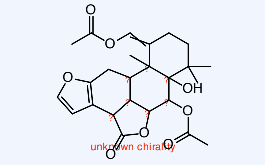	N197	N303	0.79777	0.2399999	0.8000000	0.56	2.7692
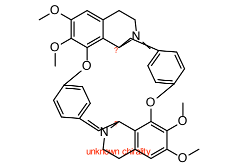	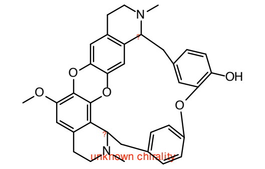	N391	N347	0.85328	1.5	1.1	0.4	2.7264
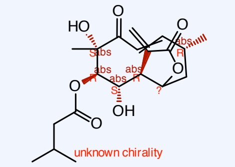	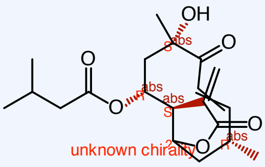	N279	N317	0.88991	0.62	0.9200000	0.3	2.7249
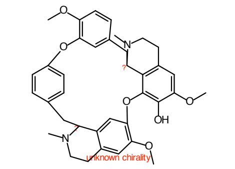	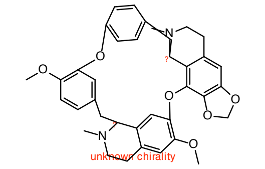	N142	N194	0.94629	0.0854246	0.2307537	0.14533	2.7057
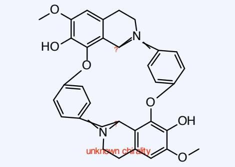	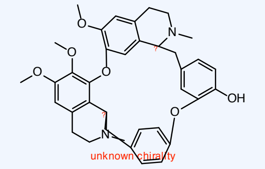	N314	N264	0.85749	0.8920000	0.5092625	0.38274	2.6857
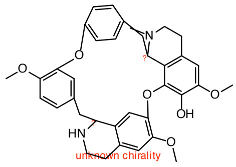	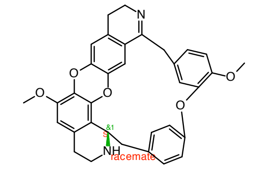	N154	N269	0.83005	0.1059363	0.5616411	0.4557	2.6814
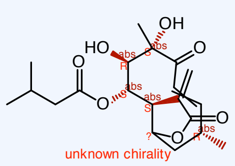	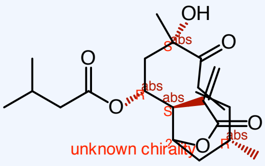	N278	N317	0.88639	0.62	0.9200000	0.3	2.6407
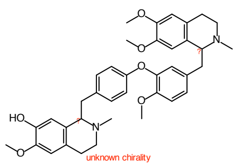	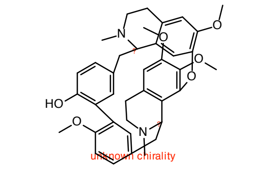	N389	N350	0.86339	1.5	1.14	0.36	2.6353
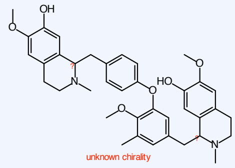	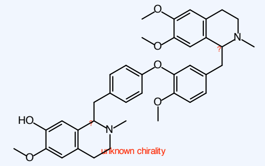	N357	N389	0.91641	1.28	1.5	0.22	2.6317
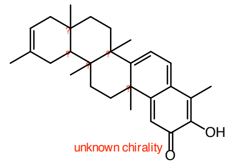	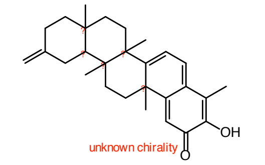	N195	N250	0.92835	0.2323372	0.4201844	0.18785	2.6218

Structure 1	Structure 2	ID 1	ID 2	Similarity	Activity 1	Activity 2	Delta Activity	SALI
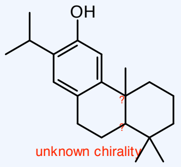	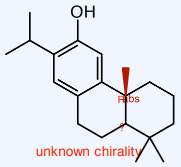	N533	N541	0.93284	3.3164415	3.4909911	0.17455	2.5988
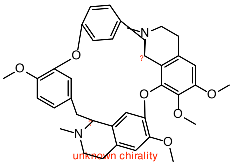	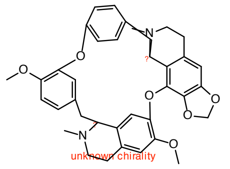	N241	N194	0.946	0.3709354‹	0.2307537:	0.14018	2.5957
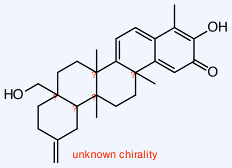	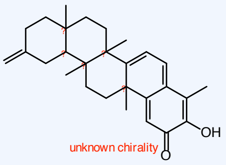	N212	N250	0.94232	0.2710519:	0.4201844(	0.14913	2.5856
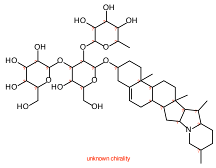	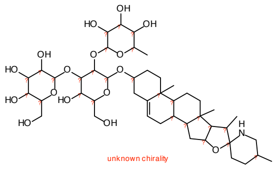	N249	N272	0.93637	0.4129999¢	0.5747	0.1617	2.5411
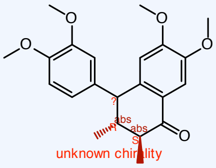	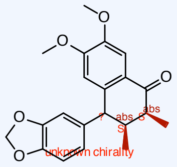	N282	N205	0.8541	0.63	0.2599999¢	0.37	2.5361
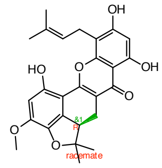	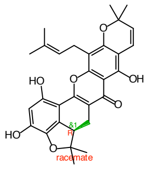	N179	N267	0.85209	0.1800000:	0.5500000:	0.37	2.5015
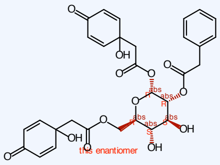	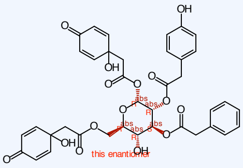	N331	N305	0.91977	1	0.8000000:	0.2	2.4928
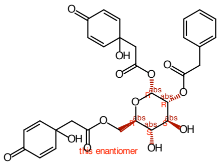	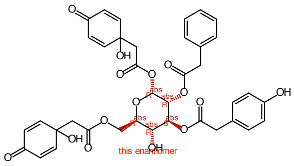	N331	N304	0.91977	1	0.8000000:	0.2	2.4928
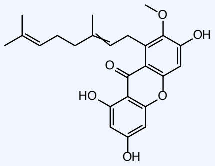	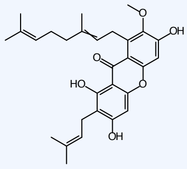	N729	N743	0.89158	6	6.2685881	0.26859	2.4772
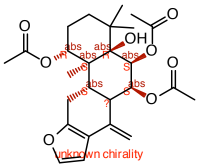	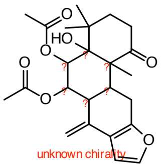	N537	N492	0.79777	3.4000001	2.9000001	0.5	2.4725
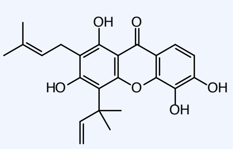	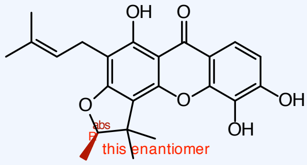	N344	N372	0.87803	1.1	1.4	0.3	2.4597

Structure 1	Structure 2	ID 1	ID 2	Similarity	Activity 1	Activity 2	Delta Activity	SALI
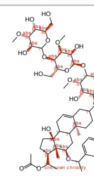	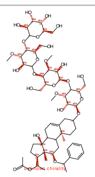	N799	N802	0.95856	7.4000001	7.5	0.1	2.4132
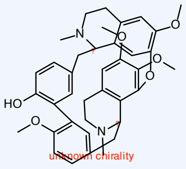	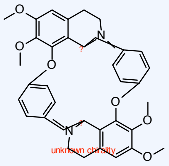	N350	N391	0.85014	1.14	1.5	0.36	2.4023
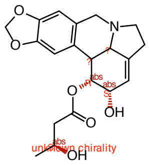	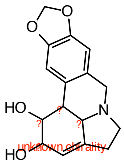	N410	N449	0.79546	1.6068573	2.0883331	0.48148	2.3539
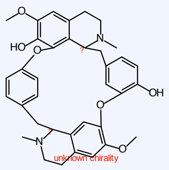	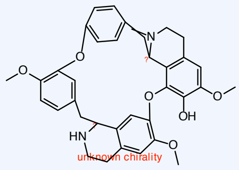	N234	N154	0.89289	0.3529999	0.1059363	0.24706	2.3067
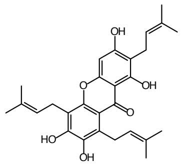	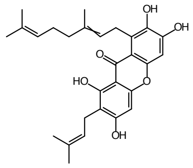	N722	N746	0.86764	6	6.3000002	0.3	2.2666
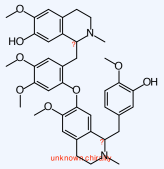	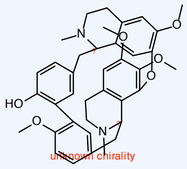	N388	N350	0.8371	1.5	1.14	0.36	2.21
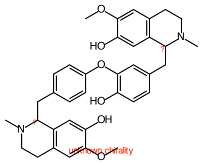	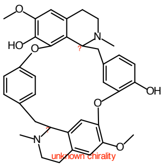	N171	N234	0.91021	0.16	0.3529999	0.193	2.1495
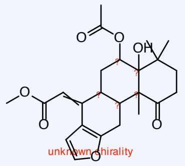	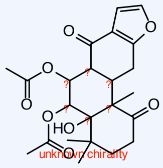	N289	N328	0.83549	0.6499999	1	0.35	2.1276
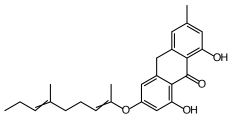	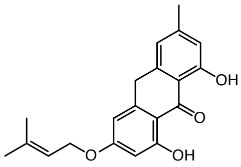	N416	N435	0.85775	1.6799999	1.98	0.3	2.1089
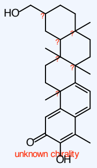	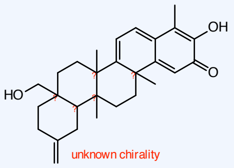	N133	N212	0.89402	0.0541884	0.2710519	0.21686	2.0462
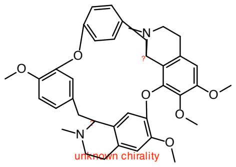	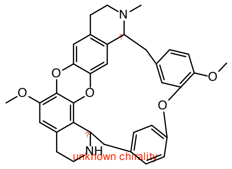	N241	N177	0.90253	0.3709354	0.1723969	0.19854	2.0369

Structure 1	Structure 2	ID 1	ID 2	Similarity	Activity 1	Activity 2	Delta Activity	SALI
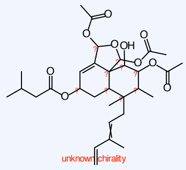	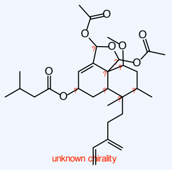	N673	N686	0.85233	5.1999998	5.5	0.3	2.0316
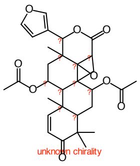	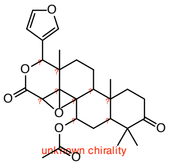	N701	N682	0.83785	5.752852	5.4273667	0.32549	2.0073
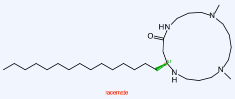	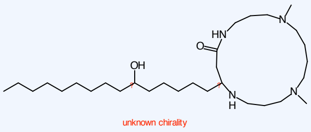	N186	N226	0.935	0.2	0.33000001	0.13	2
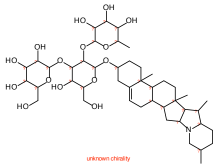	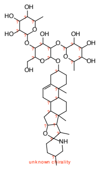	N249	N287	0.88148	0.41299999	0.64889997	0.2359	1.9903
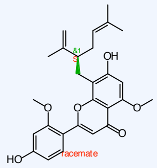	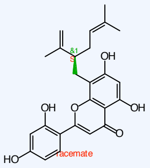	N470	N483	0.89828	2.4000001	2.5999999	0.2	1.9663
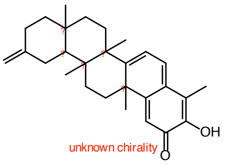	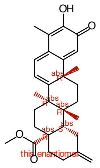	N250	N119	0.79944	0.42018446	0.02700000	0.39318	1.9604
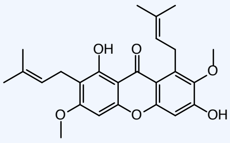	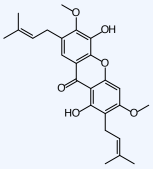	N784	N794	0.90581	7.0673671	7.25	0.18263	1.9391
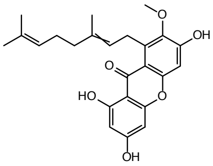	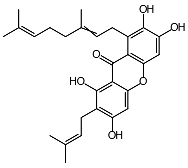	N729	N746	0.84495	6	6.3000002	0.3	1.9349
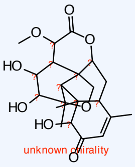	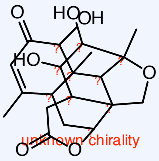	N106	N190	0.8991	0.015	0.20999999	0.195	1.9326
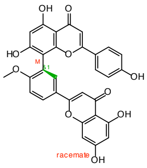	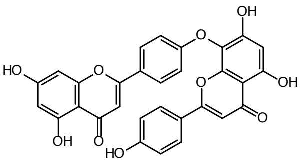	N219	N255	0.9062	0.30000001	0.47999999	0.18	1.9189
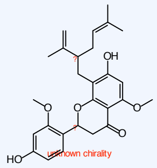	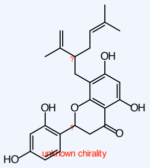	N469	N482	0.89541	2.4000001	2.5999999	0.2	1.9123

Structure 1	Structure 2	ID 1	ID 2	Similarity	Activity 1	Activity 2	Delta Activity	SALI
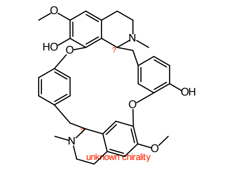	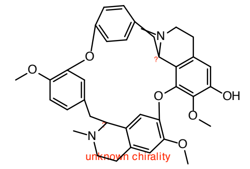	N234	N191	0.92683	0.35299999	0.21356173	0.13944	1.9056
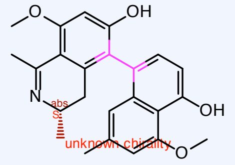	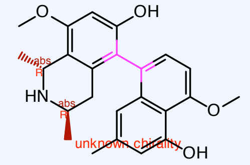	N681	N665	0.83145	5.4000001	5.0829086	0.31709	1.8813
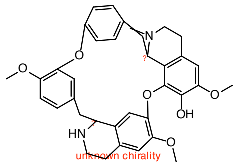	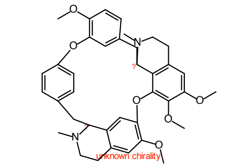	N154	N207	0.91441	0.10593635	0.26495388	0.15902	1.858
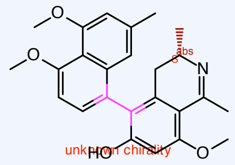	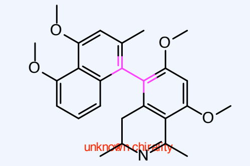	N604	N638	0.80121	4.4000001	4.7674356	0.36744	1.8483
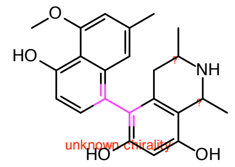	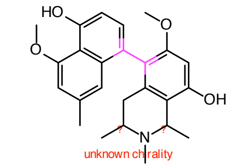	N136	N230	0.8483	0.06324962	0.34355655	0.28031	1.8478
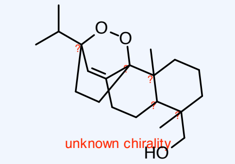	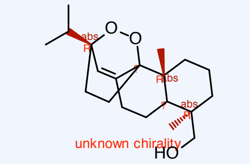	N151	N231	0.86593	0.09799999	0.34400001	0.246	1.8349
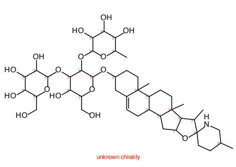	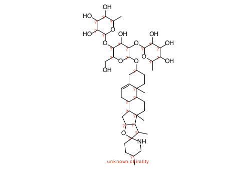	N272	N287	0.95913	0.5747	0.64889997	0.0742	1.8153
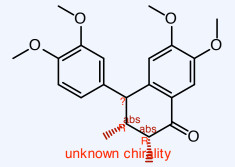	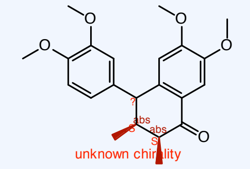	N187	N223	0.93373	0.2	0.31999999	0.12	1.8109
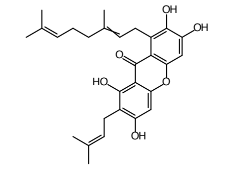	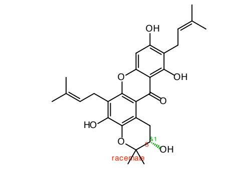	N746	N718	0.83219	6.3000002	6	0.3	1.7877
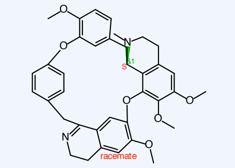	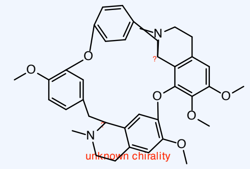	N175	N241	0.88699	0.17	0.37093544	0.20094	1.778
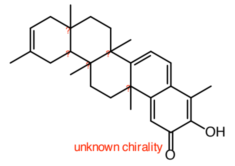	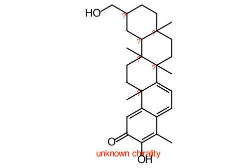	N195	N133	0.89932	0.23233728	0.05418841	0.17815	1.7694

Structure 1	Structure 2	ID 1	ID 2	Similarity	Activity 1	Activity 2	Delta Activity	SALI
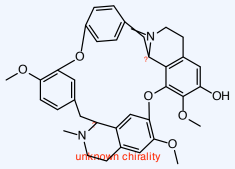	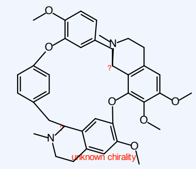	N191	N207	0.97074	0.2135617	0.2649538	5.1392e-2	1.7566
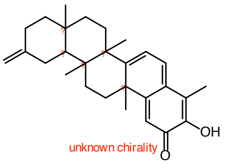	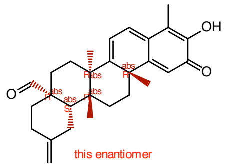	N250	N148	0.81429	0.4201844	0.0939999	0.32618	1.7564
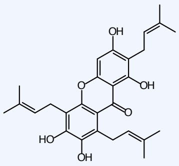	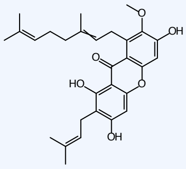	N722	N743	0.84464	6	6.2685881	0.26859	1.7288
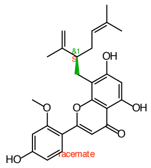	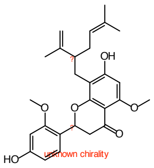	N452	N469	0.82229	2.0999999	2.4000001	0.3	1.6882
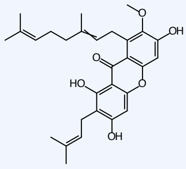	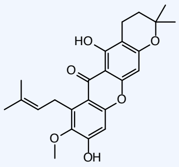	N743	N732	0.8386	6.2685881	6	0.26859	1.6641
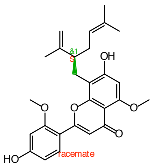	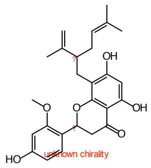	N470	N451	0.81924	2.4000001	2.0999999	0.3	1.6597
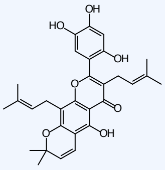	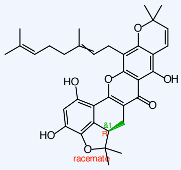	N340	N364	0.83705	1.04	1.3099999	0.27	1.6569
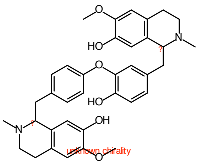	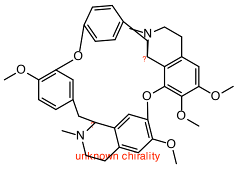	N171	N241	0.87139	0.16	0.3709354	0.21094	1.6401
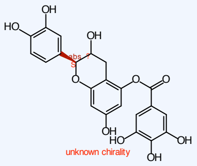	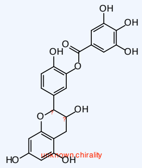	N352	N333	0.87793	1.2	1	0.2	1.6384
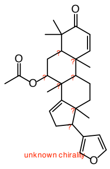	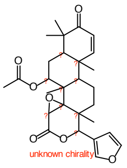	N19	N382	0.82592	1.21	1.4920259	0.28203	1.6201
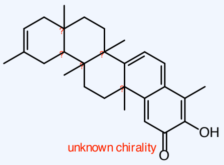	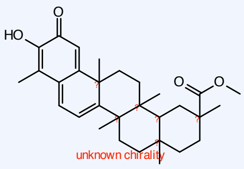	N195	N247	0.88793	0.2323372	0.4097829	0.17745	1.5833

Structure 1	Structure 2	ID 1	ID 2	Similarity	Activity 1	Activity 2	Delta Activity	SALI
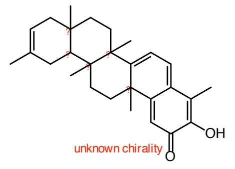	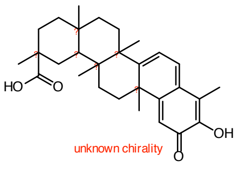	N195	N246	0.89179	0.23233728	0.40145615	0.16912	1.5629
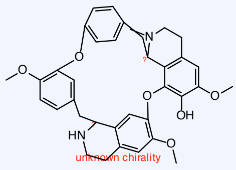	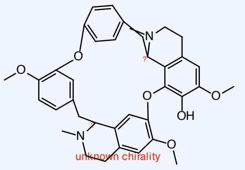	N154	N176	0.95777	0.10593635	0.17183505	6.5899e-2	1.5604
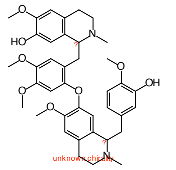	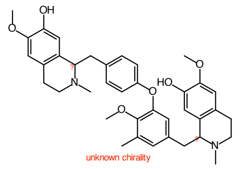	N388	N357	0.85872	1.5	1.28	0.22	1.5571
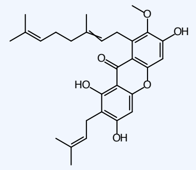	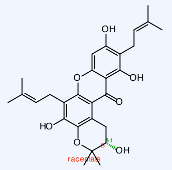	N743	N718	0.8264	6.2685881	6	0.26859	1.5472
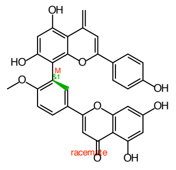	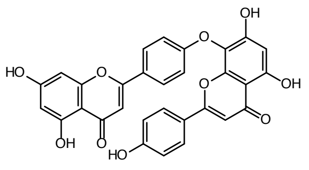	N220	N255	0.88153	0.30000001	0.47999999	0.18	1.5194
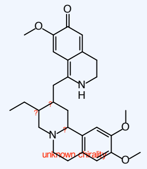	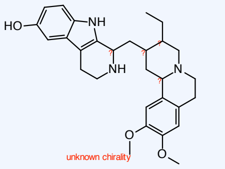	N221	N102	0.79975	0.3013368	0.01261505	0.28872	1.4418
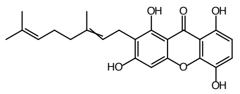	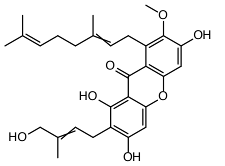	N504	N524	0.83674	3	3.2350938	0.23509	1.44
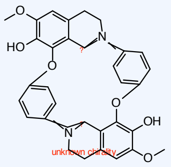	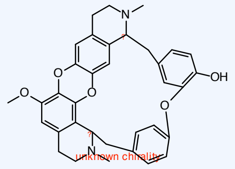	N314	N347	0.85448	0.89200002	1.1	0.208	1.4294
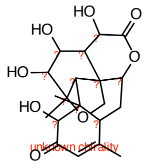	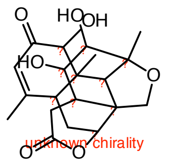	N137	N190	0.90171	0.07100000	0.20999999	0.139	1.4142
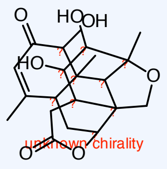	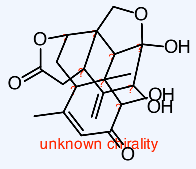	N190	N94	0.85572	0.20999999	0.00797023	0.20203	1.4003
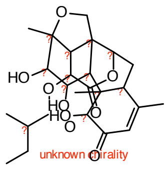	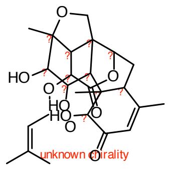	N92	N153	0.93188	0.00626917	0.10010153	9.3832e-2	1.3775

Structure 1	Structure 2	ID 1	ID 2	Similarity	Activity 1	Activity 2	Delta Activity	SALI
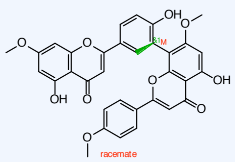	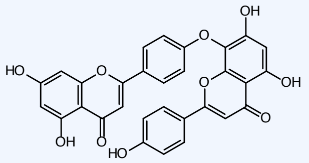	N203	N255	0.83594	0.2599999	0.4799999	0.22	1.341
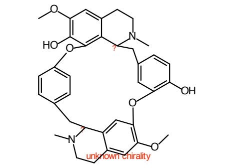	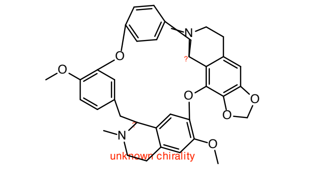	N234	N194	0.90811	0.3529999	0.2307537	0.12225	1.3304
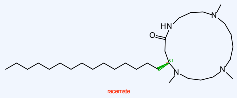	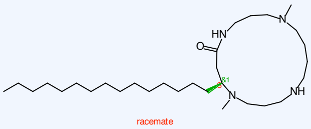	N178	N199	0.9546	0.1800000	0.2399999	0.06	1.3214
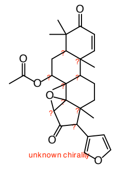	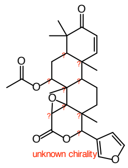	N413	N382	0.87968	1.6503562	1.4920259	0.15833	1.3159
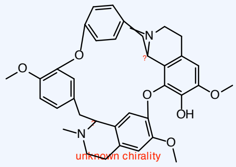	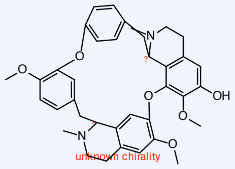	N176	N191	0.96787	0.1718350	0.2135617	4.1727e-2	1.2987
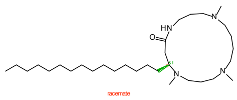	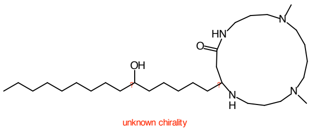	N178	N226	0.88447	0.1800000	0.3300000	0.15	1.2984
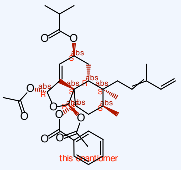	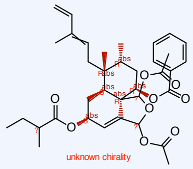	N606	N629	0.8259	4.4400001	4.6599998	0.22	1.2636
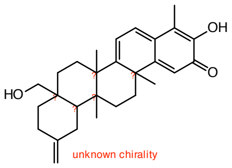	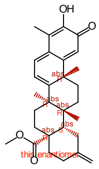	N212	N119	0.80484	0.2710519	0.0270000	0.24405	1.2506
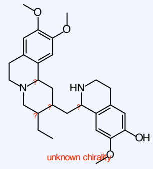	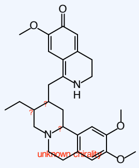	N135	N221	0.80429	0.0578638	0.3013368	0.24347	1.2441
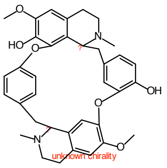	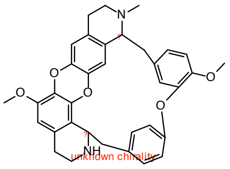	N234	N177	0.85448	0.3529999	0.1723969	0.1806	1.2411
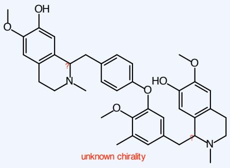	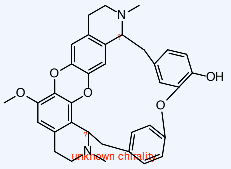	N357	N347	0.85098	1.28	1.1	0.18	1.2079

Structure 1	Structure 2	ID 1	ID 2	Similarity	Activity 1	Activity 2	Delta Activity	SALI
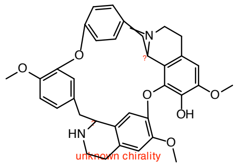	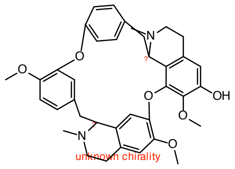	N154	N191	0.91023	0.1059363	0.2135617	0.10763	1.1989
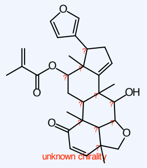	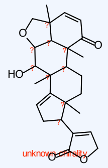	N641	N663	0.80547	4.8299999	5.0599999	0.23	1.1823
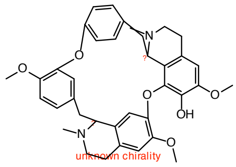	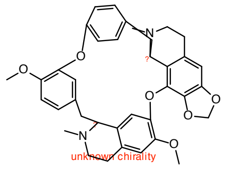	N176	N194	0.95005	0.1718350	0.2307537	5.8919e-2	1.1795
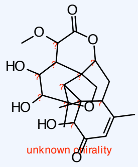	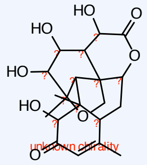	N106	N137	0.95123	0.015	0.0710000	0.056	1.1483
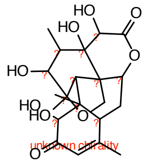	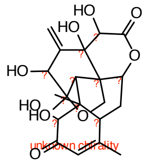	N193	N150	0.88773	0.2265998	0.0979433	0.12866	1.1459
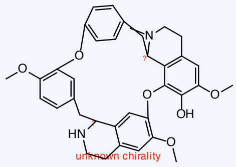	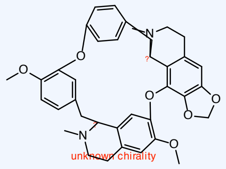	N154	N194	0.89089	0.1059363	0.2307537	0.12482	1.144
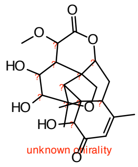	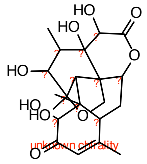	N106	N193	0.8144	0.015	0.2265998	0.2116	1.1401
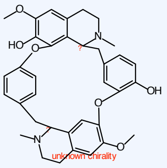	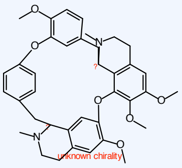	N234	N207	0.9206	0.3529999	0.2649538	8.8046e-2	1.1089
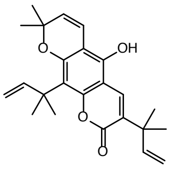	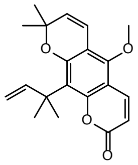	N365	N398	0.80316	1.3141408	1.5319271	0.21779	1.1064
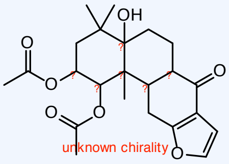	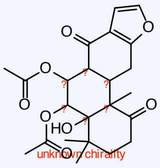	N308	N328	0.81754	0.8000000	1	0.2	1.0962
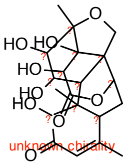	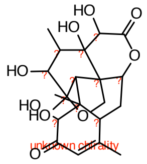	N122	N193	0.82465	0.0365483	0.2265998	0.19005	1.0839

Structure 1	Structure 2	ID 1	ID 2	Similarity	Activity 1	Activity 2	Delta Activity	SALI
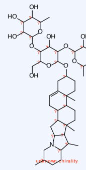	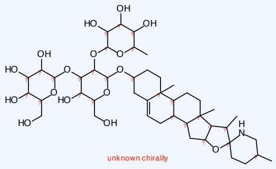	N299	N272	0.87139	0.71380001	0.5747	0.1391	1.0816
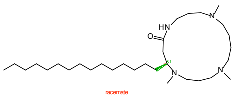	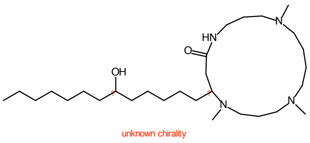	N178	N214	0.89733	0.18000001	0.28999999	0.11	1.0713
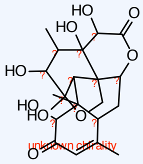	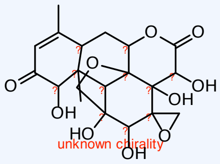	N193	N126	0.83001	0.22659998	0.04712551	0.17947	1.0558
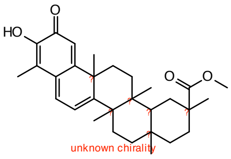	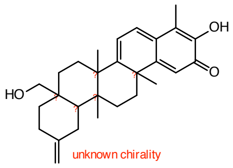	N247	N212	0.86839	0.40978298	0.27105191	0.13873	1.0541
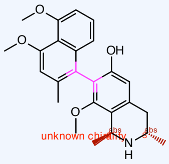	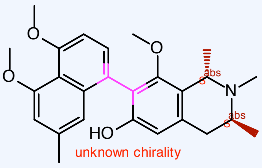	N640	N662	0.80776	4.8000002	5	0.2	1.0404
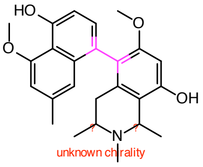	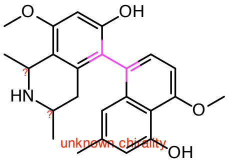	N230	N266	0.81661	0.34355655	0.52862251	0.18507	1.0091
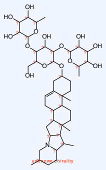	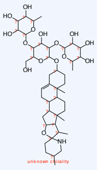	N299	N287	0.93502	0.71380001	0.64889997	0.0649	0.99869
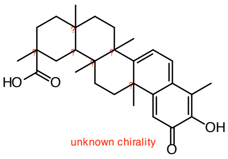	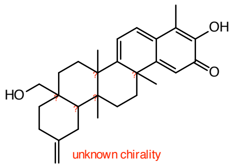	N246	N212	0.86904	0.40145615	0.27105191	0.1304	0.99578
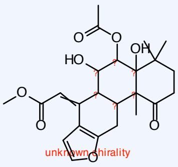	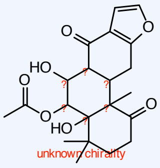	N197	N165	0.8956	0.23999999	0.14	0.1	0.95789
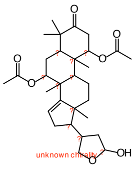	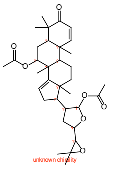	N20	N434	0.79619	1.74	1.9340975	0.1941	0.95235
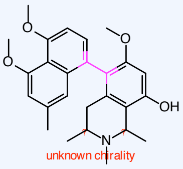	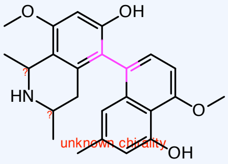	N235	N266	0.81543	0.35584772	0.52862251	0.17277	0.93609

Structure 1	Structure 2	ID 1	ID 2	Similarity	Activity 1	Activity 2	Delta Activity	SALI
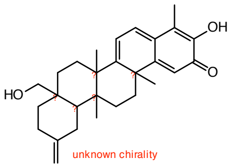	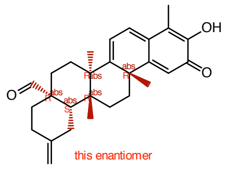	N212	N148	0.80997	0.2710519	0.0939999	0.17705	0.93172
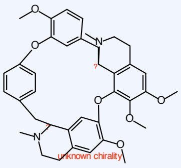	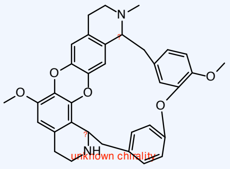	N207	N177	0.90036	0.2649538	0.1723969	9.2557e-2	0.92893
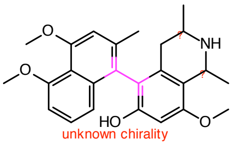	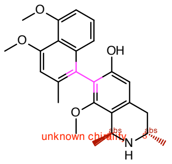	N646	N640	0.88322	4.9079509	4.8000002	0.10795	0.92438
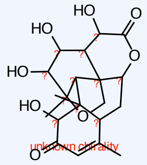	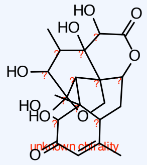	N137	N193	0.82948	0.0710000	0.2265998	0.1556	0.91253
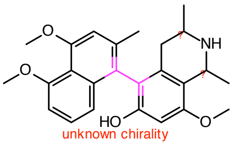	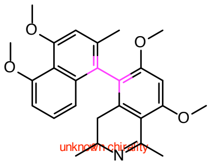	N646	N638	0.83694	4.9079509	4.7674356	0.14052	0.86176
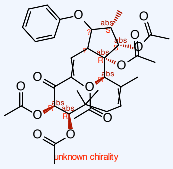	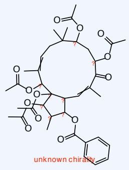	N664	N643	0.80234	5.0690041	4.9000001	0.169	0.85504
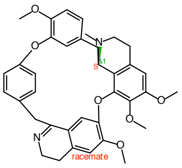	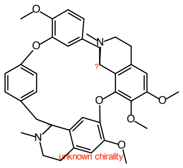	N175	N207	0.88699	0.17	0.2649538	9.4954e-2	0.84019
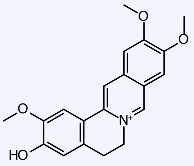	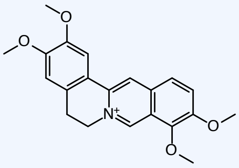	N285	N302	0.81601	0.6399999	0.7945433	0.15454	0.83997
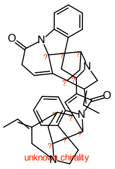	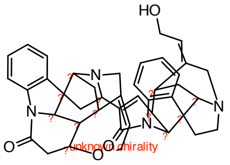	N185	N141	0.85925	0.2	0.0850000	0.115	0.81705
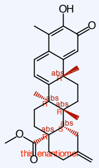	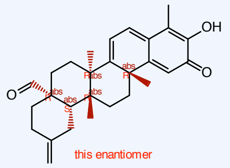	N119	N148	0.91799	0.0270000	0.0939999	0.067	0.81696
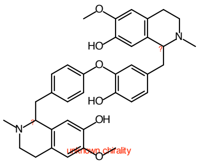	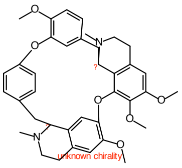	N171	N207	0.87139	0.16	0.2649538	0.10495	0.81603

Structure 1	Structure 2	ID 1	ID 2	Similarity	Activity 1	Activity 2	Delta Activity	SALI
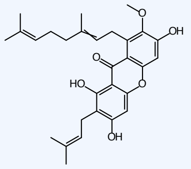	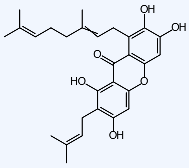	N743	N746	0.96116	6.2685881	6.3000002	3.1412e-2	0.8088
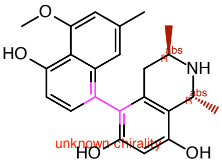	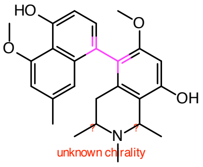	N254	N230	0.83484	0.47437215	0.34355655	0.13082	0.79204
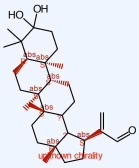	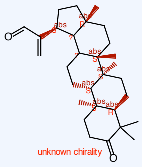	N550	N548	0.91556	3.5999999	3.533287	6.6713e-2	0.79007
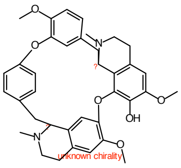	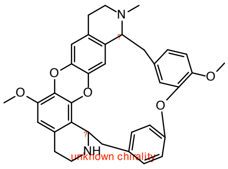	N142	N177	0.88696	0.08542469	0.17239699	8.6972e-2	0.76937
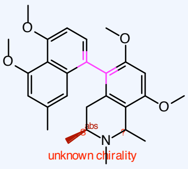	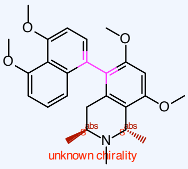	N605	N616	0.86791	4.4000001	4.5	0.1	0.75705
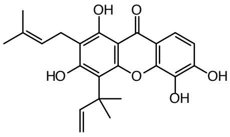	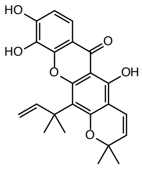	N344	N353	0.86493	1.1	1.2	0.1	0.74035
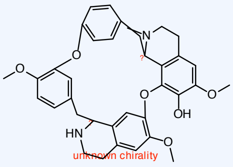	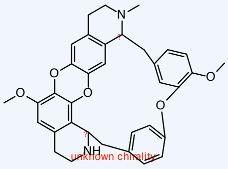	N154	N177	0.90455	0.10593635	0.17239699	6.6461e-2	0.6963
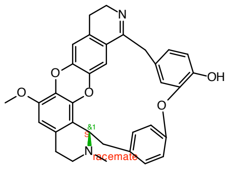	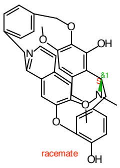	N412	N392	0.80024	1.63	1.5	0.13	0.65078
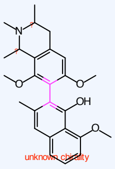	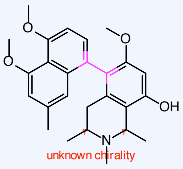	N196	N235	0.81639	0.23723181	0.35584772	0.11862	0.64602
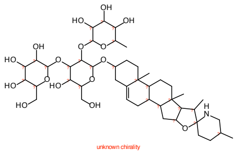	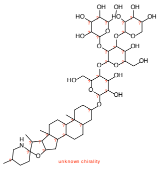	N272	N290	0.87638	0.5747	0.65249997	0.0778	0.62933
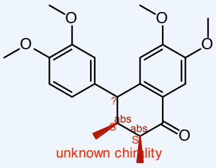	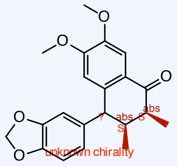	N223	N205	0.90377	0.31999999	0.25999999	0.06	0.62353

Structure 1	Structure 2	ID 1	ID 2	Similarity	Activity 1	Activity 2	Delta Activity	SALI
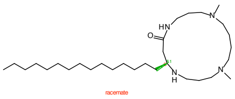	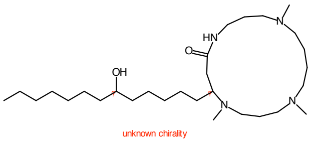	N186	N214	0.85377	0.2	0.28999999	0.09	0.61548
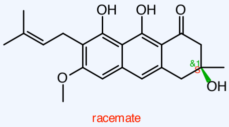	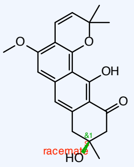	N394	N409	0.83744	1.5	1.6	0.1	0.61515
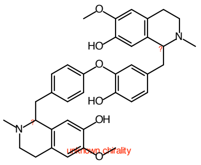	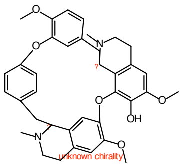	N171	N142	0.87546	0.16	0.08542469	7.4575e-2	0.59883
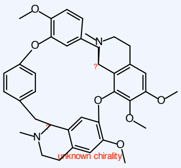	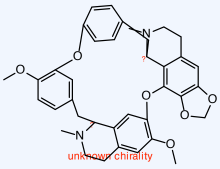	N207	N194	0.94221	0.26495388	0.23075373	0.0342	0.59178
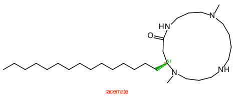	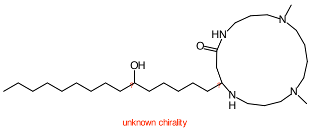	N199	N226	0.84618	0.23999999	0.33000001	0.09	0.5851
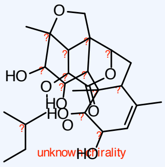	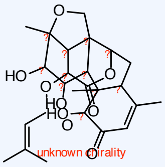	N91	N153	0.83921	0.00624287	0.10010153	9.3859e-2	0.58373
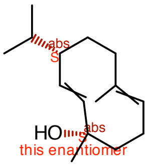	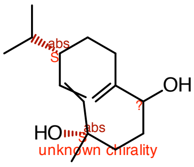	N399	N411	0.842	1.54	1.63	0.09	0.56961
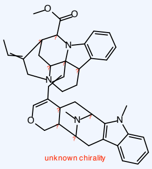	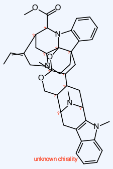	N238	N209	0.84174	0.36000001	0.27000001	0.09	0.56867
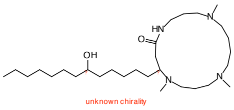	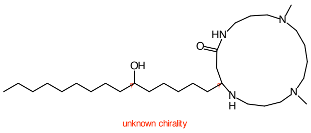	N214	N226	0.9291	0.28999999	0.33000001	0.04	0.56415
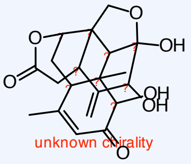	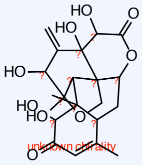	N94	N150	0.83798	0.00797023	0.09794339	8.9973e-2	0.55531
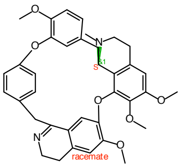	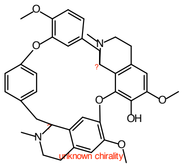	N175	N142	0.84672	0.17	0.08542469	8.4575e-2	0.55175

Structure 1	Structure 2	ID 1	ID 2	Similarity	Activity 1	Activity 2	Delta Activity	SALI
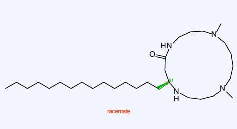	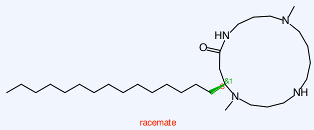	N186	N199	0.92401	0.2	0.2399999	0.04	0.52636
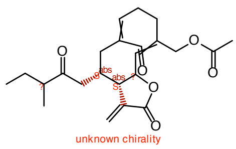	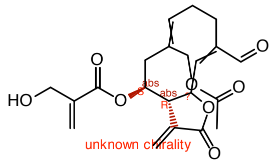	N491	N505	0.80409	2.9000001	3	0.1	0.51044
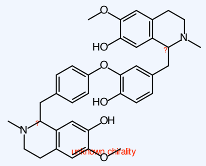	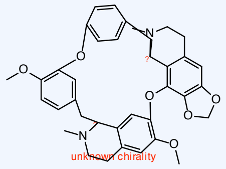	N171	N194	0.85749	0.16	0.2307537	7.0754e-2	0.49648
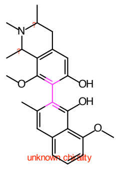	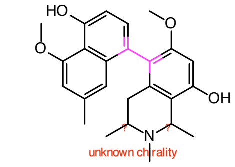	N200	N230	0.7988	0.2453975	0.3435565	9.8159e-2	0.48787
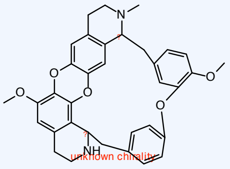	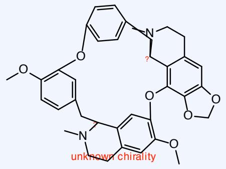	N177	N194	0.88021	0.1723969	0.2307537	5.8357e-2	0.48718
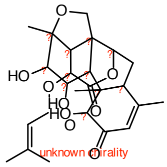	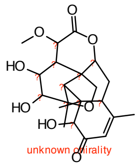	N153	N106	0.82157	0.1001015	0.015	8.5102e-2	0.47694
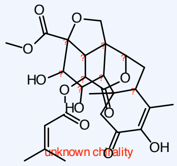	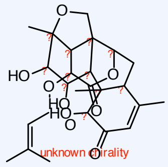	N90	N153	0.79899	0.0057634	0.1001015	9.4338e-2	0.46932
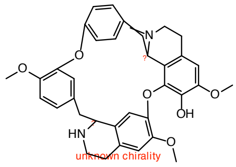	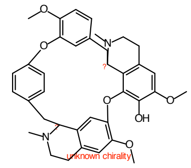	N154	N142	0.95595	0.1059363	0.0854246	2.0512e-2	0.4656
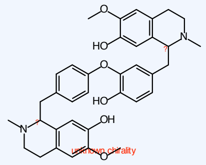	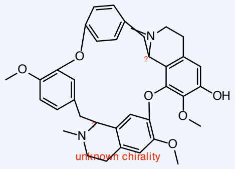	N171	N191	0.88211	0.16	0.2135617	5.3562e-2	0.45436
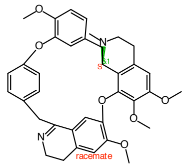	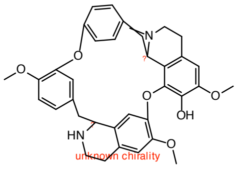	N175	N154	0.85294	0.17	0.1059363	6.4064e-2	0.43564
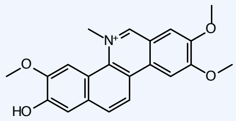	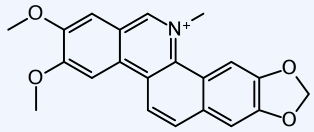	N129	N157	0.85242	0.0513716	0.1148198	6.3448e-2	0.42992

Structure 1	Structure 2	ID 1	ID 2	Similarity	Activity 1	Activity 2	Delta Activity	SALI
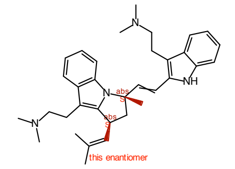	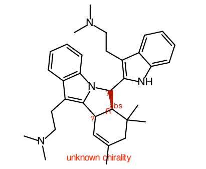	N145	N112	0.82906	0.0900000(	0.02	0.07	0.40951
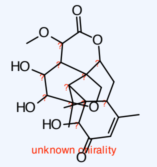	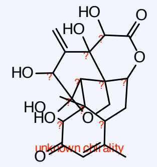	N106	N150	0.79719	0.015	0.09794339	8.2943e-2	0.40897
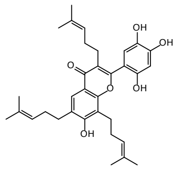	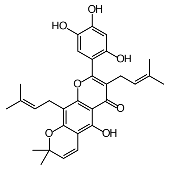	N348	N340	0.79928	1.12	1.04	0.08	0.39857
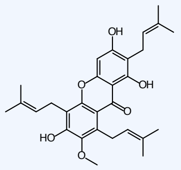	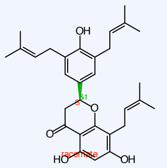	N675	N676	0.80862	5.2238231	5.3000002	7.6177e-2	0.39804
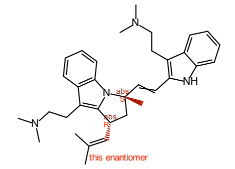	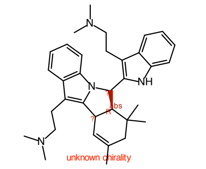	N146	N112	0.82373	0.0900000(	0.02	0.07	0.39712
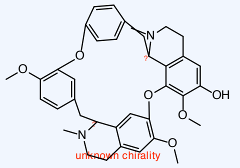	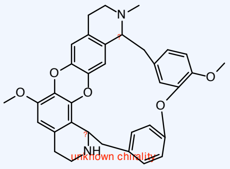	N191	N177	0.89142	0.2135617:	0.1723969	4.1165e-2	0.37911
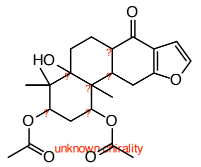	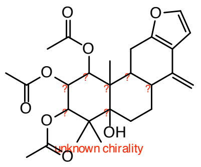	N204	N184	0.83885	0.2599999	0.2	0.06	0.37232
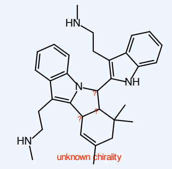	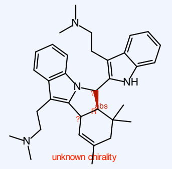	N147	N112	0.81079	0.0900000(	0.02	0.07	0.36996
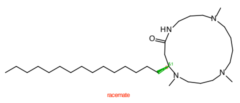	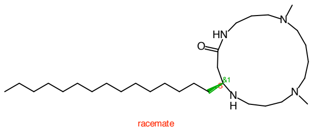	N178	N186	0.9455	0.1800000	0.2	0.02	0.36694
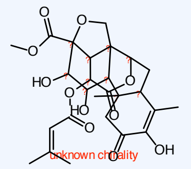	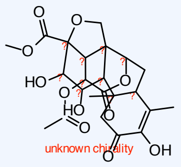	N90	N116	0.95072	0.0057634(	0.0228946	1.7131e-2	0.34763
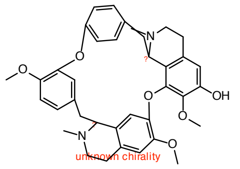	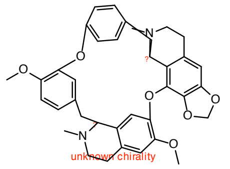	N191	N194	0.95005	0.2135617:	0.2307537:	1.7192e-2	0.34418

Structure 1	Structure 2	ID 1	ID 2	Similarity	Activity 1	Activity 2	Delta Activity	SALI
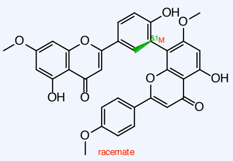	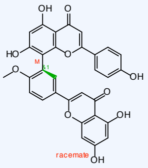	N203	N219	0.88378	0.25999999	0.30000001	0.04	0.34417
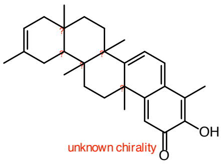	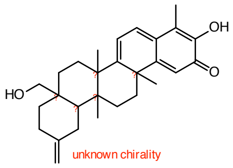	N195	N212	0.8872	0.23233728	0.27105191	3.8715e-2	0.34322
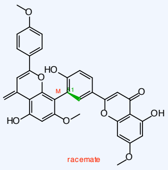	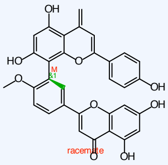	N206	N220	0.88337	0.25999999	0.30000001	0.04	0.34297
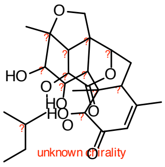	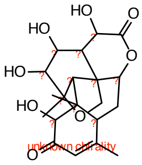	N92	N137	0.80997	0.00626917	0.07100000	6.4731e-2	0.34064
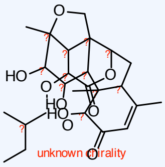	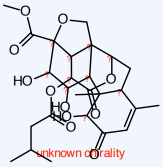	N92	N127	0.87072	0.00626917	0.05000000	4.3731e-2	0.33826
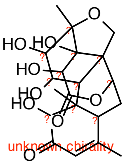	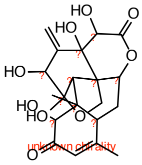	N122	N150	0.81782	0.03654836	0.09794339	6.1395e-2	0.33701
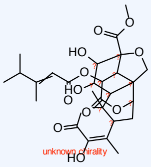	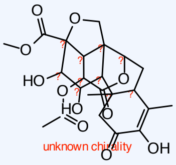	N82	N116	0.93635	0.00145831	0.02289464	2.1436e-2	0.33677
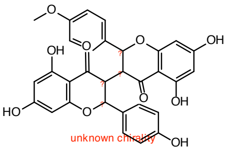	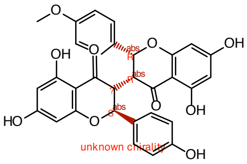	N322	N326	0.90851	0.97032207	1	2.9678e-2	0.32438
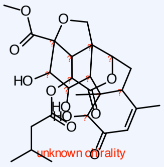	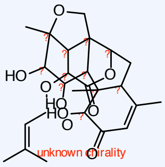	N127	N153	0.84358	0.05000000	0.10010151	5.0102e-2	0.3203
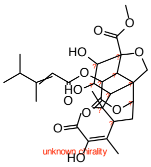	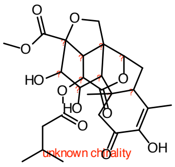	N82	N115	0.93859	0.00145831	0.02105096	1.9593e-2	0.31903
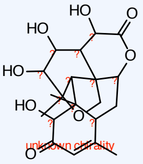	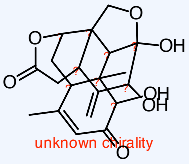	N137	N94	0.79637	0.07100000	0.00797023	0.06303	0.30953

Structure 1	Structure 2	ID 1	ID 2	Similarity	Activity 1	Activity 2	Delta Activity	SALI
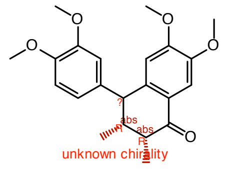	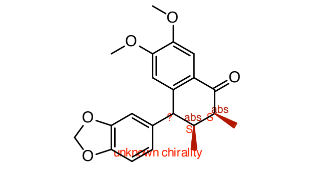	N187	N205	0.80585	0.2	0.2599999	0.06	0.30903
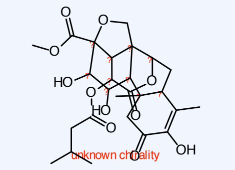	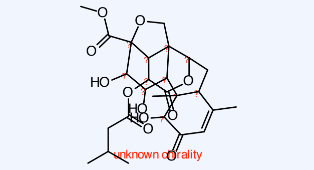	N115	N127	0.90122	0.0210509	0.0500000	2.8949e-2	0.29306
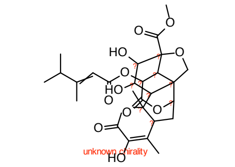	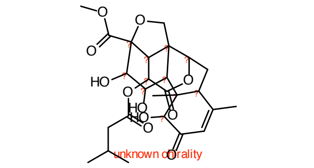	N82	N127	0.82823	0.0014583	0.0500000	4.8542e-2	0.2826
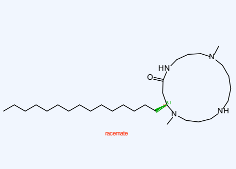	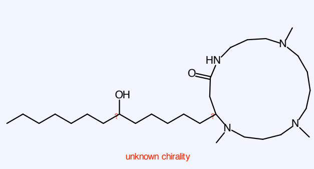	N199	N214	0.82287	0.2399999	0.2899999	0.05	0.28228
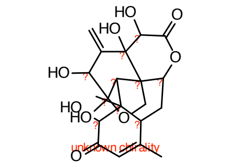	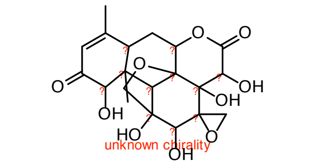	N150	N126	0.81997	0.0979433	0.0471255	5.0818e-2	0.28227
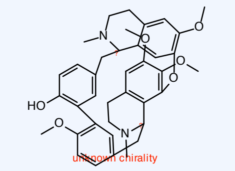	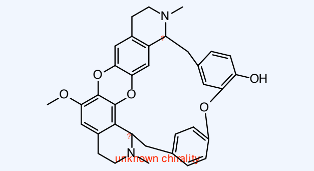	N350	N347	0.85787	1.14	1.1	0.04	0.28144
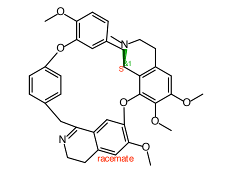	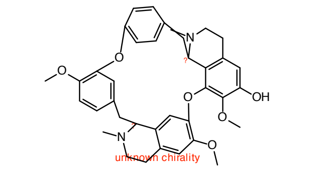	N175	N191	0.84445	0.17	0.2135617	4.3562e-2	0.28004
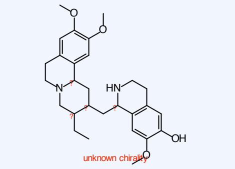	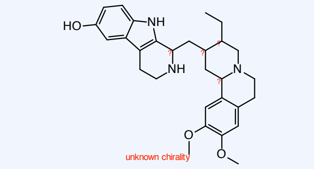	N135	N102	0.83722	0.0578638	0.0126150	4.5249e-2	0.27797
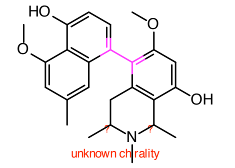	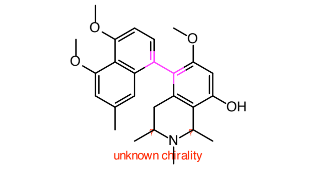	N230	N235	0.95549	0.3435565	0.3558477	1.2291e-2	0.27617
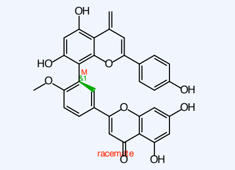	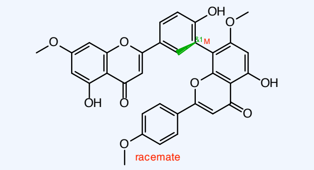	N220	N203	0.85435	0.3000000	0.2599999	0.04	0.27463
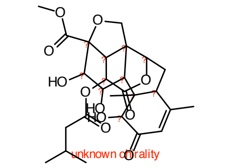	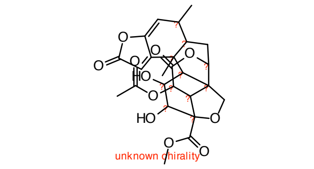	N127	N87	0.82479	0.0500000	0.0020000	0.048	0.27396

Structure 1	Structure 2	ID 1	ID 2	Similarity	Activity 1	Activity 2	Delta Activity	SALI
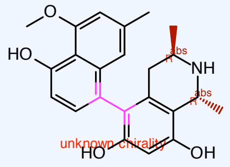	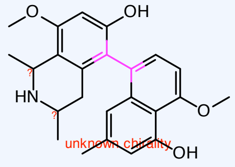	N254	N266	0.80161	0.47437215	0.52862251	0.05425	0.27345
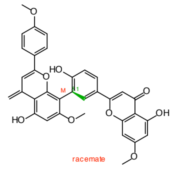	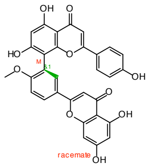	N206	N219	0.85197	0.25999999	0.30000001	0.04	0.27022
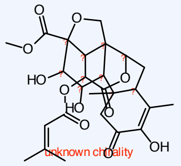	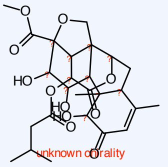	N90	N127	0.8264	0.00576340	0.05000000	4.4237e-2	0.25482
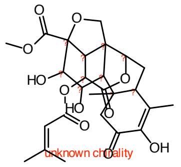	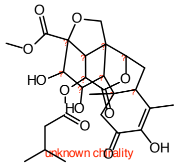	N90	N115	0.93912	0.00576340	0.02105096	1.5288e-2	0.25113
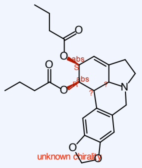	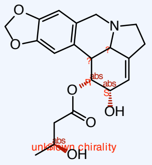	N402	N410	0.82465	1.5672873	1.6068573	0.03957	0.22566
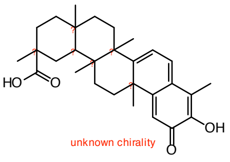	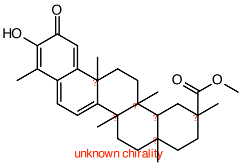	N246	N247	0.96185	0.40145615	0.40978298	8.3268e-3	0.21825
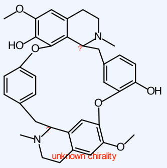	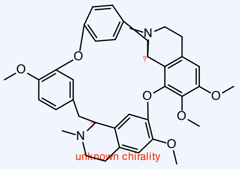	N234	N241	0.91648	0.35299999	0.37093544	1.7935e-2	0.21476
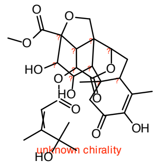	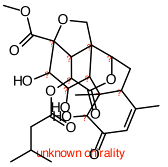	N95	N127	0.80477	0.00885616	0.05000000	4.1144e-2	0.21075
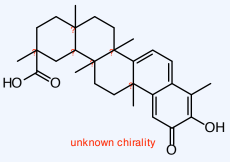	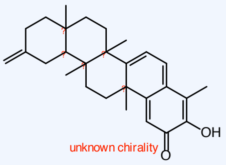	N246	N250	0.90919	0.40145615	0.42018446	1.8728e-2	0.20623
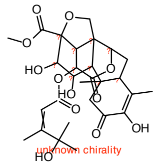	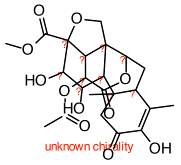	N95	N116	0.9281	0.00885616	0.02289464	1.4038e-2	0.19526
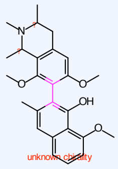	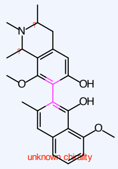	N196	N200	0.95804	0.23723181	0.24539754	8.1657e-3	0.19459

Structure 1	Structure 2	ID 1	ID 2	Similarity	Activity 1	Activity 2	Delta Activity	SALI
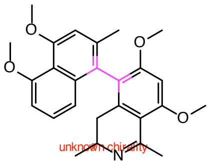	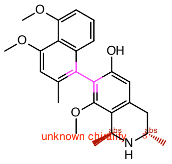	N638	N640	0.82727	4.7674356	4.8000002	3.2565e-2	0.18853
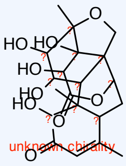	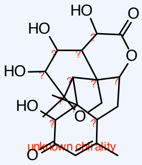	N122	N137	0.81211	0.0365483(	0.0710000(	3.4452e-2	0.18337
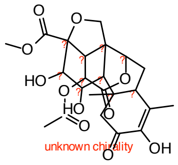	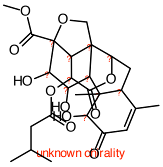	N116	N127	0.82978	0.0228946‹	0.0500000(	2.7105e-2	0.15923
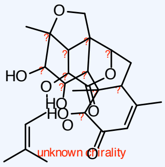	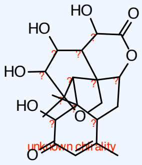	N153	N137	0.80997	0.1001015:	0.0710000(	2.9102e-2	0.15314
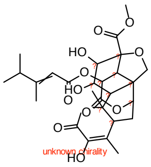	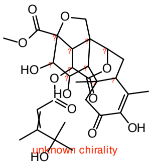	N82	N95	0.95155	0.0014583:	0.0088561(	7.3979e-3	0.1527
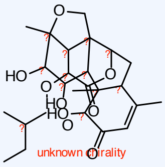	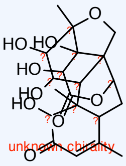	N92	N122	0.79626	0.0062691(	0.0365483(	3.0279e-2	0.14861
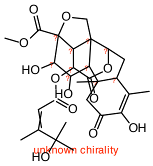	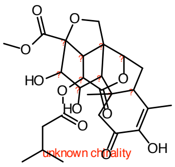	N95	N115	0.91702	0.0088561(	0.0210509(	1.2195e-2	0.14697
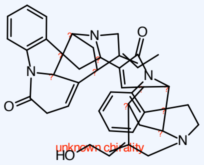	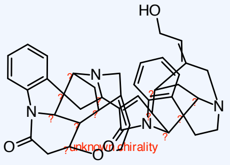	N152	N141	0.8975	0.1	0.0850000(	0.015	0.14634
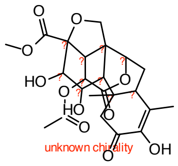	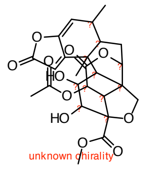	N116	N87	0.85327	0.0228946‹	0.0020000(	2.0895e-2	0.1424
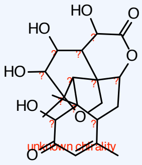	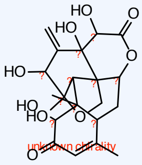	N137	N150	0.80862	0.0710000(	0.0979433:	2.6943e-2	0.14078
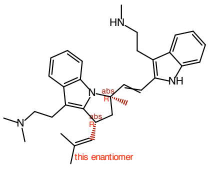	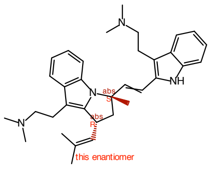	N140	N146	0.92307	0.0799999:	0.0900000(	0.01	0.12999

Structure 1	Structure 2	ID 1	ID 2	Similarity	Activity 1	Activity 2	Delta Activity	SALI
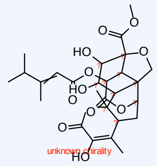	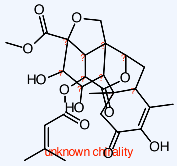	N82	N90	0.96575	0.00145831	0.0057634(	4.3051e-3	0.12569
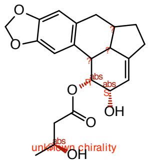	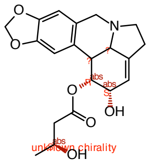	N405	N410	0.94302	1.6	1.6068573	6.8573e-3	0.12035
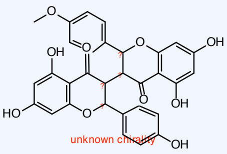	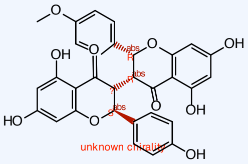	N322	N320	0.90851	0.97032207	0.95999998	1.0322e-2	0.11282
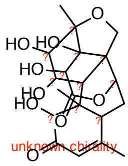	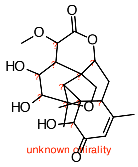	N122	N106	0.80409	0.0365483(	0.015	2.1548e-2	0.10999
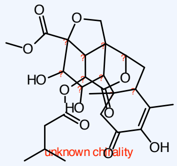	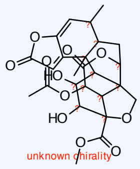	N115	N87	0.81665	0.0210509(	0.0020000(	1.9051e-2	0.10391
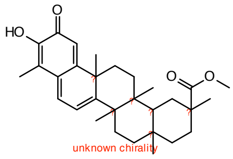	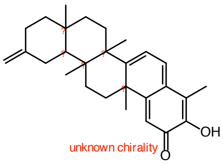	N247	N250	0.89662	0.40978298	0.4201844(	1.0401e-2	0.10061
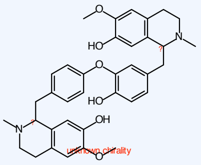	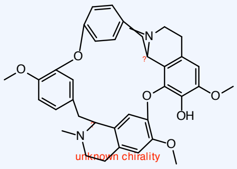	N171	N176	0.87546	0.16	0.1718350!	1.1835e-2	9.5034e-2
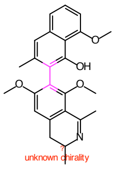	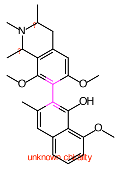	N201	N196	0.89932	0.2466175:	0.2372318!	9.3857e-3	0.09322
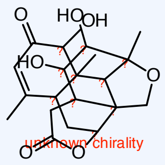	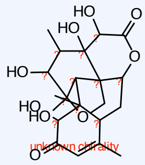	N190	N193	0.79546	0.2099999!	0.2265998!	0.0166	8.1157e-2
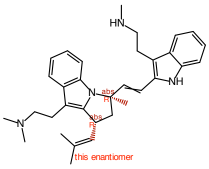	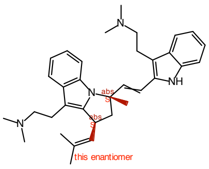	N140	N145	0.8757	0.0799999!	0.0900000(	0.01	8.0454e-2
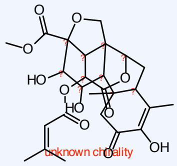	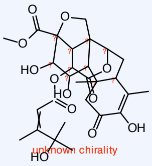	N90	N95	0.95843	0.0057634(	0.0088561(	3.0928e-3	7.4404e-2

Structure 1	Structure 2	ID 1	ID 2	Similarity	Activity 1	Activity 2	Delta Activity	SALI
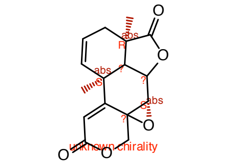	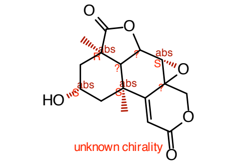	N832	N827	0.84268	8.0100002	8	0.01	6.3568e-2
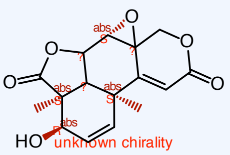	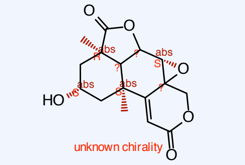	N829	N827	0.81362	8.0100002	8	0.01	5.3656e-2
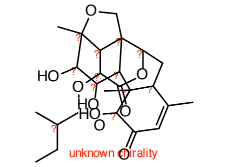	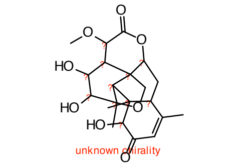	N92	N106	0.83096	0.0062691	0.015	8.7308e-3	5.1651e-2
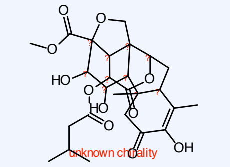	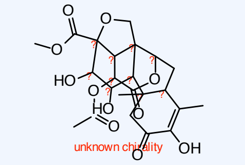	N115	N116	0.95517	0.0210509	0.0228946	1.8437e-3	4.1126e-2
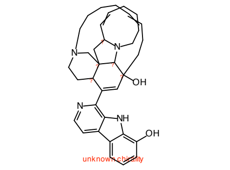	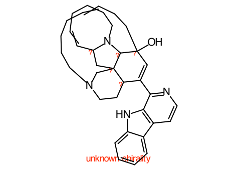	N97	N101	0.953	0.0106239	0.0125	1.876e-3	3.9918e-2
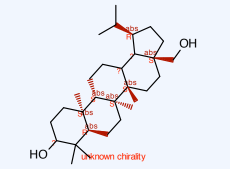	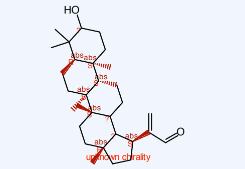	N787	N789	0.86606	7.1953335	7.1999998	4.6663e-3	3.4839e-2
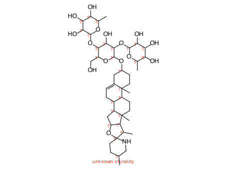	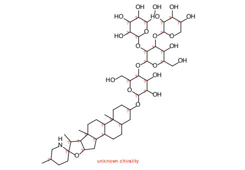	N287	N290	0.83073	0.6488999	0.6524999	0.0036	2.1268e-2
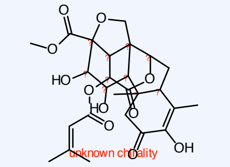	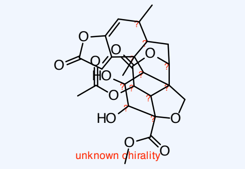	N90	N87	0.80026	0.0057634	0.0020000	3.7634e-3	1.8842e-2
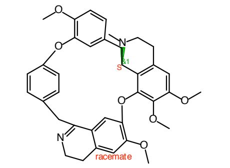	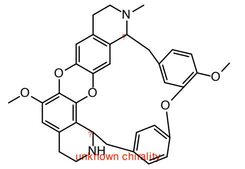	N175	N177	0.834	0.17	0.1723969	2.397e-3	1.4439e-2
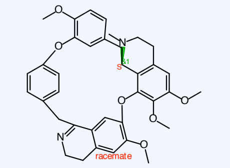	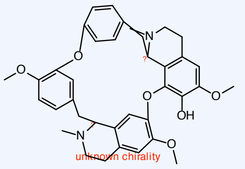	N175	N176	0.84672	0.17	0.1718350	1.835e-3	1.1972e-2
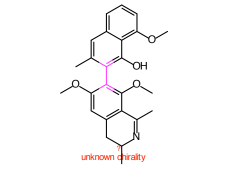	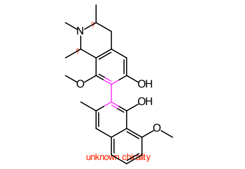	N201	N200	0.8417	0.2466175	0.2453975	0.00122	7.7068e-3

Structure 1	Structure 2	ID 1	ID 2	Similarity	Activity 1	Activity 2	Delta Activity	SALI
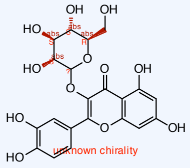	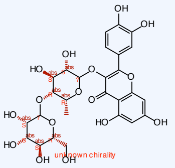	N88	N89	0.91407	0.0023000(	0.0029	0.0006	6.9824e-3
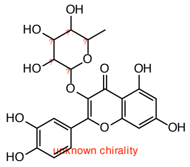	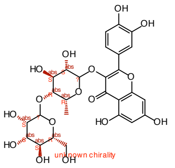	N86	N89	0.83235	0.0020000(	0.0029	0.0009	5.3684e-3
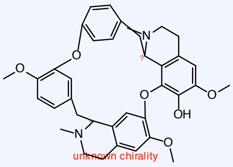	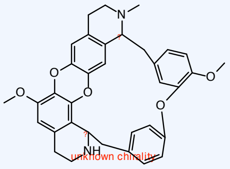	N176	N177	0.88919	0.1718350!	0.1723969!	5.6194e-4	5.0712e-3
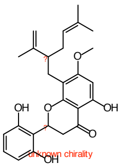	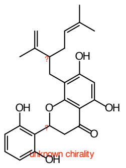	N96	N98	0.95739	0.0106	0.0108	0.0002	4.6942e-3
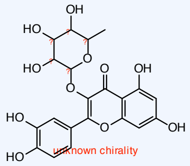	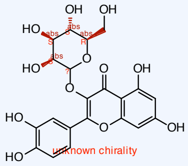	N86	N88	0.91282	0.0020000(	0.0023000(	0.0003	3.4412e-3
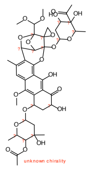	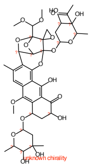	N83	N84	0.96926	0.0018247	0.0019165!	9.1883e-5	2.9889e-3
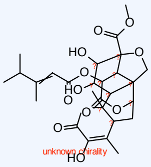	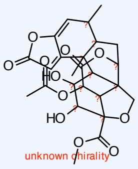	N82	N87	0.79733	0.0014583	0.0020000(	5.4169e-4	2.6727e-3
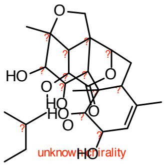	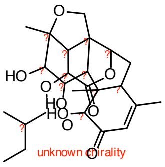	N91	N92	0.93188	0.0062428	0.0062691	2.6299e-5	3.8607e-4
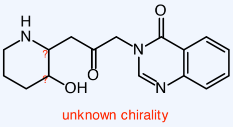	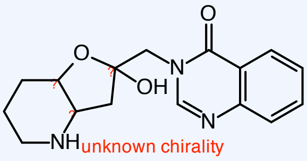	N78	N80	0.8091	0.0000007	0.0000034	2.7e-6	1.4143e-5
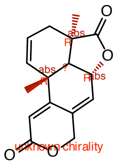	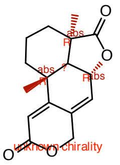	N833	N834	0.84007	8.0100002	8.0100002	0	0
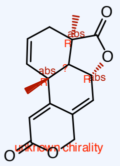	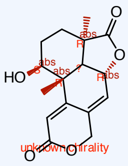	N833	N831	0.82822	8.0100002	8.0100002	0	0

Structure 1	Structure 2	ID 1	ID 2	Similarity	Activity 1	Activity 2	Delta Activity	SALI
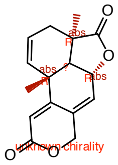	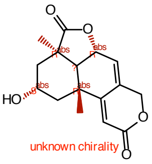	N833	N828	0.8234	8.0100002	8.0100002	0	0
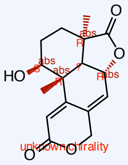	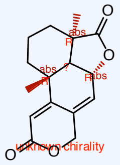	N831	N834	0.86146	8.0100002	8.0100002	0	0
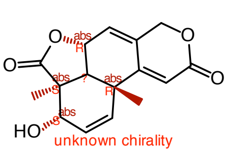	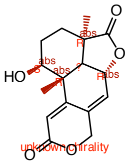	N830	N831	0.80261	8.0100002	8.0100002	0	0
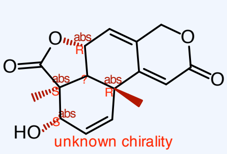	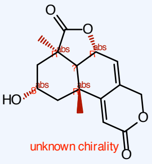	N830	N828	0.79785	8.0100002	8.0100002	0	0
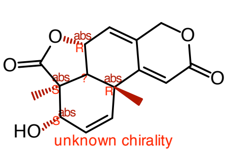	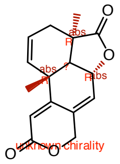	N830	N833	0.86146	8.0100002	8.0100002	0	0
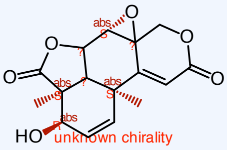	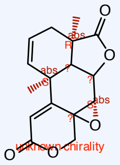	N829	N832	0.87401	8.0100002	8.0100002	0	0
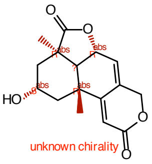	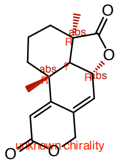	N828	N834	0.88459	8.0100002	8.0100002	0	0
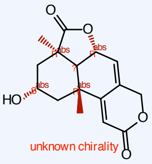	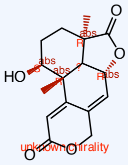	N828	N831	0.85419	8.0100002	8.0100002	0	0
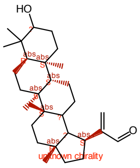	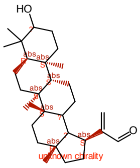	N789	N925	1	7.1999998	10.596756	3.3968	0
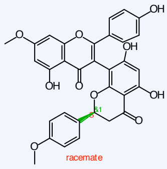	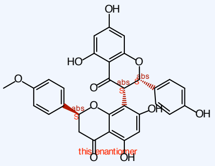	N733	N734	0.81885	6	6	0	0
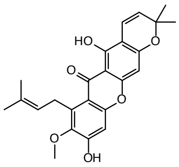	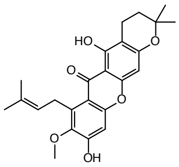	N731	N732	0.90474	6	6	0	0

Structure 1	Structure 2	ID 1	ID 2	Similarity	Activity 1	Activity 2	Delta Activity	SALI
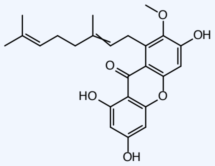	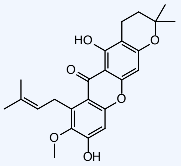	N729	N732	0.82533	6	6	0	0
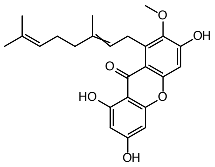	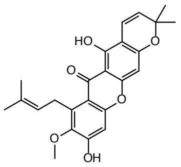	N729	N731	0.82204	6	6	0	0
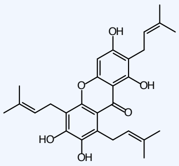	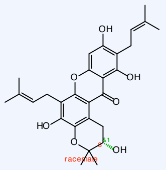	N722	N718	0.89051	6	6	0	0
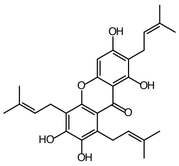	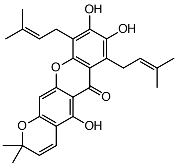	N722	N719	0.8898	6	6	0	0
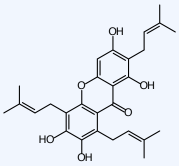	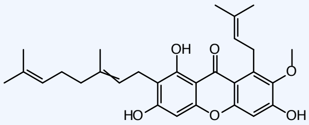	N722	N720	0.84181	6	6	0	0
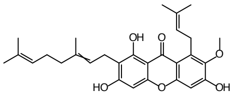	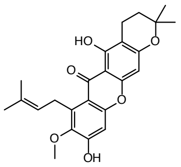	N720	N732	0.84161	6	6	0	0
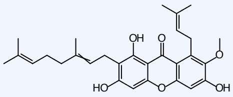	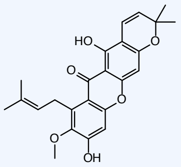	N720	N731	0.82648	6	6	0	0
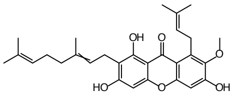	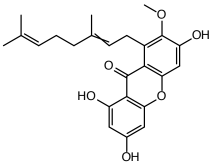	N720	N729	0.88872	6	6	0	0
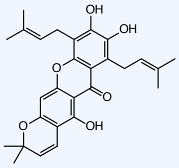	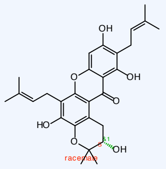	N719	N718	0.84636	6	6	0	0
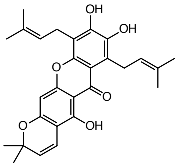	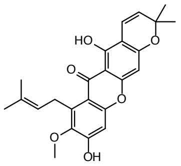	N719	N731	0.85102	6	6	0	0
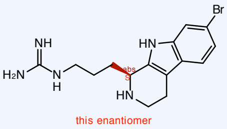	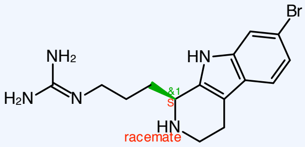	N679	N18	0.79785	5.4000001	5.4000001	0	0

Structure 1	Structure 2	ID 1	ID 2	Similarity	Activity 1	Activity 2	Delta Activity	SALI
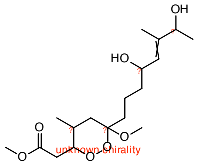	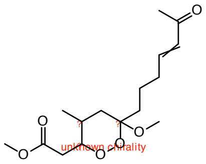	N659	N660	0.83037	5	5	0	0
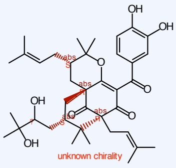	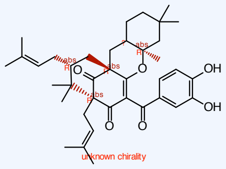	N656	N652	0.88638	5	5	0	0
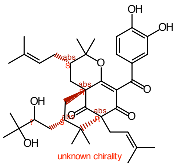	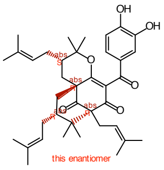	N656	N651	0.93839	5	5	0	0
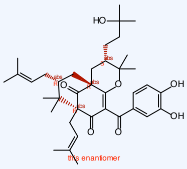	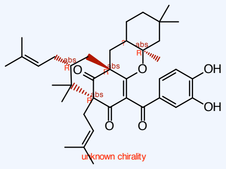	N655	N652	0.91923	5	5	0	0
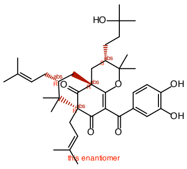	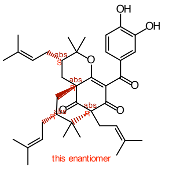	N655	N651	0.94534	5	5	0	0
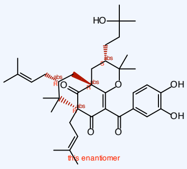	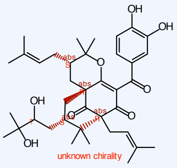	N655	N656	0.93923	5	5	0	0
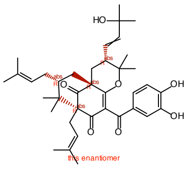	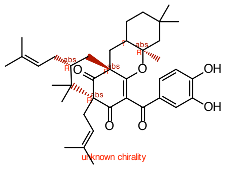	N654	N652	0.89797	5	5	0	0
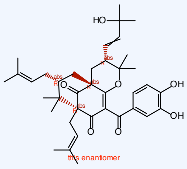	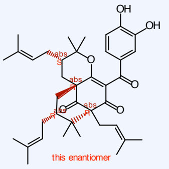	N654	N651	0.92747	5	5	0	0
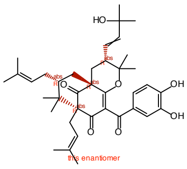	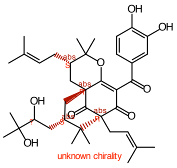	N654	N656	0.91937	5	5	0	0
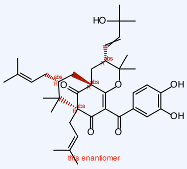	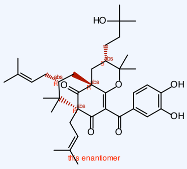	N654	N655	0.94033	5	5	0	0
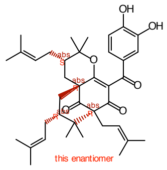	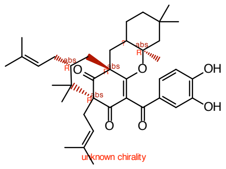	N651	N652	0.90528	5	5	0	0

Structure 1	Structure 2	ID 1	ID 2	Similarity	Activity 1	Activity 2	Delta Activity	SALI
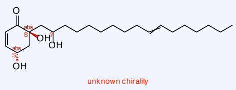	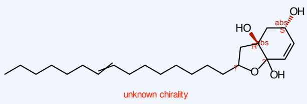	N634	N635	0.85572	4.6999998	4.6999998	0	0
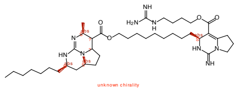	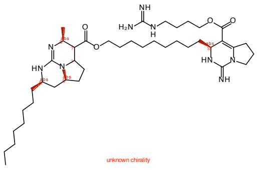	N618	N619	0.99845	4.5	4.5	0	0
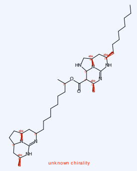	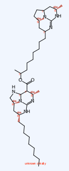	N610	N611	0.99814	4.5	4.5	0	0
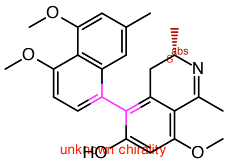	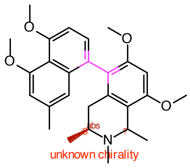	N604	N605	0.83865	4.4000001	4.4000001	0	0
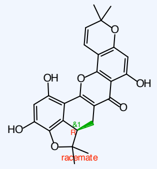	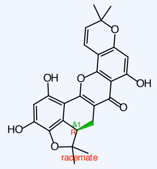	N562	N847	1	3.7	8.5167503	4.8168	0
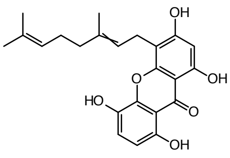	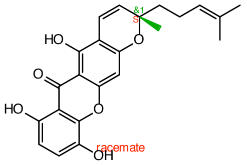	N508	N509	0.80064	3	3	0	0
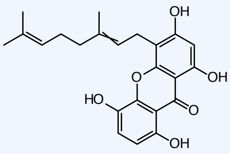	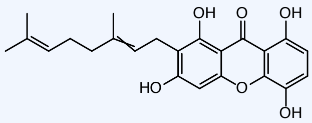	N508	N504	0.94823	3	3	0	0
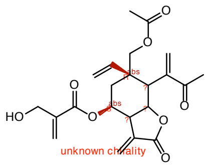	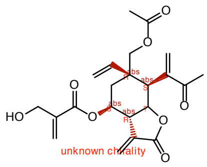	N506	N500	0.89253	3	3	0	0
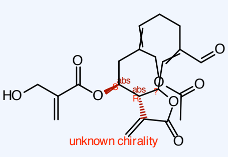	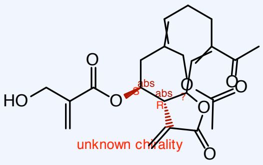	N505	N507	0.9397	3	3	0	0
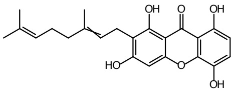	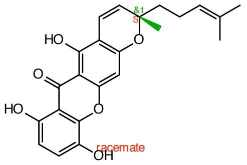	N504	N509	0.85165	3	3	0	0
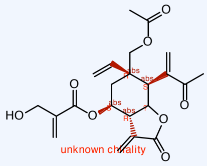	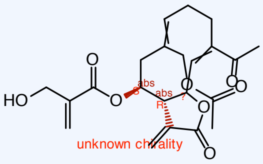	N500	N507	0.81211	3	3	0	0

Structure 1	Structure 2	ID 1	ID 2	Similarity	Activity 1	Activity 2	Delta Activity	SALI
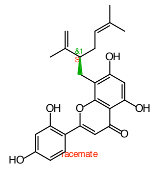	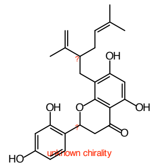	N483	N482	0.85745	2.5999999	2.5999999	0	0
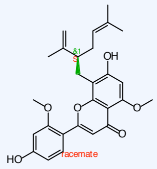	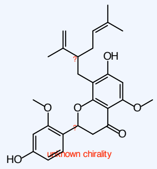	N470	N469	0.86832	2.4000001	2.4000001	0	0
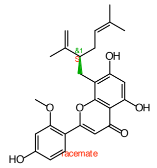	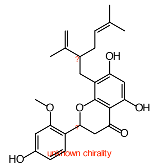	N452	N451	0.86307	2.0999999	2.0999999	0	0
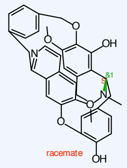	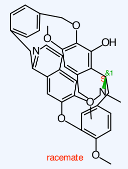	N392	N393	0.97312	1.5	1.5	0	0
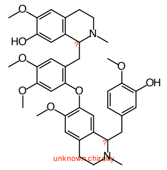	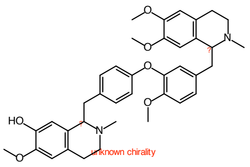	N388	N389	0.90257	1.5	1.5	0	0
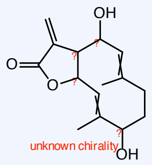	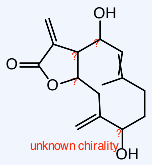	N385	N386	0.8703	1.5	1.5	0	0
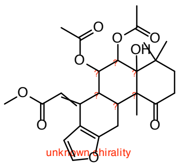	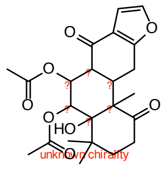	N327	N328	0.91048	1	1	0	0
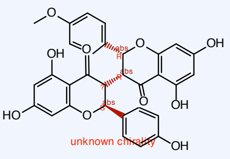	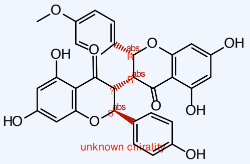	N320	N326	1	0.9599999[illegible]	1	0.04	0
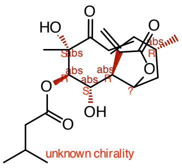	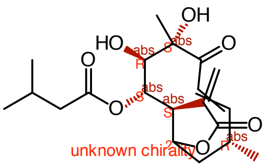	N279	N278	0.93686	0.62	0.62	0	0
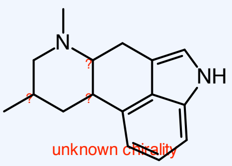	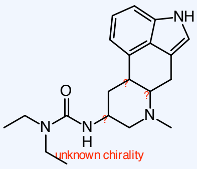	N259	N261	0.79663	0.5	0.5	0	0
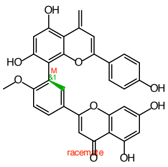	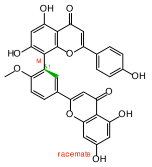	N220	N219	0.98566	0.3000000[illegible]	0.3000000[illegible]	0	0

Structure 1	Structure 2	ID 1	ID 2	Similarity	Activity 1	Activity 2	Delta Activity	SALI
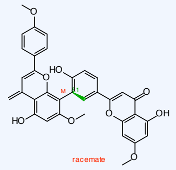	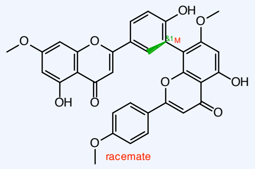	N206	N203	0.9864	0.25999999	0.25999999	0	0
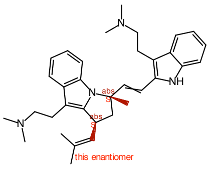	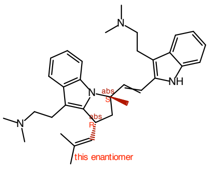	N145	N146	0.95656	0.09000000	0.09000000	0	0
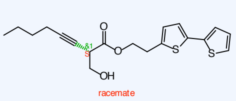	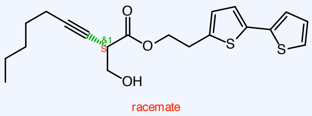	N131	N132	0.98264	0.05299999	0.05299999	0	0
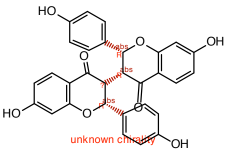	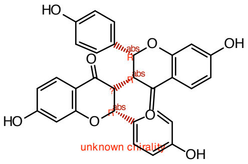	N13	N169	1	0.15671191	0.15700001	2.881e-4	0
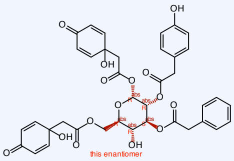	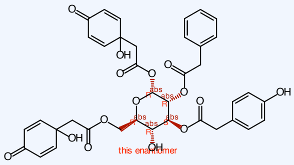	N305	N304	1	0.80000001	0.80000001	0	NaN
