# Supplementary figures and images for: Prioritization of anti-malarial hits from nature: chemo-informatic profiling of natural products with in vitro antiplasmodial activities and currently registered anti-malarial drugs
Source: Malar J. 2016 Jan 29;15:50. doi: 10.1186/s12936-016-1087-y (PMC4731946; doi:10.1186/s12936-016-1087-y)

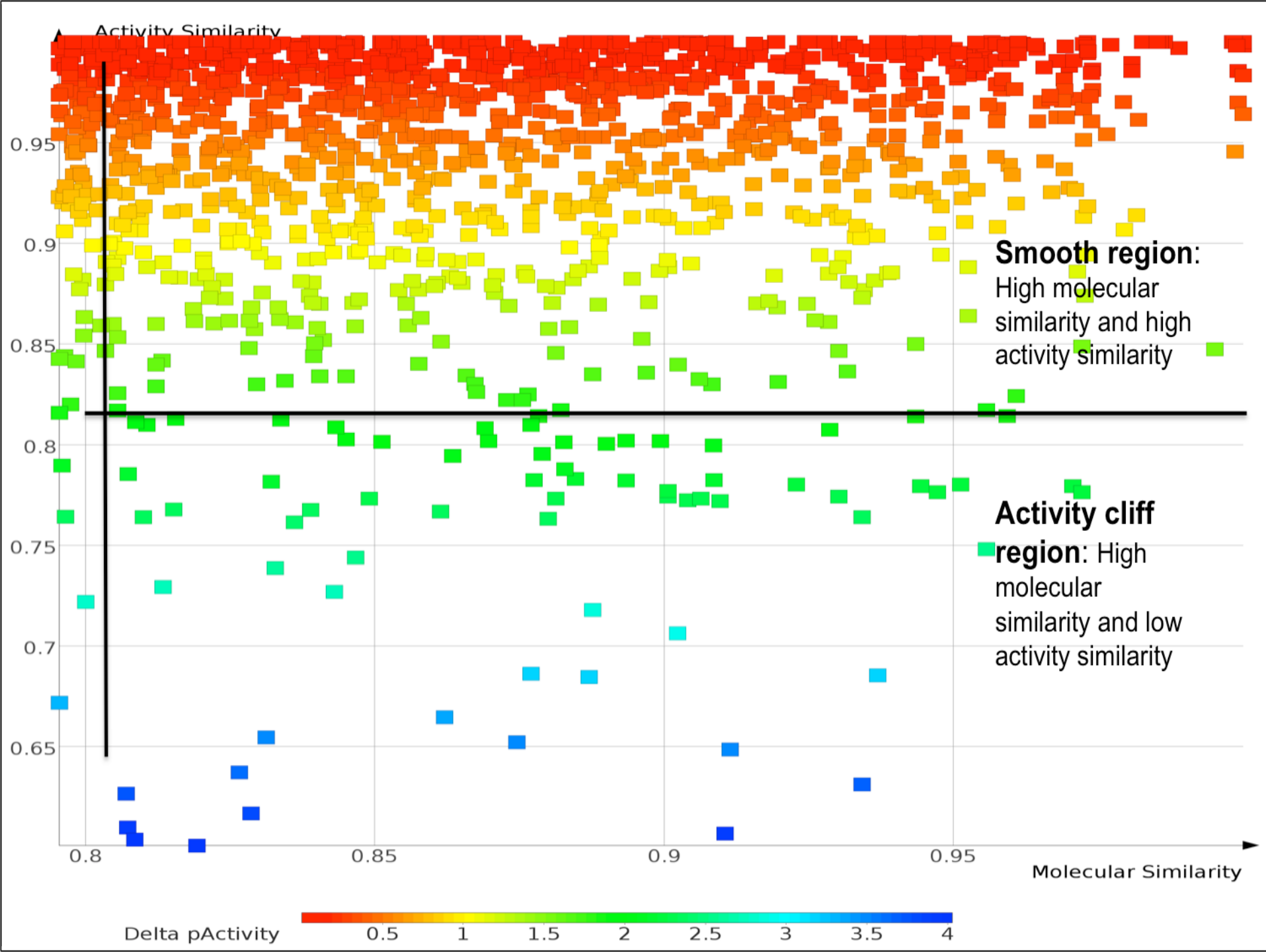

Supplement: Supplementary file 5 — 10.1186/s12936-016-1087-y Scatter plot of activity similarity and molecular similarity of natural products with in vitro antiplasmodial activities. Markers are coloured by change in activity (Delta pActivity). Activity cliff region is bounded by activity similarity below 0.8 and molecular similarity above 0.8. [file 12936_2016_1087_MOESM5_ESM.pdf]

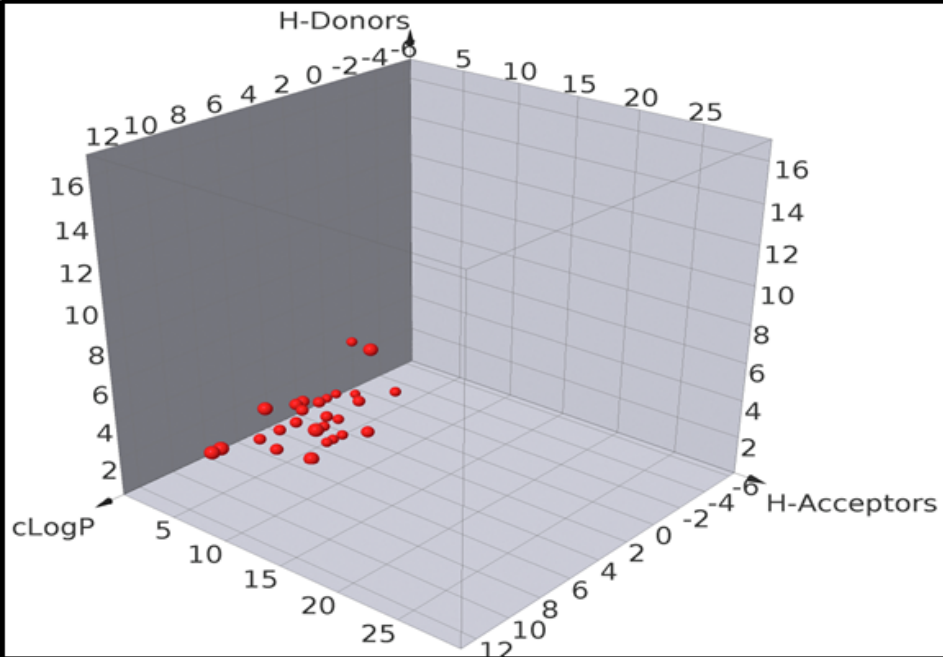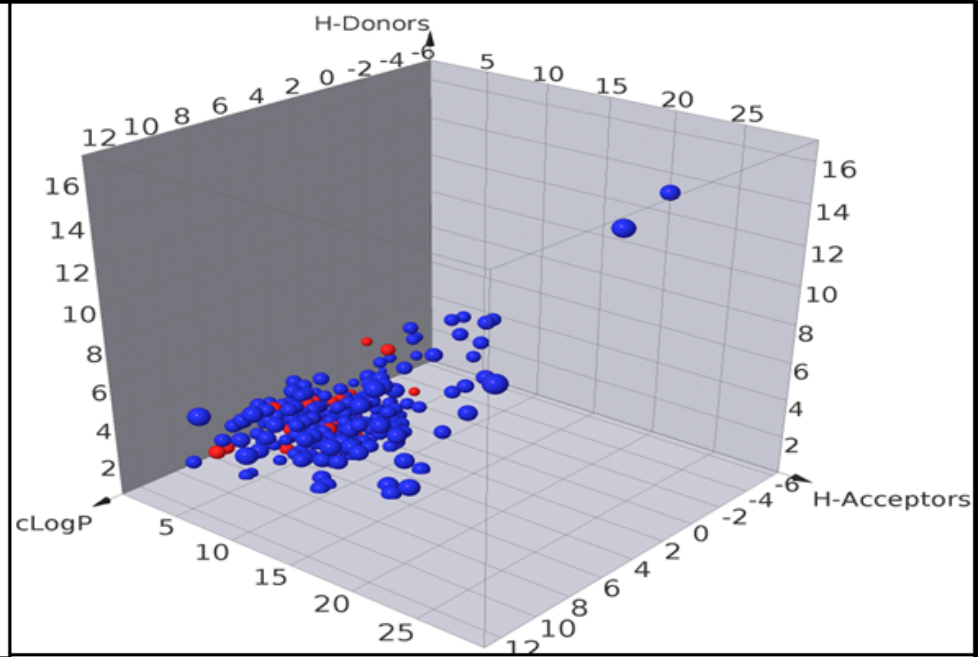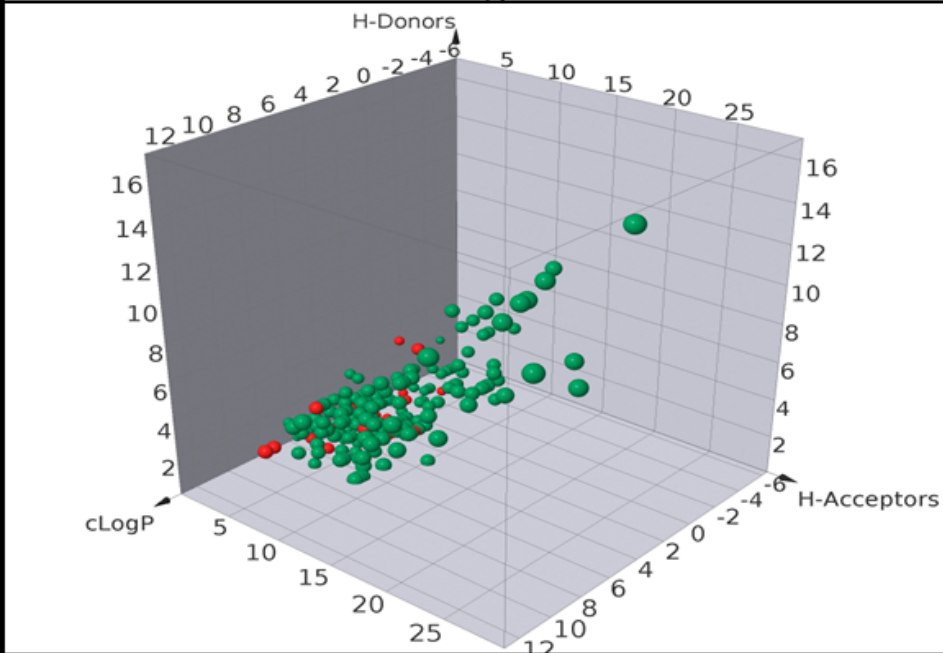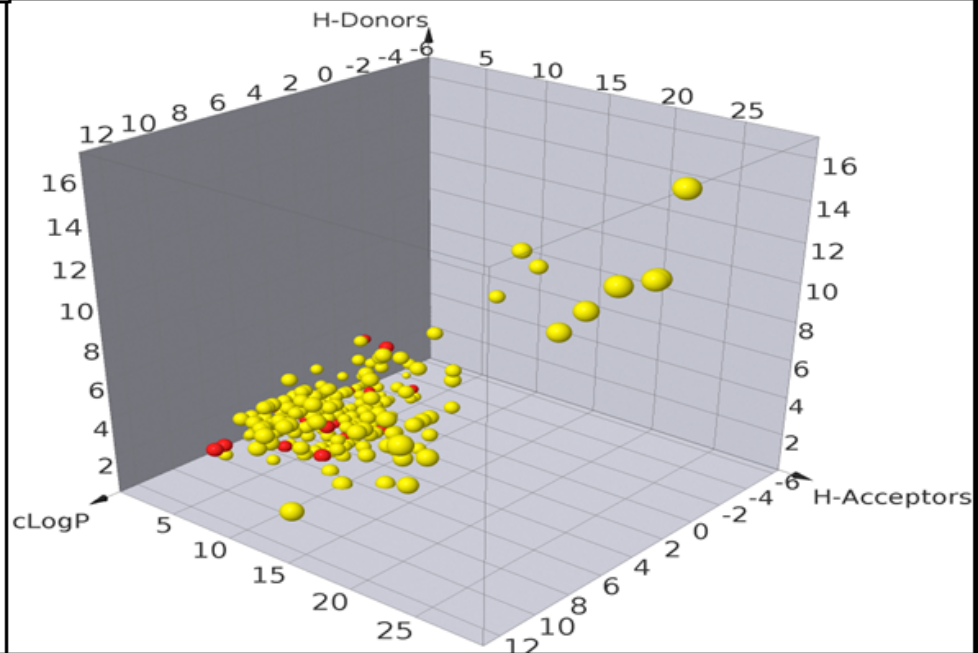

Total Molweight    • 200    • 400    • 600    • 800    • 1000    • 1200

Supplement: Supplementary file 6 — 10.1186/s12936-016-1087-y Four-dimensional plot of Lipinski’s rule of five for compound sets. NAA relative to CRAD. [file 12936_2016_1087_MOESM6_ESM.pdf]

A

CRAD

HA

MA

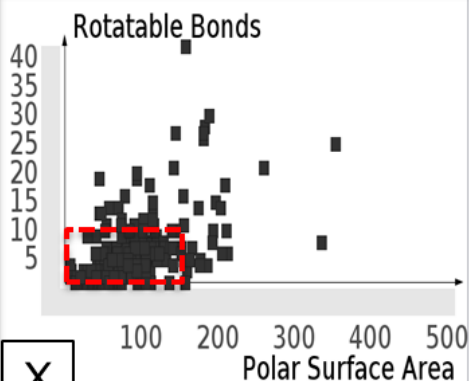

X

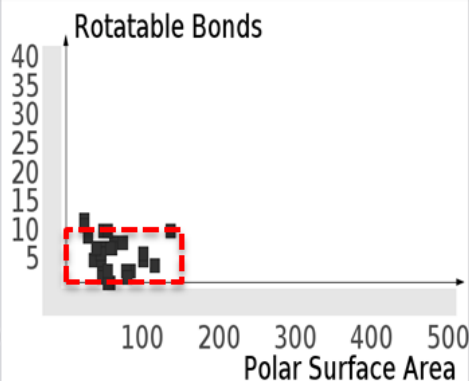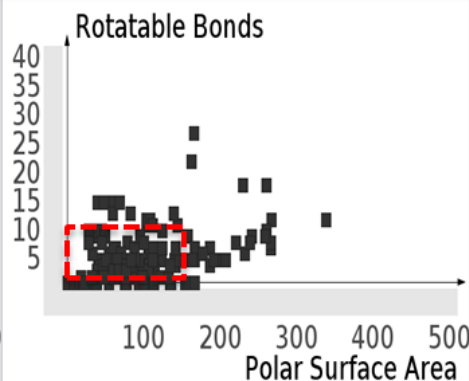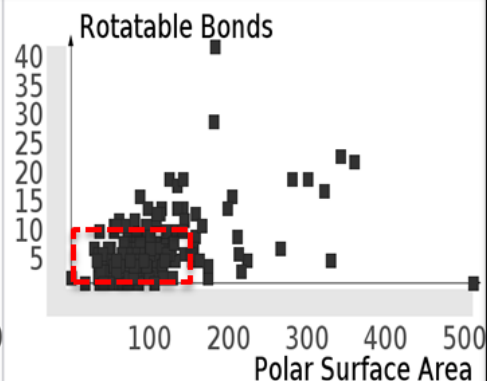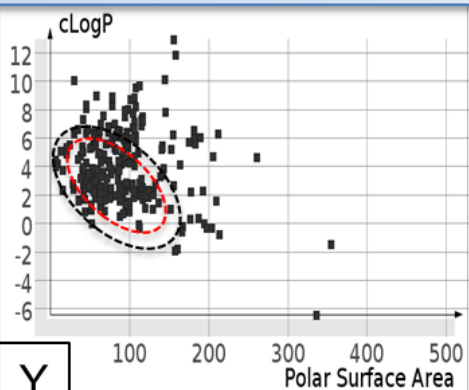

Y

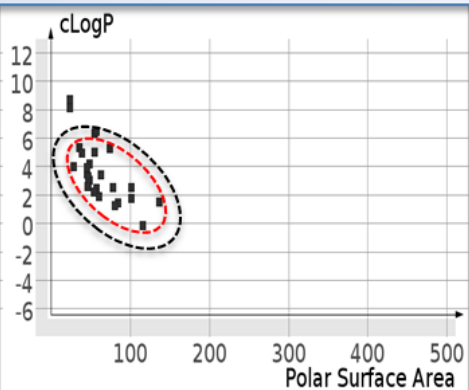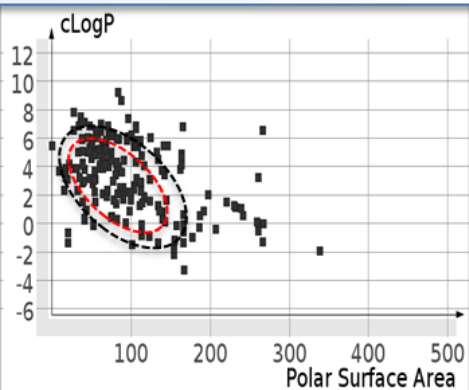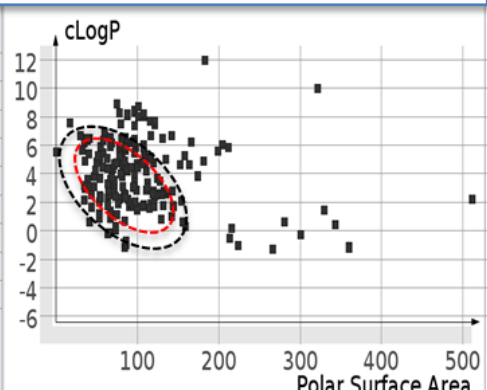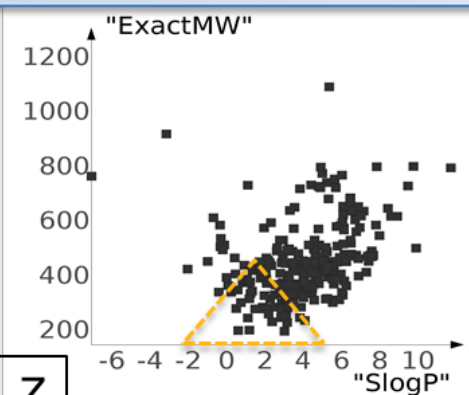

Z

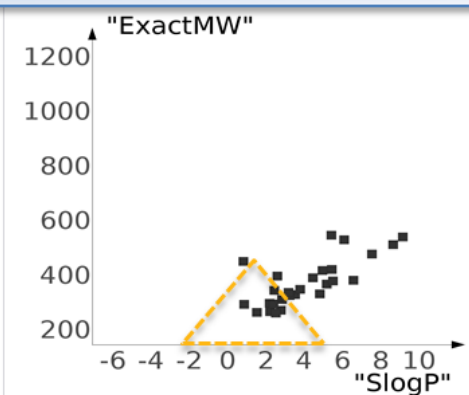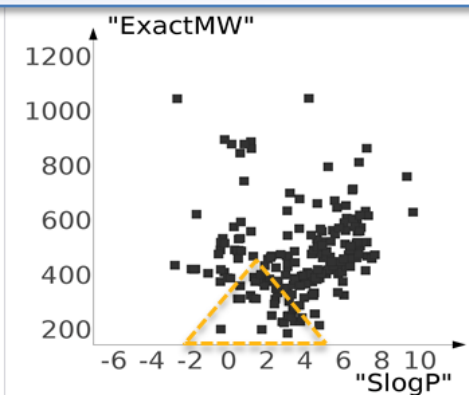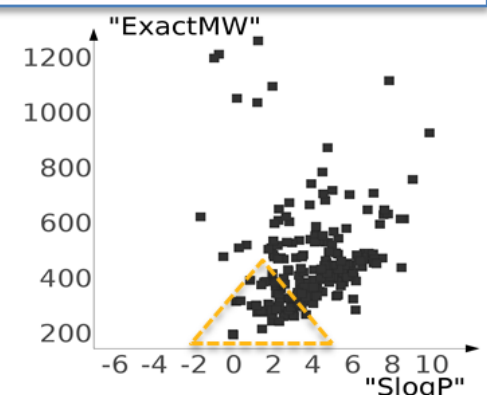

Supplement: Supplementary file 7 — 10.1186/s12936-016-1087-y Pharmacokinetic models depicting the proportion of compounds that fall within desired regions of good bioavailability. The models shown include: model by Veber et al. (X), Egan Egg model (Y) and golden triangle model (Z). The models were applied on the sub-groups of NAA (A, HA and MA) and CRAD. Marked regions encompass compounds that fall within desired regions and that may possess good bioavailability. [file 12936_2016_1087_MOESM7_ESM.pdf]
