# Supplementary material for: Prioritization of anti-malarial hits from nature: chemo-informatic profiling of natural products with in vitro antiplasmodial activities and currently registered anti-malarial drugs
Source: Malar J. 2016 Jan 29;15:50. doi: 10.1186/s12936-016-1087-y (PMC4731946; doi:10.1186/s12936-016-1087-y)
Supplement: Supplementary file 8 — 10.1186/s12936-016-1087-y Prioritized list of natural products with in vitro antiplasmodial activities. Columns include the structure of the compounds, the identity of the compounds (ID), activity_status (A, HA,MA or CRAD) and the consensus score. List was sorted by the activity status. [file 12936_2016_1087_MOESM8_ESM.pdf]

Structure of Smiles	ID	Activity_Status	Consensus_score
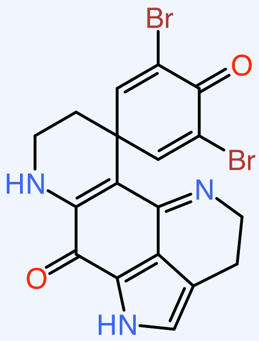	N437	A	0.75
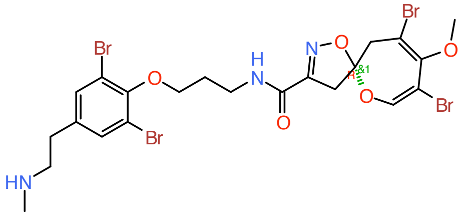	N377	A	0.25
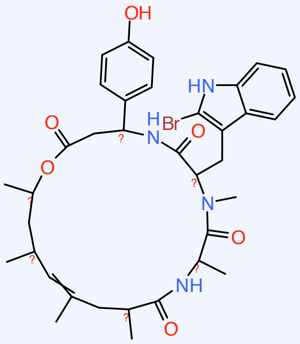	N379	A	-0.25
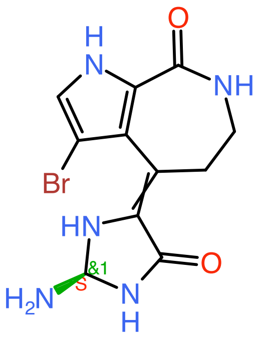	N536	A	0.5
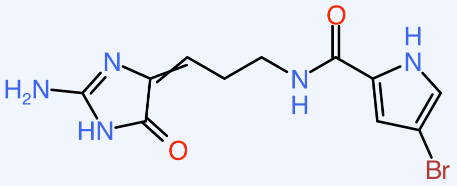	N528	A	1

Structure of Smiles	ID	Activity_Status	Consensus_score
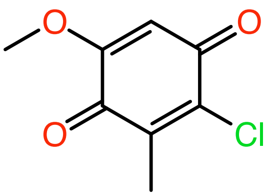	N430	A	1
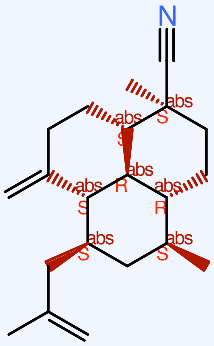	N329	A	0.5
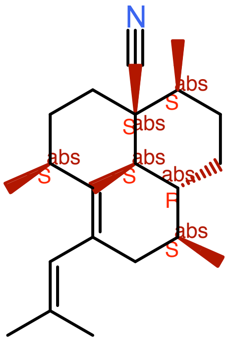	N330	A	0.5
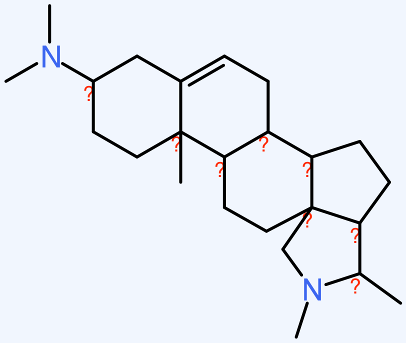	N341	A	0.75
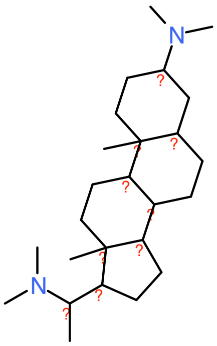	N472	A	0.5

Structure of Smiles	ID	Activity_Status	Consensus_score
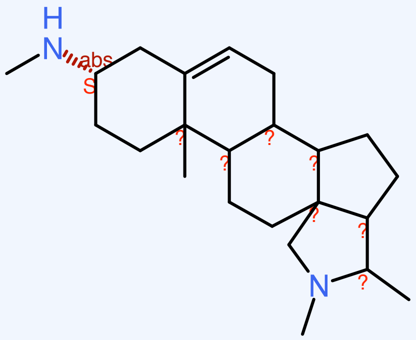	N534	A	0.75
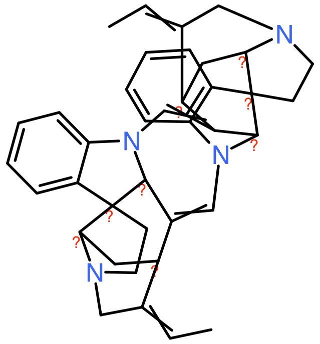	N608	A	0
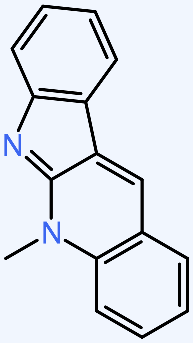	N484	A	1
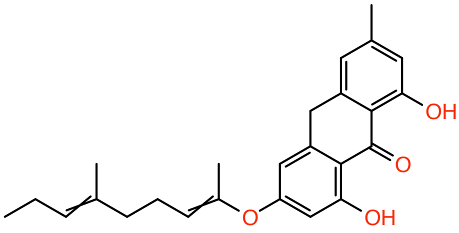	N416	A	0.25
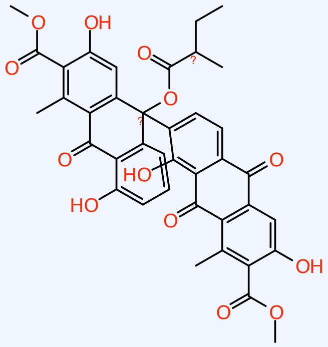	N418	A	-0.5

Structure of Smiles	ID	Activity_Status	Consensus_score
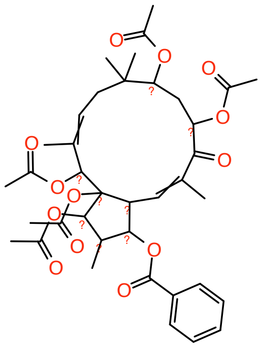	N643	A	-0.5
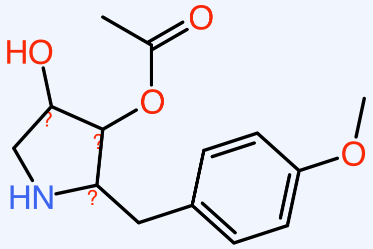	N626	A	0.75
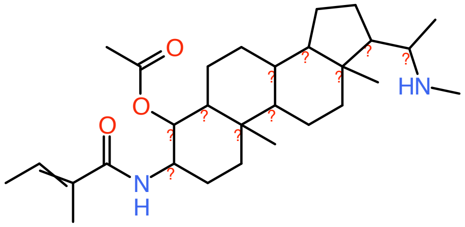	N538	A	0.5
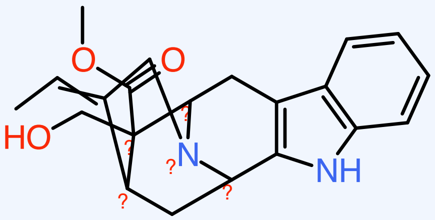	N478	A	0.75
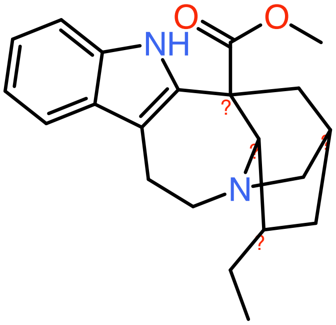	N381	A	0.75

Structure of Smiles	ID	Activity_Status	Consensus_score
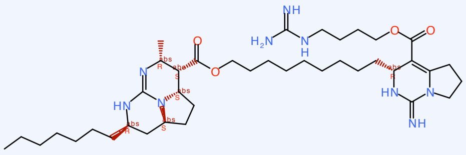	N618	A	-1
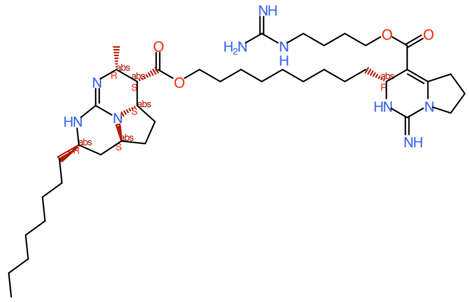	N619	A	-1
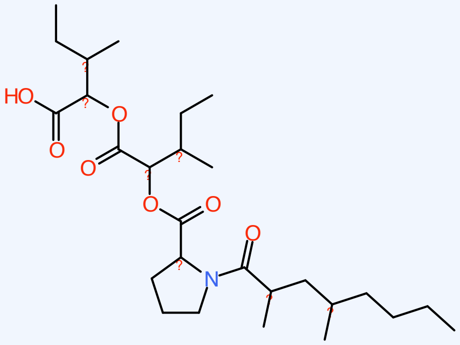	N440	A	0
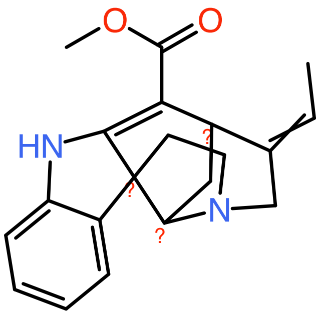	N490	A	0.75
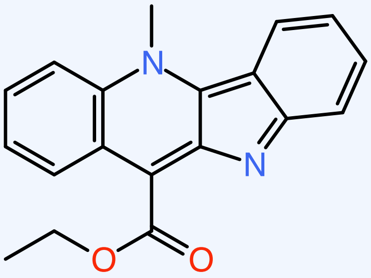	N564	A	1

Structure of Smiles	ID	Activity_Status	Consensus_score
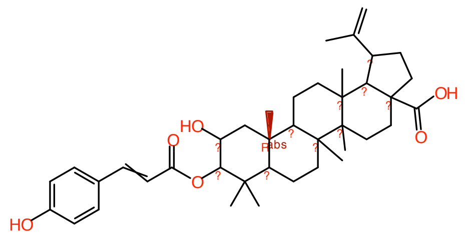	N395	A	0
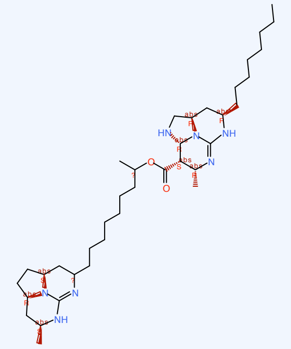	N610	A	-0.25
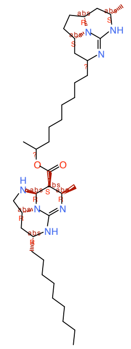	N611	A	-0.25
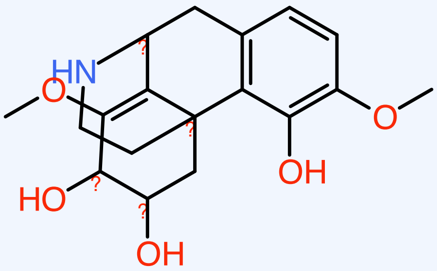	N620	A	0.75
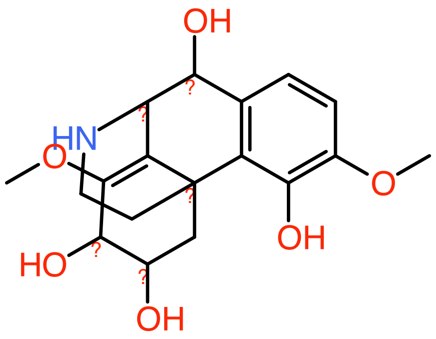	N521	A	0.5

Structure of Smiles	ID	Activity_Status	Consensus_score
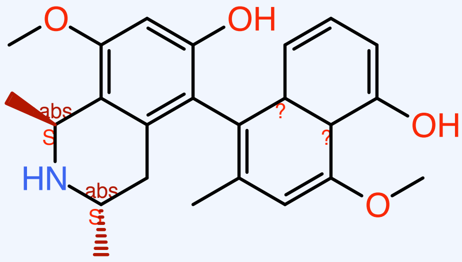	N475	A	0.75
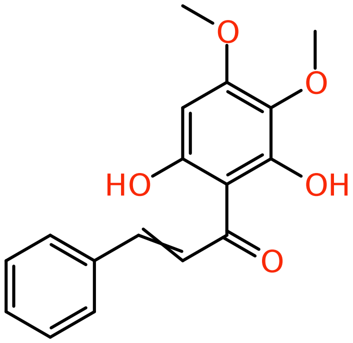	N471	A	1
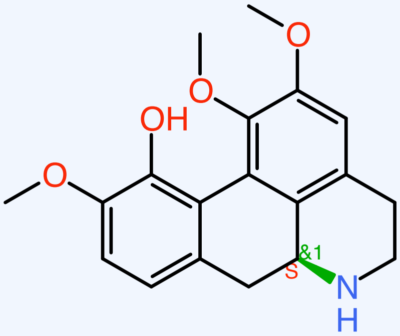	N578	A	0.75
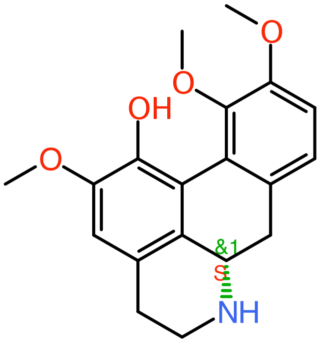	N516	A	0.75
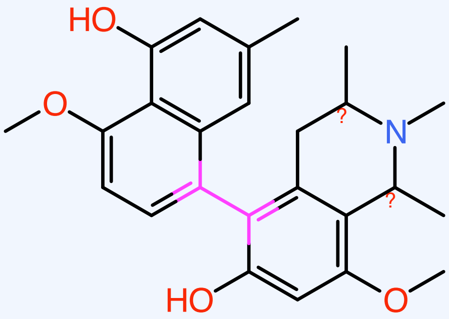	N368	A	0.5

Structure of Smiles	ID	Activity_Status	Consensus_score
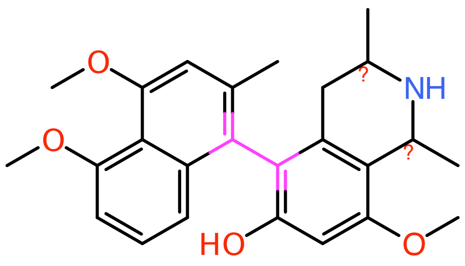	N646	A	0.5
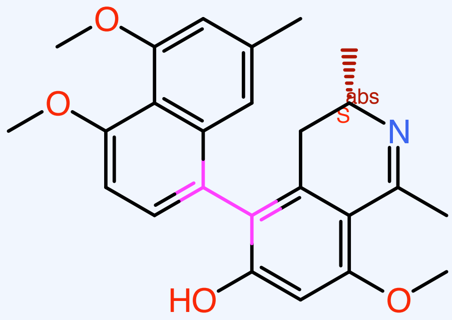	N604	A	0.25
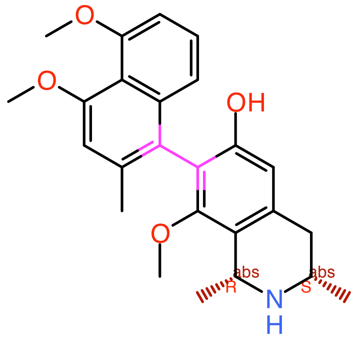	N640	A	0.5
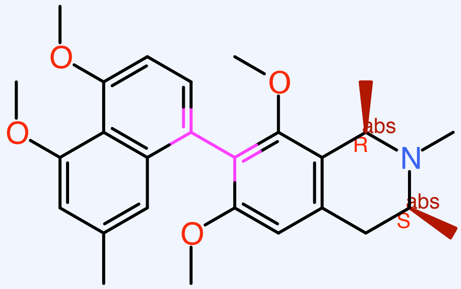	N442	A	0.25
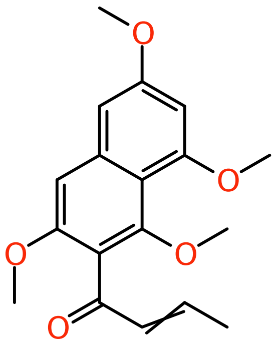	N590	A	0.75

Structure of Smiles	ID	Activity_Status	Consensus_score
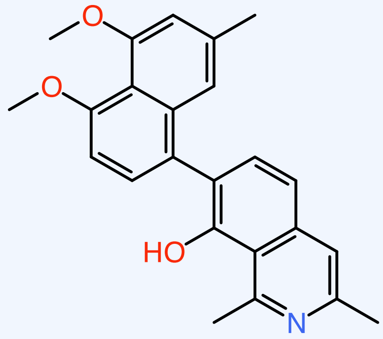	N361	A	0.25
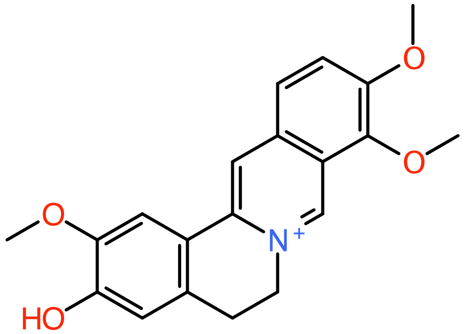	N338	A	1
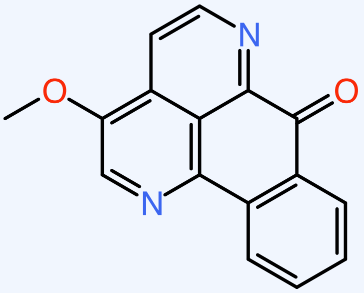	N342	A	1
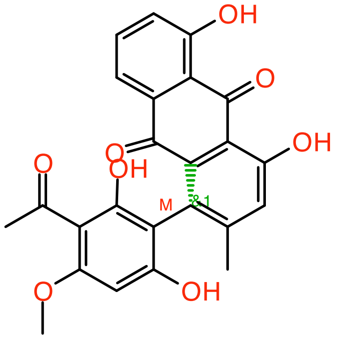	N400	A	0.5
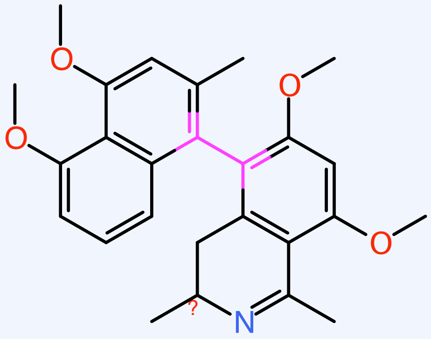	N638	A	0.25

Structure of Smiles	ID	Activity_Status	Consensus_score
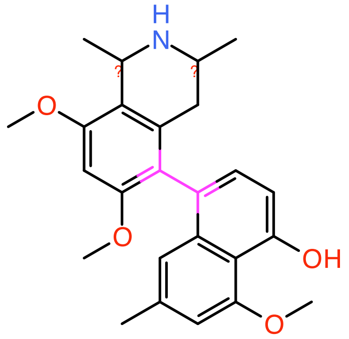	N356	A	0.5
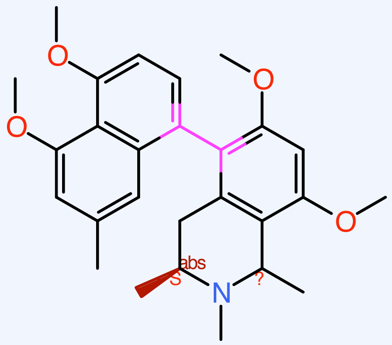	N605	A	0.25
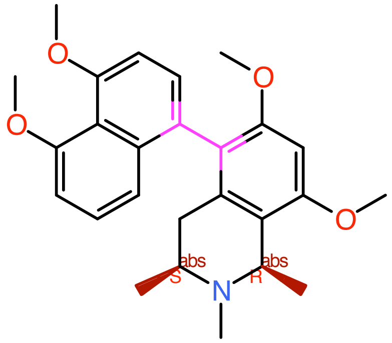	N616	A	0.25
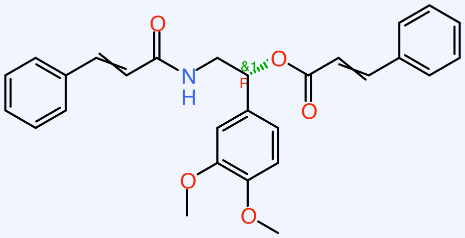	N446	A	0.5
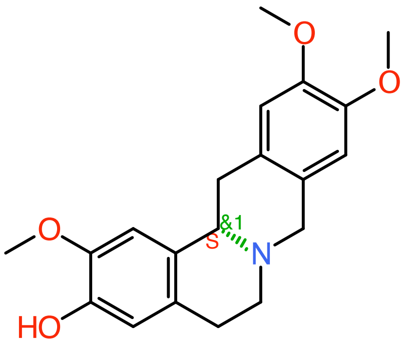	N408	A	1

Structure of Smiles	ID	Activity_Status	Consensus_score
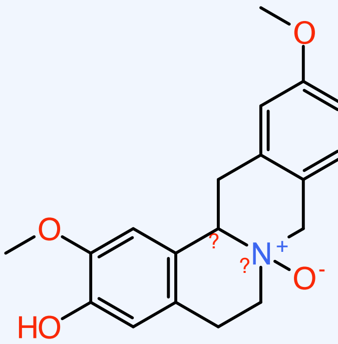	N595	A	0.75
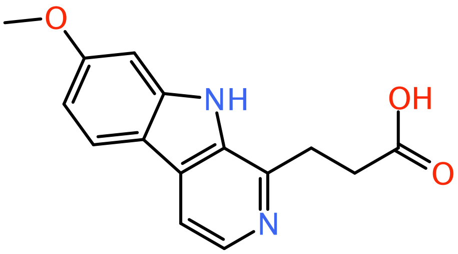	N351	A	1
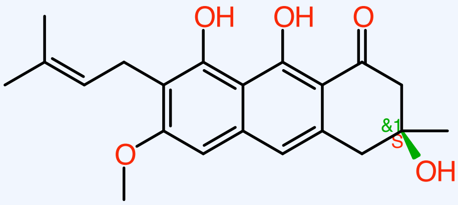	N394	A	0.75
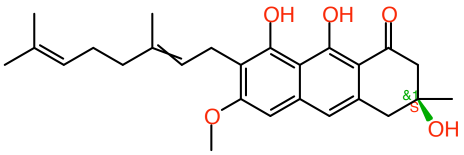	N420	A	0.25
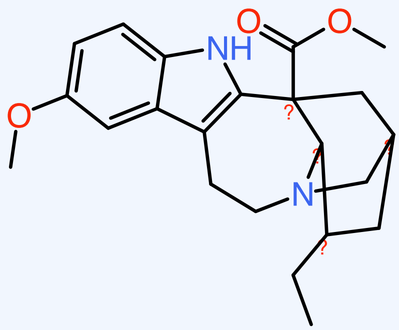	N647	A	1

Structure of Smiles	ID	Activity_Status	Consensus_score
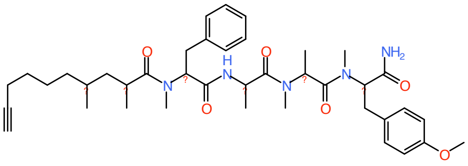	N600	A	-0.5
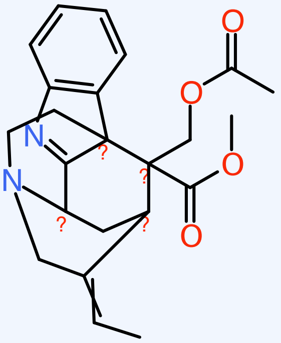	N463	A	0.75
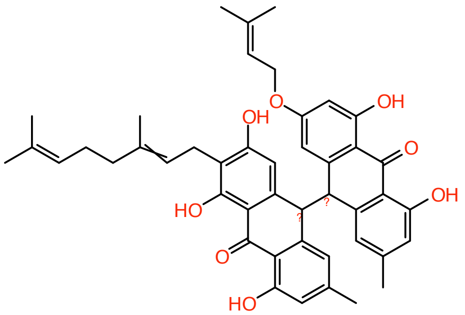	N495	A	-0.5
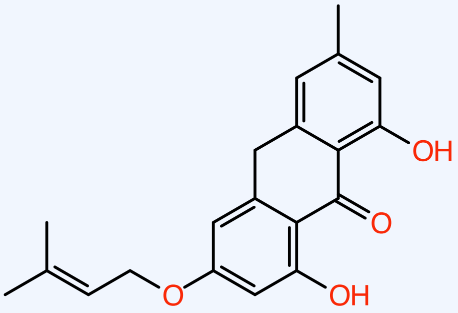	N435	A	0.75
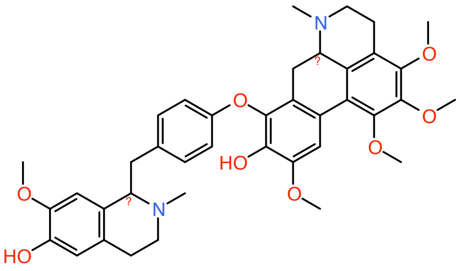	N366	A	-0.25

Structure of Smiles	ID	Activity_Status	Consensus_score
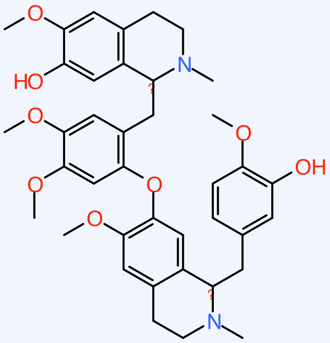	N388	A	-0.25
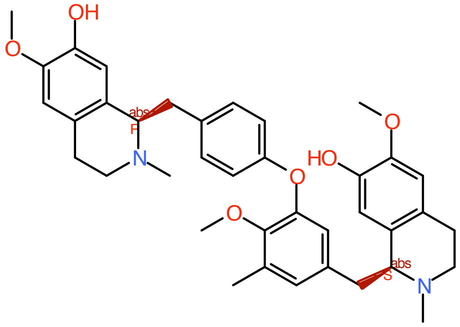	N357	A	0
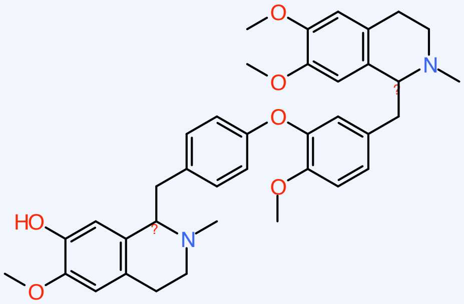	N389	A	-0.25
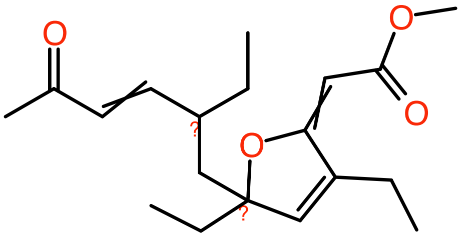	N401	A	1
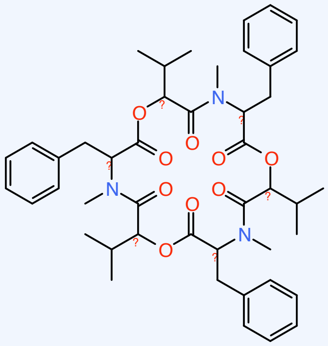	N447	A	0

Structure of Smiles	ID	Activity_Status	Consensus_score
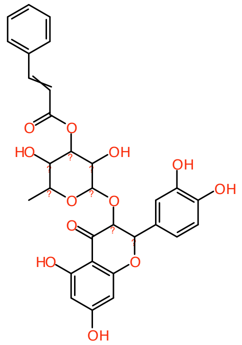	N627	A	0
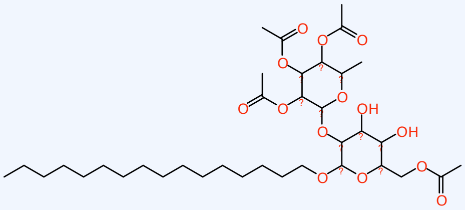	N544	A	-0.5
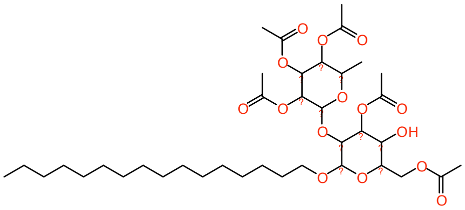	N636	A	-0.5
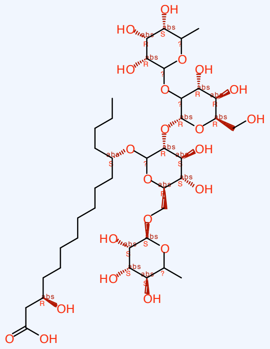	N592	A	-0.25
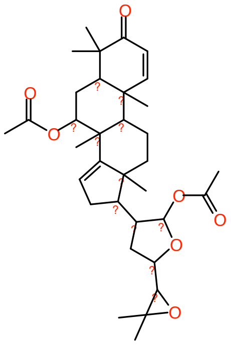	N434	A	0

Structure of Smiles	ID	Activity_Status	Consensus_score
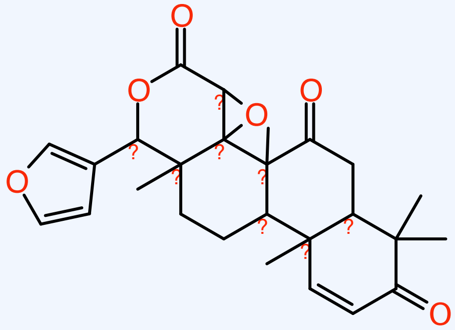	N591	A	0.75
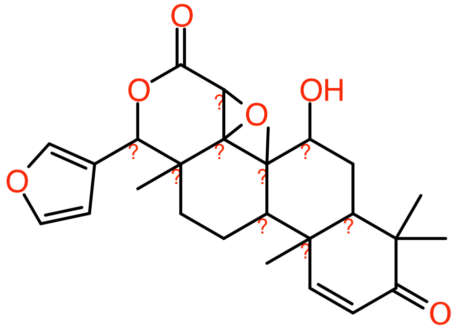	N519	A	0.75
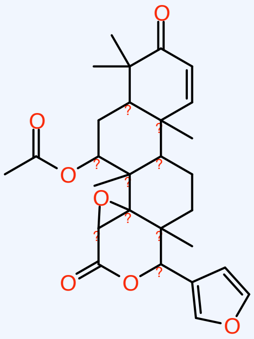	N382	A	0.75
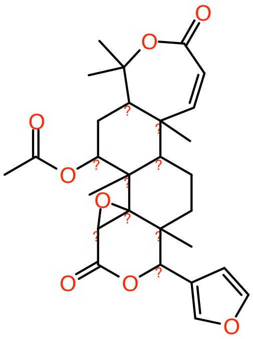	N581	A	0.75
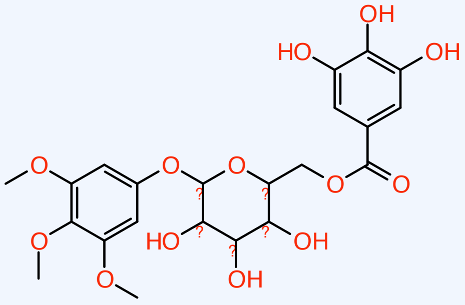	N523	A	0.25

Structure of Smiles	ID	Activity_Status	Consensus_score
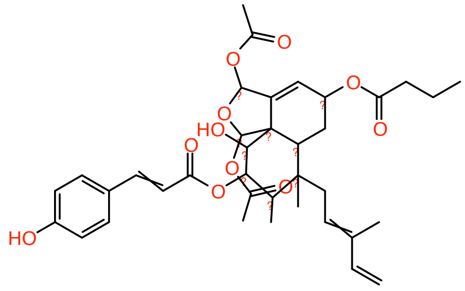	N552	A	-0.5
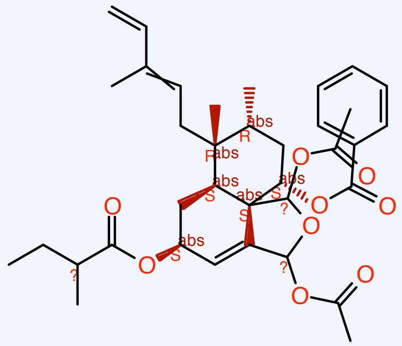	N629	A	0
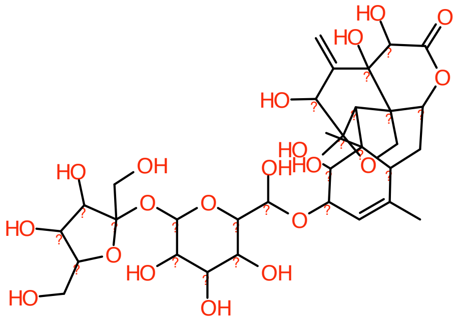	N334	A	-0.5
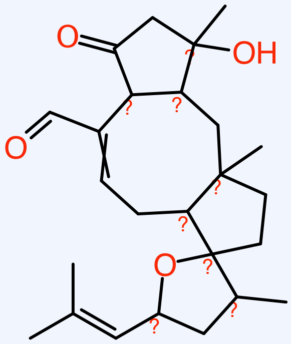	N367	A	0.5
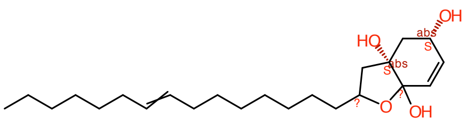	N635	A	0

Structure of Smiles	ID	Activity_Status	Consensus_score
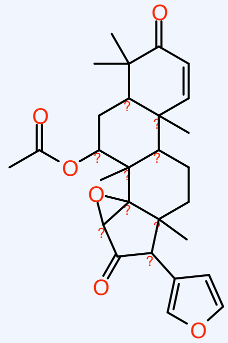	N413	A	0.5
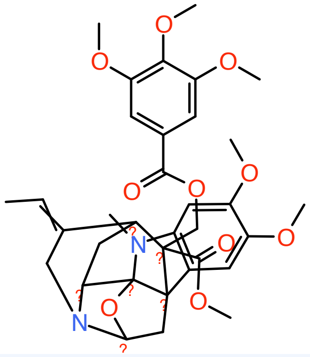	N380	A	0
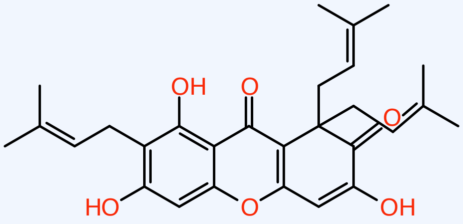	N359	A	0.25
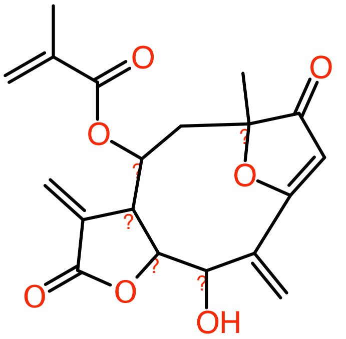	N602	A	0.75
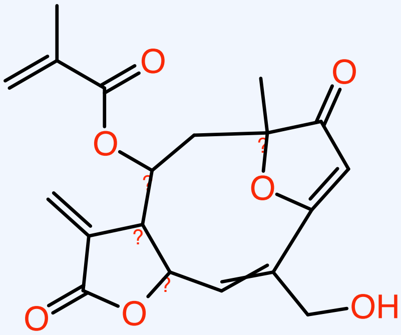	N529	A	0.75

Structure of Smiles	ID	Activity_Status	Consensus_score
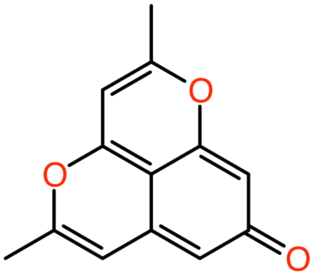	N633	A	0.75
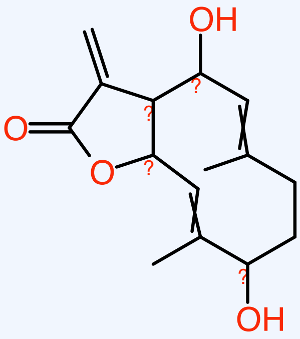	N385	A	0.75
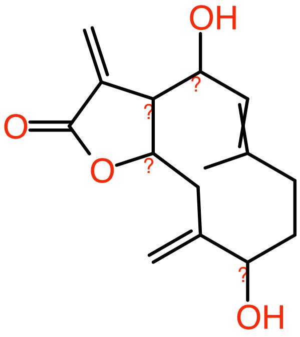	N386	A	0.75
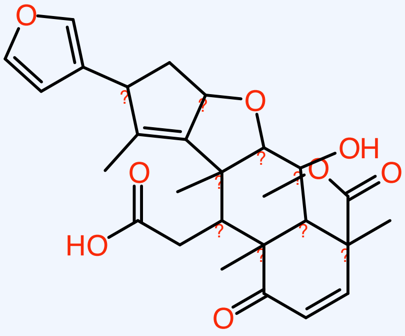	N404	A	0.75
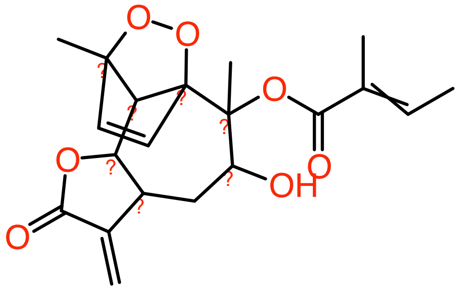	N486	A	0.75

Structure of Smiles	ID	Activity_Status	Consensus_score
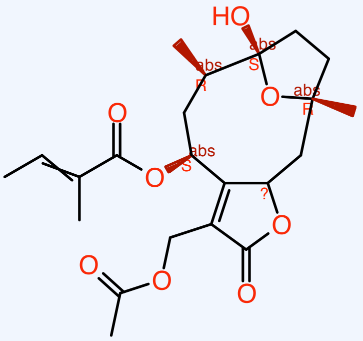	N571	A	0.75
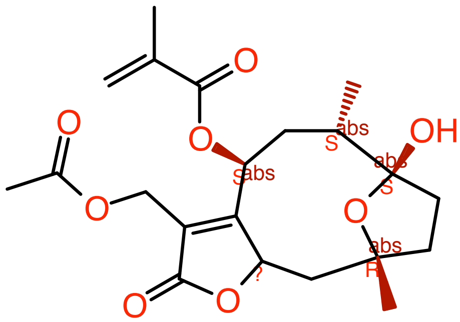	N615	A	0.75
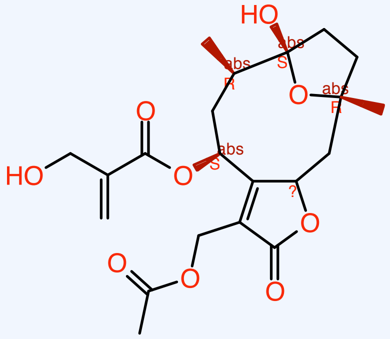	N557	A	0.75
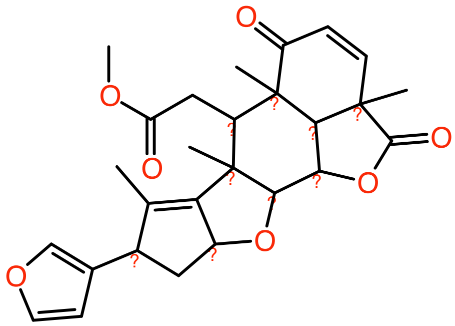	N565	A	0.75
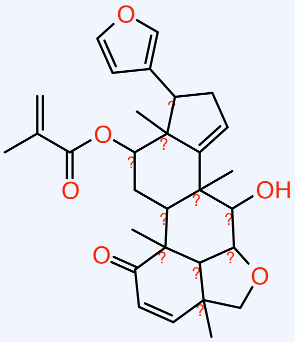	N641	A	0.5

Structure of Smiles	ID	Activity_Status	Consensus_score
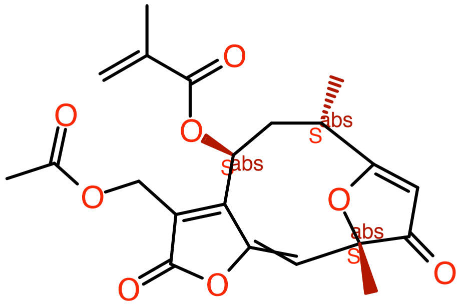	N607	A	0.75
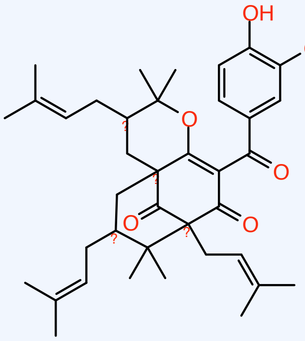	N457	A	0
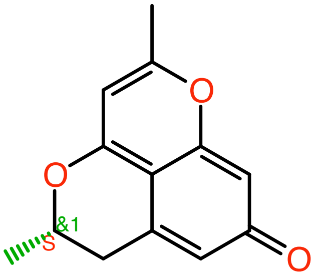	N464	A	0.75
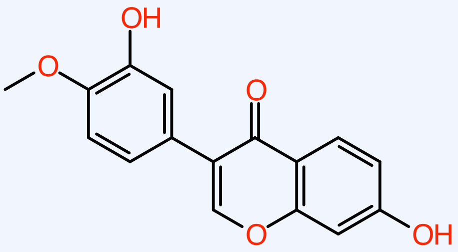	N355	A	1
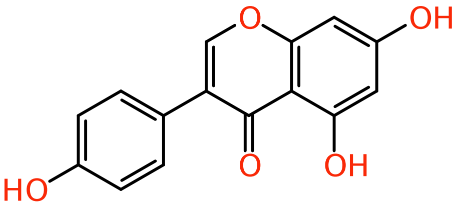	N436	A	1

Structure of Smiles	ID	Activity_Status	Consensus_score
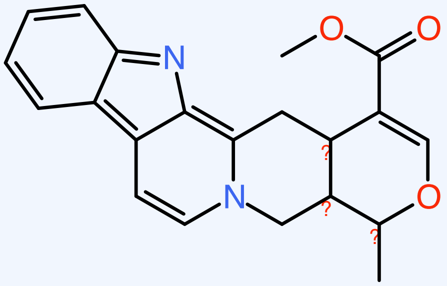	N480	A	0.75
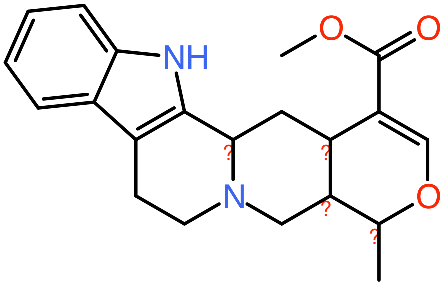	N479	A	0.75
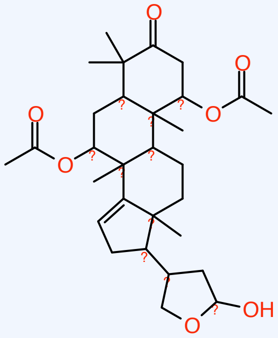	N20	A	0.25
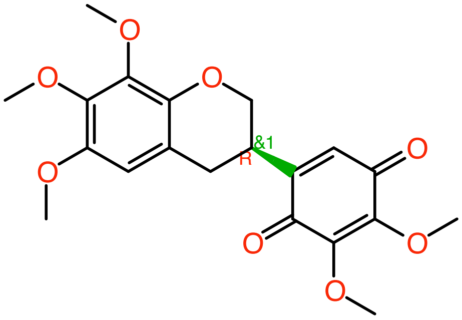	N569	A	0.75
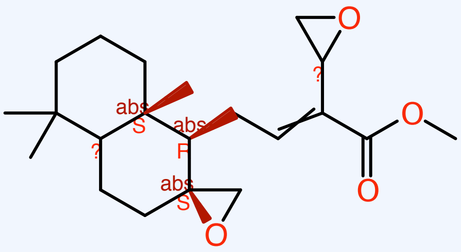	N649	A	0.75

Structure of Smiles	ID	Activity_Status	Consensus_score
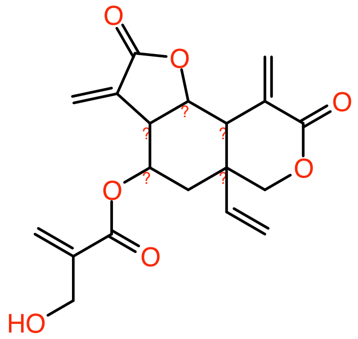	N373	A	0.75
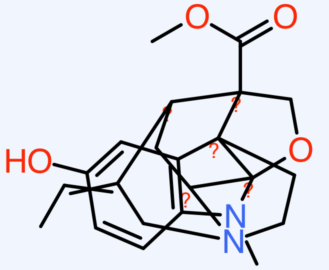	N467	A	0.75
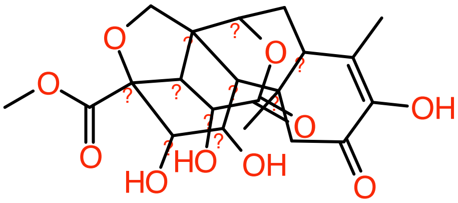	N337	A	0.25
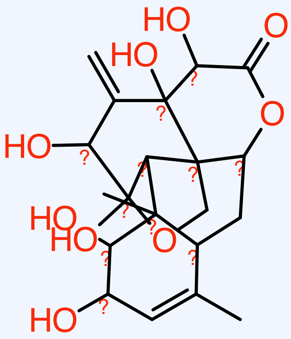	N439	A	0.25
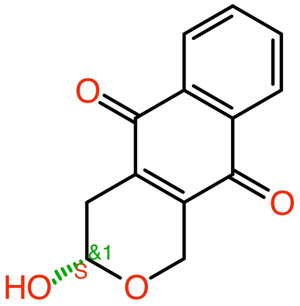	N15	A	0.75

Structure of Smiles	ID	Activity_Status	Consensus_score
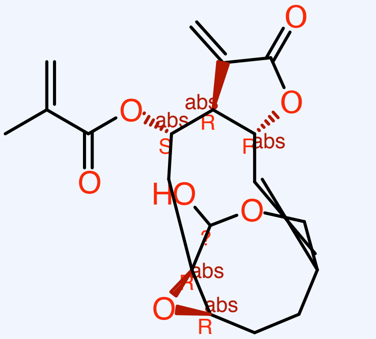	N546	A	0.75
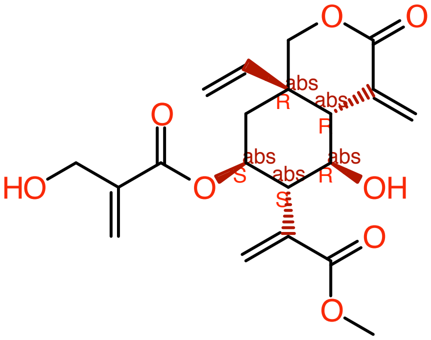	N499	A	0.75
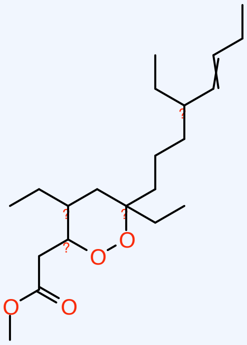	N370	A	0
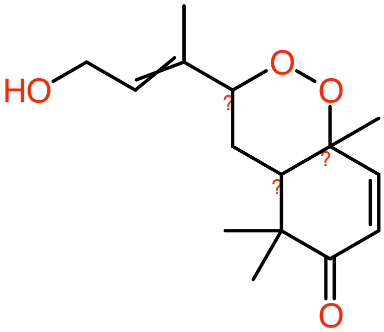	N425	A	0.75
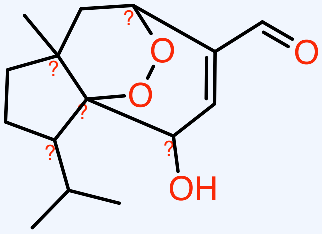	N566	A	0.75

Structure of Smiles	ID	Activity_Status	Consensus_score
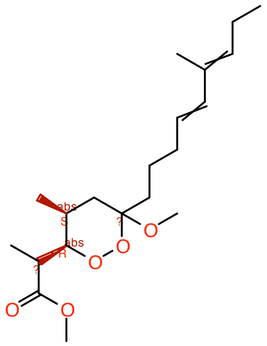	N345	A	0
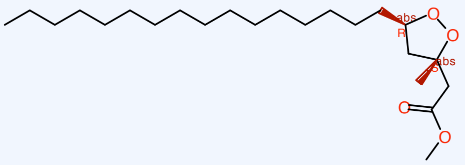	N498	A	0.25
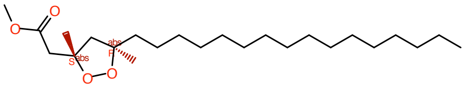	N586	A	0.25
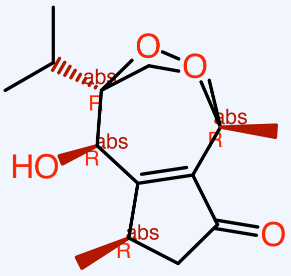	N396	A	0.75
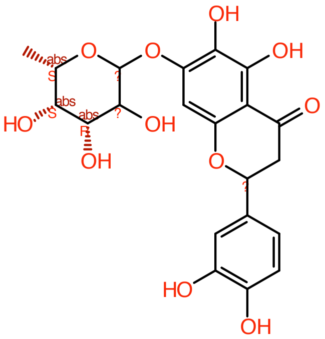	N454	A	0.25

Structure of Smiles	ID	Activity_Status	Consensus_score
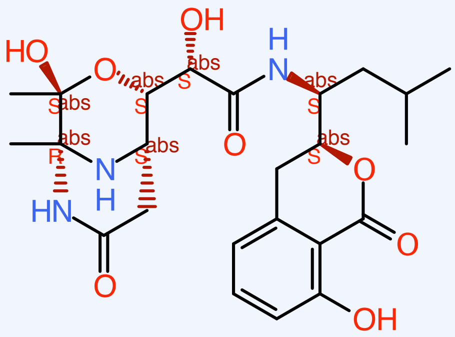	N460	A	0.25
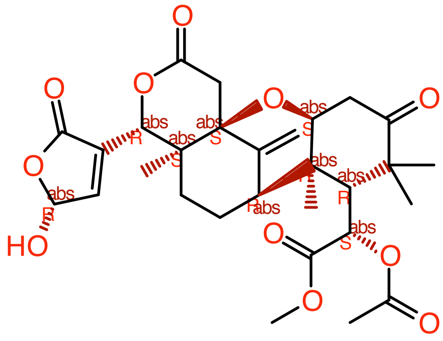	N588	A	0
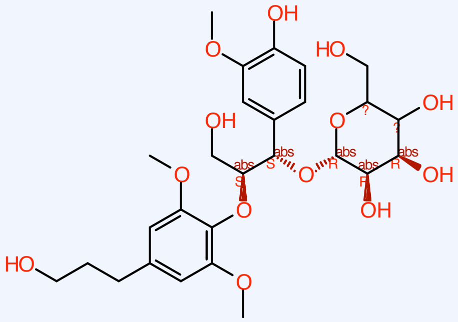	N453	A	-0.25
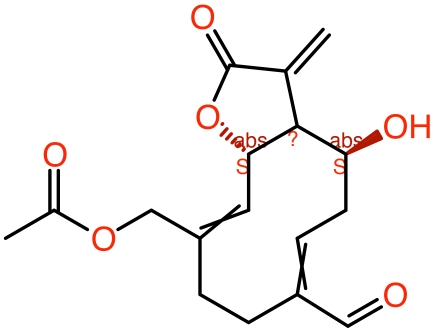	N473	A	0.75
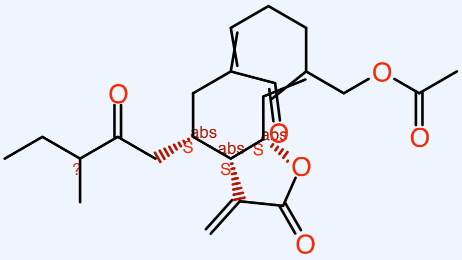	N491	A	0.75

Structure of Smiles	ID	Activity_Status	Consensus_score
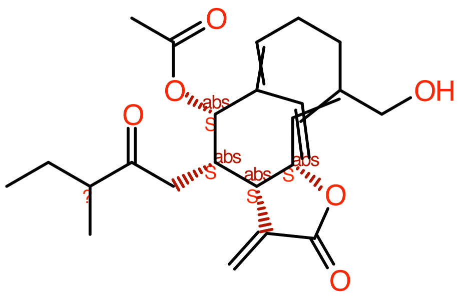	N461	A	0.5
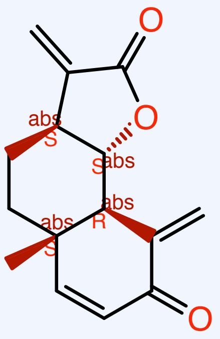	N432	A	0.75
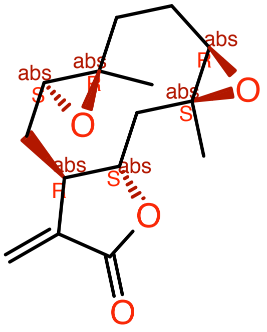	N3	A	0.75
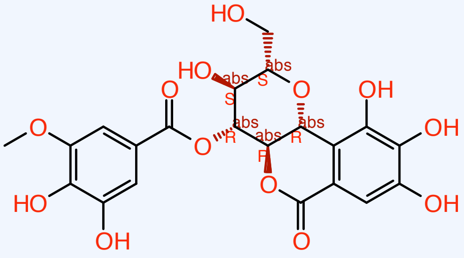	N358	A	0
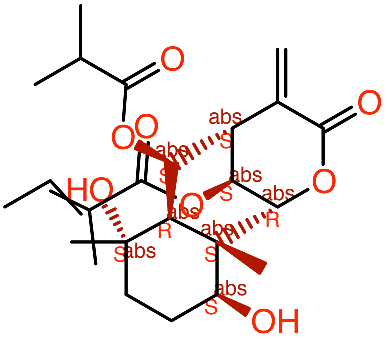	N594	A	0.75

Structure of Smiles	ID	Activity_Status	Consensus_score
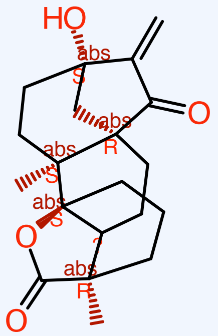	N445	A	0.75
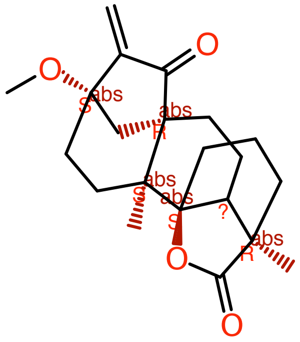	N403	A	0.75
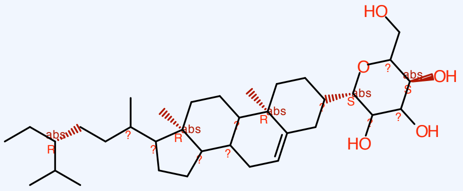	N526	A	0
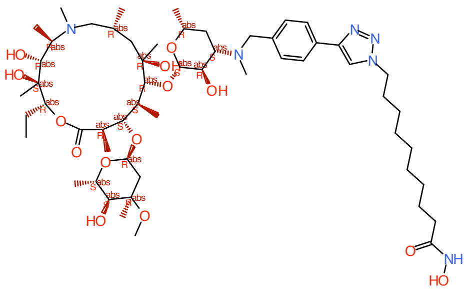	N530	A	-0.5
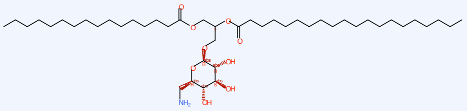	N622	A	-0.5

Structure of Smiles	ID	Activity_Status	Consensus_score
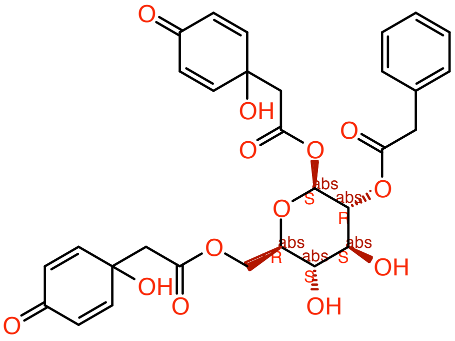	N331	A	0
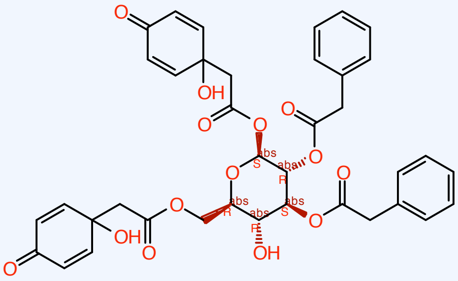	N384	A	0
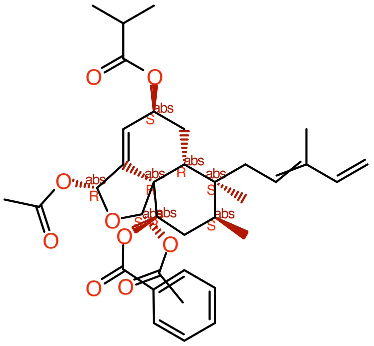	N606	A	-0.25
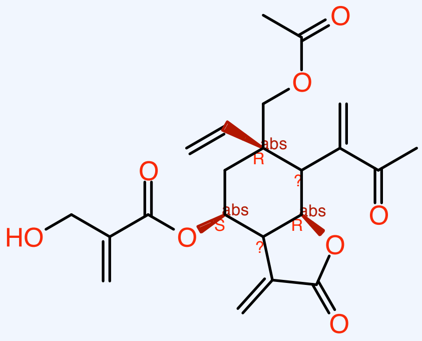	N506	A	0.5
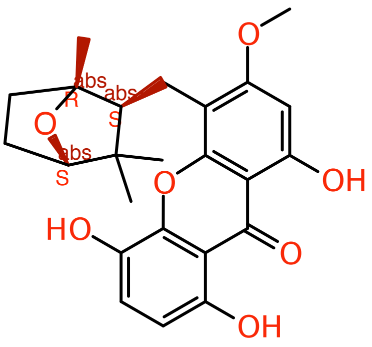	N501	A	0.5

Structure of Smiles	ID	Activity_Status	Consensus_score
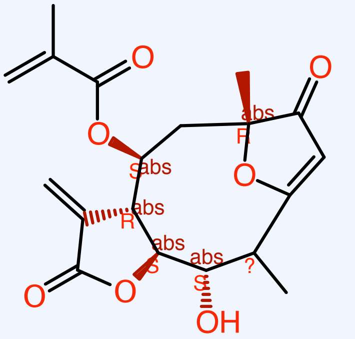	N603	A	0.75
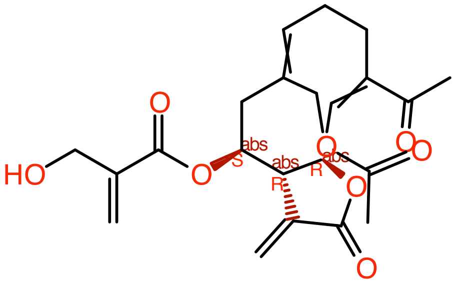	N507	A	0.75
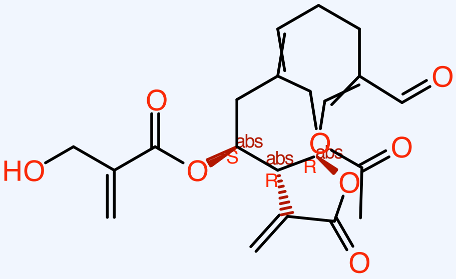	N505	A	0.75
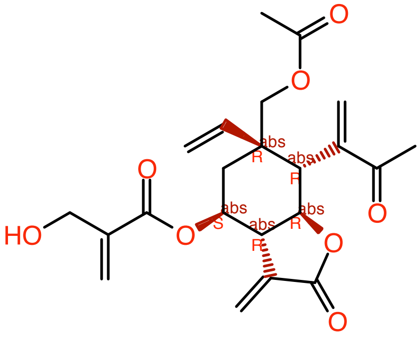	N500	A	0.5
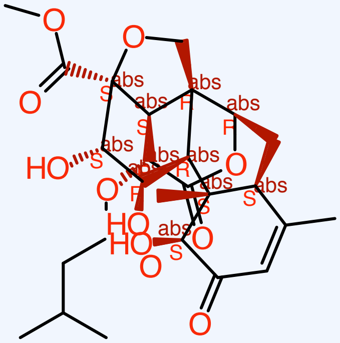	N354	A	0

Structure of Smiles	ID	Activity_Status	Consensus_score
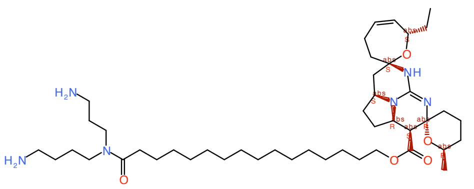	N621	A	-0.75
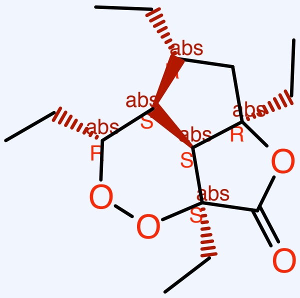	N531	A	0.75
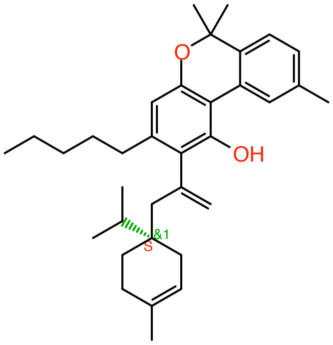	N648	A	0.25
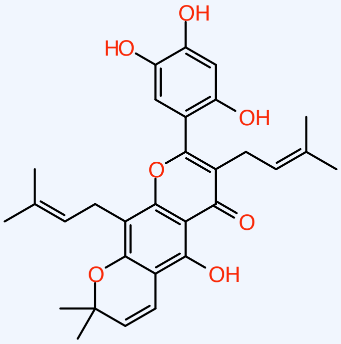	N340	A	0
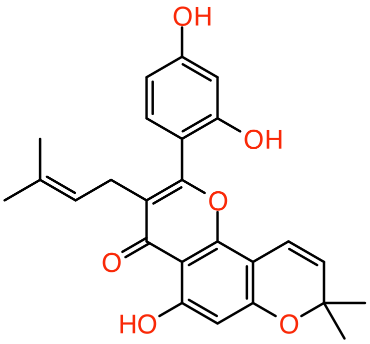	N617	A	0.25

Structure of Smiles	ID	Activity_Status	Consensus_score
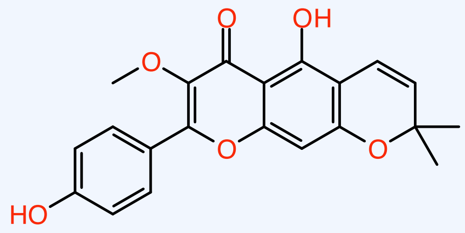	N540	A	1
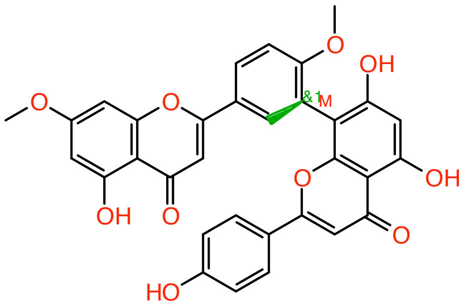	N441	A	-0.25
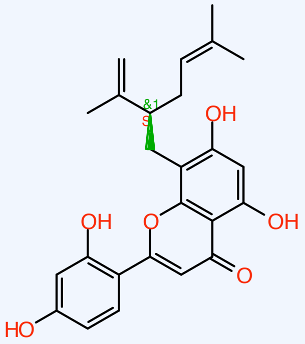	N483	A	0.25
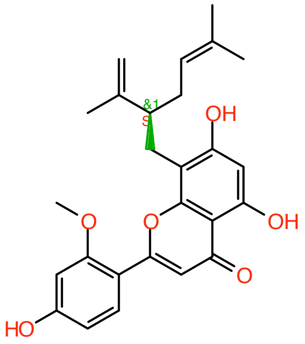	N452	A	0.25
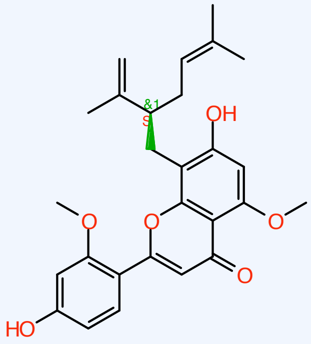	N470	A	0.25

Structure of Smiles	ID	Activity_Status	Consensus_score
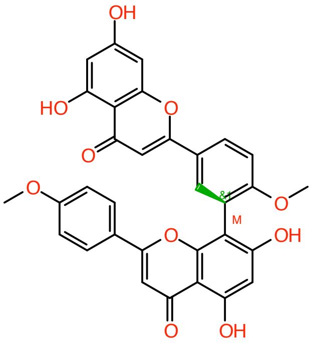	N545	A	-0.25
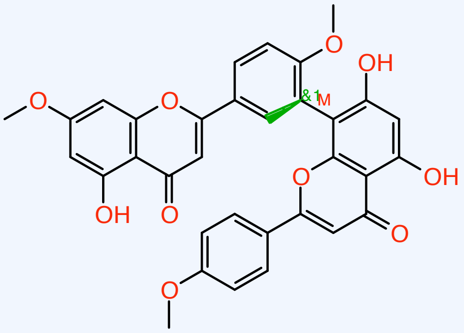	N378	A	-0.25
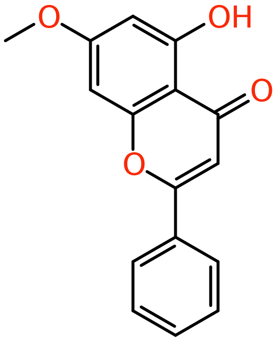	N577	A	1
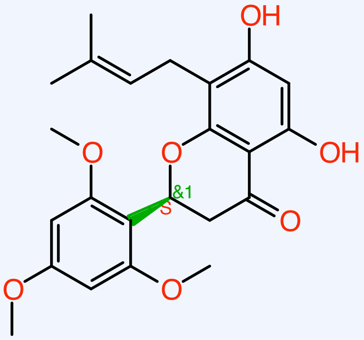	N363	A	0.5
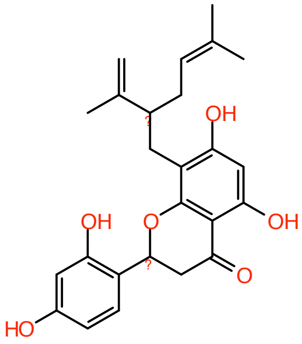	N482	A	0.25

Structure of Smiles	ID	Activity_Status	Consensus_score
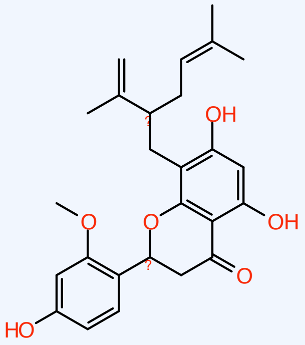	N451	A	0.25
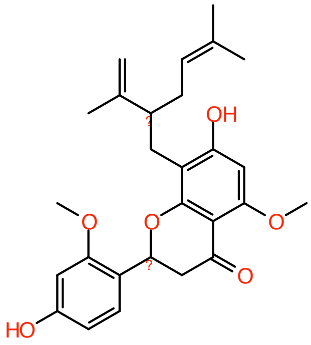	N469	A	0.25
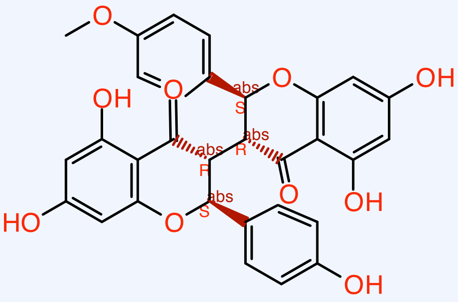	N326	A	-0.5
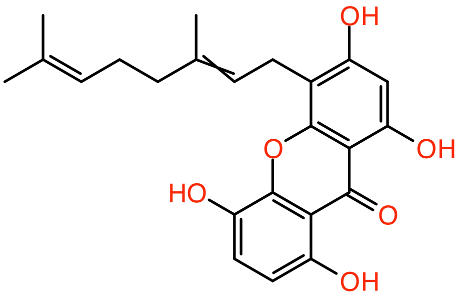	N508	A	0.25
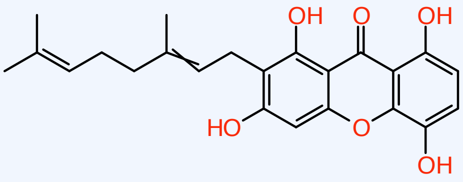	N504	A	0.25

Structure of Smiles	ID	Activity_Status	Consensus_score
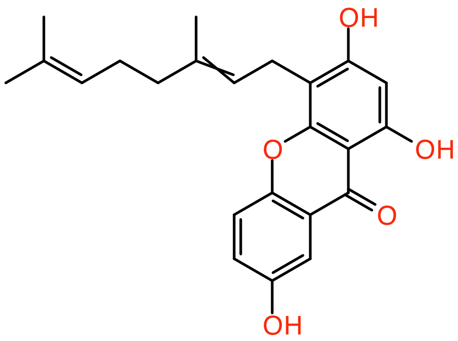	N360	A	0.25
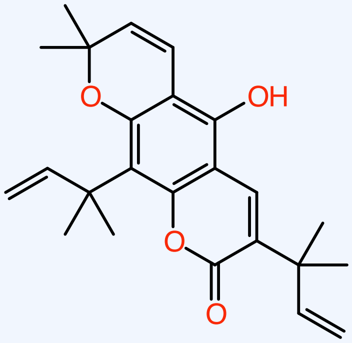	N365	A	0.25
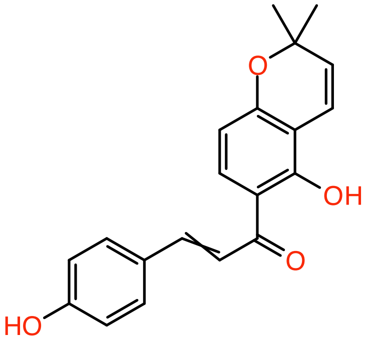	N539	A	1
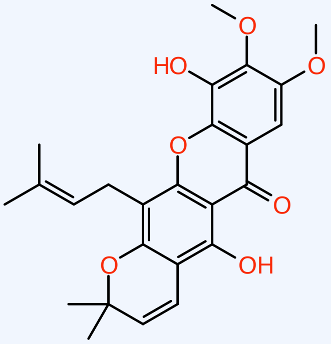	N547	A	0.25
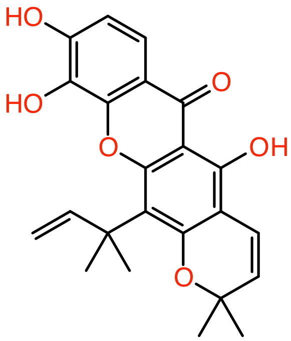	N353	A	0.25

Structure of Smiles	ID	Activity_Status	Consensus_score
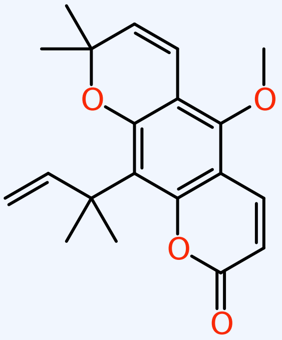	N398	A	0.75
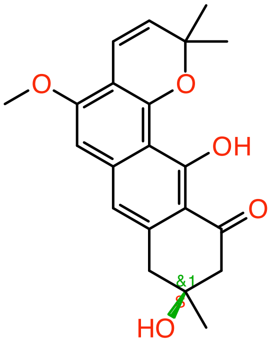	N409	A	0.75
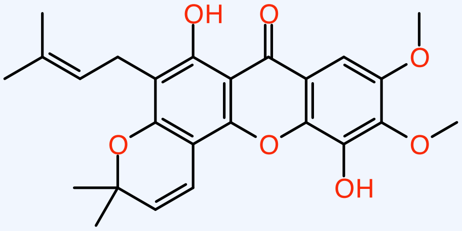	N575	A	0.25
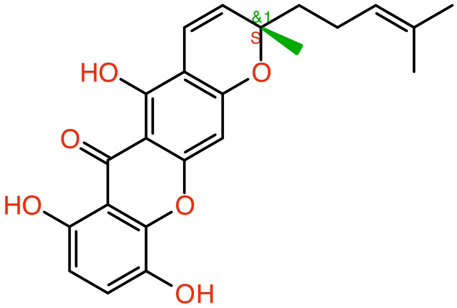	N509	A	0.25
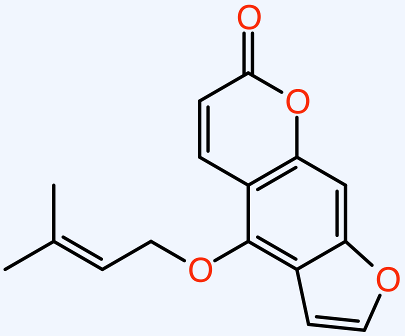	N487	A	1

Structure of Smiles	ID	Activity_Status	Consensus_score
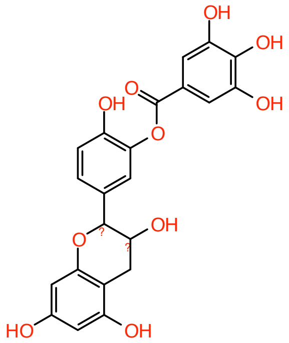	N333	A	0
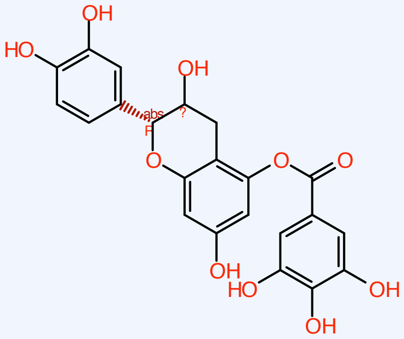	N352	A	0
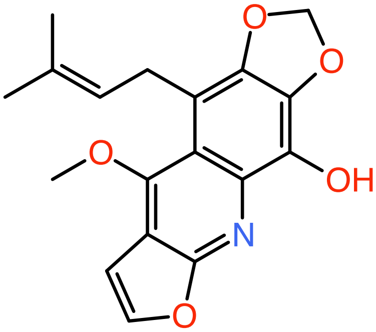	N513	A	0.75
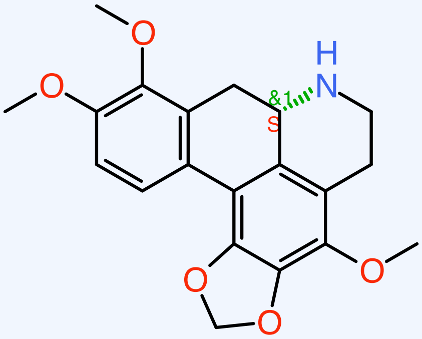	N553	A	0.75
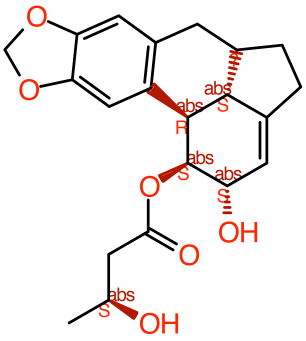	N405	A	1

Structure of Smiles	ID	Activity_Status	Consensus_score
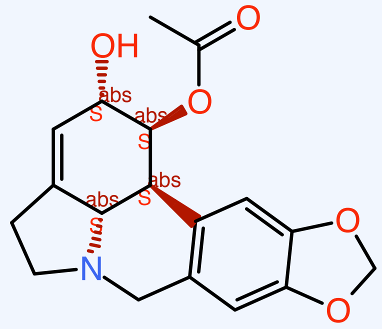	N515	A	0.75
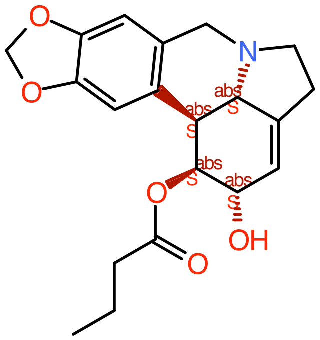	N339	A	0.75
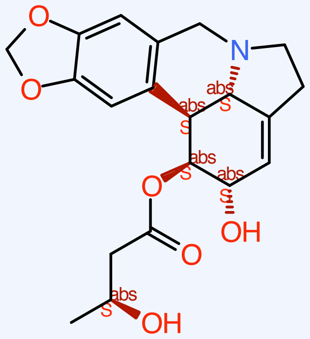	N410	A	0.75
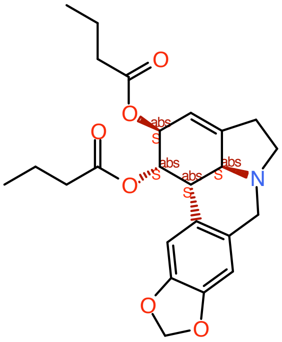	N402	A	0.5
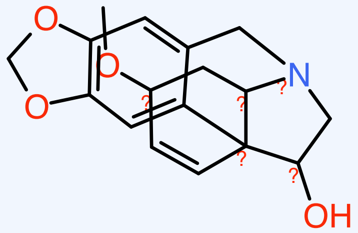	N466	A	0.75

Structure of Smiles	ID	Activity_Status	Consensus_score
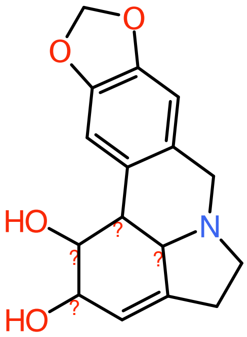	N449	A	0.75
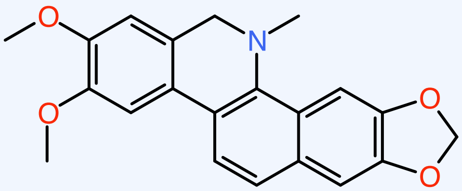	N645	A	0.75
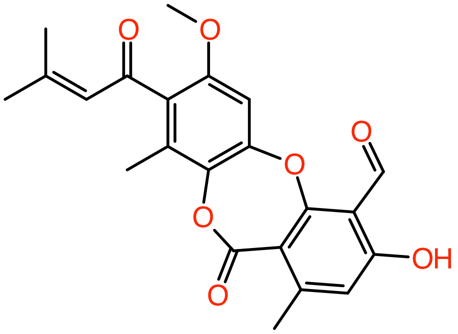	N520	A	0.75
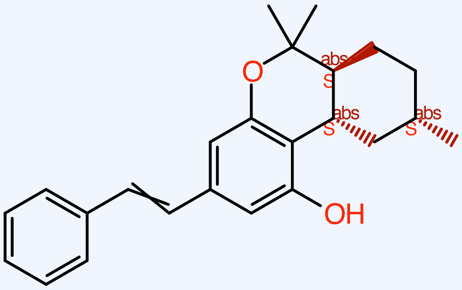	N422	A	0.25
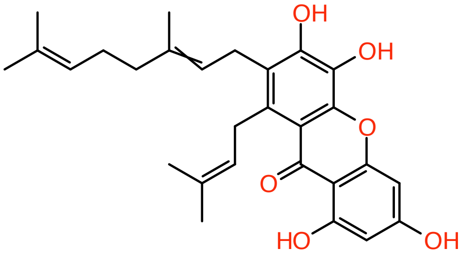	N448	A	0.25

Structure of Smiles	ID	Activity_Status	Consensus_score
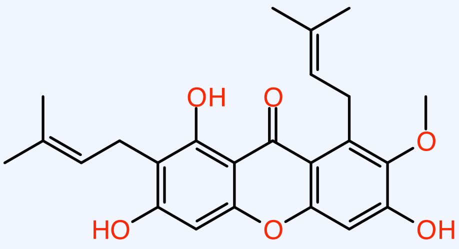	N589	A	0.25
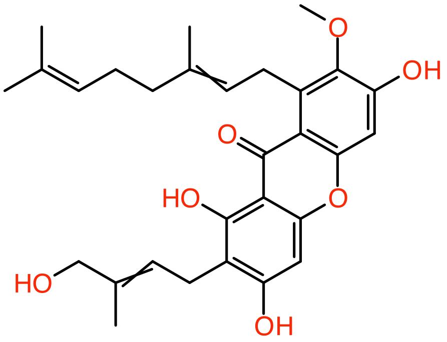	N524	A	0.25
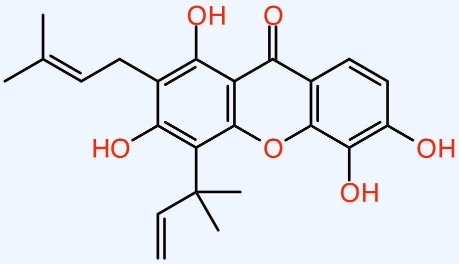	N344	A	0.25
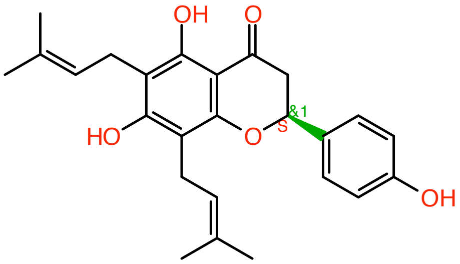	N573	A	0.25
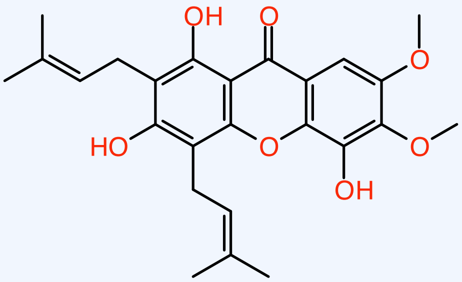	N362	A	0.25

Structure of Smiles	ID	Activity_Status	Consensus_score
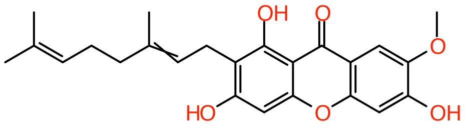	N555	A	0.25
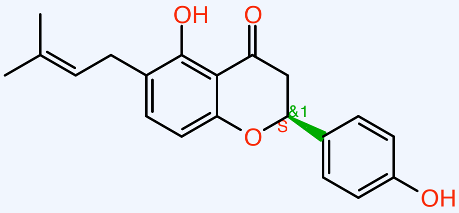	N525	A	1
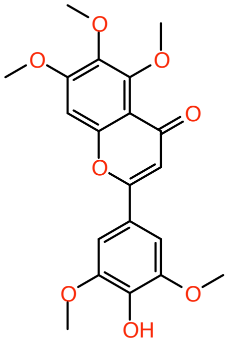	N554	A	1
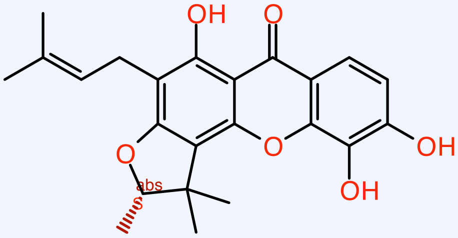	N372	A	0.25
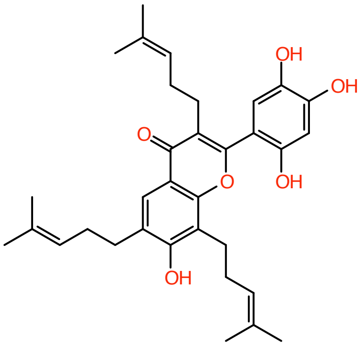	N348	A	-0.25

Structure of Smiles	ID	Activity_Status	Consensus_score
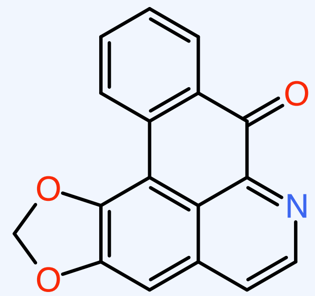	N468	A	0.75
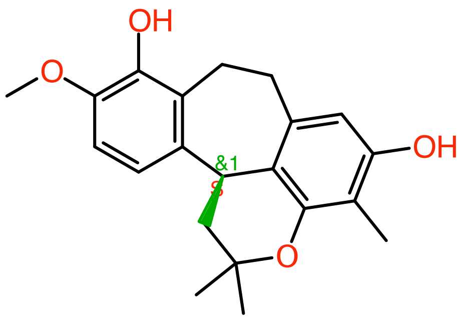	N485	A	0.75
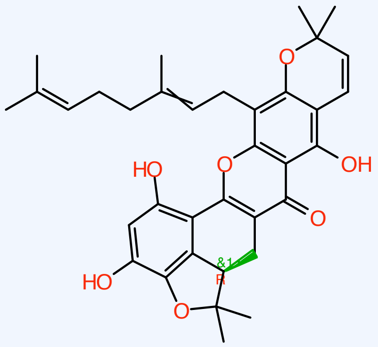	N364	A	0
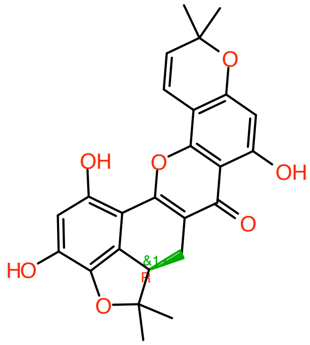	N562	A	0.5
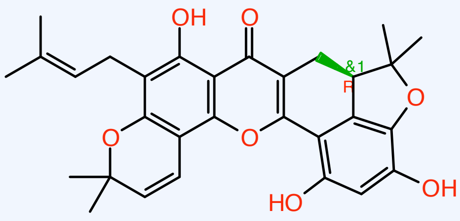	N639	A	0

Structure of Smiles	ID	Activity_Status	Consensus_score
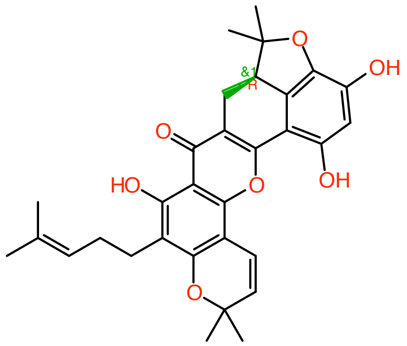	N628	A	0
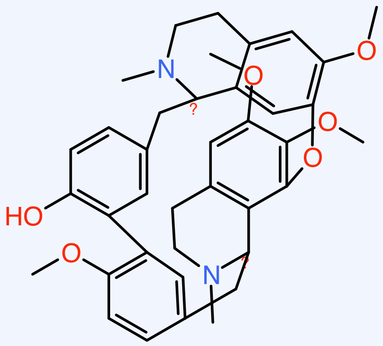	N350	A	0
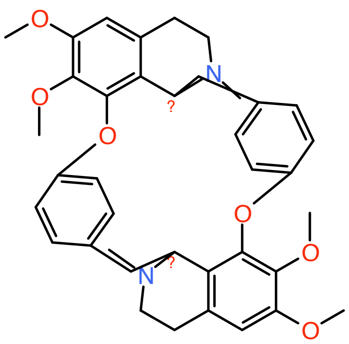	N391	A	0
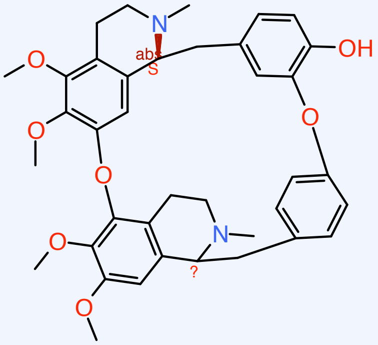	N527	A	0
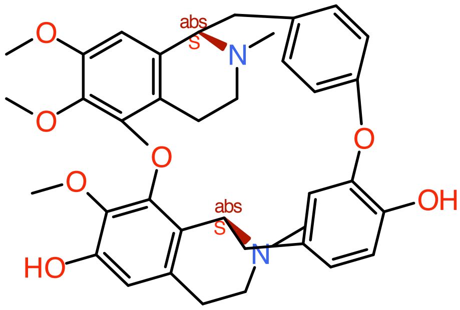	N609	A	0

Structure of Smiles	ID	Activity_Status	Consensus_score
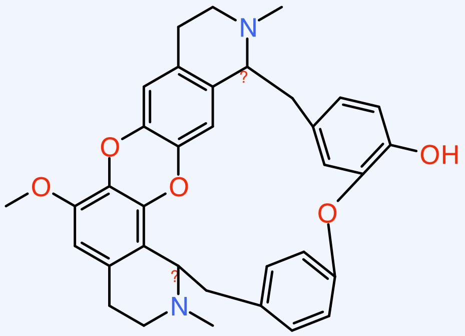	N347	A	0
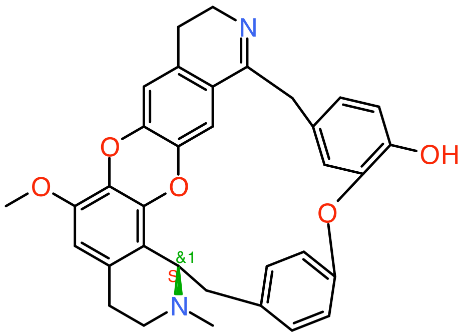	N412	A	0.25
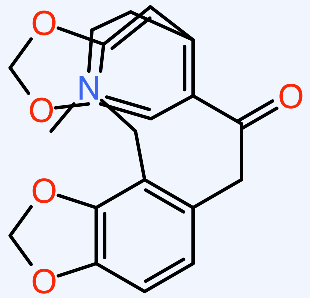	N489	A	1
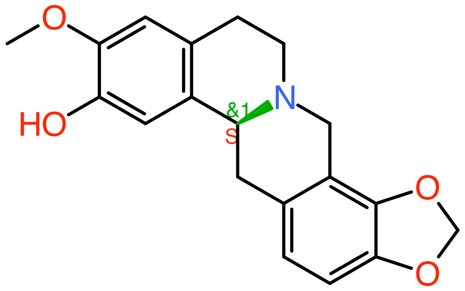	N598	A	1
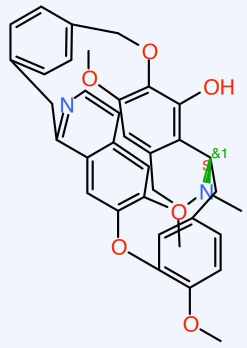	N393	A	0

Structure of Smiles	ID	Activity_Status	Consensus_score
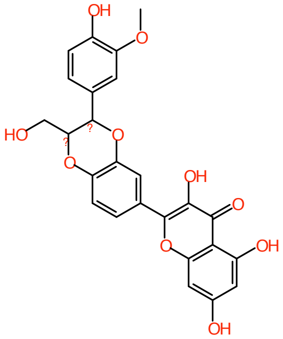	N419	A	0.25
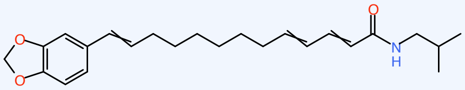	N561	A	0
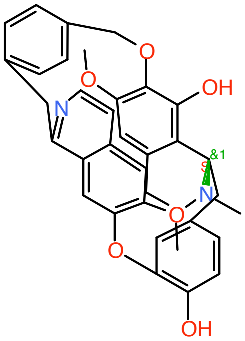	N392	A	0
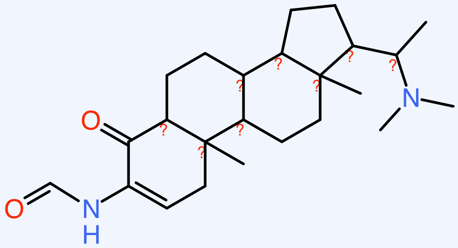	N543	A	0.75
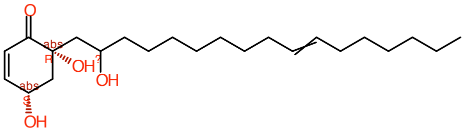	N634	A	0

Structure of Smiles	ID	Activity_Status	Consensus_score
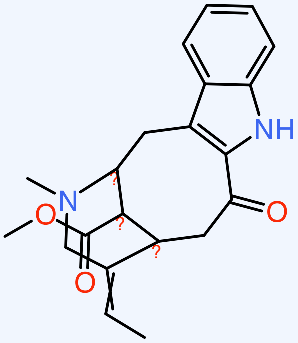	N369	A	0.75
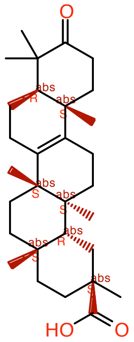	N558	A	0.25
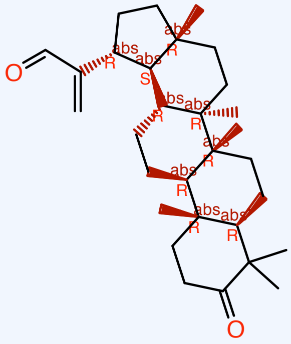	N548	A	0.25
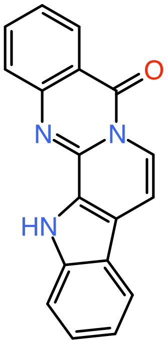	N535	A	0.75
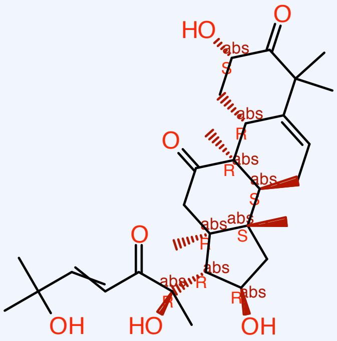	N580	A	0.5

Structure of Smiles	ID	Activity_Status	Consensus_score
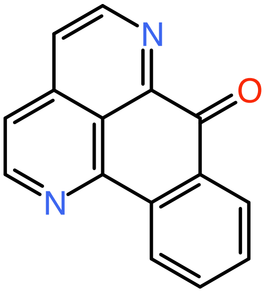	N429	A	1
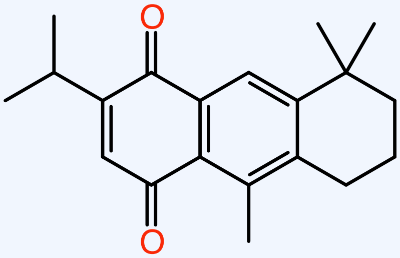	N631	A	0.75
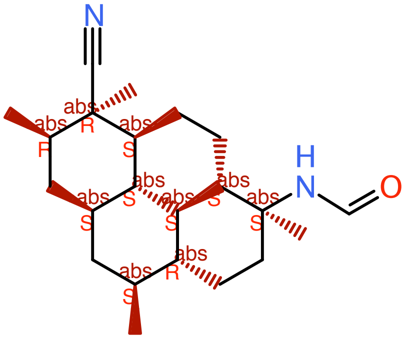	N383	A	0.75
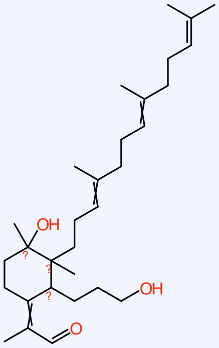	N576	A	0
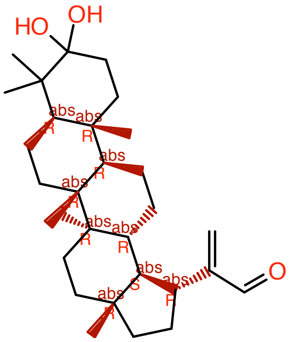	N550	A	0.25

Structure of Smiles	ID	Activity_Status	Consensus_score
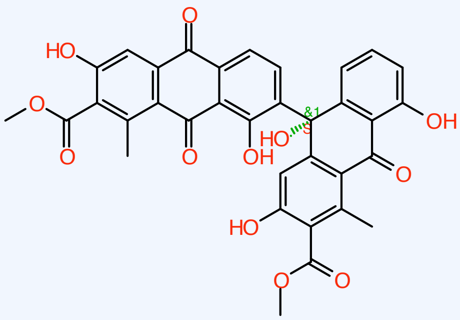	N582	A	-0.5
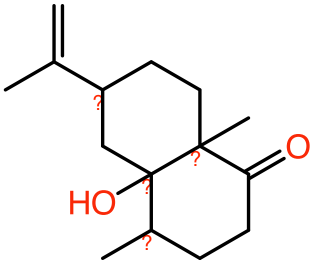	N623	A	0.75
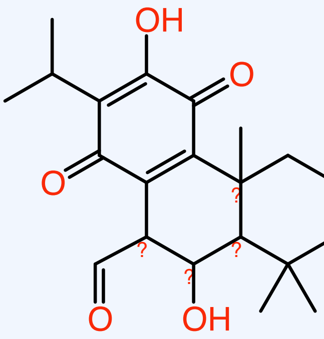	N625	A	0.75
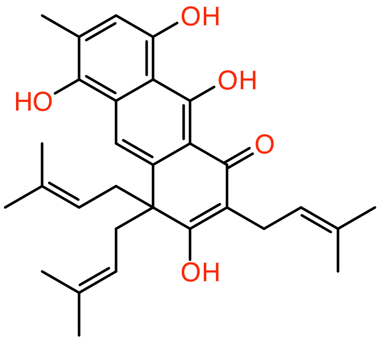	N428	A	0.25
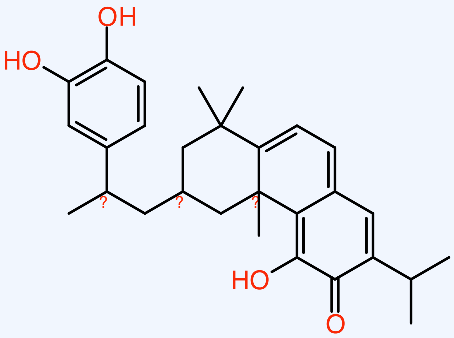	N630	A	0.25

Structure of Smiles	ID	Activity_Status	Consensus_score
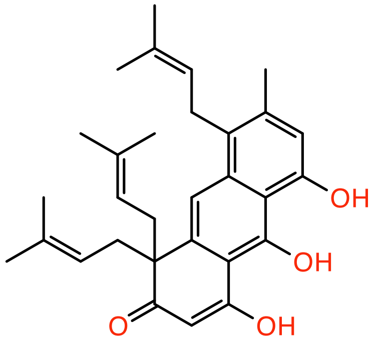	N488	A	0.25
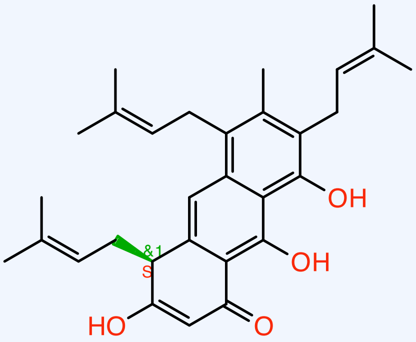	N559	A	0.25
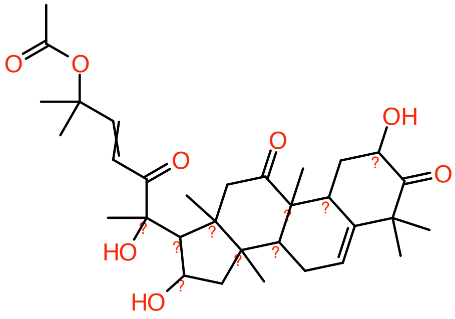	N406	A	0.25
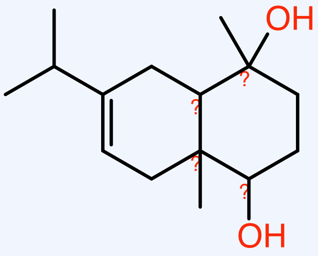	N593	A	0.75
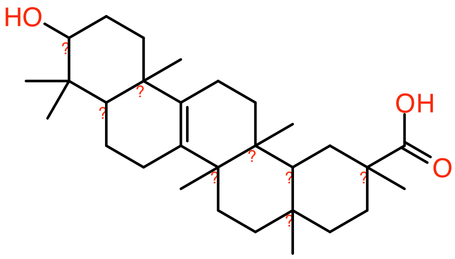	N443	A	0.25

Structure of Smiles	ID	Activity_Status	Consensus_score
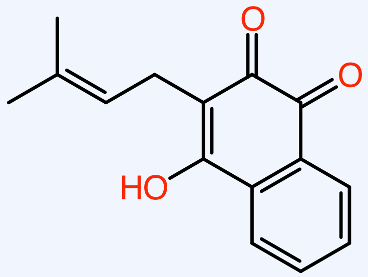	N642	A	1
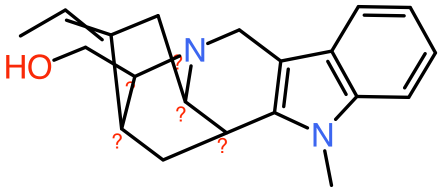	N556	A	0.75
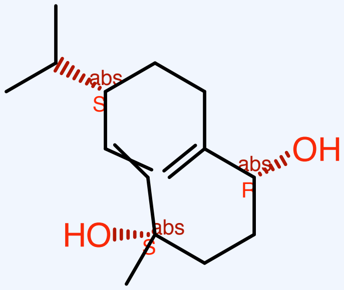	N411	A	0.75
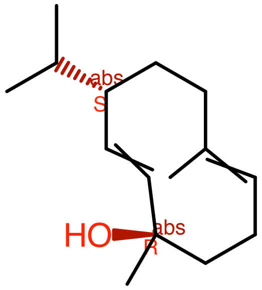	N399	A	0.75
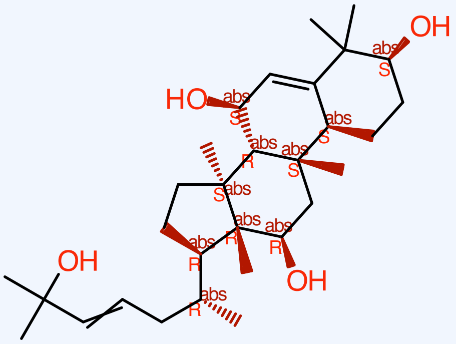	N579	A	0.25

Structure of Smiles	ID	Activity_Status	Consensus_score
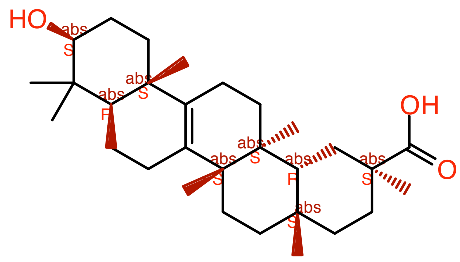	N601	A	0.25
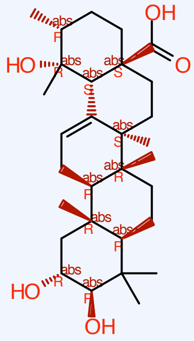	N431	A	0.5
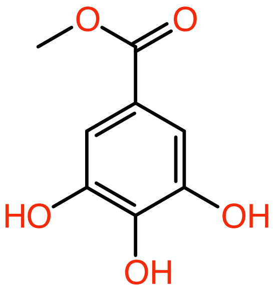	N444	A	1
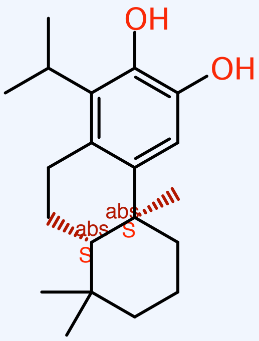	N476	A	0.5
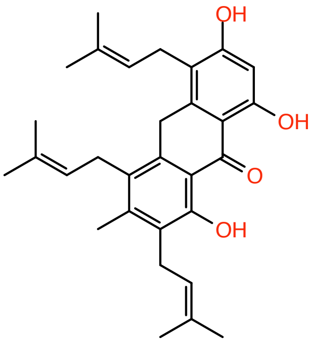	N563	A	0.25

Structure of Smiles	ID	Activity_Status	Consensus_score
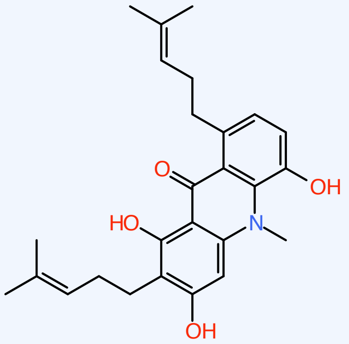	N481	A	0.25
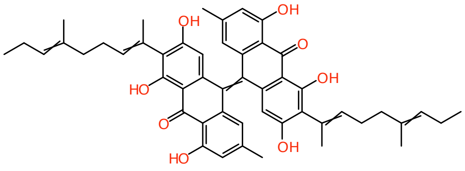	N477	A	-0.75
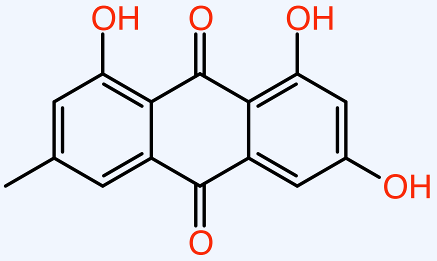	N585	A	1
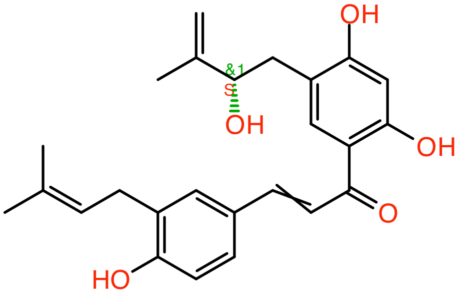	N456	A	0.25
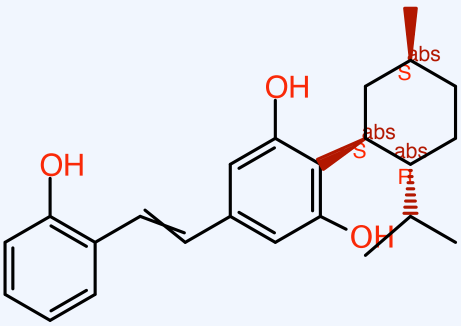	N424	A	0.25

Structure of Smiles	ID	Activity_Status	Consensus_score
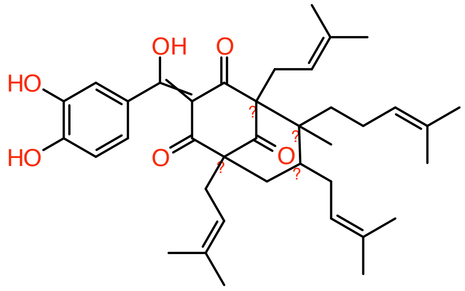	N522	A	-0.25
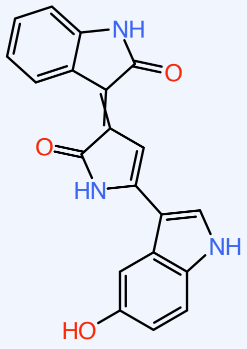	N332	A	1
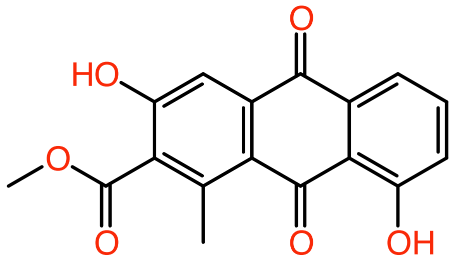	N583	A	0.75
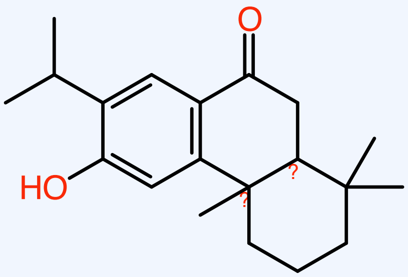	N374	A	0.75
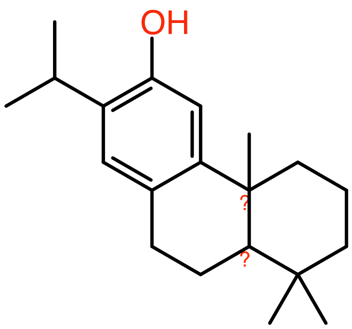	N533	A	0.5

Structure of Smiles	ID	Activity_Status	Consensus_score
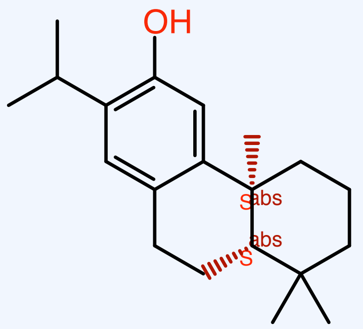	N541	A	0.5
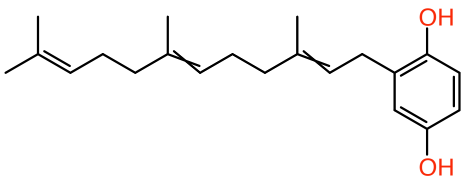	N371	A	0.25
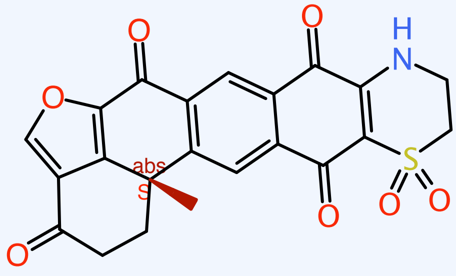	N572	A	1
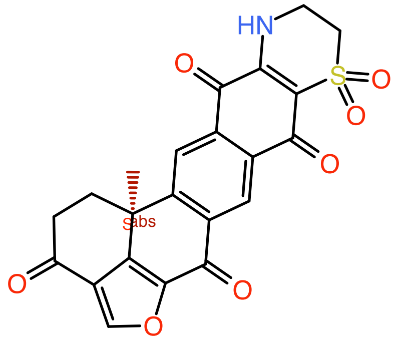	N343	A	1
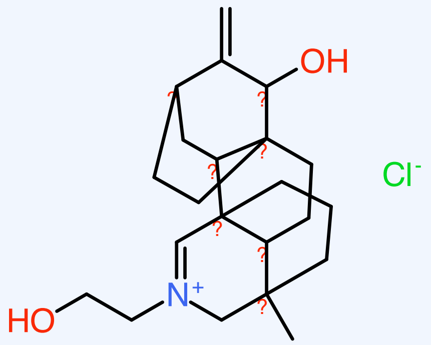	N551	A	0.75

Structure of Smiles	ID	Activity_Status	Consensus_score
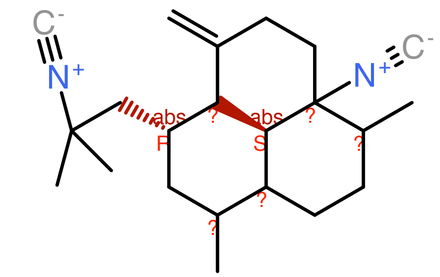	N335	A	0.75
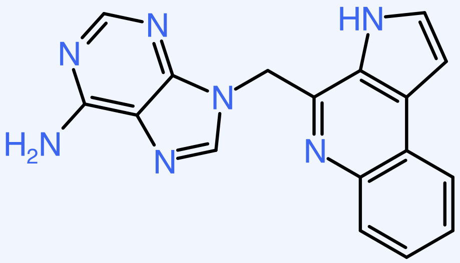	N17	A	1
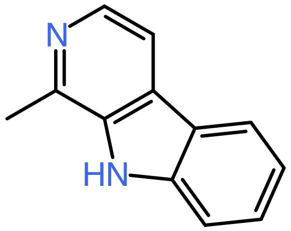	N387	A	1
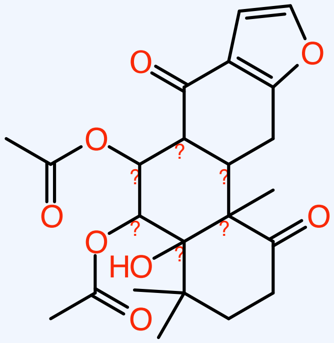	N328	A	0.75
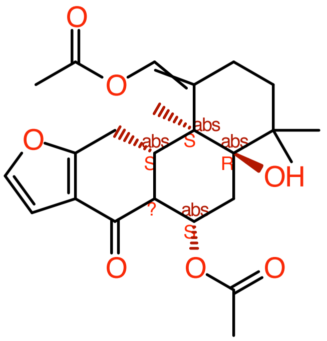	N518	A	0.75

Structure of Smiles	ID	Activity_Status	Consensus_score
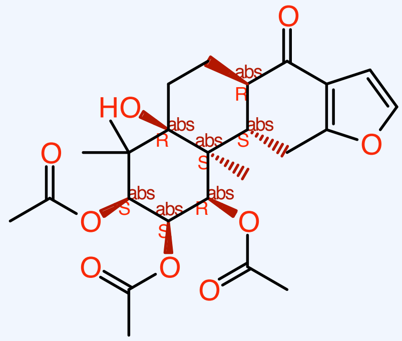	N438	A	0.75
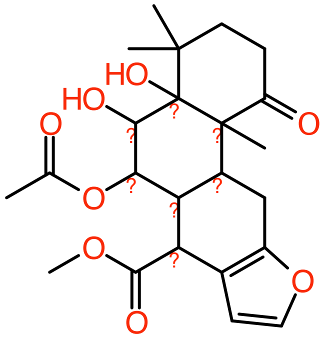	N407	A	0.75
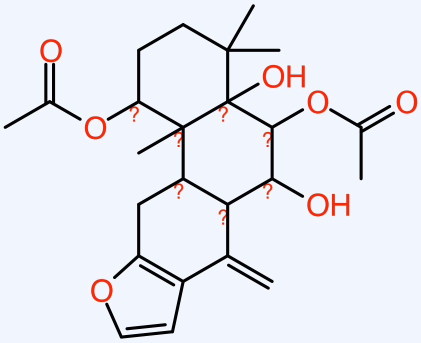	N568	A	0.75
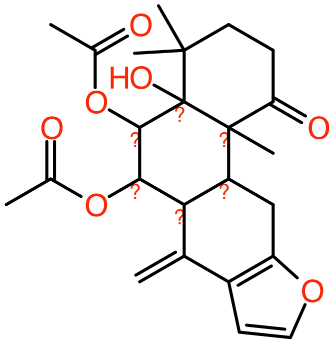	N492	A	0.75
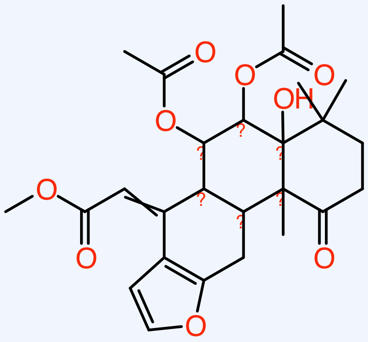	N327	A	0.75

Structure of Smiles	ID	Activity_Status	Consensus_score
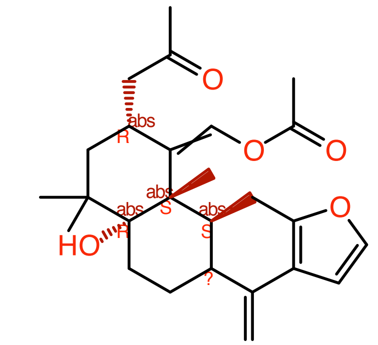	N417	A	0.75
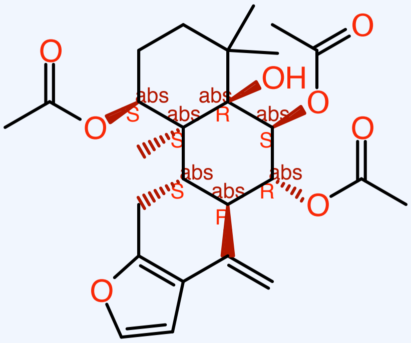	N537	A	0.75
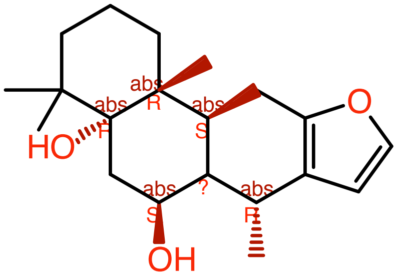	N450	A	0.75
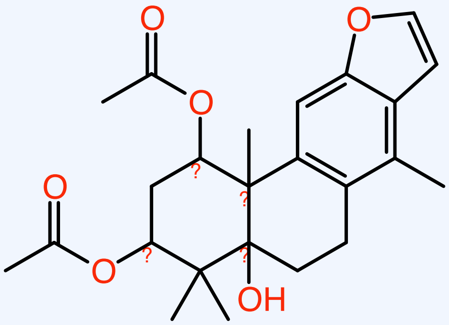	N584	A	0.75
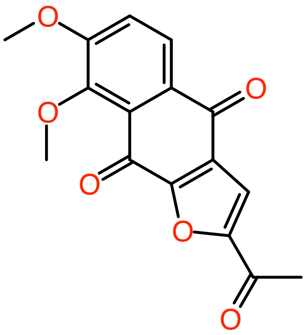	N496	A	1

Structure of Smiles	ID	Activity_Status	Consensus_score
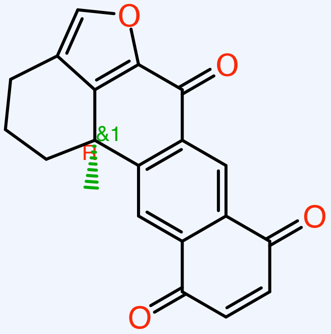	N346	A	1
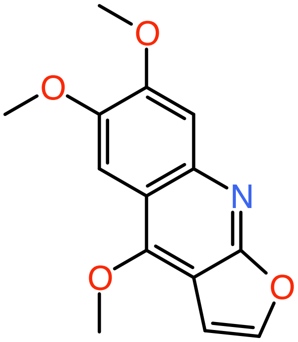	N511	A	1
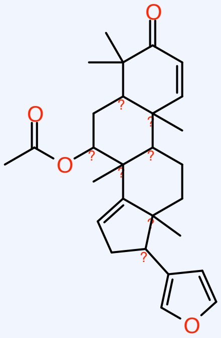	N19	A	0.25
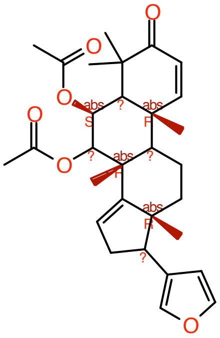	N596	A	0.25
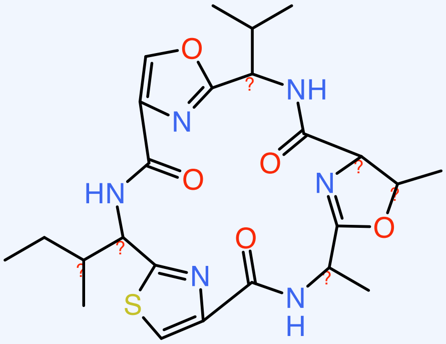	N465	A	0.25

Structure of Smiles	ID	Activity_Status	Consensus_score
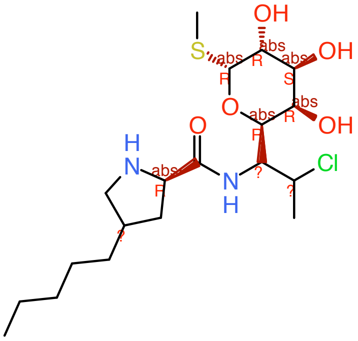	Mirincamycin	CRAD	0.75
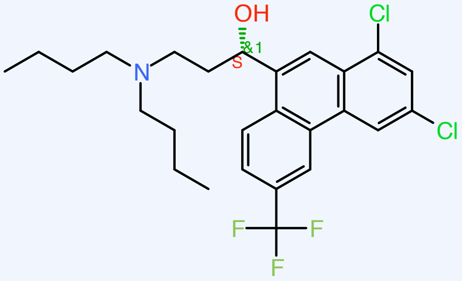	Halofantrine	CRAD	0
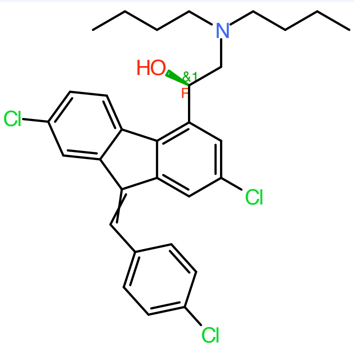	Lumefantrine	CRAD	0
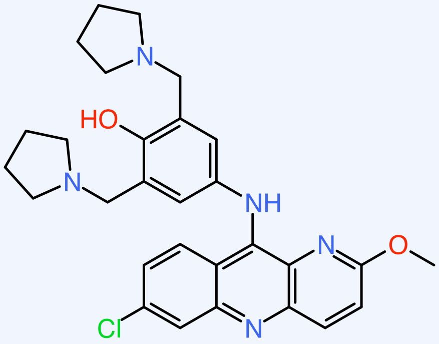	Pyronaridine	CRAD	0.5
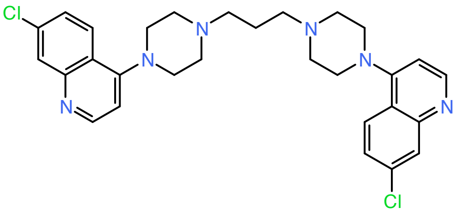	Piperaquine	CRAD	0.5

Structure of Smiles	ID	Activity_Status	Consensus_score
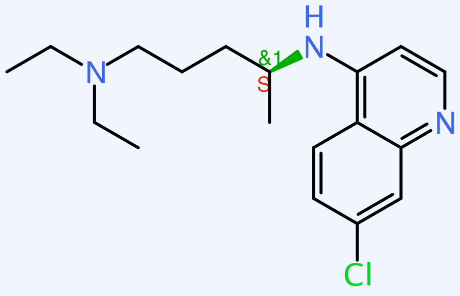	Chloroquine	CRAD	1
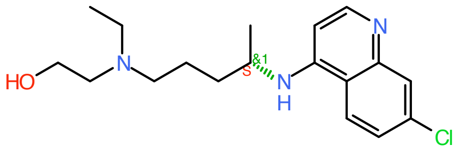	Hydroxychloroquine	CRAD	1
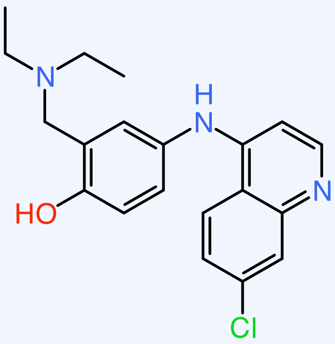	Amodiaquine	CRAD	1
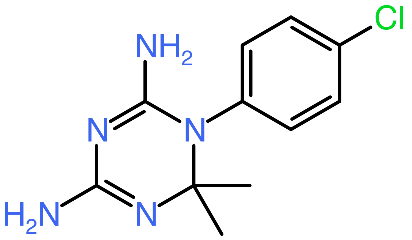	Cycloguanil Embonate	CRAD	1
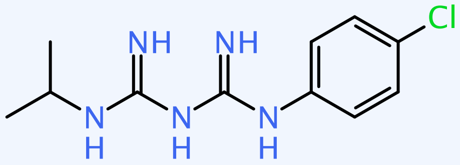	Proguanil	CRAD	0.75

Structure of Smiles	ID	Activity_Status	Consensus_score
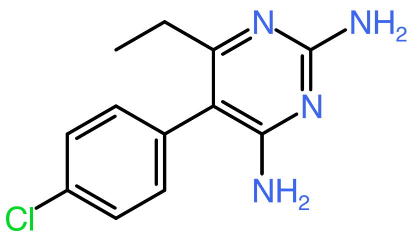	Pyrimethamine	CRAD	1
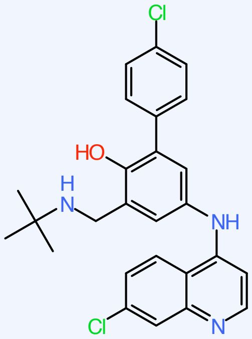	Tebuquine	CRAD	0.75
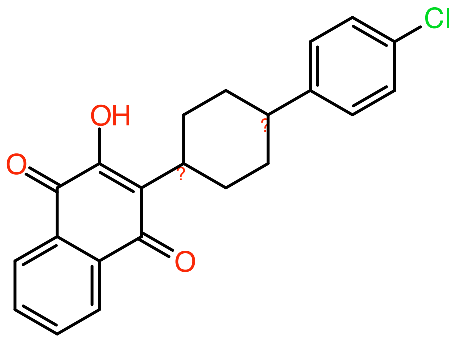	Atovaquone	CRAD	0.75
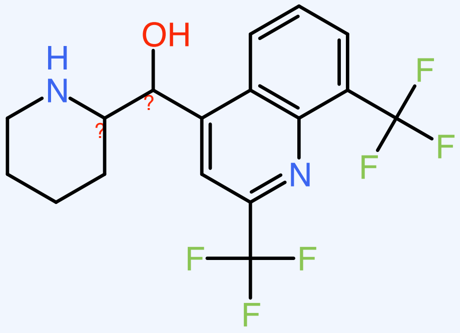	Mefloquine	CRAD	1
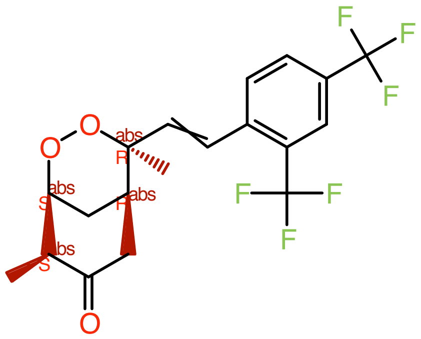	Arteflene	CRAD	0.5

Structure of Smiles	ID	Activity_Status	Consensus_score
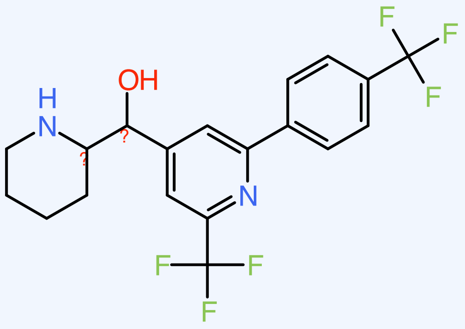	Enpiroline	CRAD	0.75
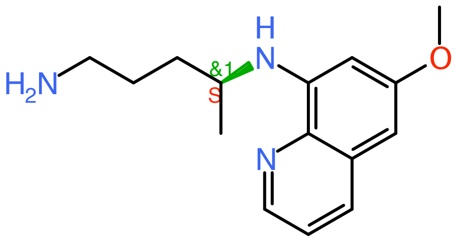	Primaquine	CRAD	1
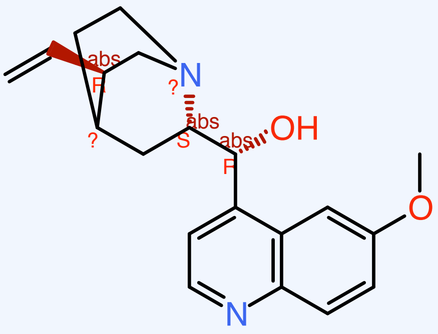	Quinine	CRAD	1
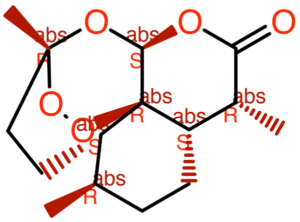	Artemisinin	CRAD	0.75
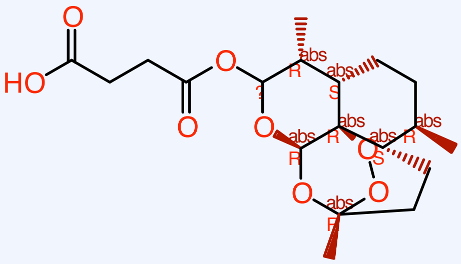	Artesunate	CRAD	0.75

Structure of Smiles	ID	Activity_Status	Consensus_score
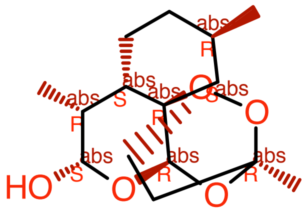	Artenimol	CRAD	0.75
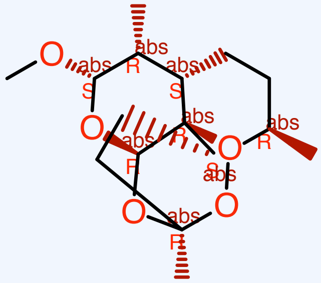	Artemether	CRAD	0.75
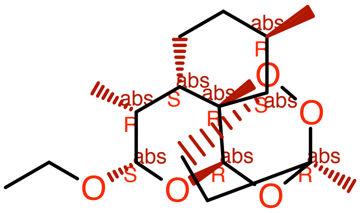	Artemotil	CRAD	0.75
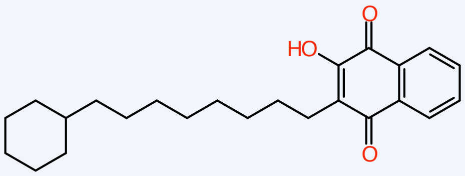	Menoctone	CRAD	0.5
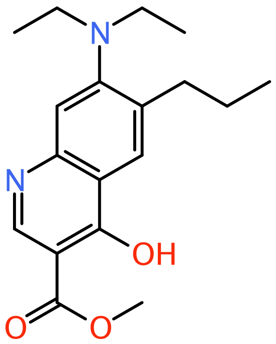	Amquinate	CRAD	1

Structure of Smiles	ID	Activity_Status	Consensus_score
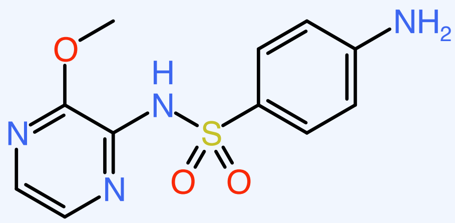	Sulfametopyrazine	CRAD	1
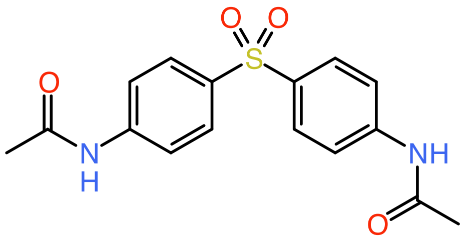	Acedapsone	CRAD	1
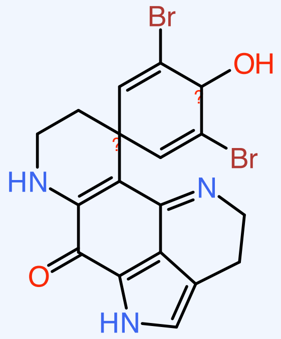	N163	HA	0.75
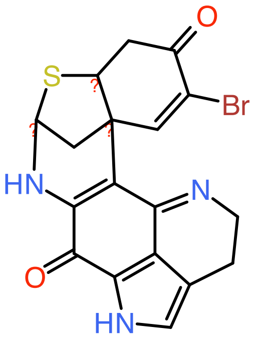	N130	HA	1
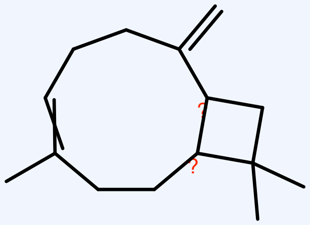	N232	HA	0.5

Structure of Smiles	ID	Activity_Status	Consensus_score
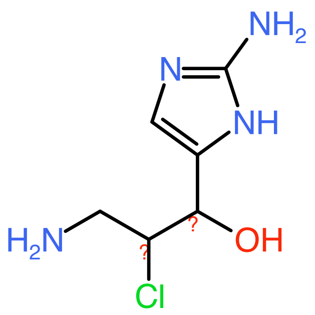	N192	HA	1
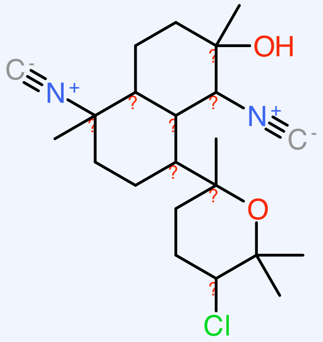	N79	HA	0.75
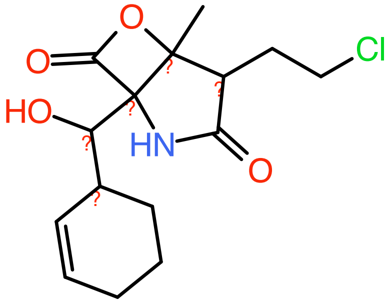	N100	HA	0.75
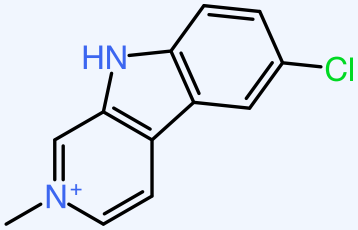	N270	HA	1
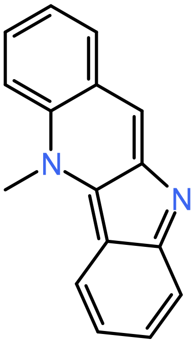	N164	HA	1

Structure of Smiles	ID	Activity_Status	Consensus_score
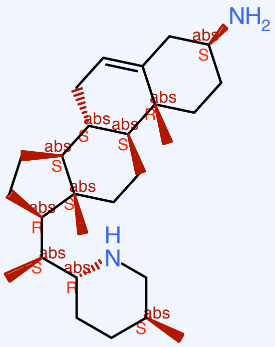	N118	HA	0.25
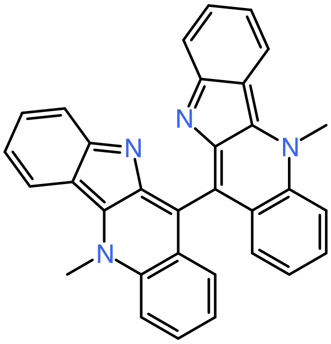	N211	HA	0.5
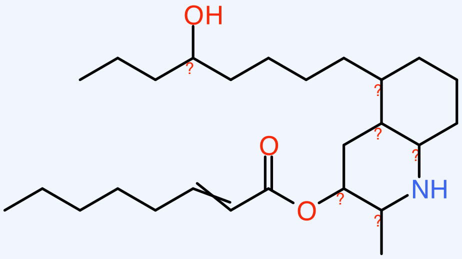	N253	HA	0
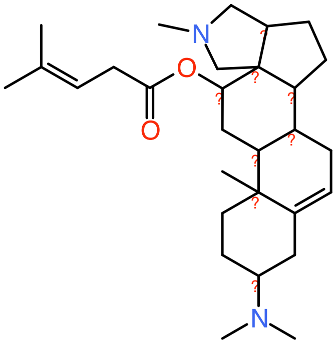	N321	HA	0.25
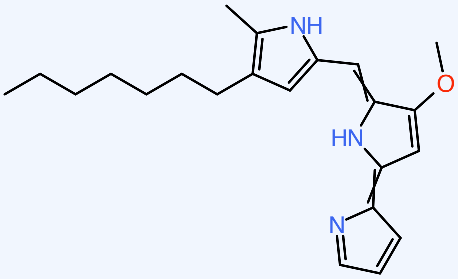	N124	HA	0.75

Structure of Smiles	ID	Activity_Status	Consensus_score
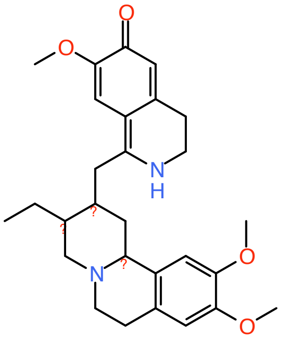	N221	HA	0.5
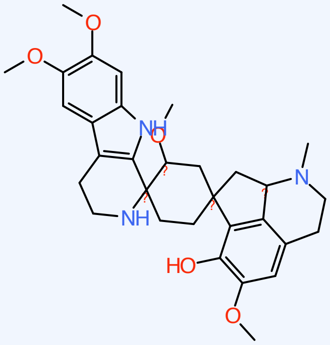	N298	HA	0.25
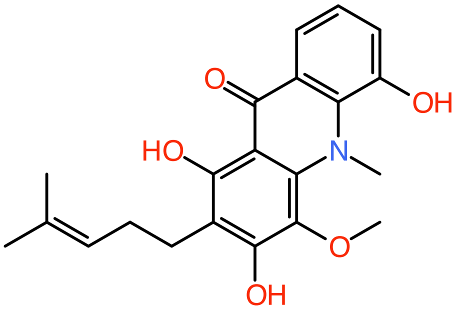	N218	HA	0.75
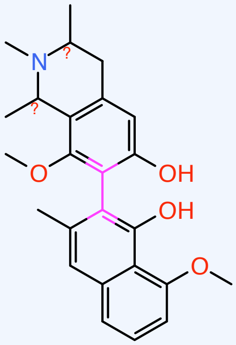	N200	HA	0.5
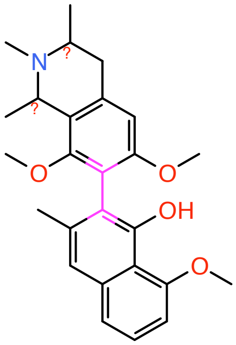	N196	HA	0.25

Structure of Smiles	ID	Activity_Status	Consensus_score
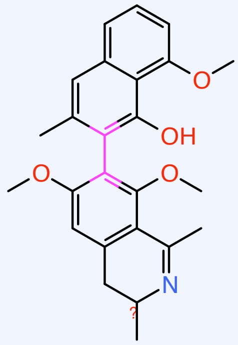	N201	HA	0.25
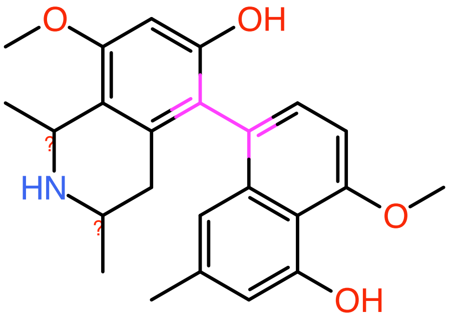	N266	HA	0.5
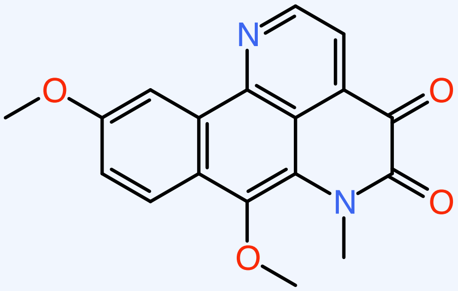	N242	HA	0.75
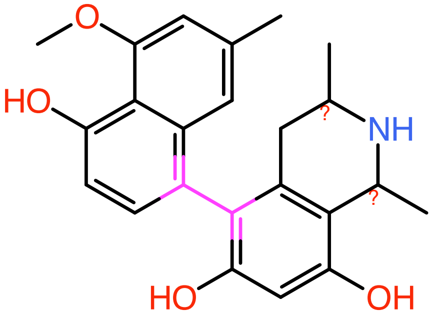	N136	HA	0.75
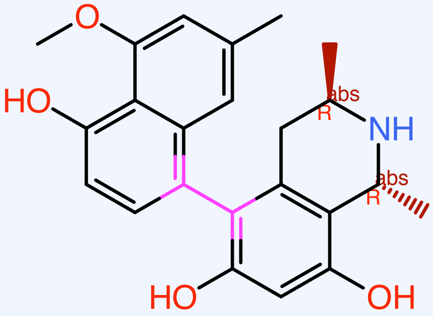	N254	HA	0.75

Structure of Smiles	ID	Activity_Status	Consensus_score
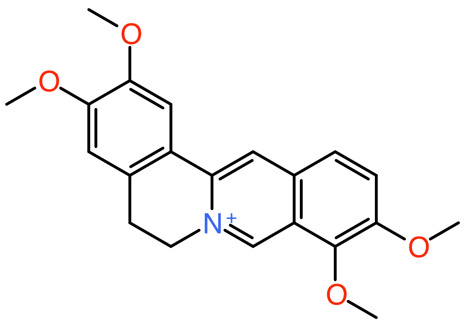	N302	HA	1
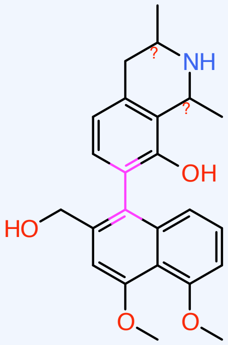	N103	HA	0.75
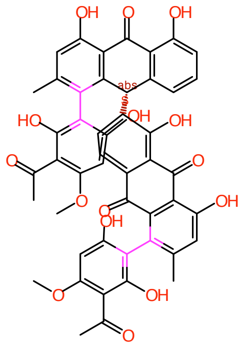	N173	HA	-0.75
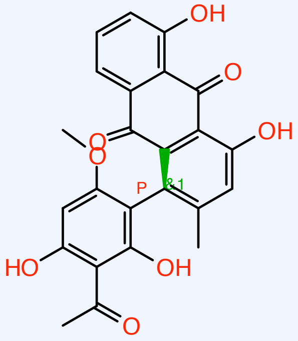	N213	HA	0.5
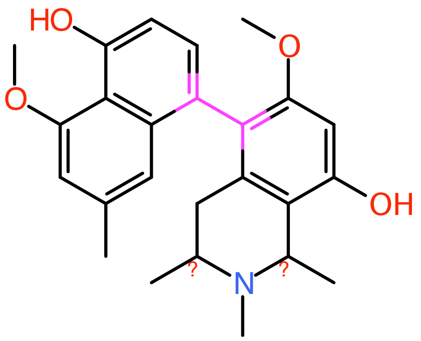	N230	HA	0.5

Structure of Smiles	ID	Activity_Status	Consensus_score
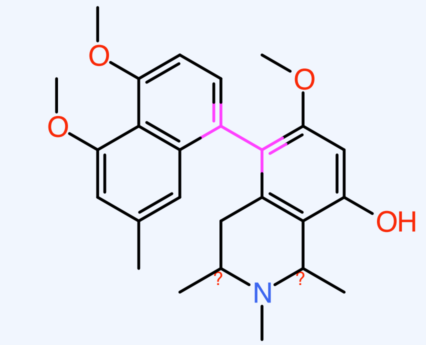	N235	HA	0.25
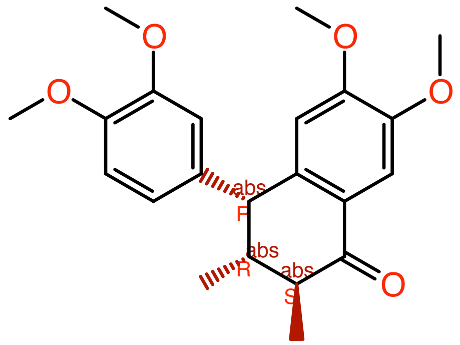	N223	HA	1
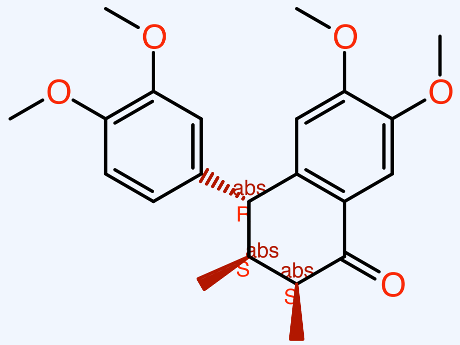	N282	HA	1
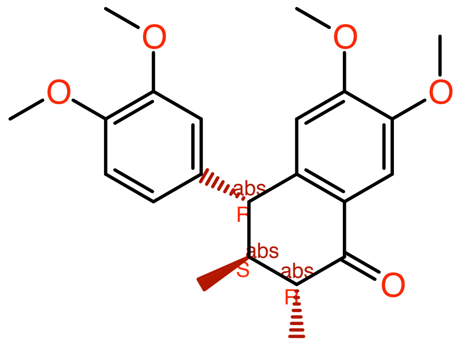	N187	HA	1
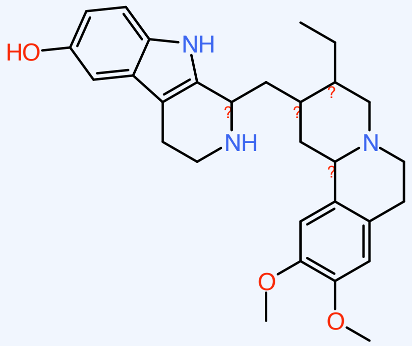	N102	HA	0.5

Structure of Smiles	ID	Activity_Status	Consensus_score
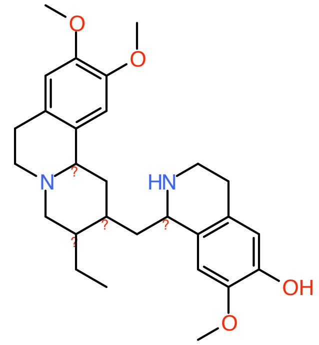	N135	HA	0.75
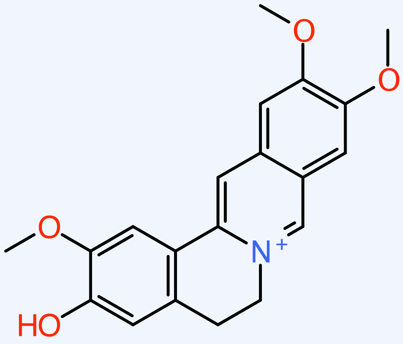	N285	HA	1
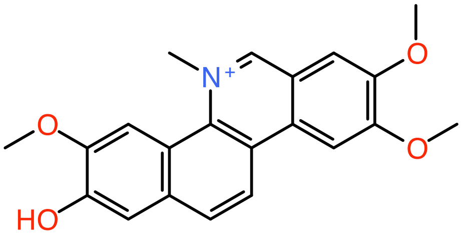	N129	HA	0.75
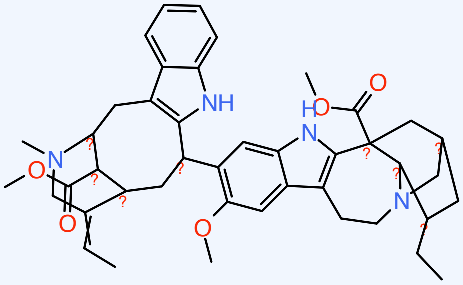	N228	HA	0
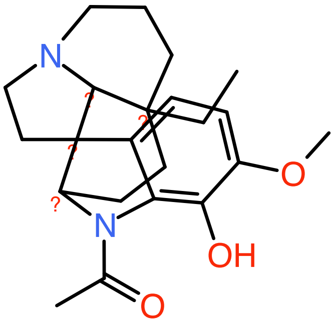	N111	HA	0.75

Structure of Smiles	ID	Activity_Status	Consensus_score
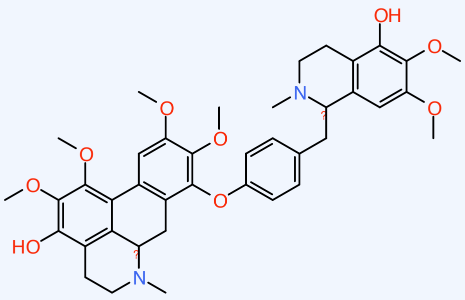	N229	HA	-0.25
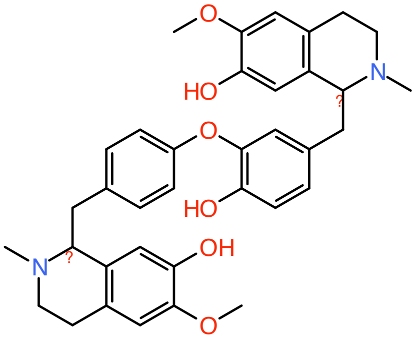	N171	HA	0
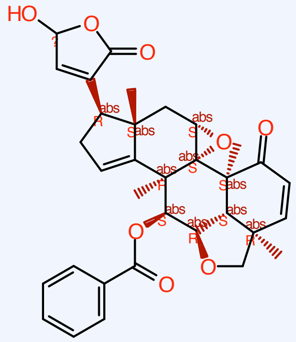	N237	HA	0.5
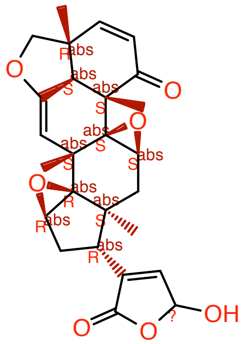	N292	HA	0.75
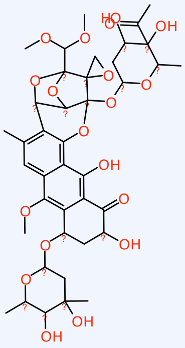	N84	HA	0

Structure of Smiles	ID	Activity_Status	Consensus_score
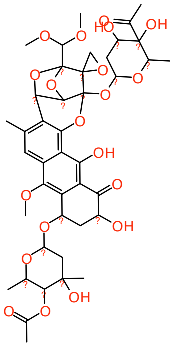	N83	HA	-0.25
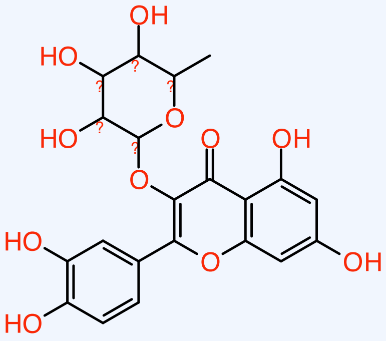	N86	HA	0.25
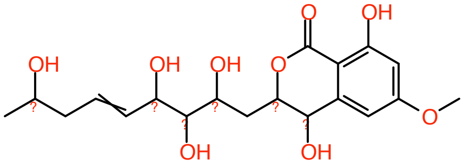	N99	HA	0.25
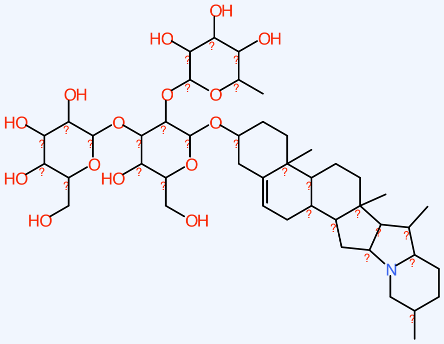	N249	HA	0
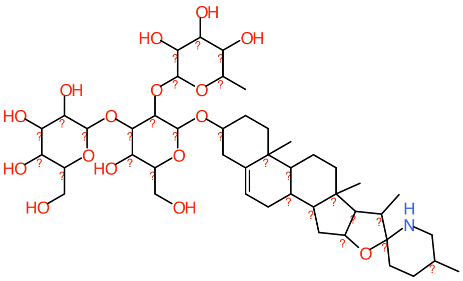	N272	HA	0

Structure of Smiles	ID	Activity_Status	Consensus_score
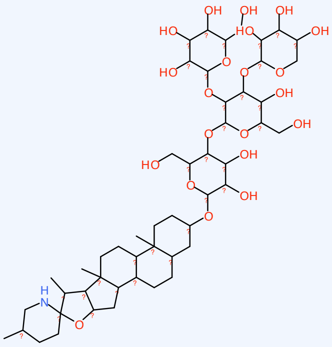	N290	HA	-0.5
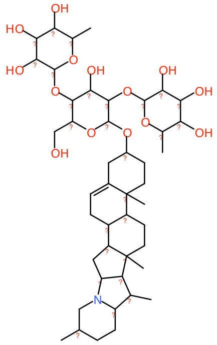	N299	HA	0
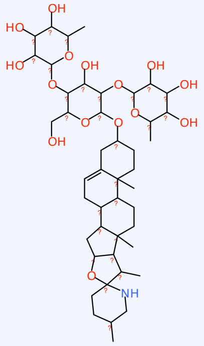	N287	HA	0
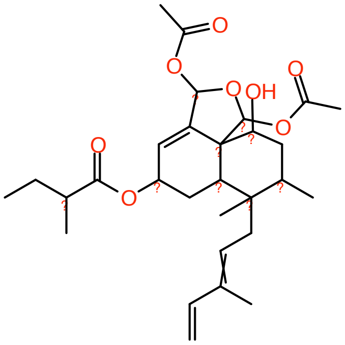	N281	HA	-0.25
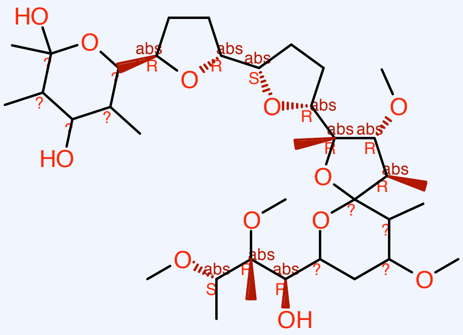	N215	HA	-0.25

Structure of Smiles	ID	Activity_Status	Consensus_score
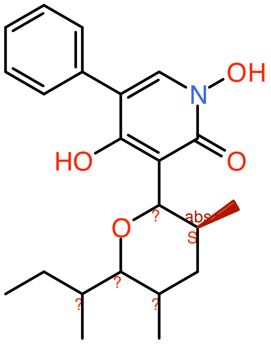	N260	HA	1
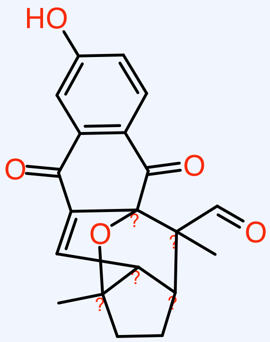	N202	HA	0.75
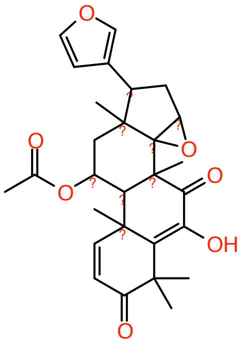	N174	HA	0.75
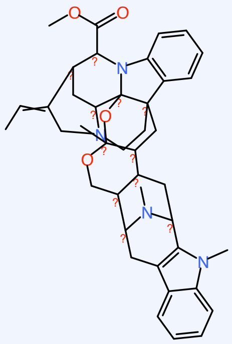	N209	HA	0.25
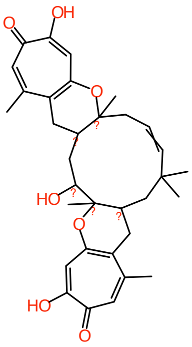	N240	HA	0.25

Structure of Smiles	ID	Activity_Status	Consensus_score
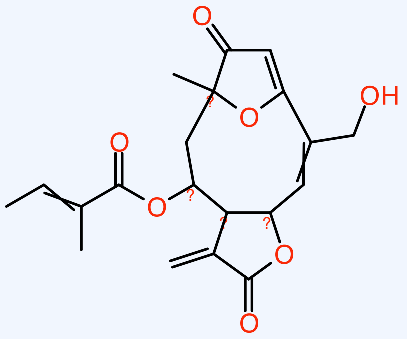	N310	HA	0.75
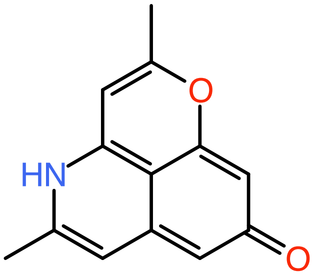	N117	HA	0.75
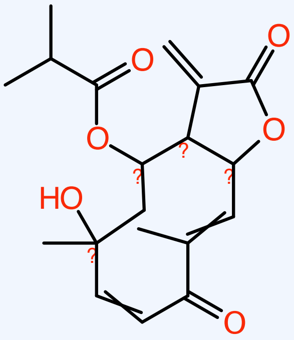	N318	HA	0.75
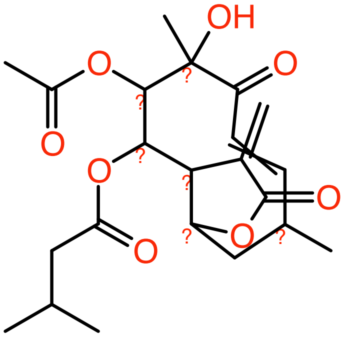	N280	HA	0.75
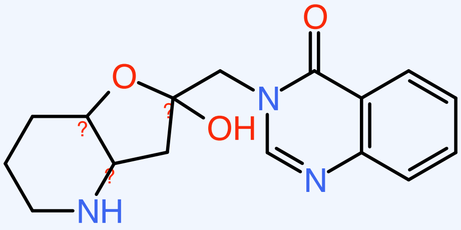	N80	HA	1

Structure of Smiles	ID	Activity_Status	Consensus_score
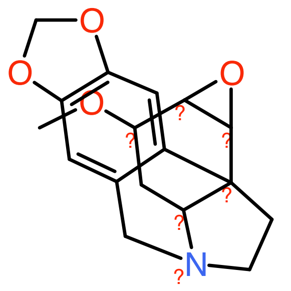	N251	HA	0.75
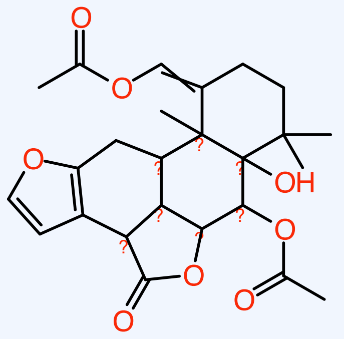	N303	HA	0.75
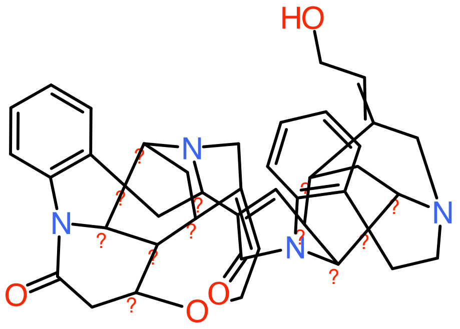	N141	HA	0.5
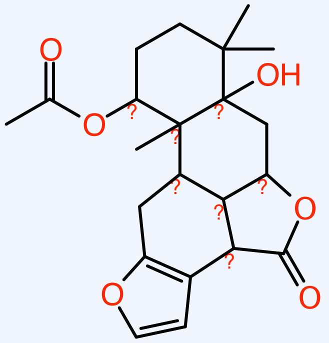	N309	HA	0.75
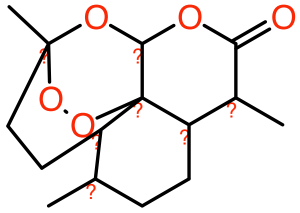	N121	HA	0.75

Structure of Smiles	ID	Activity_Status	Consensus_score
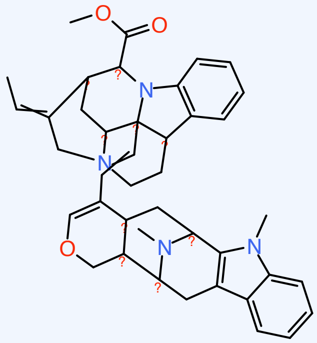	N238	HA	0
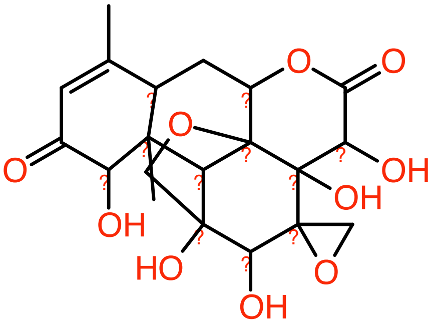	N126	HA	0
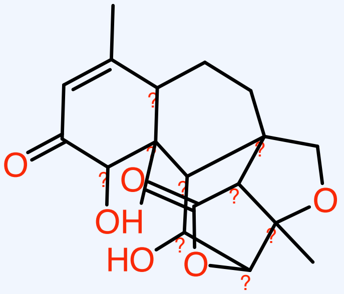	N189	HA	0.75
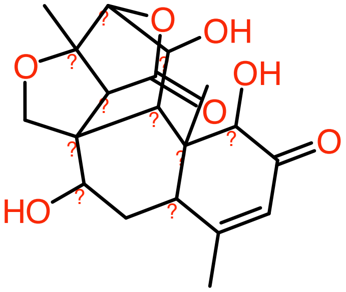	N283	HA	0.75
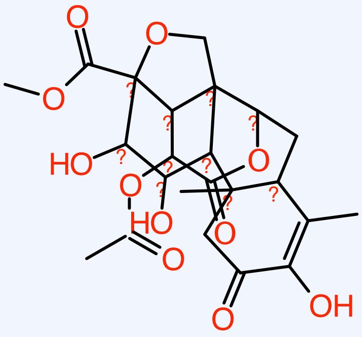	N116	HA	0.25

Structure of Smiles	ID	Activity_Status	Consensus_score
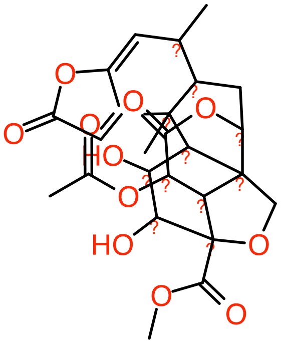	N87	HA	0
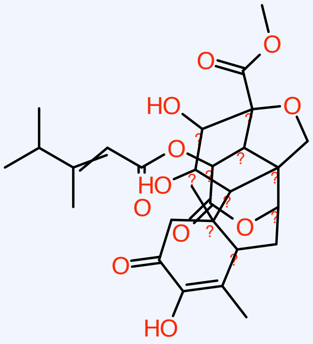	N82	HA	0
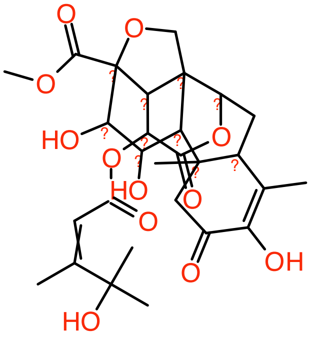	N95	HA	0
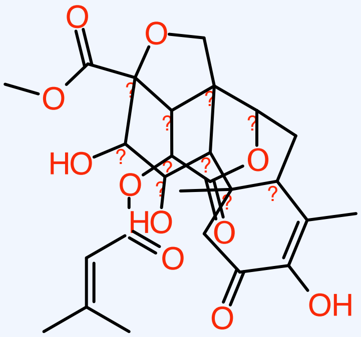	N90	HA	0
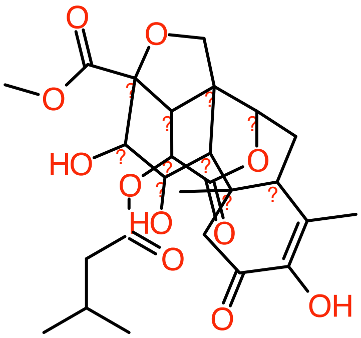	N115	HA	0

Structure of Smiles	ID	Activity_Status	Consensus_score
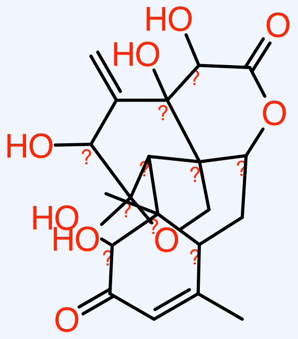	N150	HA	0.25
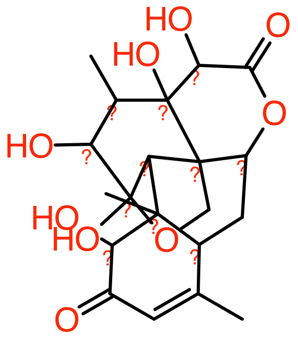	N193	HA	0.25
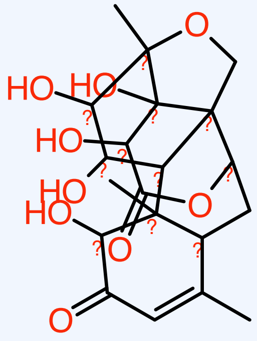	N122	HA	0.25
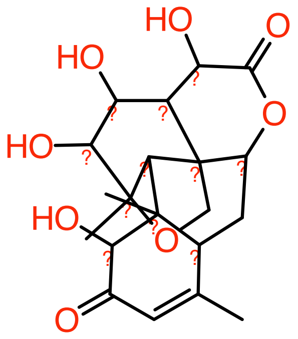	N137	HA	0.75
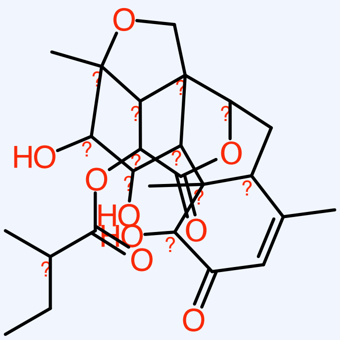	N92	HA	0.75

Structure of Smiles	ID	Activity_Status	Consensus_score
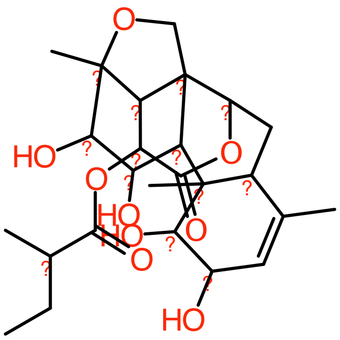	N91	HA	0.5
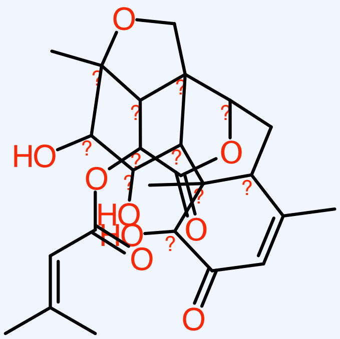	N153	HA	0.75
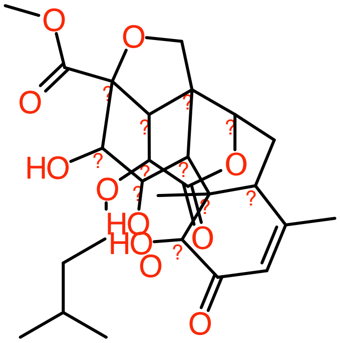	N127	HA	0
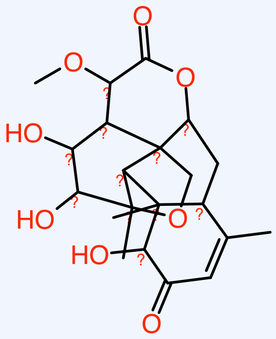	N106	HA	0.75
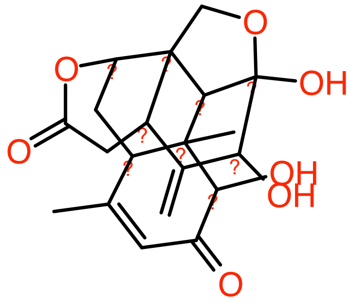	N94	HA	0.75

Structure of Smiles	ID	Activity_Status	Consensus_score
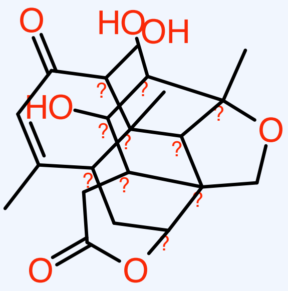	N190	HA	0.75
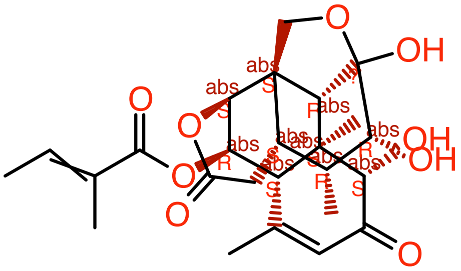	N161	HA	0.75
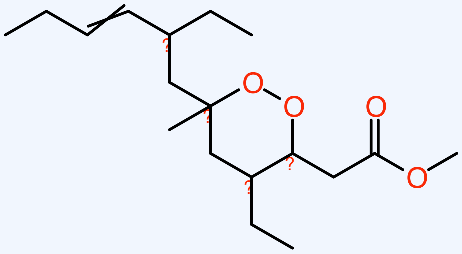	N245	HA	0.5
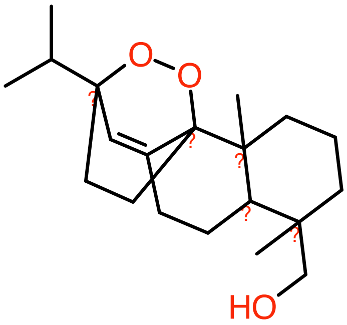	N151	HA	0.75
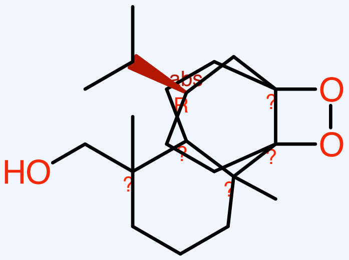	N222	HA	0.75

Structure of Smiles	ID	Activity_Status	Consensus_score
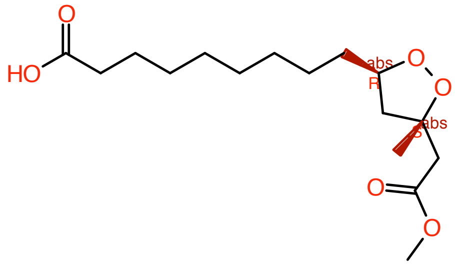	N217	HA	0.75
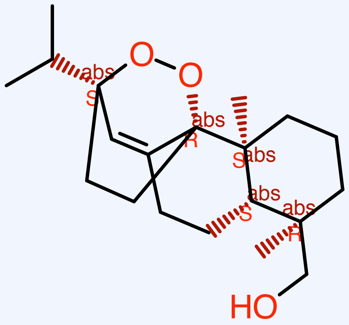	N231	HA	0.75
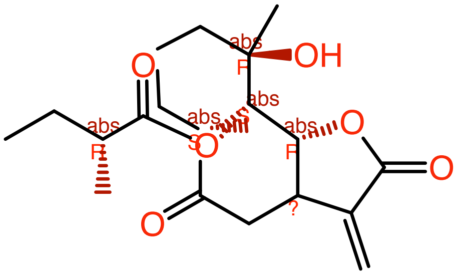	N108	HA	0.75
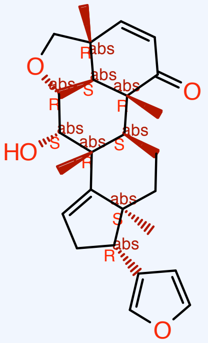	N268	HA	0.75
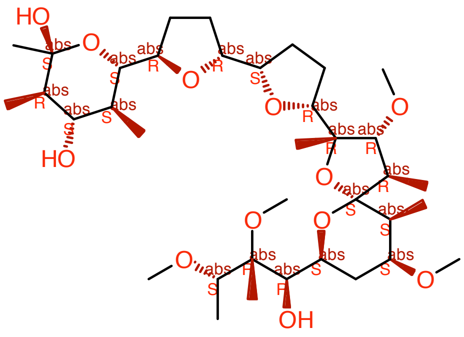	N216	HA	-0.25

Structure of Smiles	ID	Activity_Status	Consensus_score
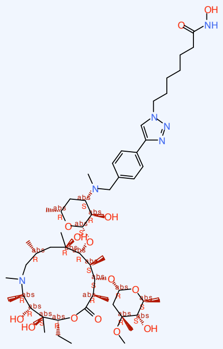	N149	HA	-0.5
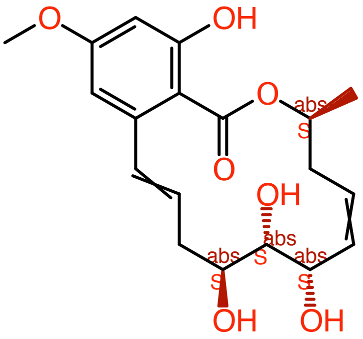	N114	HA	0.75
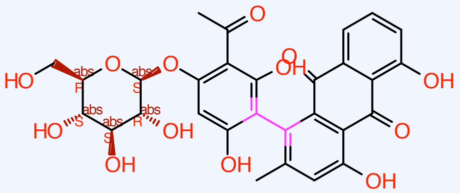	N297	HA	-0.25
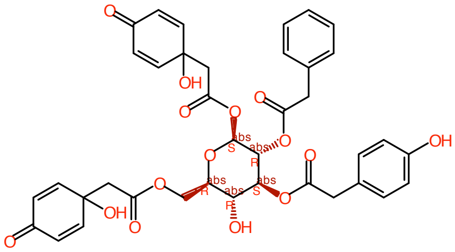	N304	HA	0
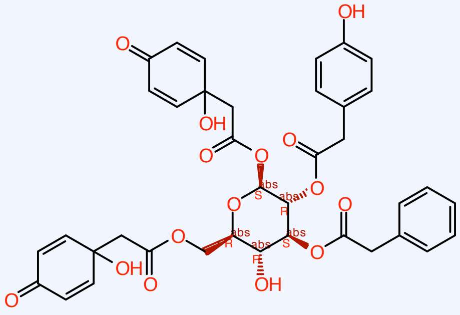	N305	HA	0

Structure of Smiles	ID	Activity_Status	Consensus_score
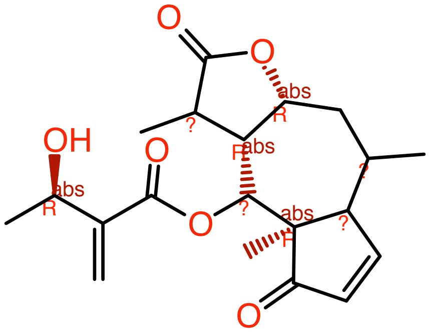	N248	HA	0.75
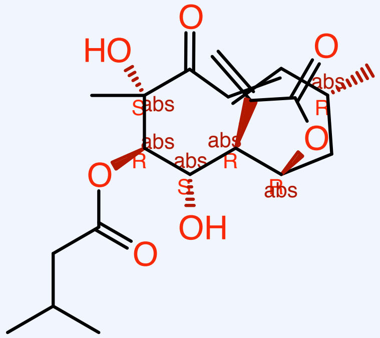	N279	HA	0.75
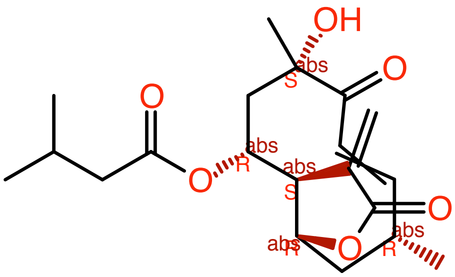	N317	HA	0.75
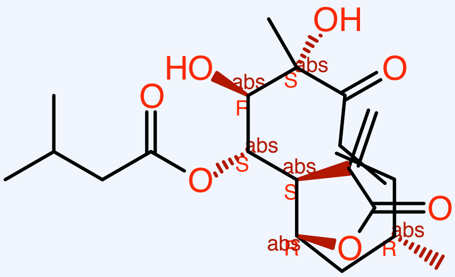	N278	HA	0.75
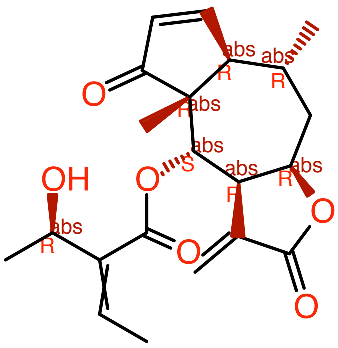	N182	HA	0.75

Structure of Smiles	ID	Activity_Status	Consensus_score
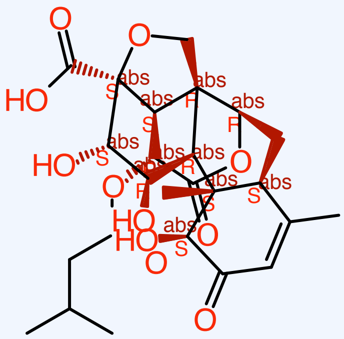	N277	HA	0
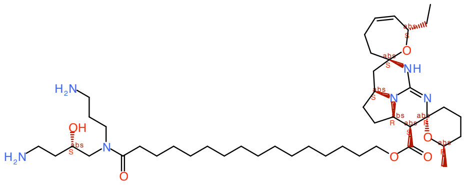	N198	HA	-0.75
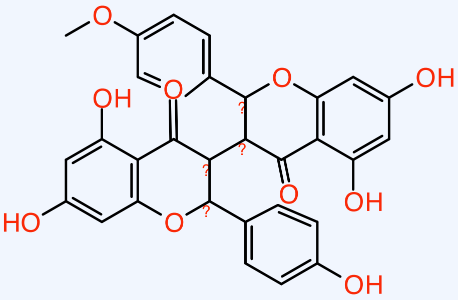	N322	HA	-0.5
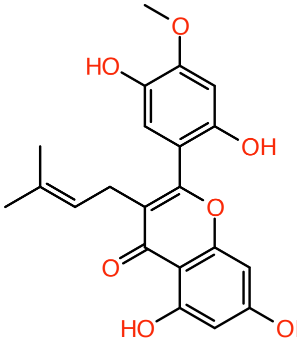	N160	HA	1
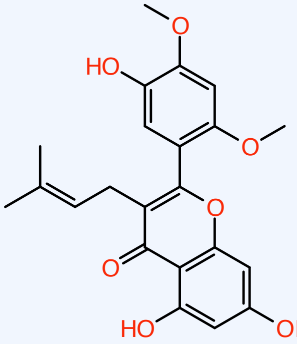	N291	HA	0.75

Structure of Smiles	ID	Activity_Status	Consensus_score
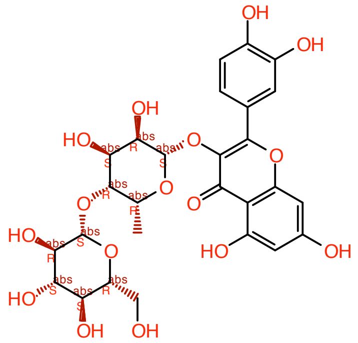	N89	HA	0
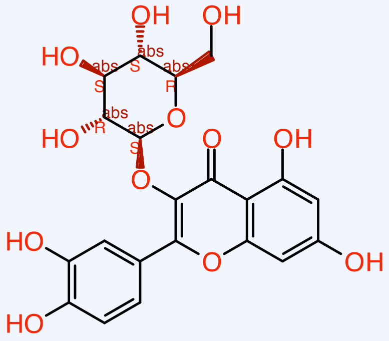	N88	HA	0.25
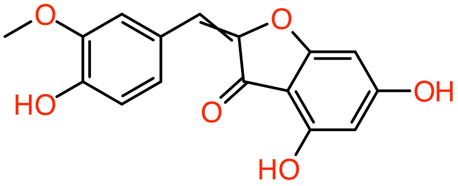	N120	HA	0.75
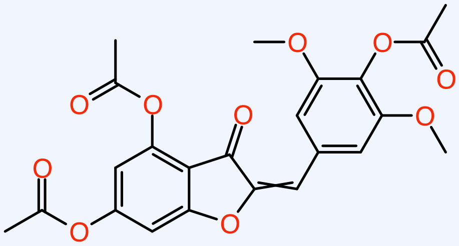	N93	HA	0.75
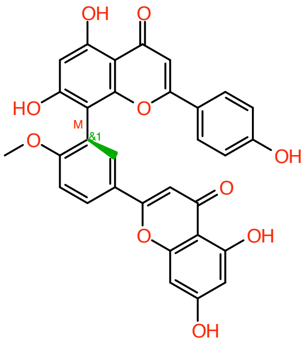	N219	HA	-0.25

Structure of Smiles	ID	Activity_Status	Consensus_score
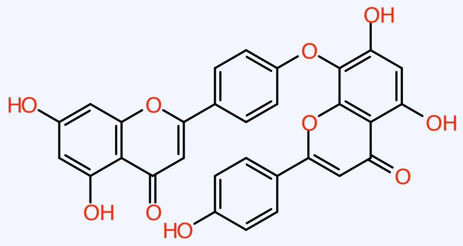	N255	HA	-0.25
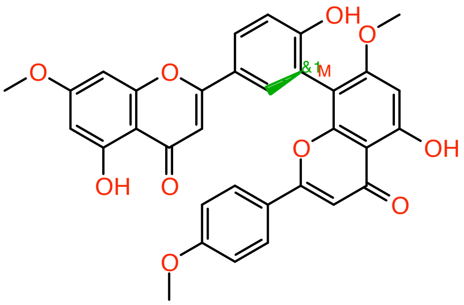	N203	HA	-0.25
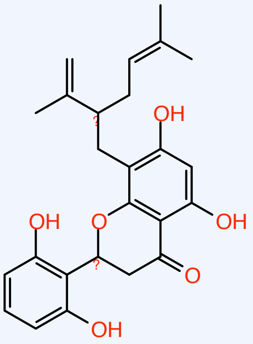	N98	HA	0.25
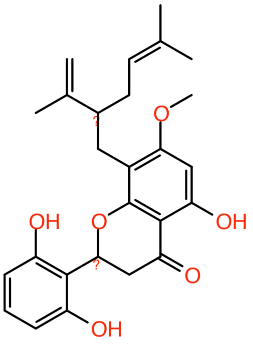	N96	HA	0.25
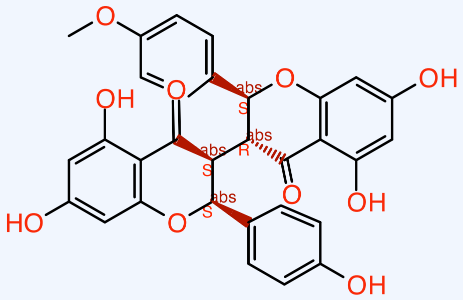	N320	HA	-0.5

Structure of Smiles	ID	Activity_Status	Consensus_score
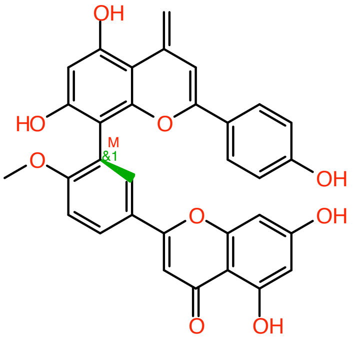	N220	HA	-0.25
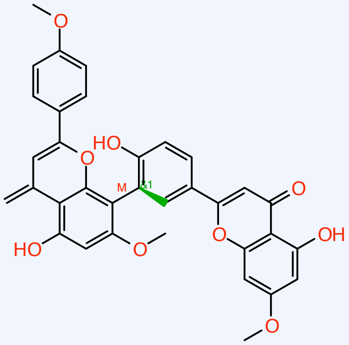	N206	HA	0.25
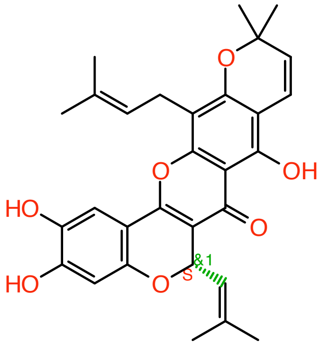	N113	HA	0
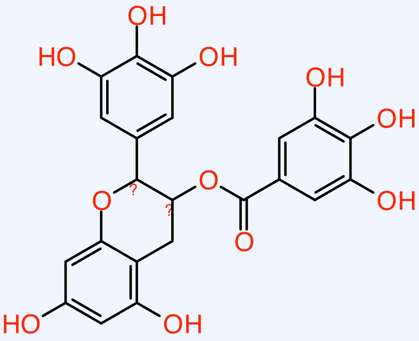	N139	HA	0.25
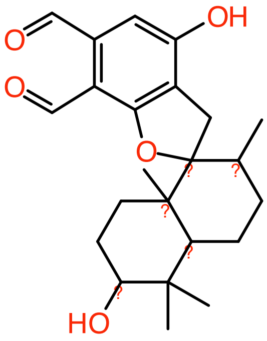	N243	HA	0.75

Structure of Smiles	ID	Activity_Status	Consensus_score
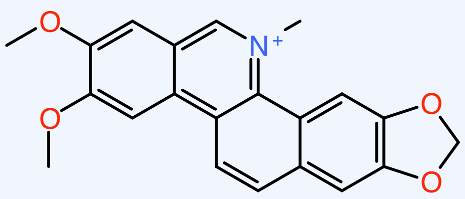	N157	HA	0.75
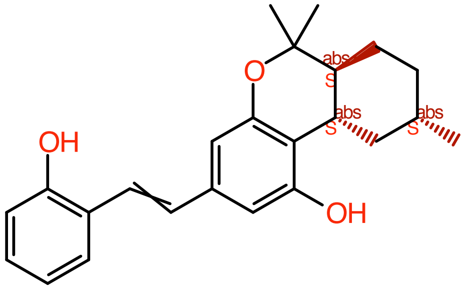	N225	HA	0.25
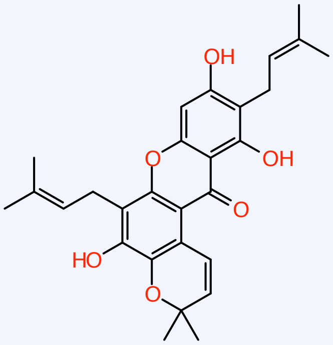	N295	HA	0.25
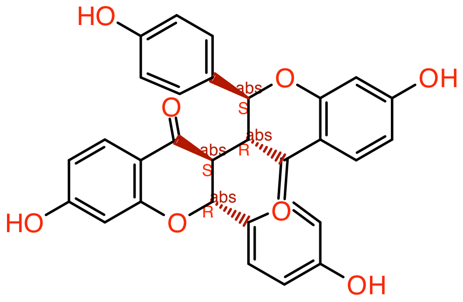	N13	HA	0.5
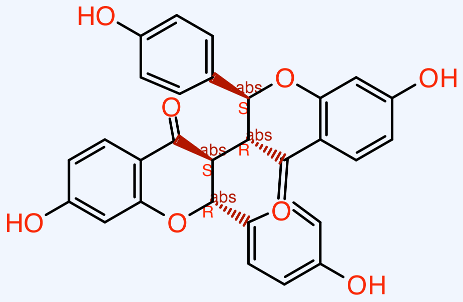	N169	HA	0.5

Structure of Smiles	ID	Activity_Status	Consensus_score
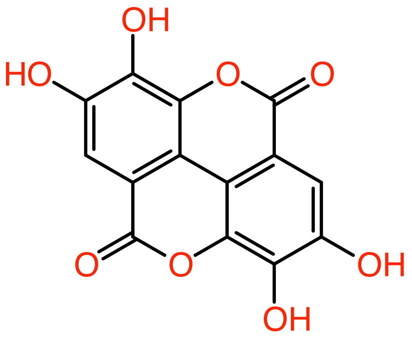	N12	HA	1
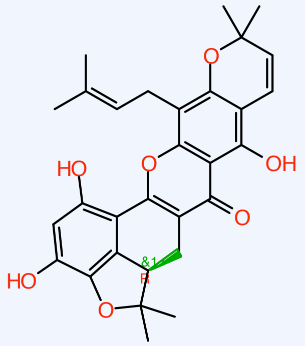	N267	HA	0
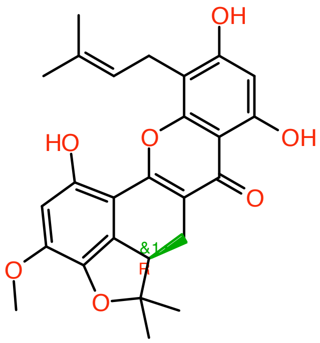	N179	HA	0.25
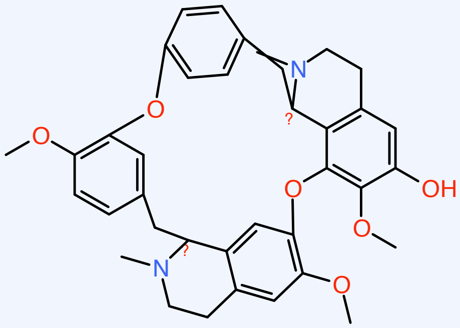	N191	HA	0
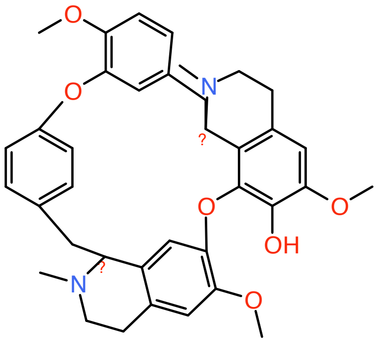	N142	HA	0

Structure of Smiles	ID	Activity_Status	Consensus_score
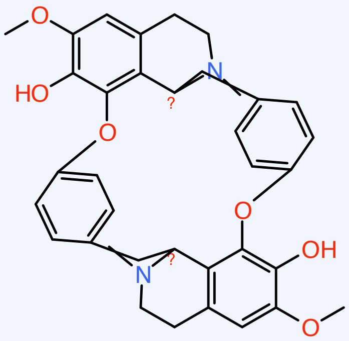	N314	HA	0
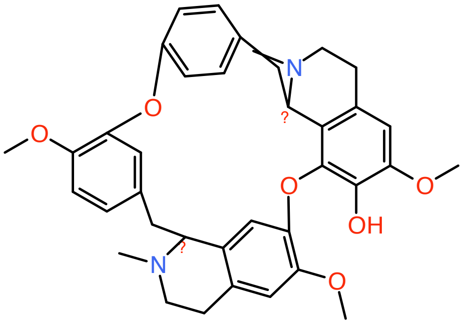	N176	HA	0
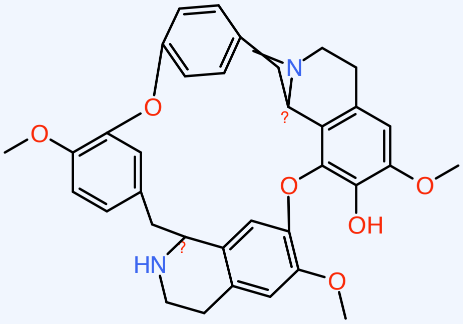	N154	HA	0
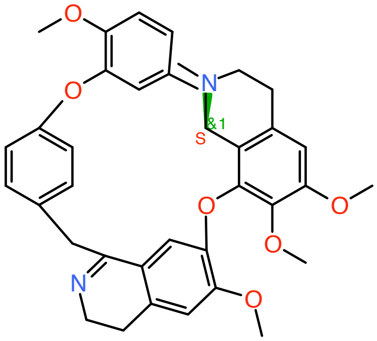	N175	HA	0.25
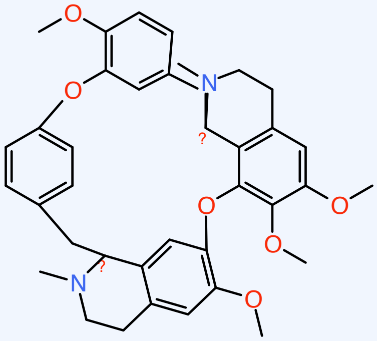	N207	HA	0

Structure of Smiles	ID	Activity_Status	Consensus_score
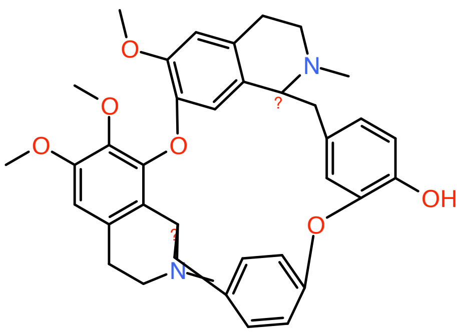	N264	HA	0
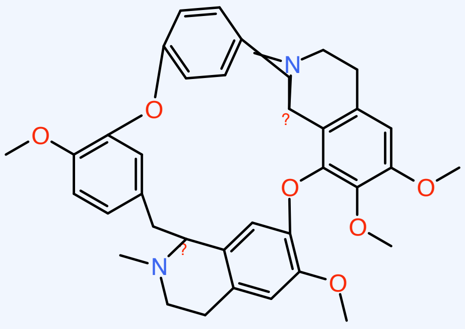	N241	HA	0
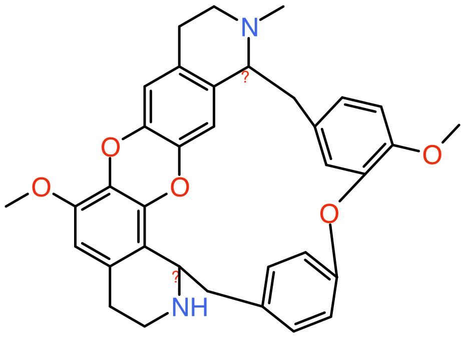	N177	HA	0
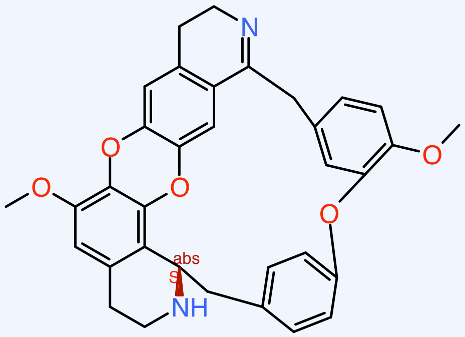	N269	HA	0.25
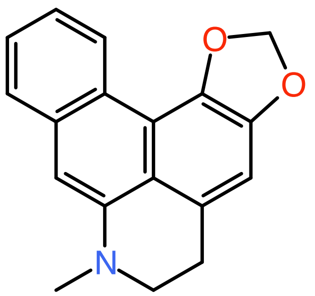	N239	HA	0.75

Structure of Smiles	ID	Activity_Status	Consensus_score
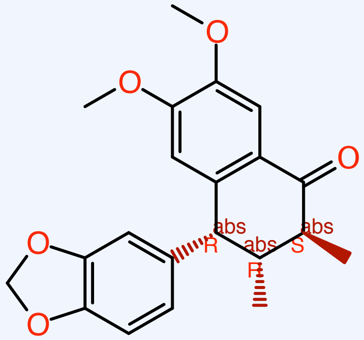	N205	HA	1
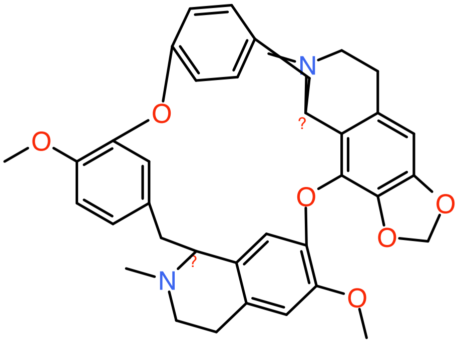	N194	HA	0
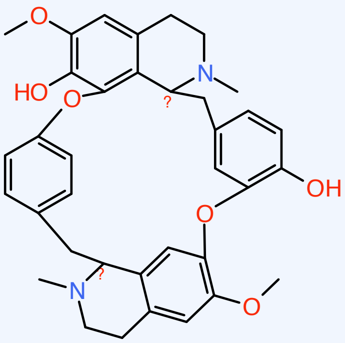	N234	HA	0
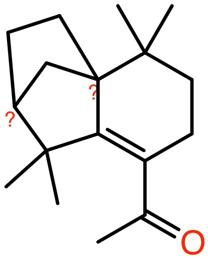	N167	HA	0.75
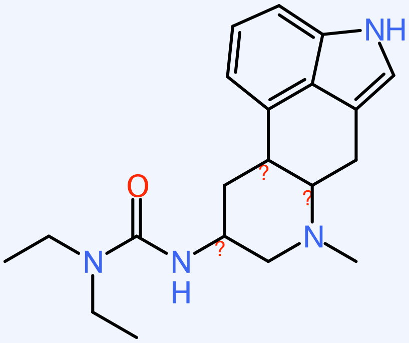	N261	HA	1

Structure of Smiles	ID	Activity_Status	Consensus_score
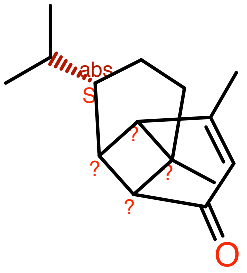	N284	HA	0.75
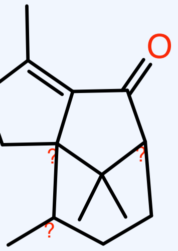	N180	HA	0.75
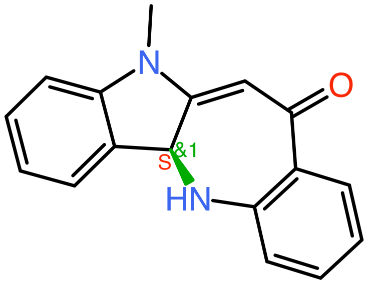	N313	HA	1
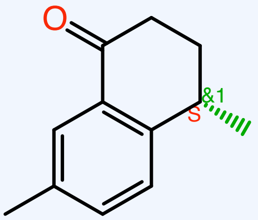	N143	HA	1
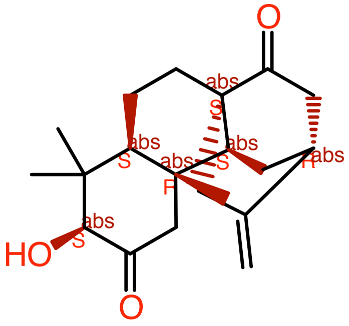	N107	HA	0.75

Structure of Smiles	ID	Activity_Status	Consensus_score
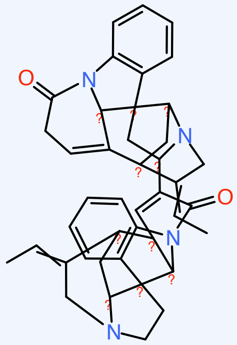	N185	HA	0
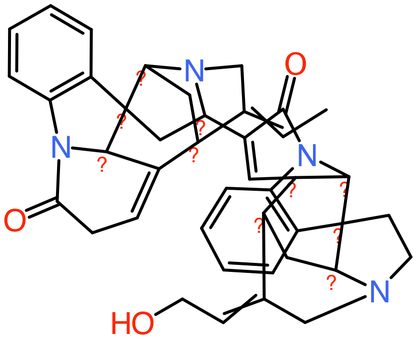	N152	HA	0.5
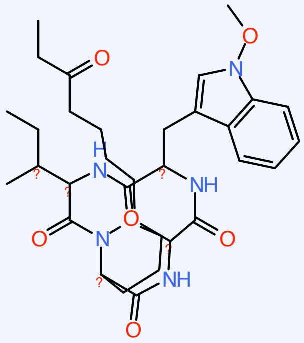	N134	HA	0
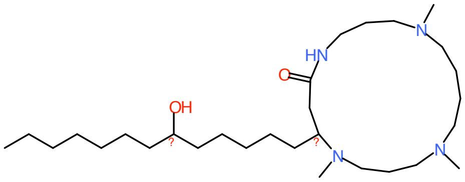	N214	HA	0
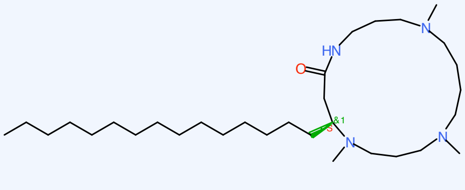	N178	HA	0

Structure of Smiles	ID	Activity_Status	Consensus_score
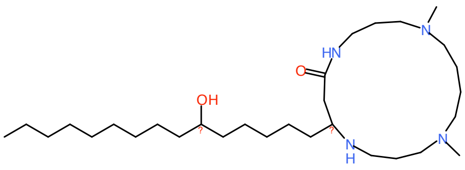	N226	HA	-0.25
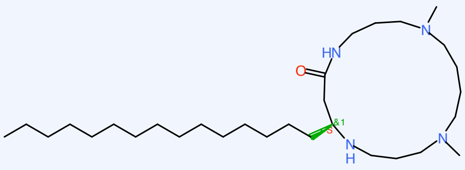	N186	HA	0.25
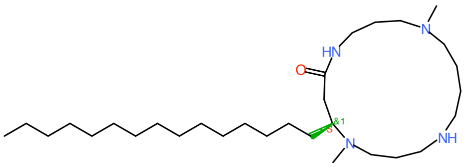	N199	HA	0.25
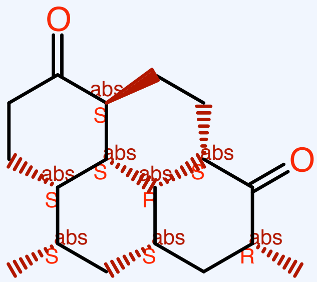	N1	HA	0.75
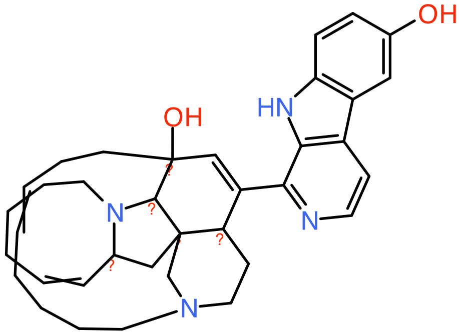	N300	HA	0

Structure of Smiles	ID	Activity_Status	Consensus_score
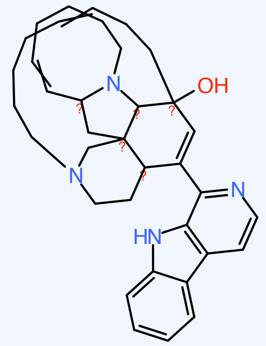	N101	HA	0
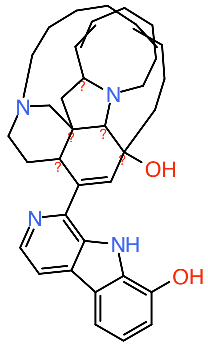	N97	HA	0
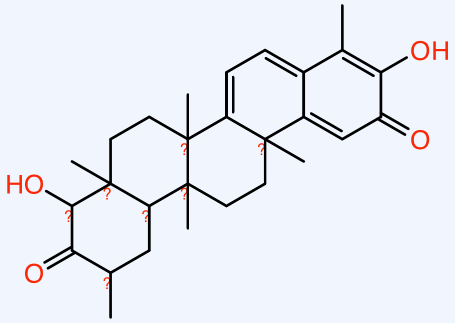	N311	HA	0.5
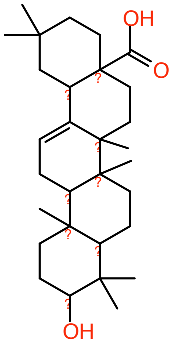	N123	HA	0.25
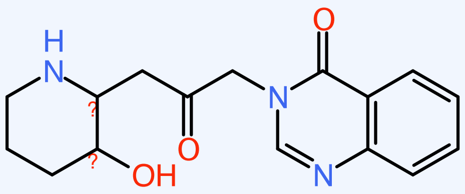	N78	HA	1

Structure of Smiles	ID	Activity_Status	Consensus_score
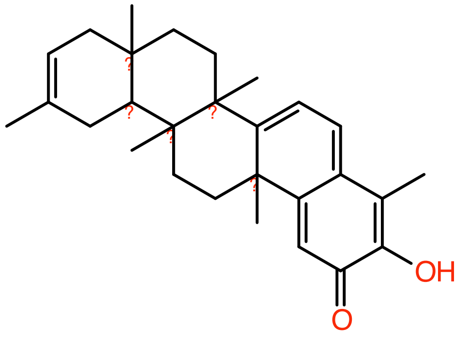	N195	HA	0.25
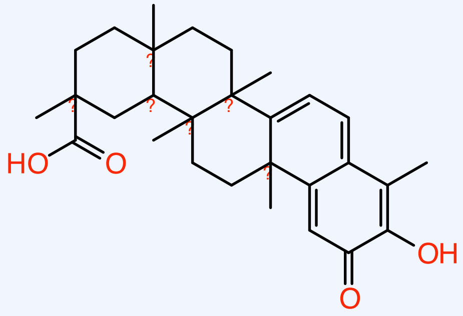	N246	HA	0.25
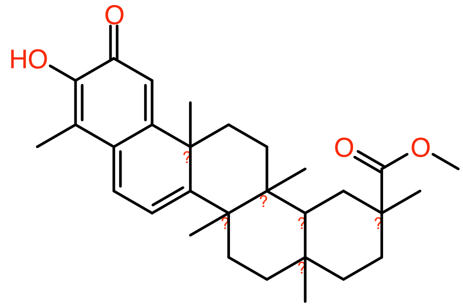	N247	HA	0.25
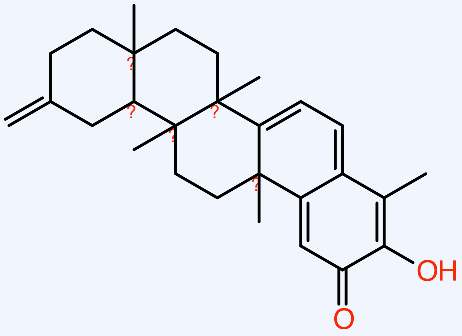	N250	HA	0.25
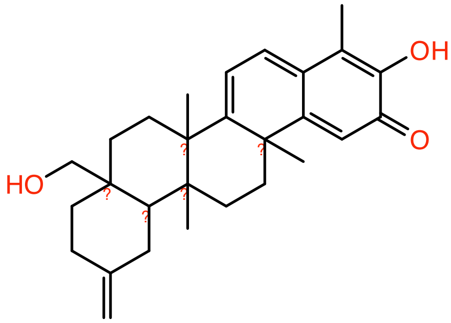	N212	HA	0.25

Structure of Smiles	ID	Activity_Status	Consensus_score
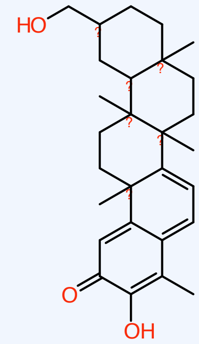	N133	HA	0.25
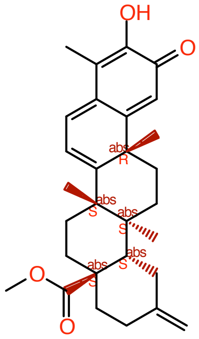	N119	HA	0.25
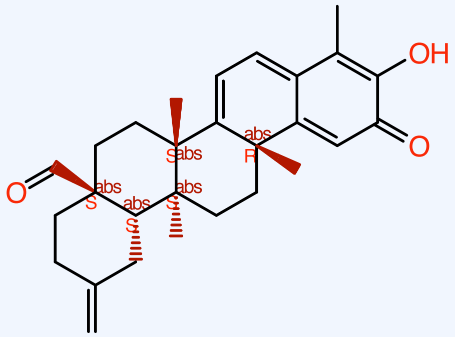	N148	HA	0.5
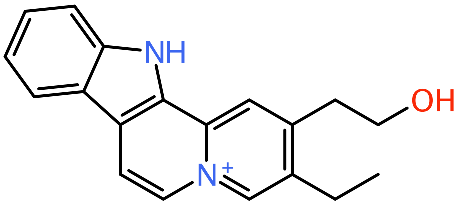	N156	HA	0.75
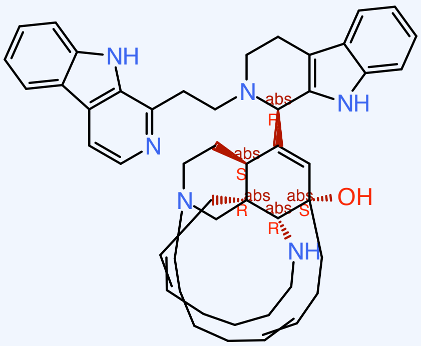	N306	HA	0

Structure of Smiles	ID	Activity_Status	Consensus_score
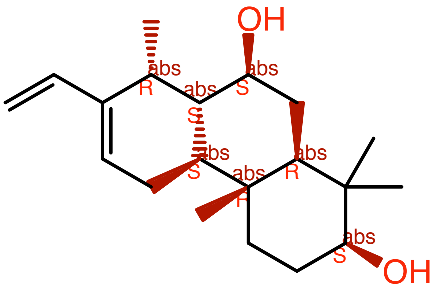	N288	HA	0.75
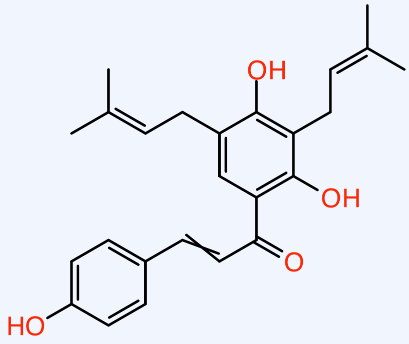	N307	HA	0.25
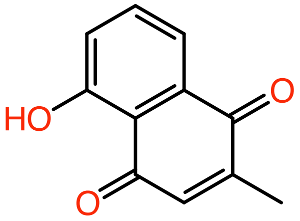	N210	HA	0.75
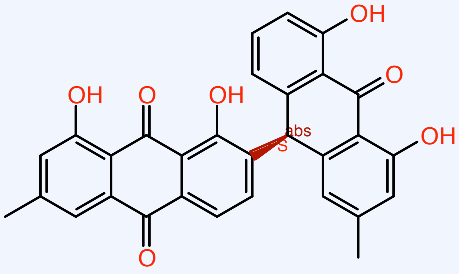	N257	HA	0.5
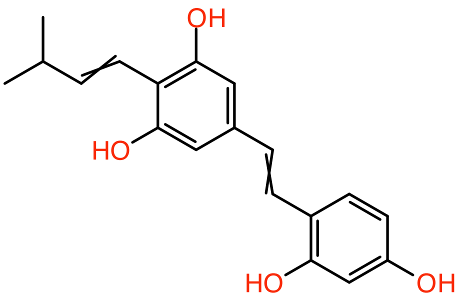	N286	HA	0.75

Structure of Smiles	ID	Activity_Status	Consensus_score
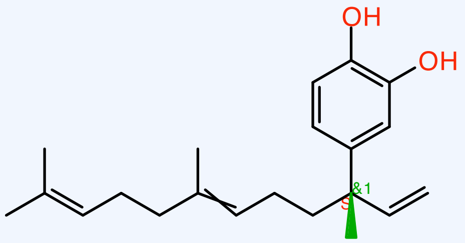	N293	HA	0.25
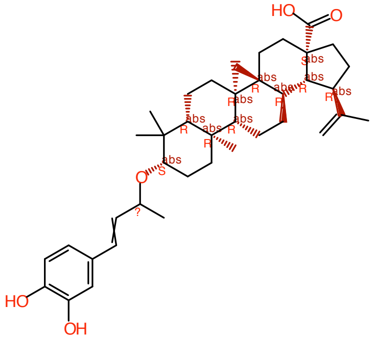	N323	HA	0
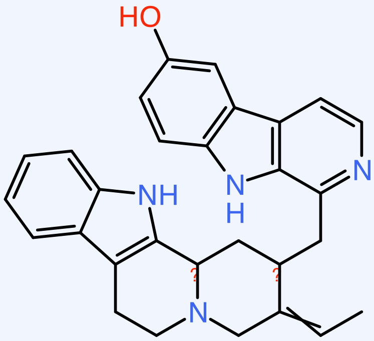	N236	HA	0.5
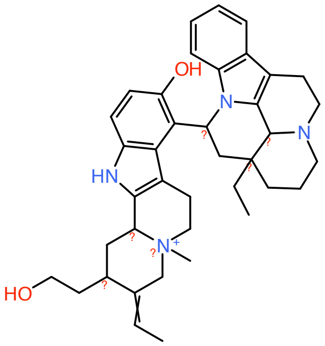	N128	HA	0.25
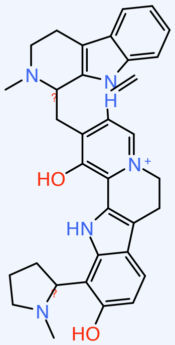	N262	HA	0.5

Structure of Smiles	ID	Activity_Status	Consensus_score
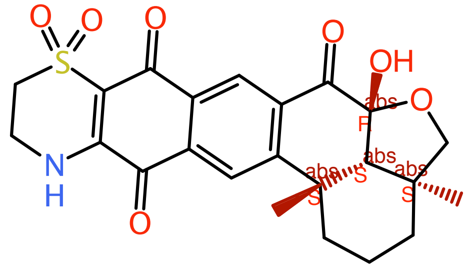	N7	HA	1
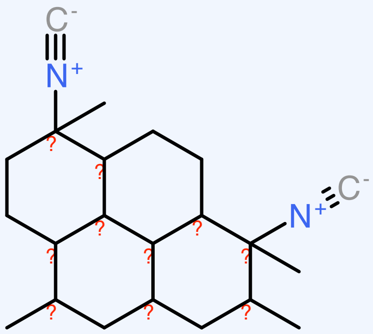	N104	HA	0.75
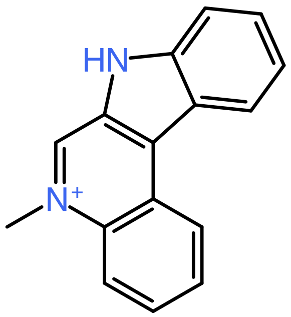	N183	HA	1
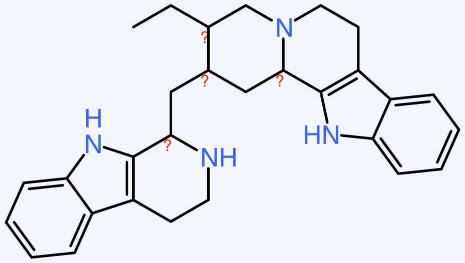	N166	HA	0.25
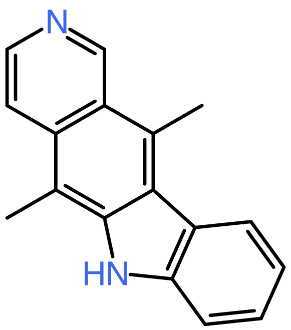	N138	HA	0.75

Structure of Smiles	ID	Activity_Status	Consensus_score
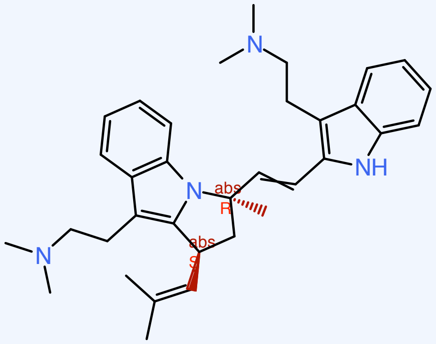	N145	HA	0.25
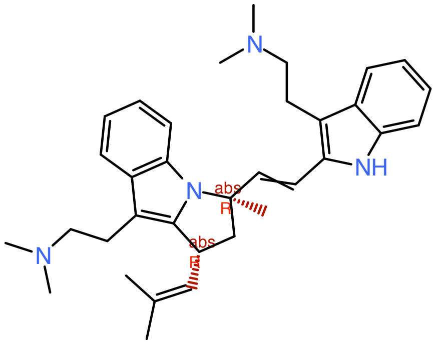	N146	HA	0.25
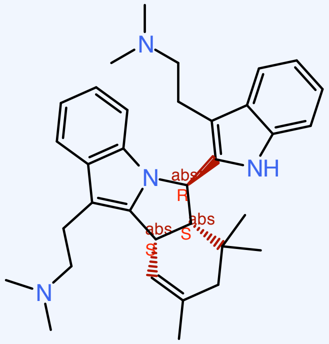	N112	HA	0
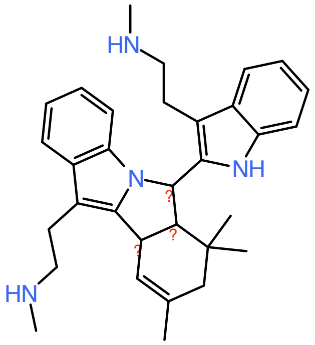	N147	HA	0.25
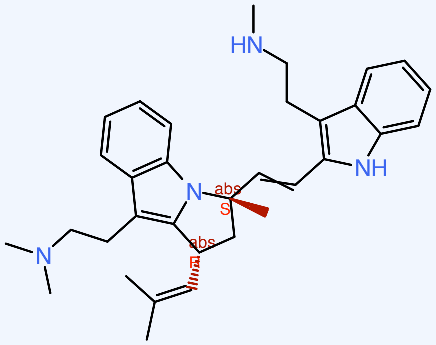	N140	HA	0.25

Structure of Smiles	ID	Activity_Status	Consensus_score
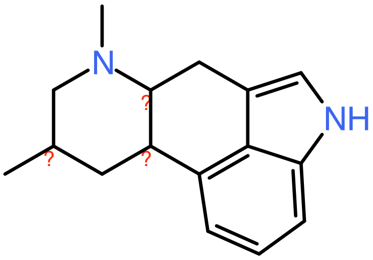	N259	HA	0.75
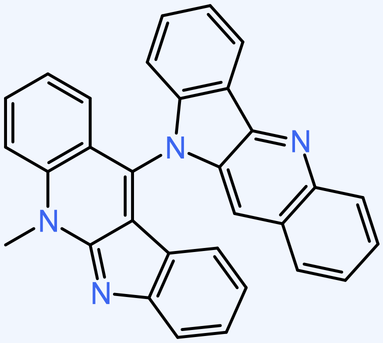	N312	HA	0.5
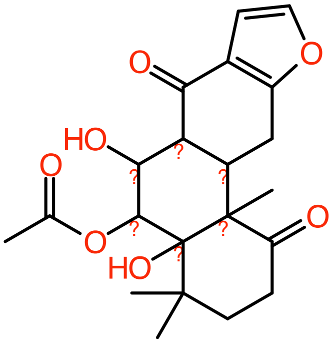	N165	HA	0.75
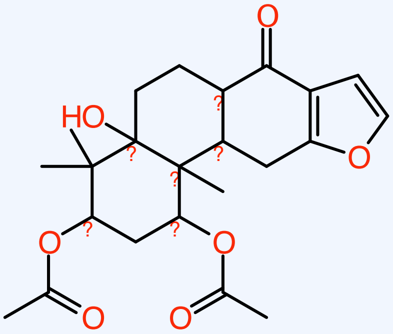	N204	HA	0.75
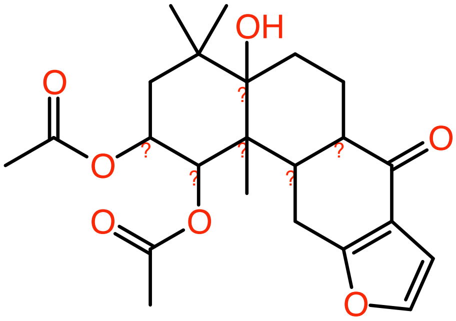	N308	HA	0.75

Structure of Smiles	ID	Activity_Status	Consensus_score
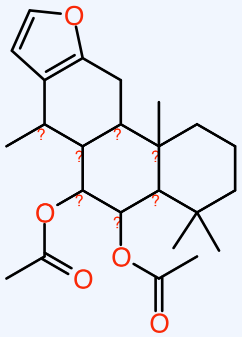	N110	HA	0.75
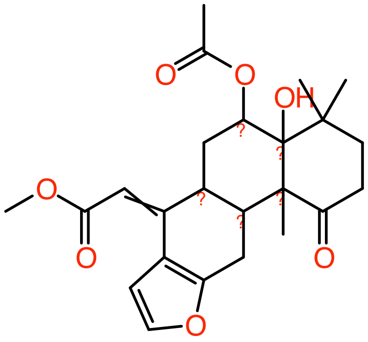	N289	HA	0.75
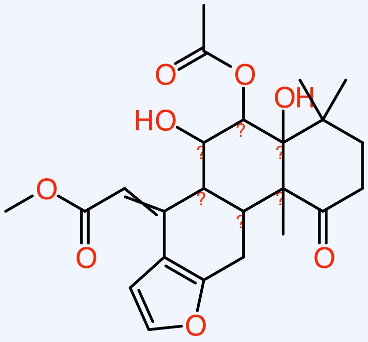	N197	HA	0.75
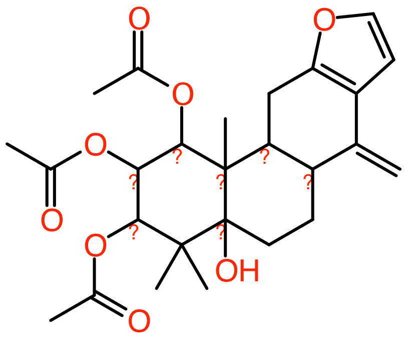	N184	HA	0.75
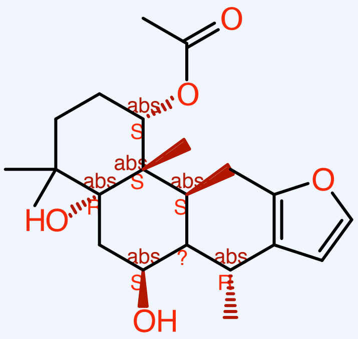	N244	HA	0.75

Structure of Smiles	ID	Activity_Status	Consensus_score
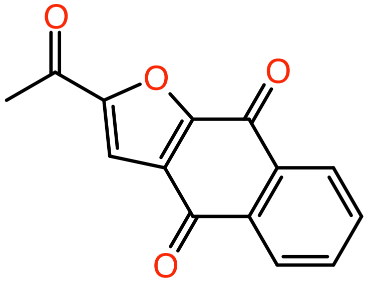	N85	HA	1
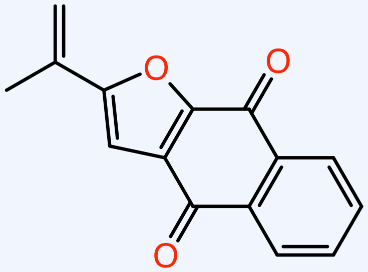	N168	HA	1
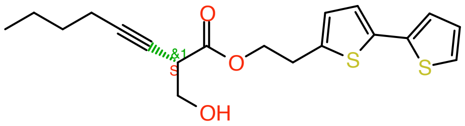	N131	HA	0.5
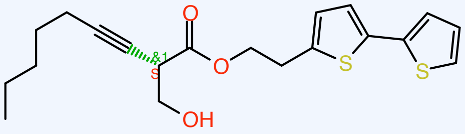	N132	HA	0.25
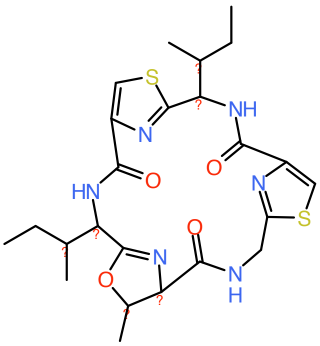	N296	HA	0.25

Structure of Smiles	ID	Activity_Status	Consensus_score
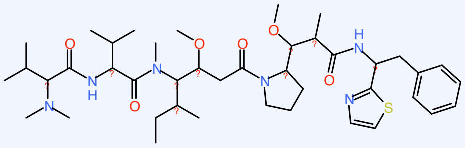	N81	HA	-0.5
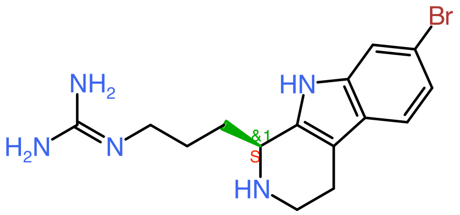	N18	MA	1
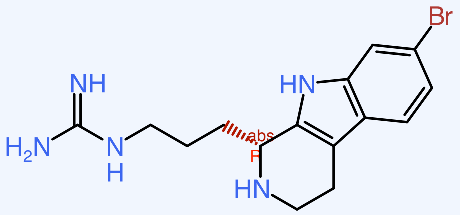	N679	MA	0.75
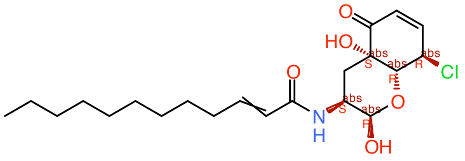	N669	MA	0.5
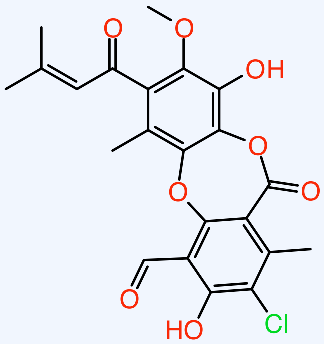	N792	MA	0.75

Structure of Smiles	ID	Activity_Status	Consensus_score
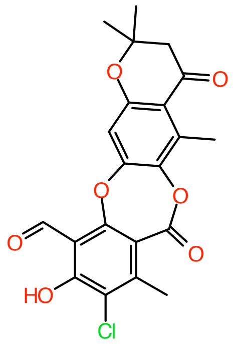	N852	MA	0.75
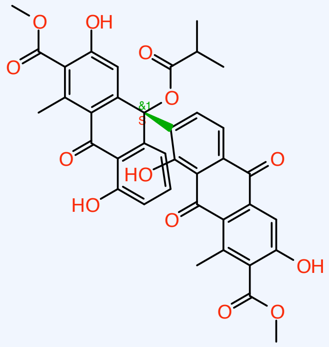	N820	MA	-0.5
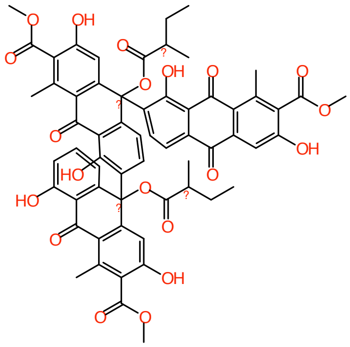	N661	MA	-1
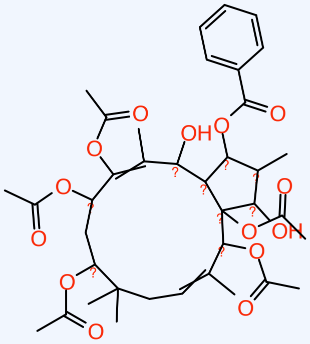	N717	MA	-0.5
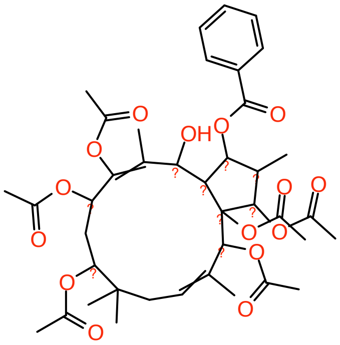	N703	MA	-0.5

Structure of Smiles	ID	Activity_Status	Consensus_score
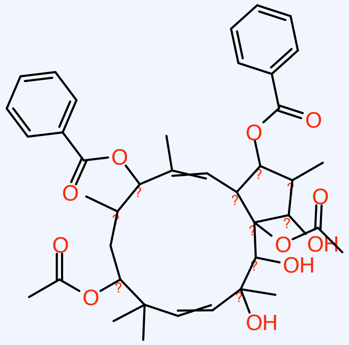	N711	MA	-0.75
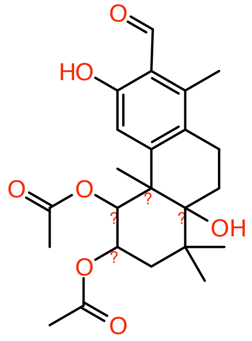	N657	MA	0.75
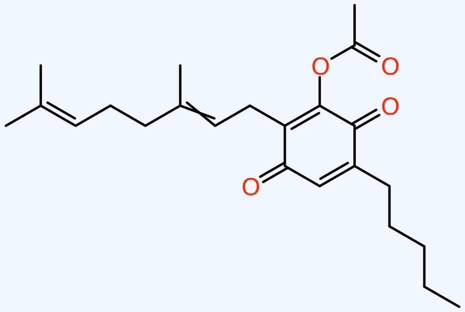	N781	MA	0
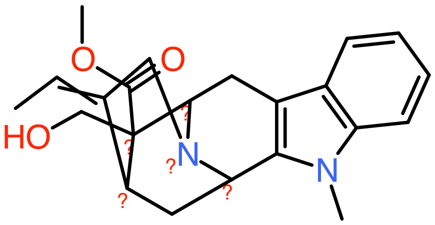	N783	MA	0.75
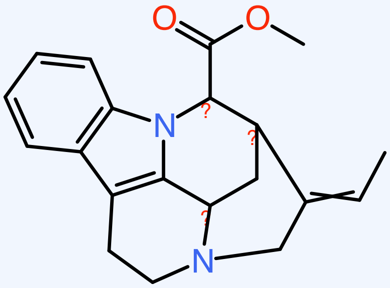	N753	MA	0.75

Structure of Smiles	ID	Activity_Status	Consensus_score
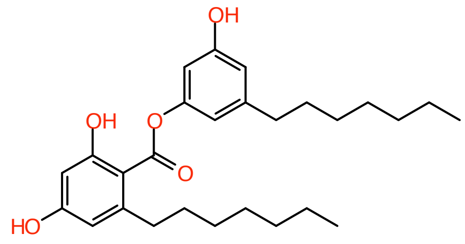	N891	MA	0
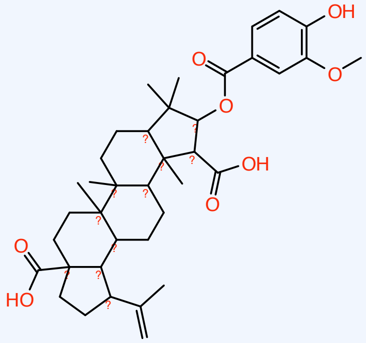	N704	MA	0
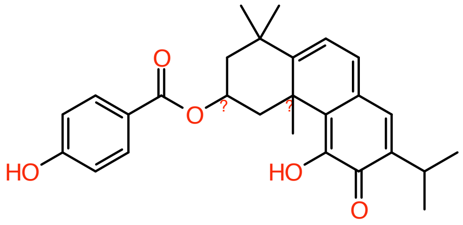	N677	MA	0.5
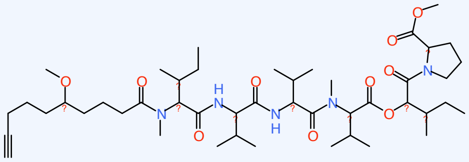	N706	MA	-0.25
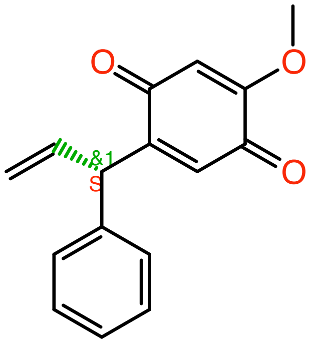	N707	MA	0.75

Structure of Smiles	ID	Activity_Status	Consensus_score
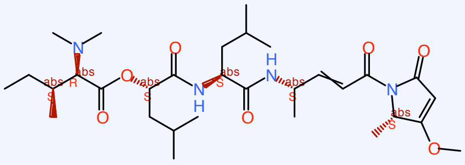	N845	MA	0.25
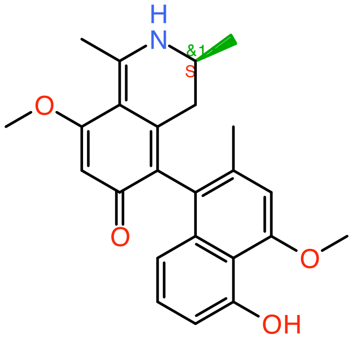	N793	MA	0.75
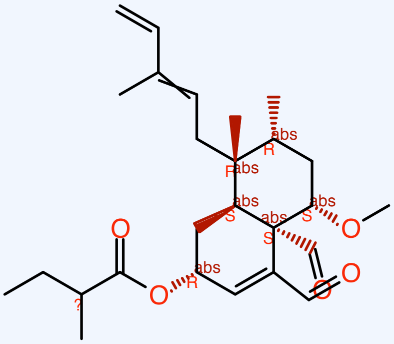	N735	MA	0.25
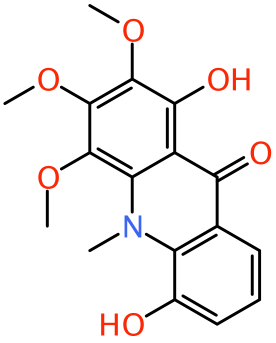	N738	MA	0.75
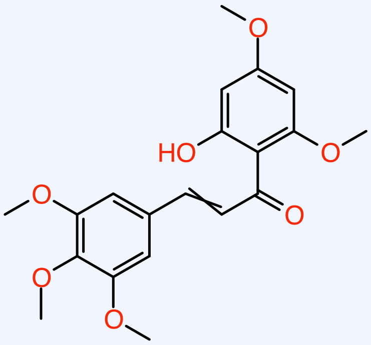	N774	MA	1

Structure of Smiles	ID	Activity_Status	Consensus_score
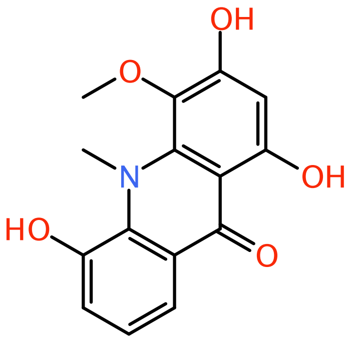	N861	MA	0.75
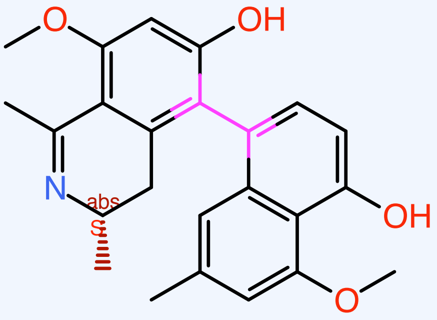	N681	MA	0.5
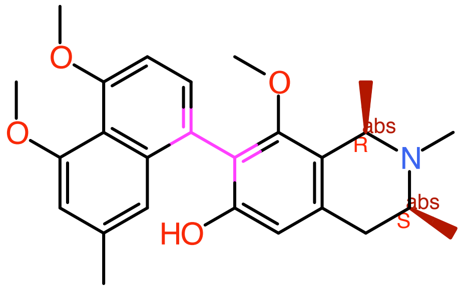	N662	MA	0.25
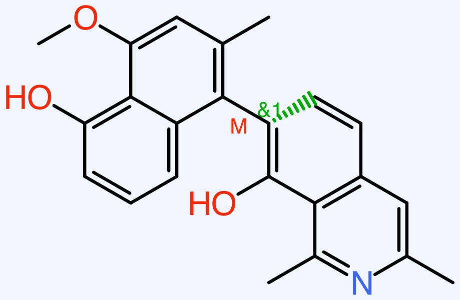	N853	MA	0.25
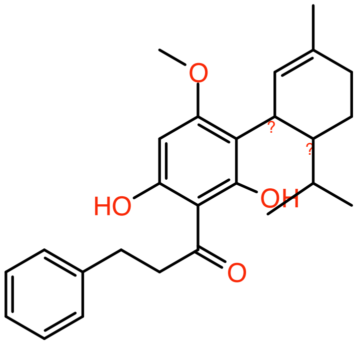	N693	MA	0.25

Structure of Smiles	ID	Activity_Status	Consensus_score
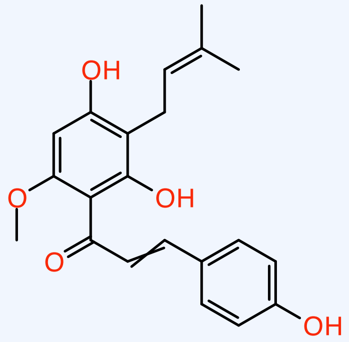	N838	MA	0.75
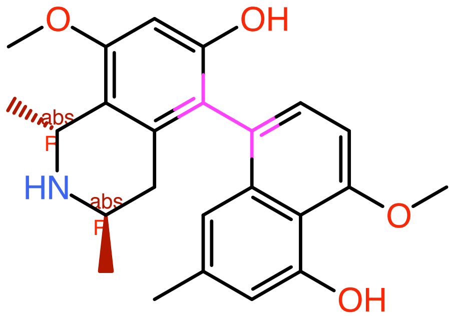	N665	MA	0.5
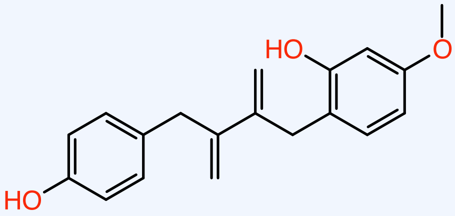	N866	MA	1
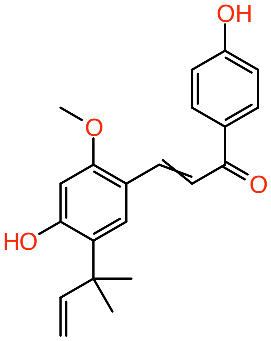	N713	MA	1
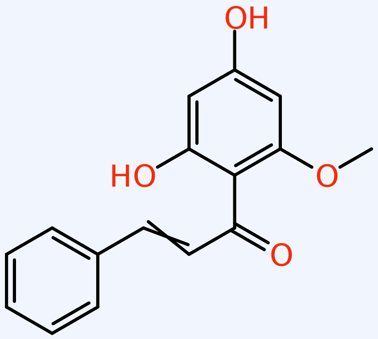	N876	MA	1

Structure of Smiles	ID	Activity_Status	Consensus_score
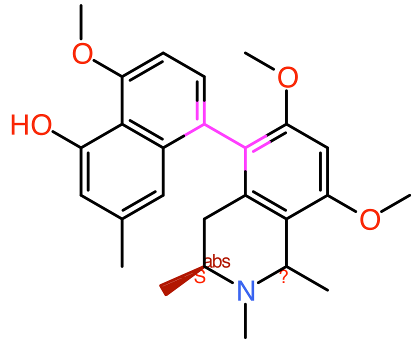	N742	MA	0.25
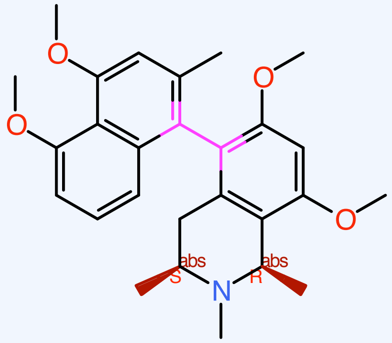	N864	MA	0.25
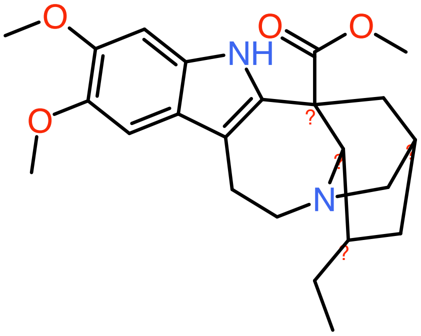	N688	MA	1
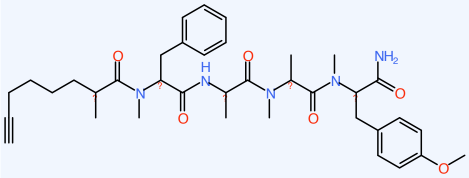	N725	MA	-0.25
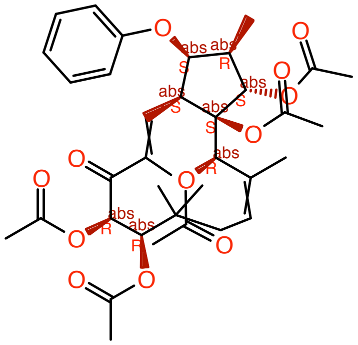	N664	MA	-0.5

Structure of Smiles	ID	Activity_Status	Consensus_score
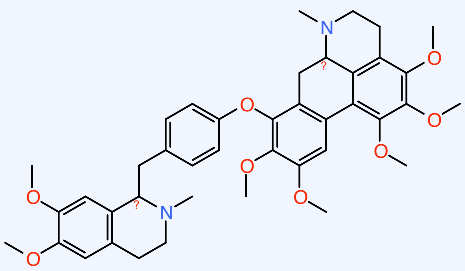	N798	MA	-0.5
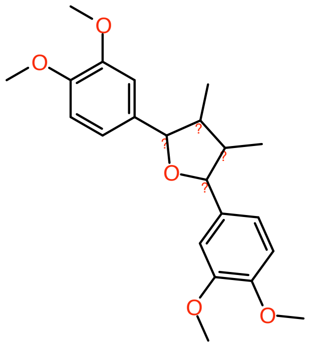	N842	MA	0.75
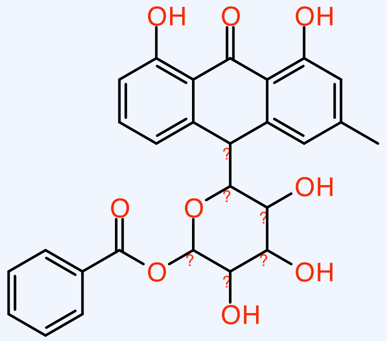	N667	MA	0.25
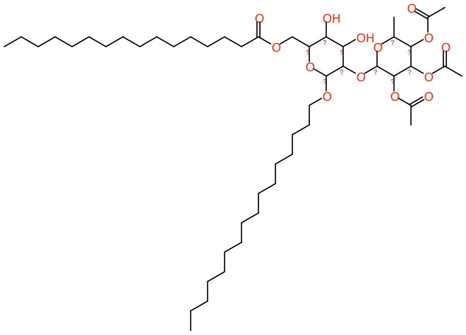	N836	MA	-0.5
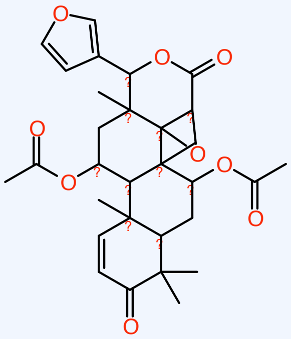	N701	MA	0.5

Structure of Smiles	ID	Activity_Status	Consensus_score
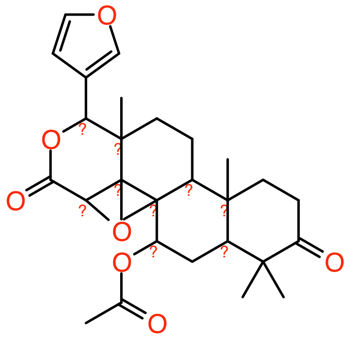	N682	MA	0.75
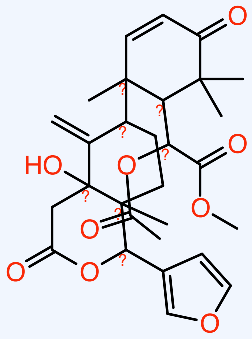	N721	MA	0.5
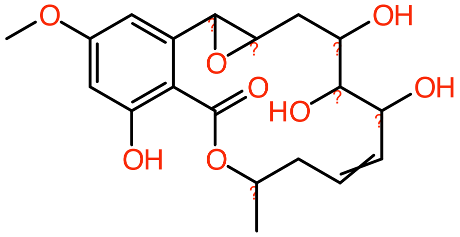	N780	MA	0.75
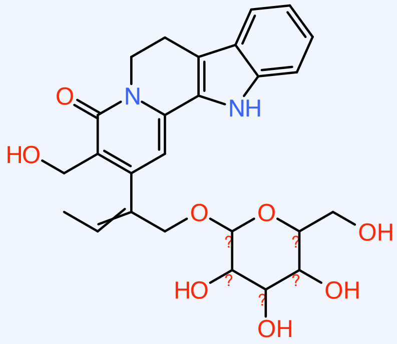	N885	MA	0.25
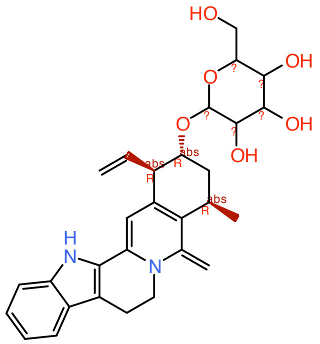	N823	MA	0.25

Structure of Smiles	ID	Activity_Status	Consensus_score
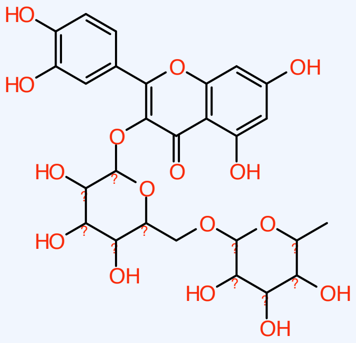	N785	MA	0
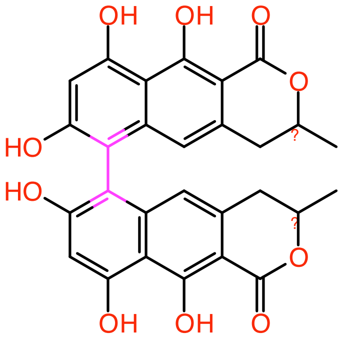	N796	MA	-0.5
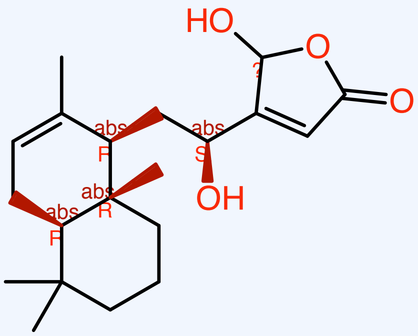	N788	MA	0.75
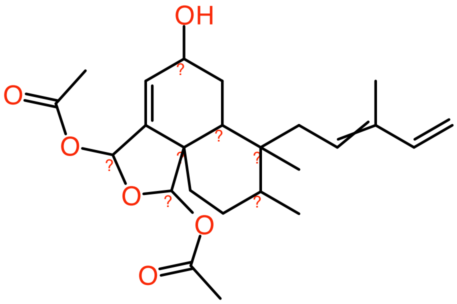	N821	MA	0.5
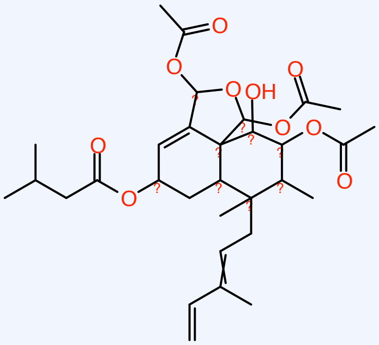	N673	MA	-0.5

Structure of Smiles	ID	Activity_Status	Consensus_score
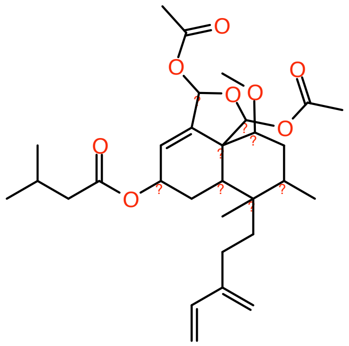	N686	MA	-0.25
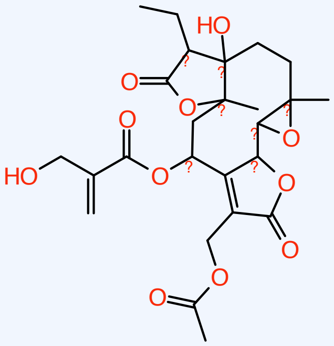	N710	MA	0.25
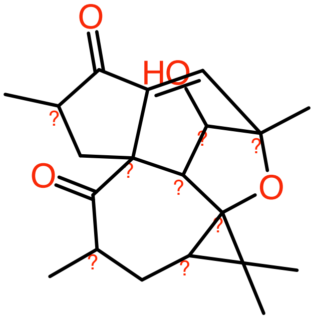	N871	MA	0.75
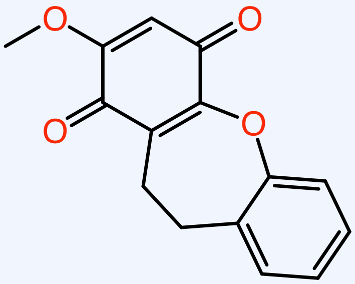	N709	MA	1
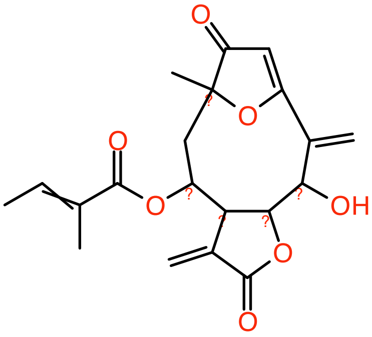	N692	MA	0.75

Structure of Smiles	ID	Activity_Status	Consensus_score
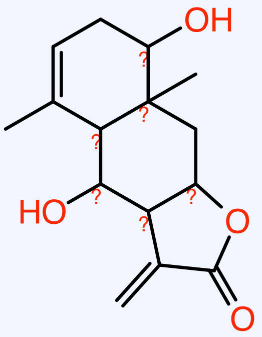	N888	MA	0.75
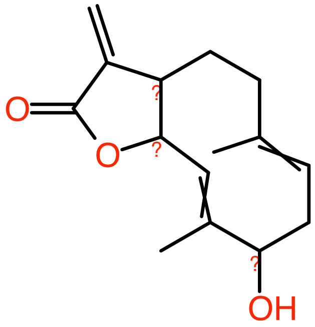	N886	MA	0.75
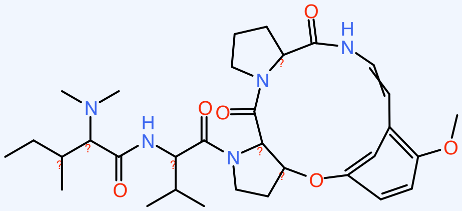	N712	MA	0.5
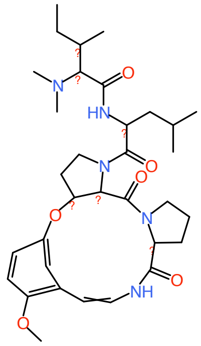	N751	MA	0.5
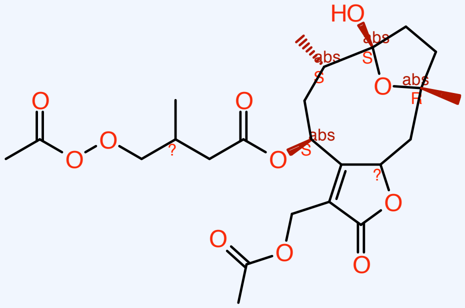	N685	MA	0

Structure of Smiles	ID	Activity_Status	Consensus_score
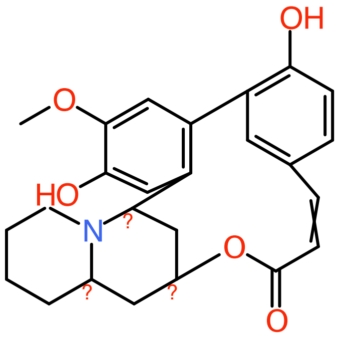	N768	MA	0.5
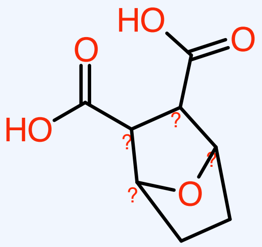	N816	MA	1
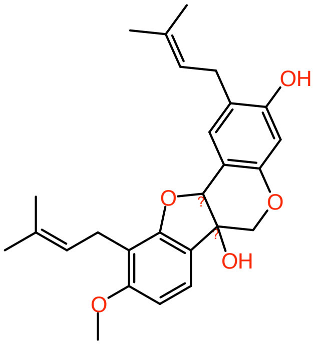	N865	MA	0.25
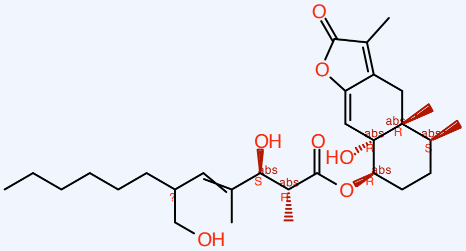	N680	MA	-0.25
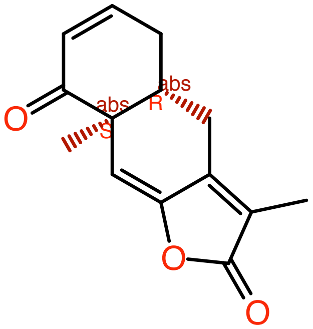	N801	MA	0.75

Structure of Smiles	ID	Activity_Status	Consensus_score
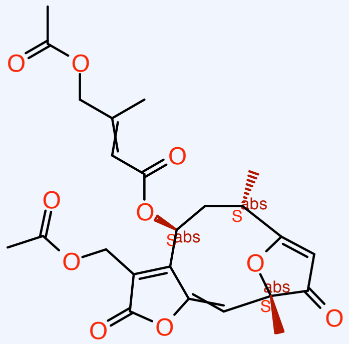	N684	MA	0.5
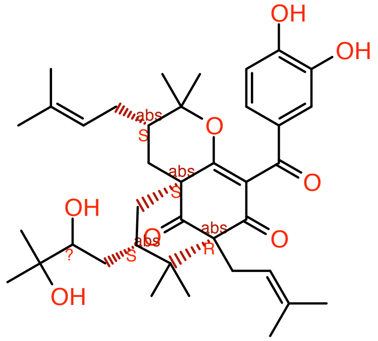	N656	MA	-0.25
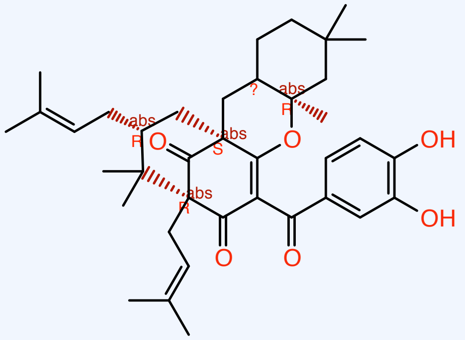	N652	MA	0
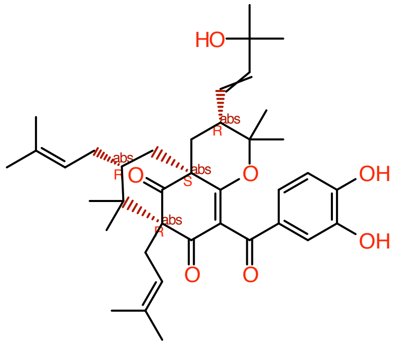	N654	MA	0
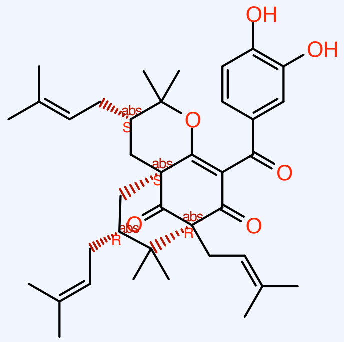	N651	MA	0

Structure of Smiles	ID	Activity_Status	Consensus_score
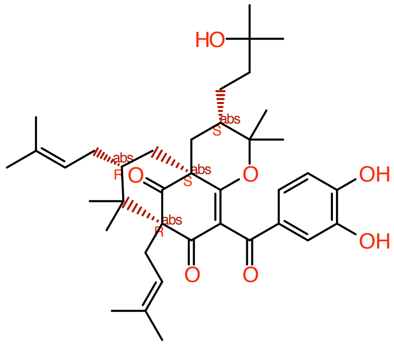	N655	MA	0
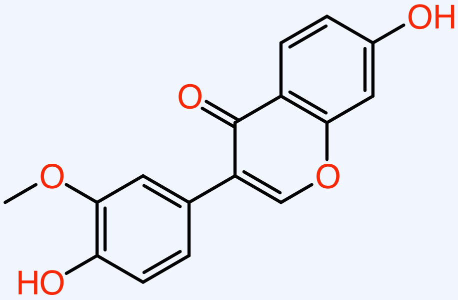	N771	MA	1
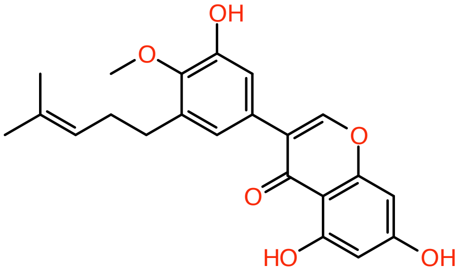	N858	MA	0.5
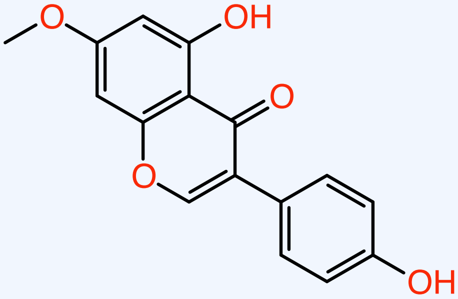	N848	MA	1
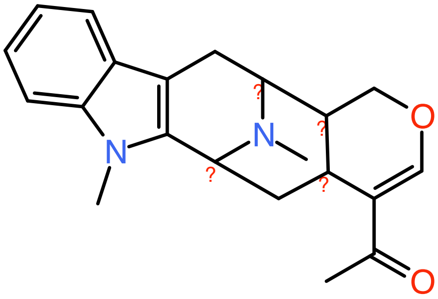	N884	MA	1

Structure of Smiles	ID	Activity_Status	Consensus_score
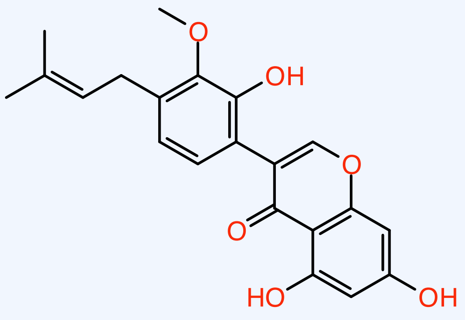	N748	MA	0.75
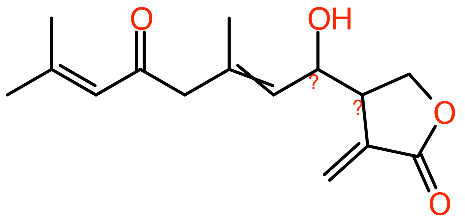	N805	MA	0.75
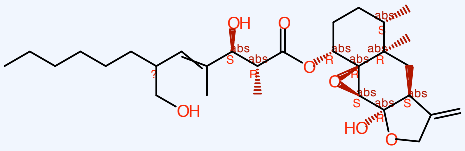	N715	MA	0
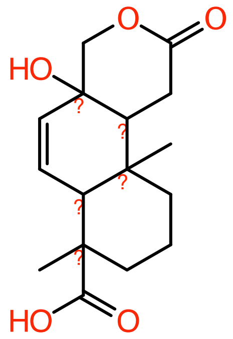	N835	MA	0.75
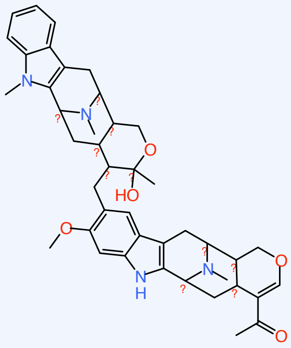	N863	MA	0.25

Structure of Smiles	ID	Activity_Status	Consensus_score
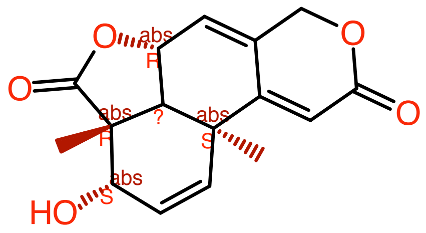	N830	MA	0.75
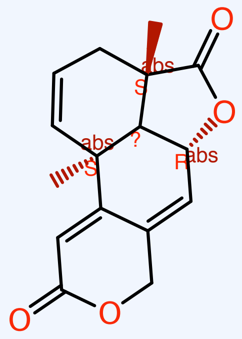	N833	MA	0.75
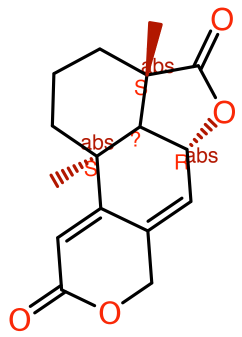	N834	MA	0.75
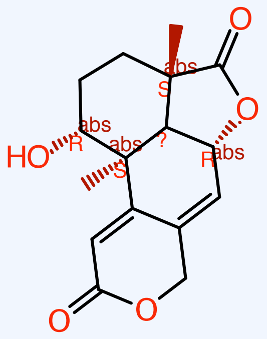	N831	MA	0.75
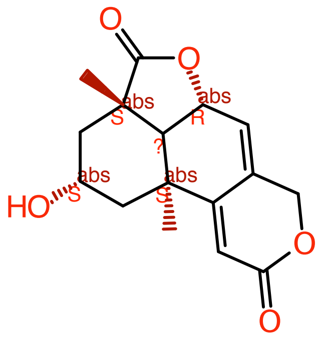	N828	MA	0.75

Structure of Smiles	ID	Activity_Status	Consensus_score
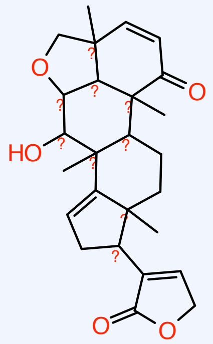	N663	MA	0.75
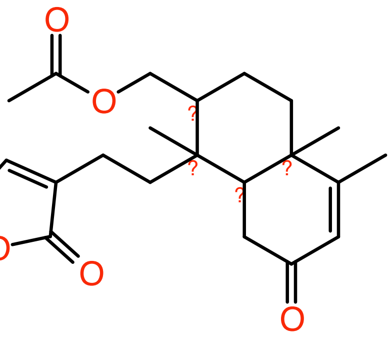	N887	MA	0.75
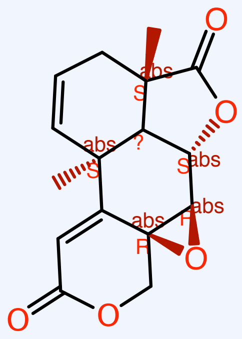	N832	MA	0.75
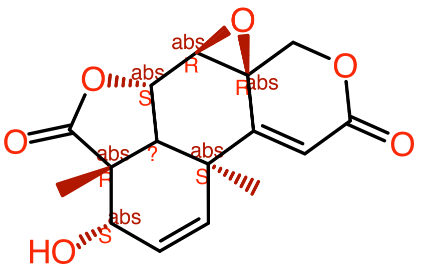	N829	MA	0.75
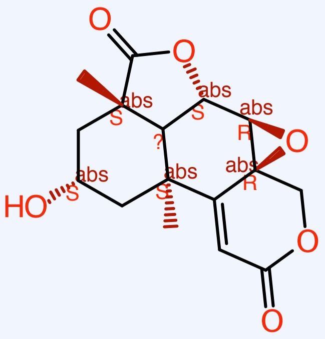	N827	MA	0.75

Structure of Smiles	ID	Activity_Status	Consensus_score
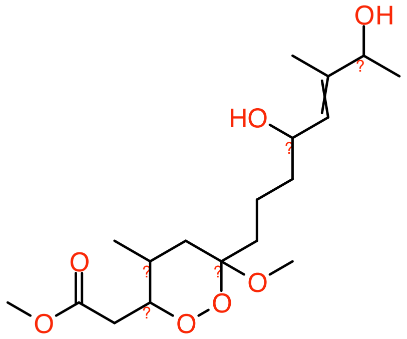	N659	MA	0.5
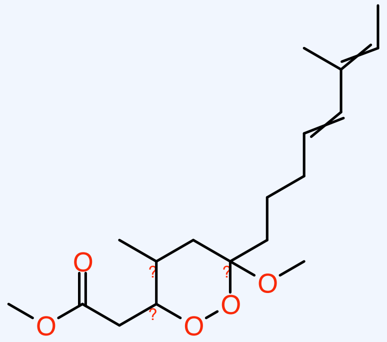	N727	MA	0.75
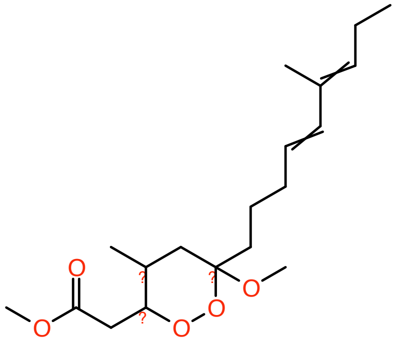	N770	MA	0
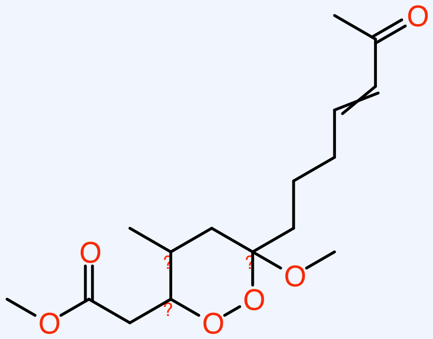	N660	MA	1
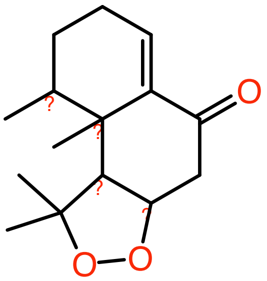	N689	MA	0.75

Structure of Smiles	ID	Activity_Status	Consensus_score
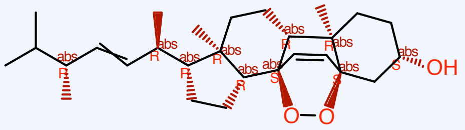	N744	MA	0.25
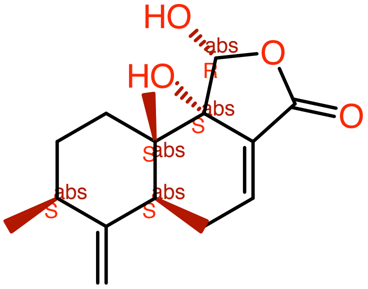	N752	MA	0.75
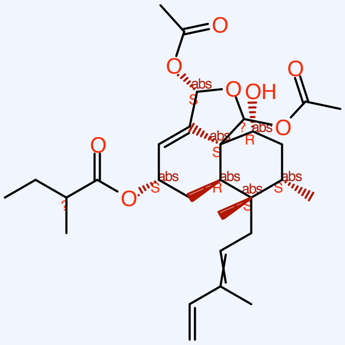	N716	MA	-0.25
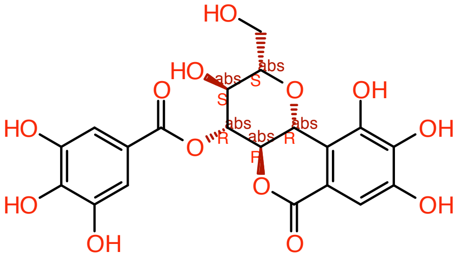	N844	MA	0
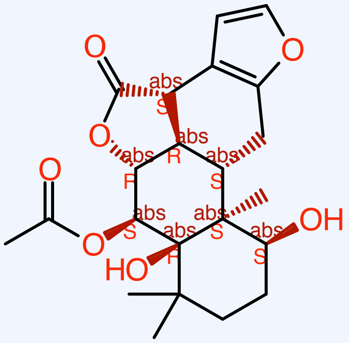	N674	MA	0.75

Structure of Smiles	ID	Activity_Status	Consensus_score
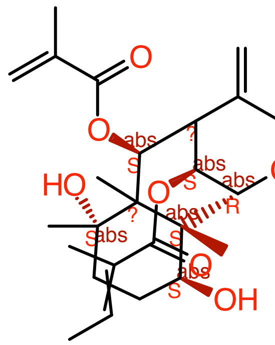	N868	MA	0.75
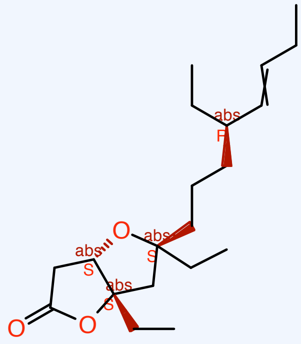	N856	MA	0.5
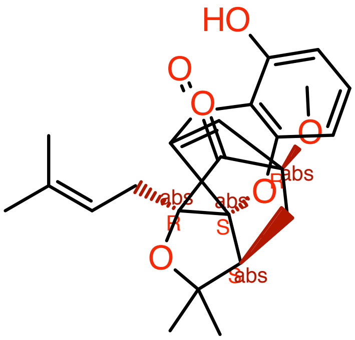	N745	MA	1
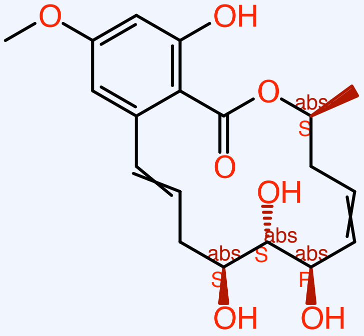	N782	MA	0.75
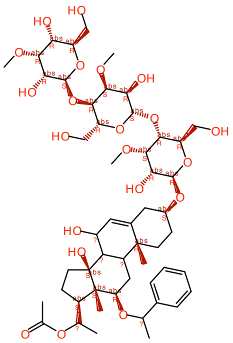	N757	MA	-0.25

Structure of Smiles	ID	Activity_Status	Consensus_score
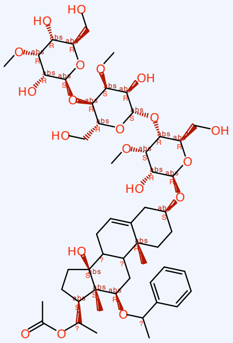	N799	MA	-0.25
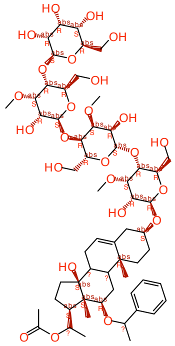	N802	MA	-0.25
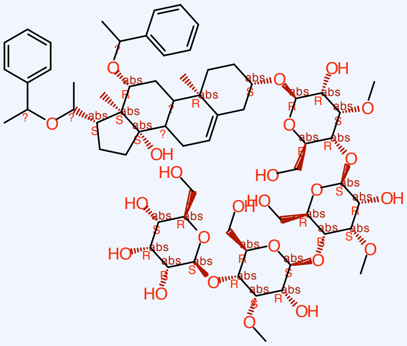	N698	MA	-0.25
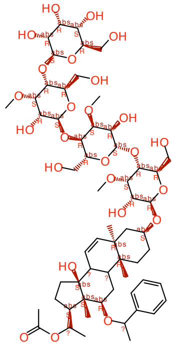	N883	MA	-0.25
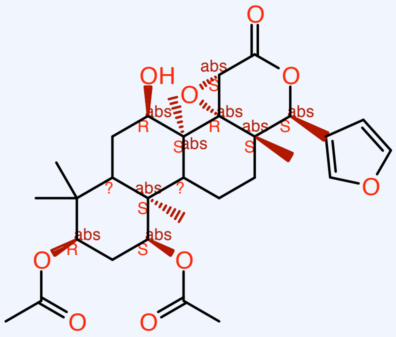	N875	MA	0.5

Structure of Smiles	ID	Activity_Status	Consensus_score
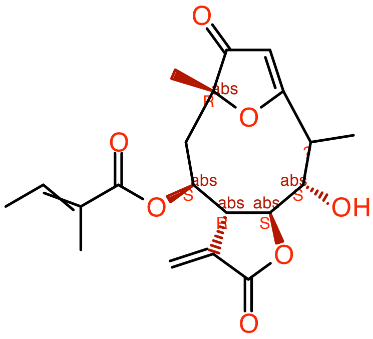	N824	MA	0.75
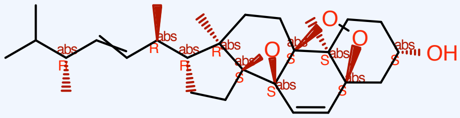	N786	MA	0.25
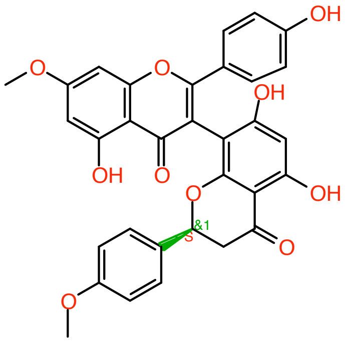	N733	MA	-0.25
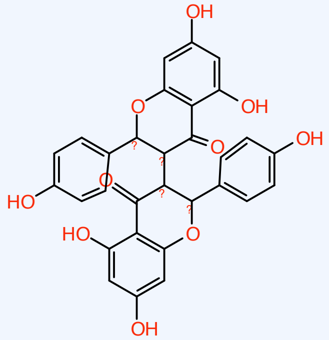	N795	MA	-0.5
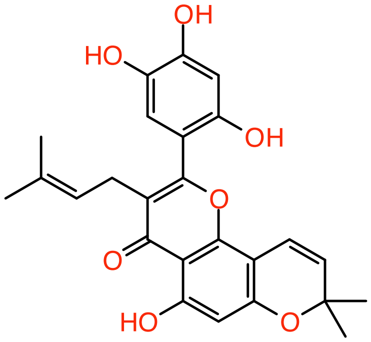	N749	MA	0.25

Structure of Smiles	ID	Activity_Status	Consensus_score
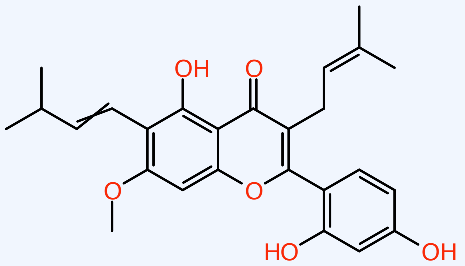	N773	MA	0.25
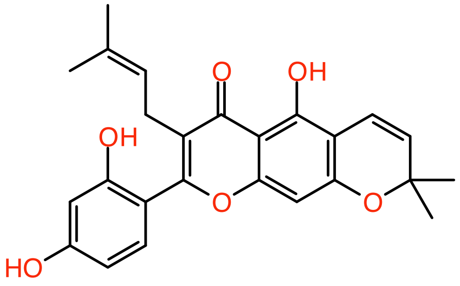	N672	MA	0.25
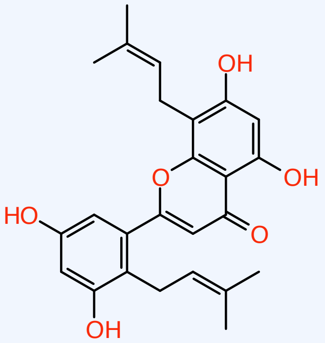	N759	MA	0.25
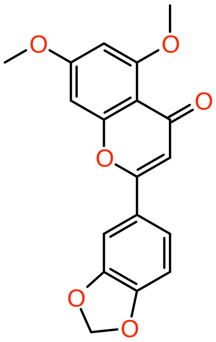	N806	MA	1
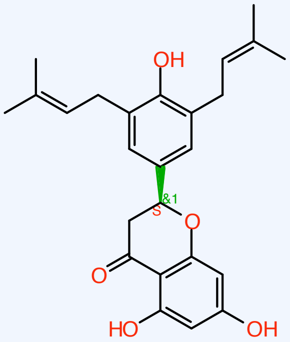	N737	MA	0.5

Structure of Smiles	ID	Activity_Status	Consensus_score
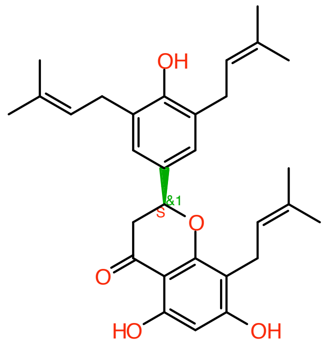	N676	MA	0.25
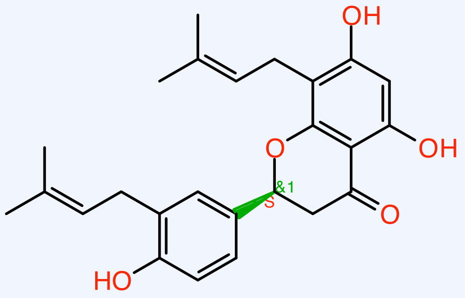	N869	MA	0.25
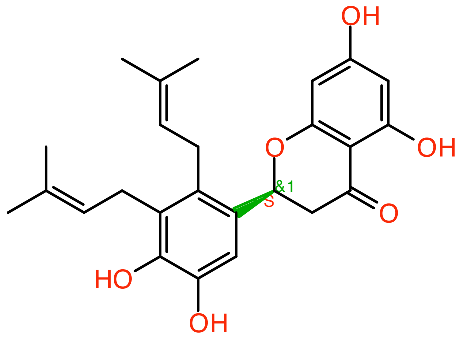	N671	MA	0.25
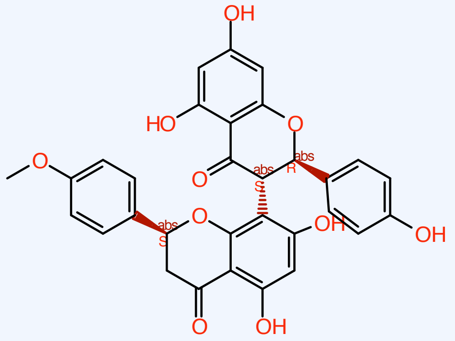	N734	MA	-0.5
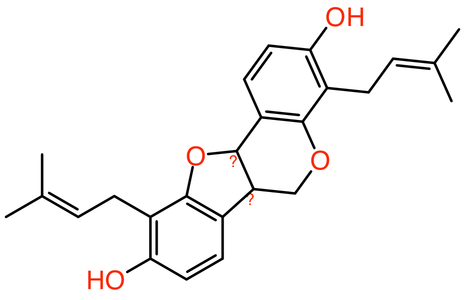	N855	MA	0.25

Structure of Smiles	ID	Activity_Status	Consensus_score
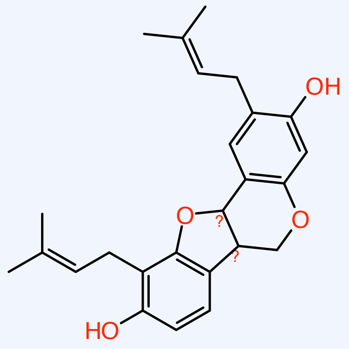	N756	MA	0.25
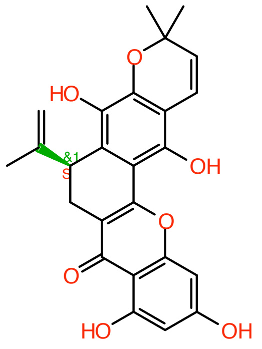	N819	MA	0.25
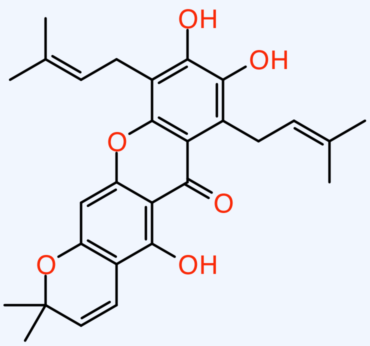	N719	MA	0.25
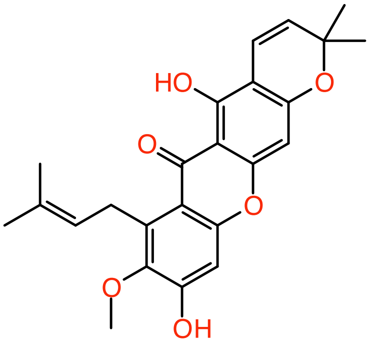	N731	MA	0.25
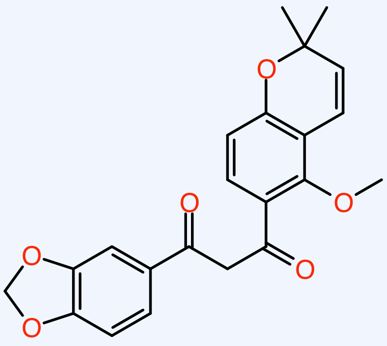	N878	MA	1

Structure of Smiles	ID	Activity_Status	Consensus_score
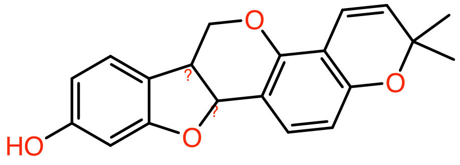	N761	MA	0.75
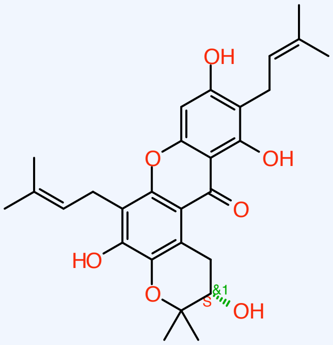	N718	MA	0.25
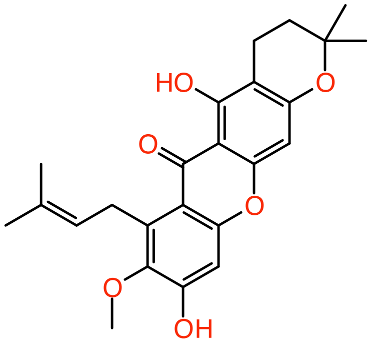	N732	MA	0.25
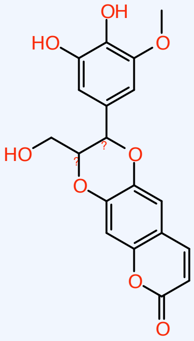	N687	MA	1
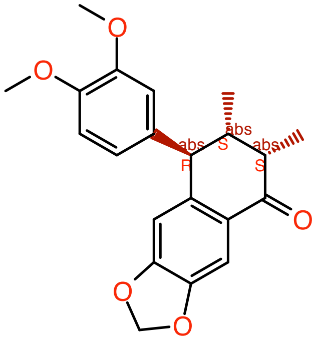	N826	MA	1

Structure of Smiles	ID	Activity_Status	Consensus_score
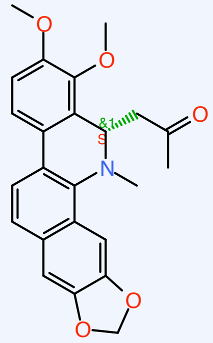	N699	MA	0.5
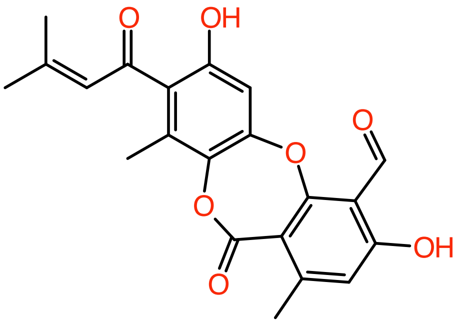	N779	MA	0.75
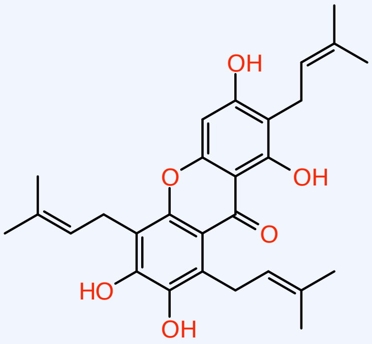	N722	MA	0.25
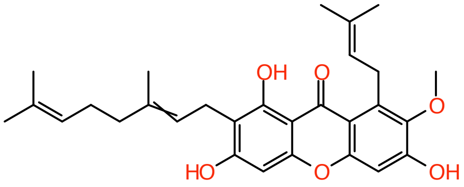	N720	MA	0.25
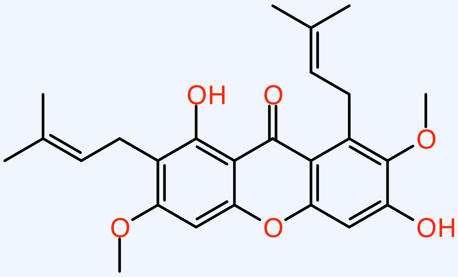	N784	MA	0.25

Structure of Smiles	ID	Activity_Status	Consensus_score
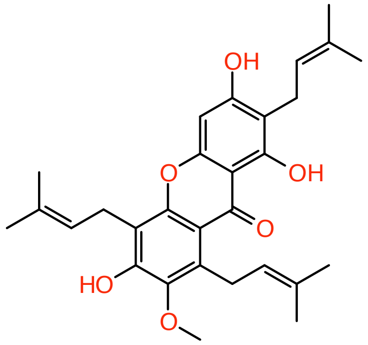	N675	MA	0.25
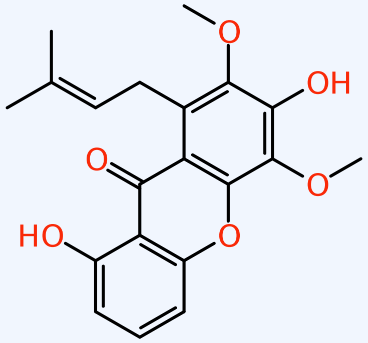	N862	MA	0.5
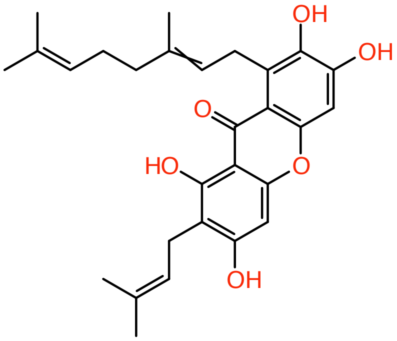	N746	MA	0.25
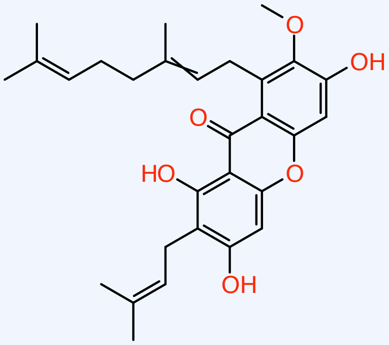	N743	MA	0.25
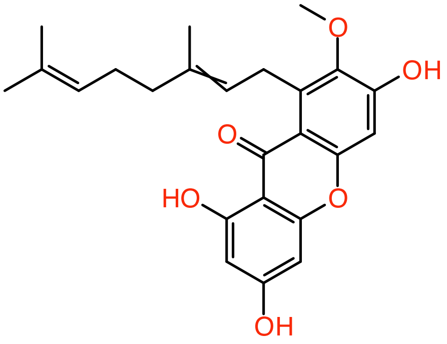	N729	MA	0.25

Structure of Smiles	ID	Activity_Status	Consensus_score
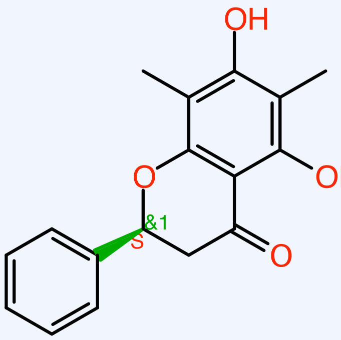	N877	MA	0.75
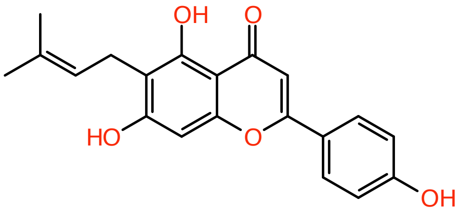	N766	MA	0.75
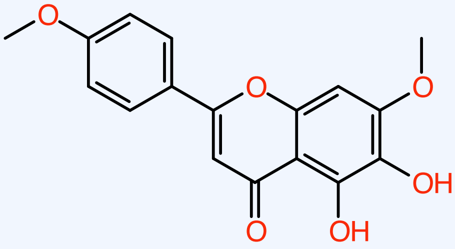	N867	MA	1
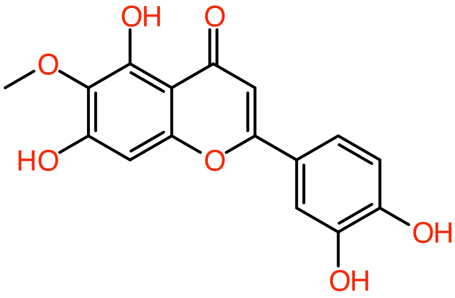	N763	MA	1
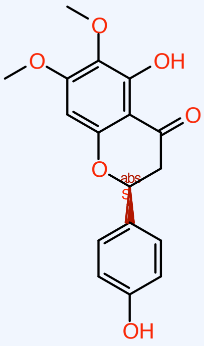	N860	MA	1

Structure of Smiles	ID	Activity_Status	Consensus_score
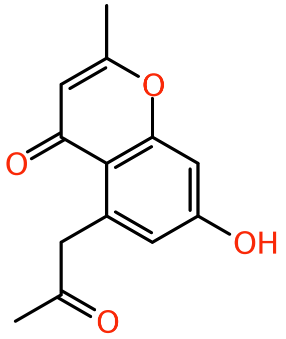	N850	MA	0.75
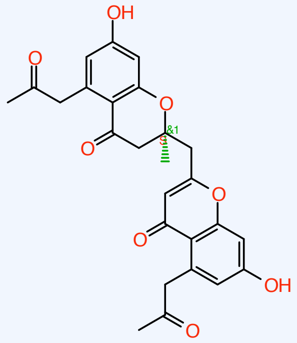	N690	MA	0.5
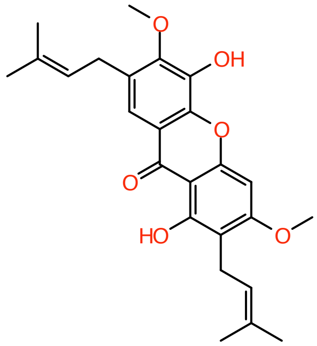	N794	MA	0.25
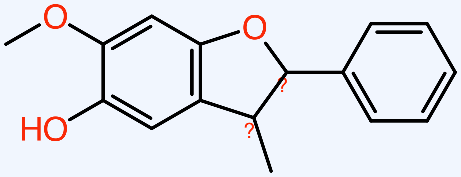	N857	MA	0.75
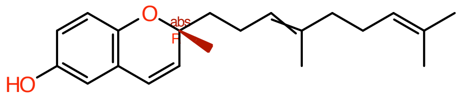	N879	MA	0.25

Structure of Smiles	ID	Activity_Status	Consensus_score
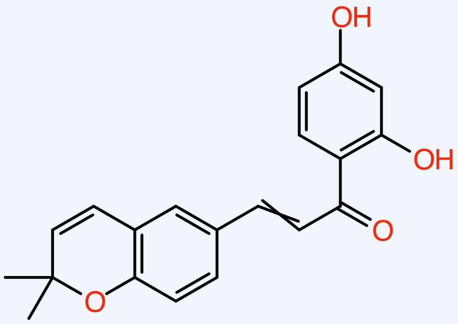	N882	MA	1
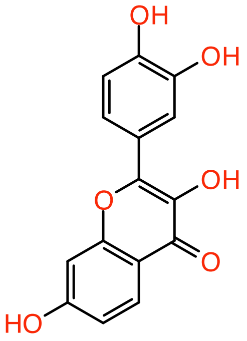	N837	MA	1
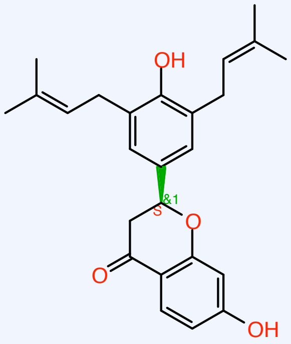	N808	MA	0.5
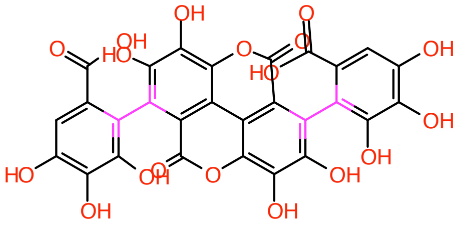	N803	MA	-0.25
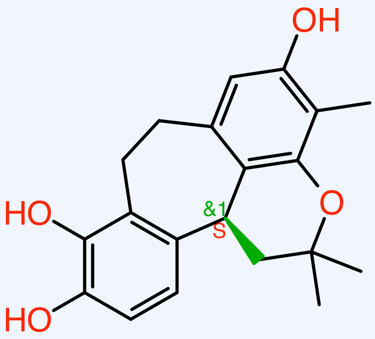	N741	MA	0.75

Structure of Smiles	ID	Activity_Status	Consensus_score
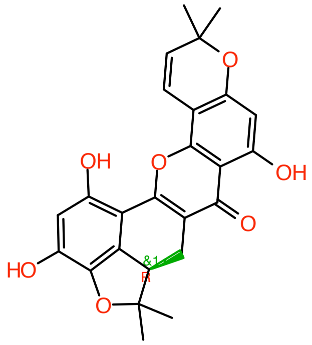	N847	MA	0.5
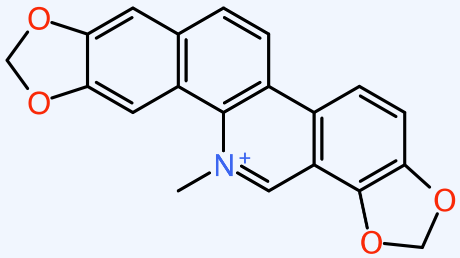	N822	MA	0.75
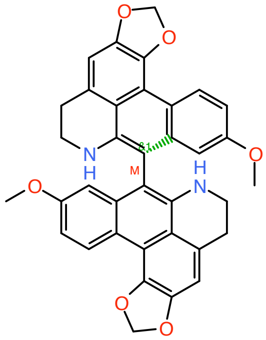	N814	MA	0
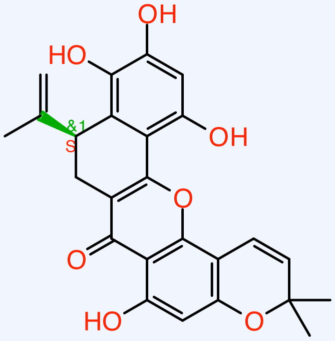	N772	MA	0.25
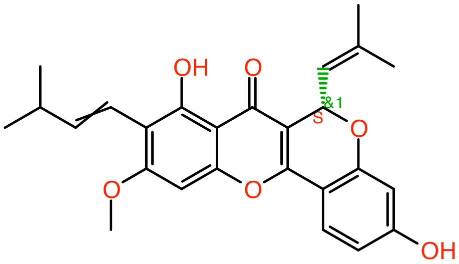	N890	MA	0.25

Structure of Smiles	ID	Activity_Status	Consensus_score
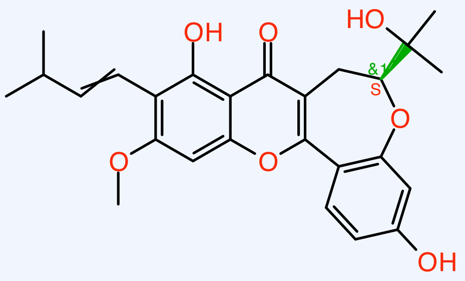	N813	MA	0.5
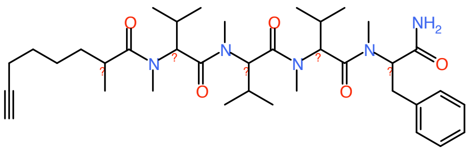	N809	MA	0
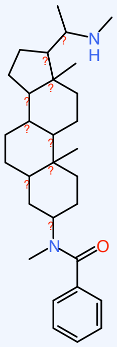	N760	MA	0.25
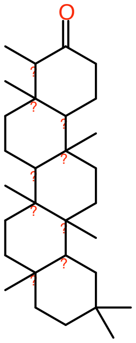	N812	MA	0.25
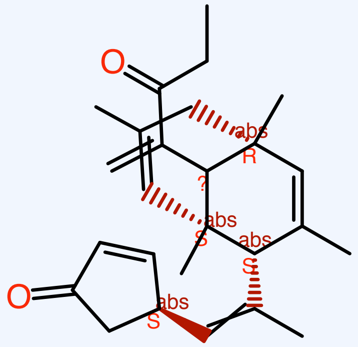	N666	MA	0.25

Structure of Smiles	ID	Activity_Status	Consensus_score
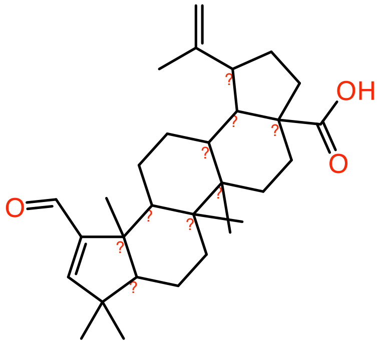	N758	MA	0.25
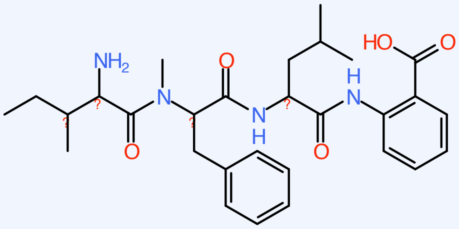	N825	MA	0.25
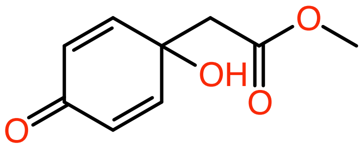	N797	MA	1
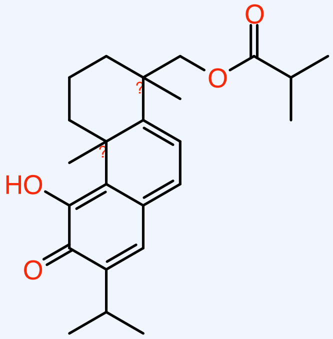	N724	MA	0.5
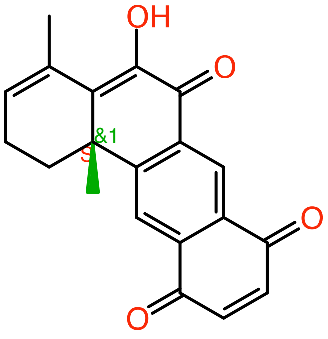	N872	MA	0.75

Structure of Smiles	ID	Activity_Status	Consensus_score
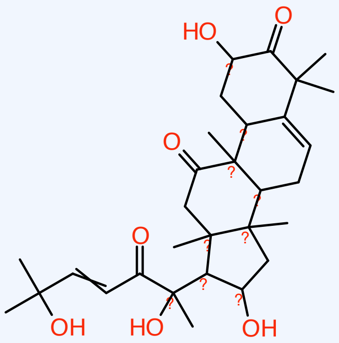	N811	MA	0.5
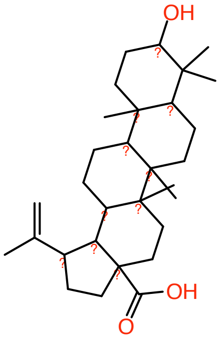	N889	MA	0.25
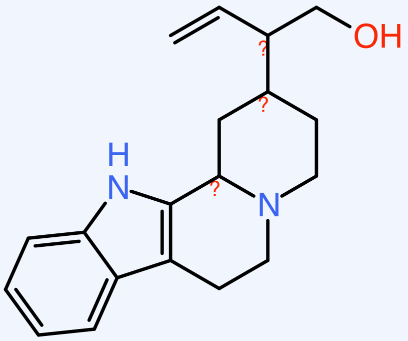	N804	MA	0.75
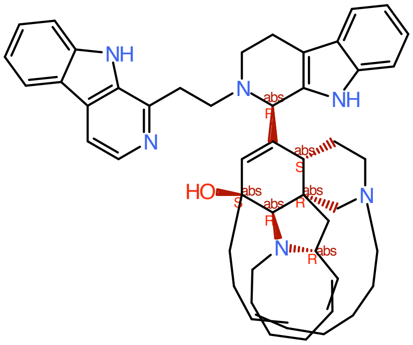	N880	MA	0
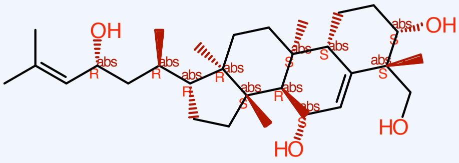	N854	MA	0.25

Structure of Smiles	ID	Activity_Status	Consensus_score
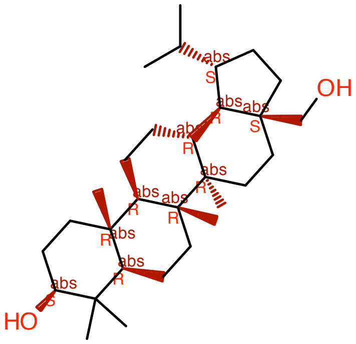	N787	MA	0.25
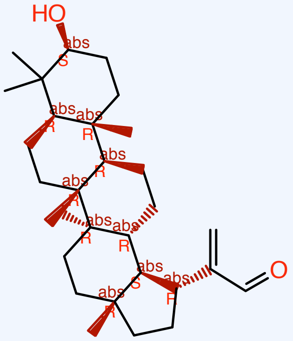	N789	MA	0.25
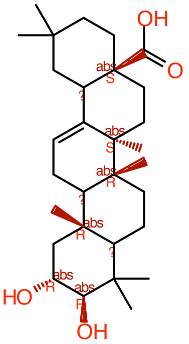	N851	MA	0.25
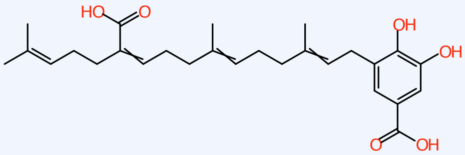	N775	MA	0
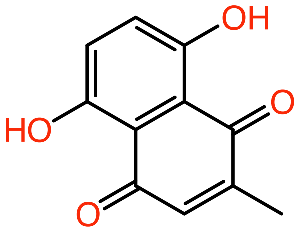	N702	MA	0.75

Structure of Smiles	ID	Activity_Status	Consensus_score
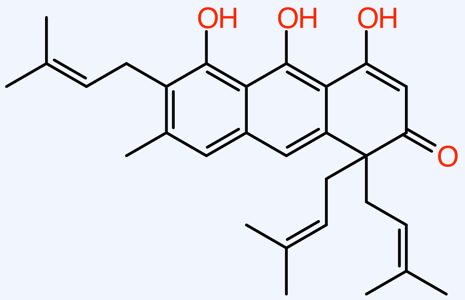	N658	MA	0.25
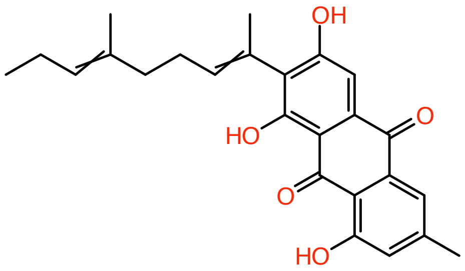	N678	MA	0.25
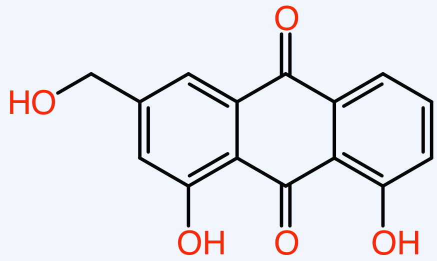	N14	MA	1
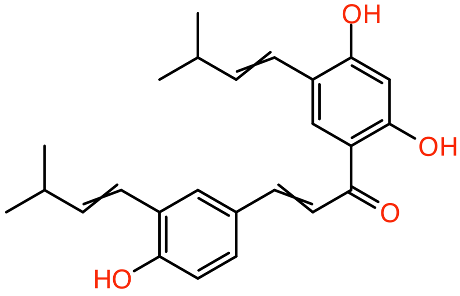	N670	MA	0.25
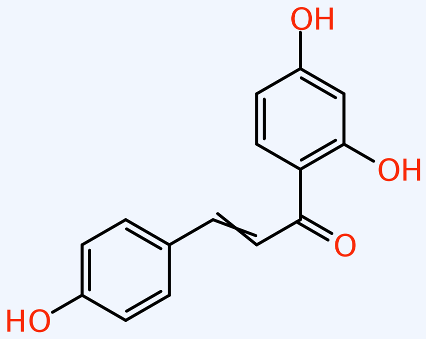	N815	MA	1

Structure of Smiles	ID	Activity_Status	Consensus_score
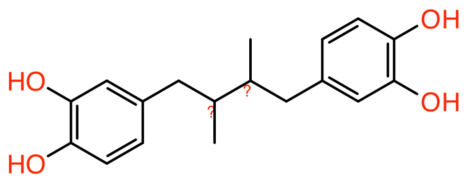	N810	MA	1
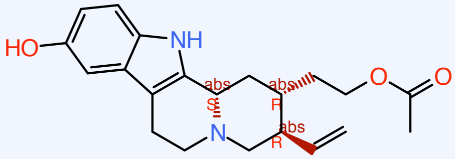	N653	MA	0.75
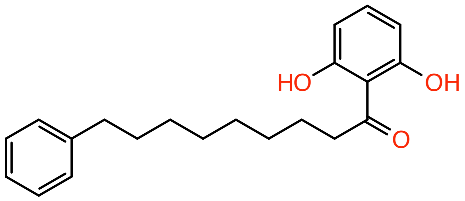	N849	MA	0.25
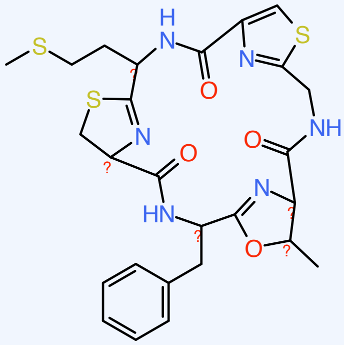	N747	MA	0.25
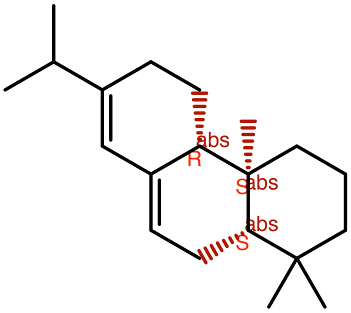	N800	MA	0.5

Structure of Smiles	ID	Activity_Status	Consensus_score
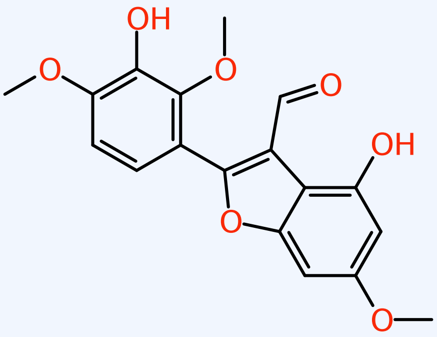	N740	MA	0.75
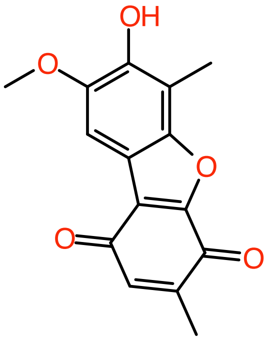	N764	MA	0.75
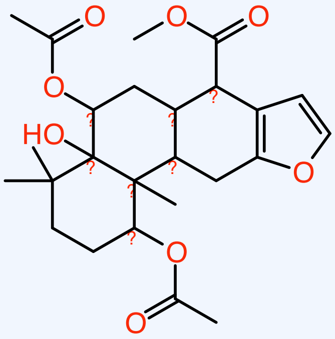	N754	MA	0.75
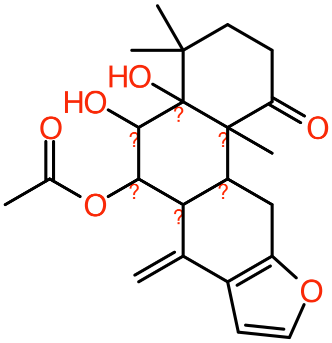	N705	MA	0.75
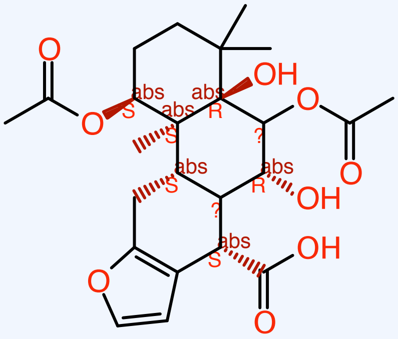	N776	MA	0.5

Structure of Smiles	ID	Activity_Status	Consensus_score
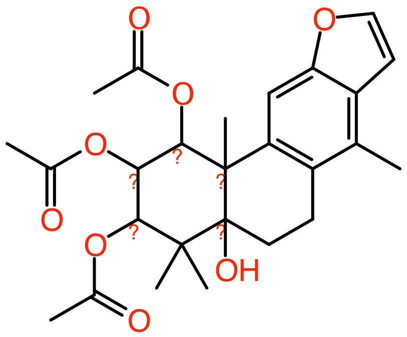	N755	MA	0.5
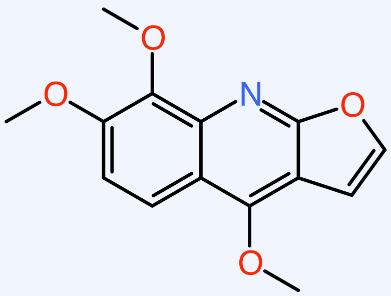	N874	MA	1
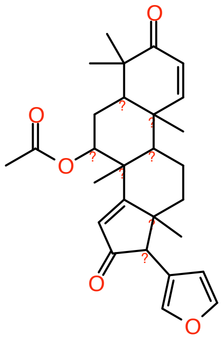	N750	MA	0.5
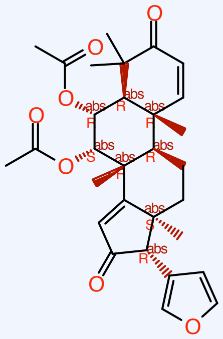	N700	MA	0.25
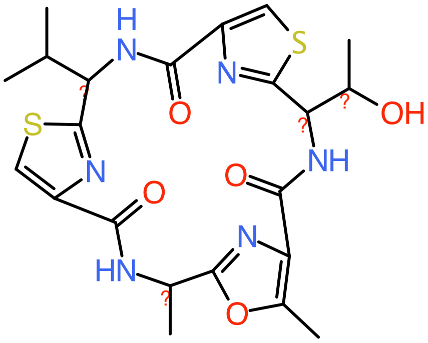	N694	MA	0.25
